# Supplementary material for: Catalytic atroposelective synthesis of axially chiral benzonitriles via chirality control during bond dissociation and CN group formation
Source: Nat Commun. 2022 Jan 10;13:36. doi: 10.1038/s41467-021-27813-4 (PMC8748609; doi:10.1038/s41467-021-27813-4)
Supplement: Supplementary file 1 — Supplementary Information [file 41467_2021_27813_MOESM1_ESM.pdf]

Supplementary Information

Catalytic Atroposelective Synthesis of Axially Chiral Benzonitriles via Chirality Control during Bond Dissociatoin and CN Group Formation

Ya Lv,[1] Guoyong Luo,[2] Qian Liu,[1] Zhichao Jin,[1]* Xinglong Zhang[4]* and Yonggui Robin Chi[1,3]*

[1]State Key Laboratory Breeding Base of Green Pesticide and Agricultural Bioengineering, Key Laboratory of Green Pesticide and Agricultural Bioengineering, Ministry of Education, Guizhou University, Huaxi District, Guiyang 550025, China.

[2]School of Pharmacy, Guizhou University of Traditional Chinese Medicine, Huaxi District, Guiyang 550025, China.

[3]Division of Chemistry & Biological Chemistry, School of Physical & Mathematical Sciences, Nanyang Technological University, Singapore 637371, Singapore.

[4]Institute of High Performance Computing, A*STAR (Agency for Science, Technology and Research), Singapore 138632, Singapore.

*Corresponding authors e-mails:

zcjin@gzu.edu.cn

Zhang_Xinglong@ihpc.a-star.edu.sg

robinchi@ntu.edu.sg.

Table of Contents

Supplementary Methods 4

General information 4

Synthesis of substrates...... 5

Synthesis of NHC-G...... 7

Condition optimization...... 8

General procedure for the catalytic reactions...... 10

Synthetic transformations of axially chiral products and their applications...... 10

X-ray crystallography of compounds **3a**, **11** and NHC-**G**...... 17

Density functional theory (DFT) calculations...... 19

Computational methods......19

Model system calculation......20

Key steps and key transition state structures for the full reaction......23

Rotational barriers for atropisomers......25

Optimised structures and absolute energies, zero-point energies......26

Characterization of substrates and products 32

Supplementary References 180

Supplementary Tables

Supplementary Table 1. Condition optimization for the synthesis of **3a** 8

Supplementary Table 2. Condition optimization for the synthesis of **3g** 9

Supplementary Table 3. X-ray crystallography of compounds **3a**, **11** and NHC-**G**......17

Supplementary Table 4. Optimised structures and absolute energies, zero-point energies 27

Supplementary Table 5. Raw energy values obtained at SMD(toluene)-DLPNO-CCSD(T)/cc-pV(DT)Z basis sets and the complete basis set (CBS) extrapolation……28

Supplementary Table 6. Raw energy values obtained at SMD(toluene)-DLPNO-CCSD(T)/aug-cc-pV(DT)Z basis sets and the complete basis set (CBS) extrapolation……………………………………………………30

Supplementary Figures

Supplementary Figure 1. Synthetic route of substrates......................................5

Supplementary Figure 2. Synthetic route of NHC-**G** 7

Supplementary Figure 3. Model NHC and model imine used for the calculation of Gibbs energy profile 21

Supplementary Figure 4. Gibbs energy profile for the model cyanation reaction 22

Supplementary Figure 5. DFT optimized transition state structures and their HOMO plots for the rate-determining step of loss of *p*-toluenesulfinate in the full reaction..23

Supplementary Figure 6. Gibbs energy profile for the key steps of the full reaction………………………………………………………………….....24

Supplementary Figure 7. Relaxed PES scan about the dihedral angles for the barriers of isomerisation of atropisomers of (a) substrate **1a**, (b) product **3a**, and (c) condensed imine 26

Supplementary Figure 8. Evidence of bridged hemiacetalvia **1a'** via ^{1}H NMR analysis ………………………………………………………………………. 31

Supplementary Figure 9. Evidence of imine intermediate **4a** via HRMS analysis...31

Supplementary Methods

General information

Commercially available materials purchased from Energy Chemical were used as received. Unless otherwise specified, all reactions were prepared using 4.0 mL vial under N_2 atmosphere in glove-box from UNILAB SP. NMR spectra were recorded on a Brüker ASCEND 400 (400 MHz) spectrometer (^{1}H: 400 MHz, ^{13}C: 101 MHz, ^{19}F: 377 MHz, ^{31}P: 162 MHz). Chemical shifts (δ) for ^{1}H and ^{13}C NMR spectra are given in ppm relative to TMS. The residual solvent signals were used as references for ^{1}H and ^{13}C NMR spectra and the chemical shifts converted to the TMS scale ($CDCl_3$: δH = 7.26 ppm, δC = 77.16 ppm; Acetone-d_6: δH = 2.05 ppm, δC = 206.26, 29.84 ppm; DMSO-d_6: δH = 2.50 ppm, δC = 39.52 ppm). The following abbreviations were used to explain the multiplicities: s = singlet, d = doublet, t = triplet, q = quartet, m = multiplet, b = broad, and etc. All first-order splitting patterns were assigned on the base of the appearance of the multiplet. Splitting patterns that could not be easily interpreted are designated as multiplet (m) or broad (br). High resolution mass spectrometer analysis (HRMS) was performed on Thermo Fisher Q Exactive mass spectrometer. HPLC analyses were measured on Waters systems with Empower 3 system controller, Alliance column heater, and 2998 Diode Array Waters 2489 UV/Vis detector. Chiralcel brand chiral columns from Daicel Chemical Industries were used with models IA, IB, IC, AD-H, AS-H or OD-H in 4.6 x 250 mm size. UPLC analyses were measured on Waters systems with Empower 3 system controller, Waters UPLC H-Class, and Waters ACQUITY UPLC PDA detector. Chiralcel brand chiral columns from Daicel Chemical Industries were used with models IA-U, IB-U, IC-U, or OD-3 in 3.0 x 100 mm size. Optical rotations were measured on a Insmark IP-digi Polarimeter in a 1 dm cuvette. The concentration (*c*) is given in g / 100 mL. Melting point (m.p.): melting points were measured on a Beijing Tech Instrument X-4 digital display micro melting point apparatus and are uncorrected. Analytical thin-layer chromatography (TLC) was carried out on pre-coated silica gel plate (0.2 mm thickness). Visualization was performed using a UV lamp.

Synthesis of substrates[1]

Method A:

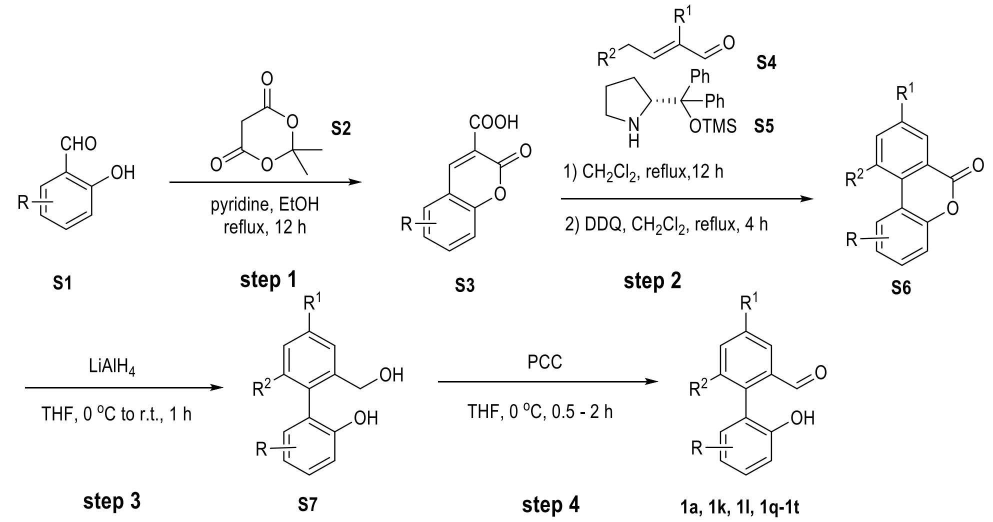

Method B:

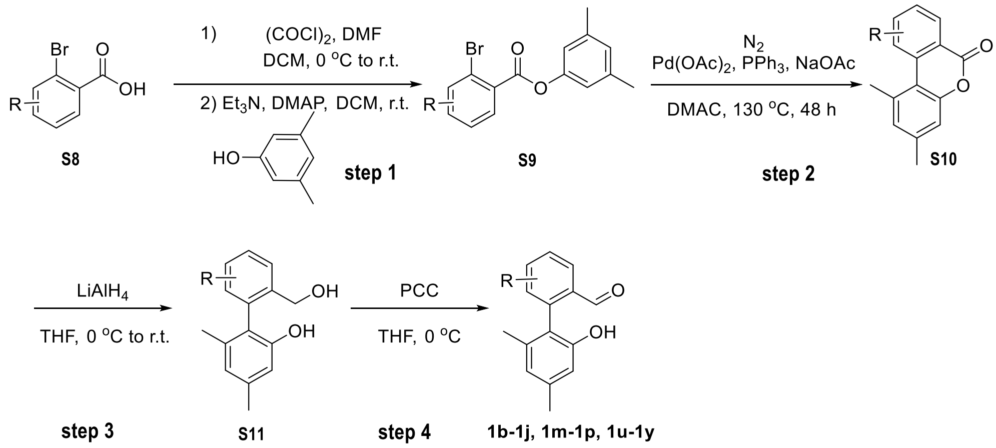

Method C:

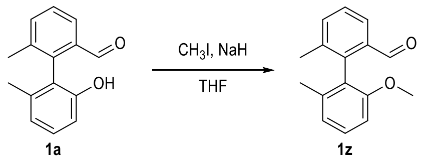

Supplementary Figure 1. Synthetic route of substrates

Method A:

step 1: To a solution of **S1** (5.0 g, 36.7 mmol) in ethanol (50.0 mL), **S2** (36.7 mmol) and a catalytic amount of pyridine (0.1 mL) were in sequence added. The reaction mixture was stirred at 80 ℃ for 4 h. The mixture was allowed to cool to room temperature before being stirred at 0 °C for another hour. The solid which precipitated

out of solution was filtered off, washed thoroughly with ethanol and dried in vacuo to afford the desired acids **S3**.

step 2: To a solution of **S3** (24.5 mmol) in dichloromethane (50 mL), aldehydes **S4** (36.7 mmol) and catalyst **S5** (1.6 g, 4.9 mmol) were added. The reaction mixture was allowed to reflux for 12 h, followed by addition of 2,3-dichloro-5,6-dicyano-1,4-benzoquinone (11.1 g, 49.0 mmol). After 4 h, the reaction mixture was cooled to room temperature and filtered through celite to remove insoluble solids. The filtrate was evaporated under reduced pressure and the residue was subjected to column chromatography on silica gel (50:1 petroleum ether / EtOAc) to afford lactones **S6**.

step 3: To a solution of **S6** (3.0 g, 36.7 mmol) in THF (5 mL / mmol **S6**) at 0 ^{o}C was added $LiAlH_4$ (1.2 e.q.) slowly. The solution was warmed to room temperature and stirred for 1h, then the reaction mixture was quenched carefully with 2 M HCl, extracted with ethyl acetate, and dried over Na_2SO_4. The solvent was removed in vacuo and the residue was chromatographed on silica gel (5:1 petroleum ether / EtOAc)) to give the alcohols **S7**.

step 4: 1.5 equivalents of pyridinium chlorochromate and 1.0 g of silica gel were added in portions at 0 ℃ to a solution of the **alcohols S7** in THF (5.0 mL / mmol **S7**). After stirring at 0 ^{o}C for 0.5 to 2.0 h, the reaction mixture was filtered through celite to remove insoluble solids. The filtrate was evaporated under reduced pressure and the residue was subjected to column chromatography on silica gel (10:1 petroleum ether / EtOAc) to afford the hydroxy aldehydes.

Method B:

step 1: To a suspension of 1.0 equivalent of 2-bromo-3-methylbenzoic acids **S8** in dry dichloromethane (10.0 mL / 0.75 mmol **S8**) and 5 drops of DMF as catalyst, 1.1 equivalents of oxalyl chloride was added at 0 ^{o}C. The mixture was stirred at 0 ^{o}C for 1 h and at room temperature for another 3 h. This solution was added dropwise to a solution of 1.0 equivalent of phenol and 1.5 equivalents of triethylamine dissolved in dichloromethane (10.0 mL / 0.75 mmol **S8**), to which have been added 0.05 equivalents

of *N*, *N*-dimethylpyridin-4-amine as catalyst. Then the mixture is stirred at room temperature for 3 h. After evaporation of the solvent under reduced pressure, the residue is chromatographed to yield the esters **S9**.

step 2: A mixture of the esters **S9** (1.0 equiv.), $Pd(OAc)_2$ (0.1 equiv.), PPh_3 (0.2 equiv.), and NaOAc (2.0 equiv.) were added to *N*,*N*-dimethylacetamide (DMAC ,7.5 mL / mmol), and the reaction mixture was heated to 130 ℃ under N_2 atmosphere. After 24 h, the reaction mixture was cooled to room temperature and filtered through celite to remove insoluble solids. The filtrate was evaporated under reduced pressure and the residue was subjected to column chromatography on silica gel (50:1 petroleum ether / EtOAc) to afford lactones **S10**. The **step 3** and **step 4** were shown in Method A.

Method C:

To a solution of **1a** (1.33 mmol, 300.0 mg) in THF (20.0 mL) at 0 ^{o}C, NaH (1.59 mmol, 60% dispersion in mineral oil, 63.6 mg) was added slowly, then CH_3I (1.46 mmol, 207.0 mg) was added. The mixture was warmed to room temperature and stirred for 1 h, then quenched carefully with 1 M HCl, extracted with ethyl acetate, and dried over Na_2SO_4. The solvent was removed in vacuo and the residue was chromatographed on silica gel (20:1 petroleum ether / EtOAc)) to give **1z** (95% yield, 303.0 mg).

Synthesis of NHC-G

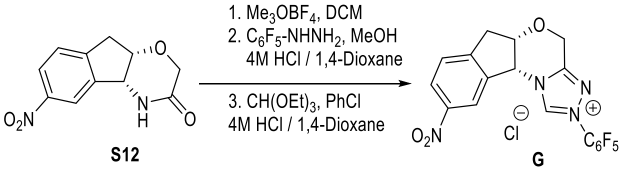

Supplementary Figure 2. Synthetic route of NHC-**G**

To a solution of **S12** (8.5 mmol, 2.0 g) in DCM (50.0 mL) was added Me_3OBF_4 (1.4g, 9.5 mmol), and the mixture was stirred at room temperature for 12 h. Then, the residue was washed with saturated $NaHCO_3$ (3 × 50.0 mL), separation, C_6F_5-$NHNH_2$

(8.5 mmol, 1.69 g) and 4 M HCl / Dioxane (0.2 mL) was added, and the mixture was stirred at 60 °C for 1 h. The mixture was concentrated in vacuo, and the residue was dissolved in PhCl (20.0 mL) followed by the addition of $(EtO)_3CH$ (68.5 mmol, 11.5 mL) and 4M HCl / Dioxane (5.0 mL). The mixture was heated at 130 °C for 4 h. The solvent was removed on a rotary evaporator, the crude product was purified by column chromatography (50:1 DCM / MeOH) to give **G** in an overall 20.3% yield (800.0 mg).

Condition optimization

Supplementary Table 1. Condition optimization for the synthesis of **3a**[a]

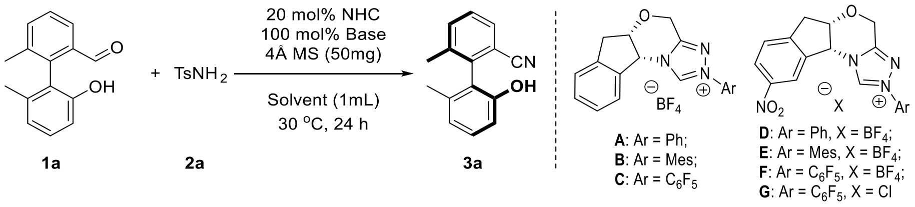

Entry	NHC	Base	Solvent	Yield (%)[b]	er (%)[c]
1	A	Cs_2CO_3	Toluene	95	80:20
2	B	Cs_2CO_3	Toluene	92	60:40
3	C	Cs_2CO_3	Toluene	94	61:39
4	D	Cs_2CO_3	Toluene	24	89:11
5	E	Cs_2CO_3	Toluene	65	60:40
6	F	Cs_2CO_3	Toluene	89	90:10
7	G	Cs_2CO_3	Toluene	97	90:10
8	G	NaOH	Toluene	41	96:4
9	G	CsOAc	Toluene	28	92:8
10	G	K_2CO_3	Toluene	46	96:4
11	G	Na_2CO_3	Toluene	18	96:4
12	G	TEA	Toluene	57	97:3
13	G	DIEA	Toluene	36	97:3
14	G	DABCO	Toluene	49	97:3
15	G	$NH(Et)_2$	Toluene	96	98:2
16	G	DBU	Toluene	82	79:21
17	G	NH(*i*-Pr)$_2$	Toluene	84	97:3
18	G	$NH(Et)_2$	$PhCF_3$	89	95:5
19	G	$NH(Et)_2$	PhCl	88	96:4
20	G	$NH(Et)_2$	Mesitylene	92	97:3
21	G	$NH(Et)_2$	PhOMe	94	96:4
22	G	$NH(Et)_2$	EA	75	94:6
23	G	$NH(Et)_2$	THF	62	91:9

24	G	$NH(Et)_2$	$CHCl_3$	91	97:3
25	G	$NH(Et)_2$	CH_2Cl_2	93	94:6
26	G	$NH(Et)_2$	MeCN	70	75:25
27	G	$NH(Et)_2$	MTBE	90	93:7
28[d]	G	$NH(Et)_2$	Toluene	87	98:2
29[e]	G	$NH(Et)_2$	Toluene	78	98:2

[a] General conditions (unless otherwise specified): **1a** (0.10 mmol), **2a** (0.11 mmol), NHC-**G** (0.02 mmol), base (0.10 mmol), 4Å MS (50 mg) and solvent (1.0 mL) at 30 ^{o}C for 24 h. [b] Yields of isolated products after column chromatography. [c] The er values of **3a** were determined by HPLC using a chiral stationary phase. [d] NHC-**G** (0.01mmol). [e] without 4Å MS.

Supplementary Table 2. Condition optimization for the synthesis of **3g**[a]

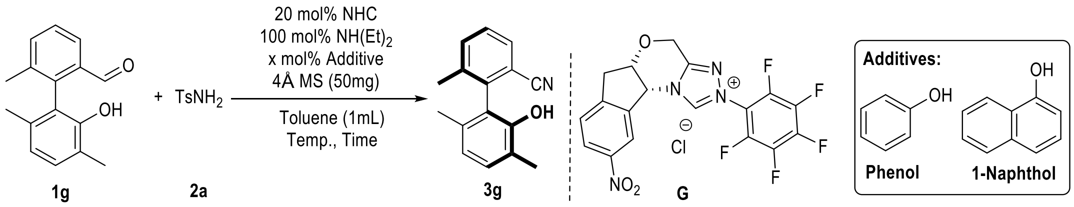

Entry	Additive (mmol%)	Temp.	Time	Yield (%)[b]	er (%)[c]
1	--	30 ^{o}C	12 h	92	89:11
2	Phenol (0.2)	30 ^{o}C	12 h	97	88:12
3	1-Naphthol (0.2)	30 ^{o}C	24 h	96	93:7
4	1-Naphthol (0.4)	30 ^{o}C	24 h	96	93:7
5	1-Naphthol (0.5)	30 ^{o}C	24 h	95	93:7
6	1-Naphthol (1.0)	30 ^{o}C	24 h	98	93:7
7	1-Naphthol (0.2)	0 ^{o}C	24 h	82	95:5
8	1-Naphthol (0.2)	0 ^{o}C	36 h	97	95:5

[a] General conditions (unless otherwise specified): **1g** (0.10 mmol), **2a** (0.11 mmol), NHC (0.02 mmol), base (0.10 mmol), additive (0.20-1.00 mmol) 4Å MS (50 mg) and solvent (1.0 mL) at 30 ^{o}C for 24 h. [b] Yields of isolated products after column chromatography. [c] The er values of **3g** were determined by HPLC using a chiral stationary phase.

General procedure for the catalytic reactions

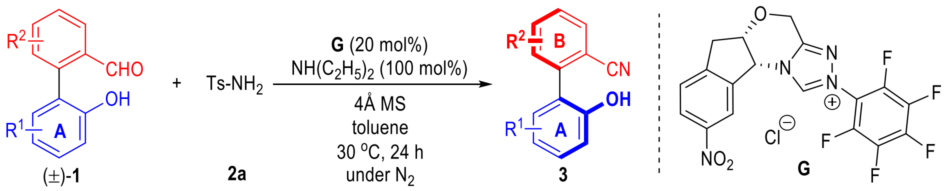

To a 4.0 mL oven-dried vial equipped with a magnetic stir bar was added chiral NHC pre-catalyst **G** (0.02 mmol, 9.2 mg), 4 Å molecular sieves (50 mg), substrates **1** (0.10 mmol) and **2a** (0.11 mmol). Then dried toluene (1.0 mL) and $NH(C_2H_5)_2$ (0.10 mmol, 10.3 μL) was added via syringe in a glove box under N_2 atmosphere. Then the reaction mixture was stirred for 24 hours at 30 °C and then subjected to column chromatography on silica gel (10:1 petroleum ether / EtOAc) directly to give the desired pure products **3** in 62% to 99% isolated yields.

Synthetic transformations of axially chiral products and their applications

General procedure for the enantioselective synthesis of (5)

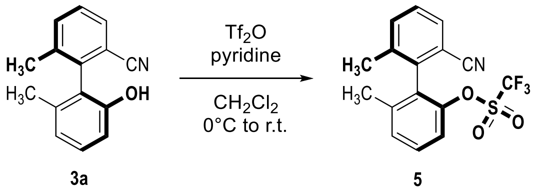

To a Schlenk tube was added **3a** (0.67 mmol, 150.0 mg) and the tube was closed with a septum. The reaction tube was evacuated and backfilled with N_2 for 3 times and then anhydrous dichloromethane (5.0 mL) and pyridine (0.81 mmol, 63.8 mg) were added via syringe. The resulting solution was cooled to 0 ℃ and trifluoromethanesulfonic anhydride (0.81 mmol 227.5 mg) was slowly added. After that the reaction mixture was raised to room temperature and stirred for 2 h. Then the reaction mixture was directly subjected to column chromatography eluting with CH_2Cl_2 to give the pure product **5** in 90% yield (213.6 mg, 97:3 er).

General procedure for the enantioselective synthesis of (6)

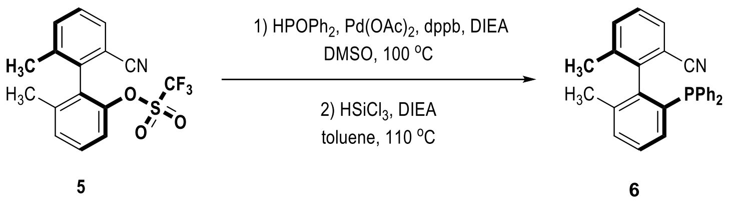

A mixture of the triflate **5** (0.60 mmol, 213.6 mg), $HP(O)Ph_2$ (1.30 mmol, 263.8 mg), $Pd(OAc)_2$ (0.05 mmol, 6.8 mg), dppb (0.05 mmol, 12.8 mg) and DIEA (3.08 mmol, 510.0 μL) in DMSO (2.0 mL) was heated to 100 ℃ and stirred for 3 h under N_2. Then the reaction mixture was cooled to room temperature and concentrated in vacuum. Ethyl acetate (30.0 mL) was added and the solution was washed with H_2O (3 × 10.0 mL), dried over Na_2SO_4 and the solvent was removed in vacuum. The residue containing phosphine oxide was used directly subjected to a flame-dried Schlenk tube and was evacuated and backfilled with N_2 for 3 times. Anhydrous toluene (2.0 mL) and DIEA (15.73 mmol, 2.6 mL) were then added via syringe and the mixture was treated with $HSiCl_3$ (5.24 mmol, 530.0 μL,) at room temperature. Then the reaction was heated to reflux with stirring for 2 h. After cooling to room temperature, saturated $NaHCO_3$ aqueous solution was added to quench the reaction and the resulting precipitate was removed by filtration over celite and washed with diethyl ether (3 × 10.0 mL). The combined organic layer was separated and dried over $MgSO_4$. After concentration under vacuum, the residue was purified via SiO_2 flash chromatography (20:1 petroleum ether / EtOAc) to afford the pure product **6** in 73% yield (170.7 mg, 97:3 er). The product **6** was recrystallized from CH_2Cl_2 / petroleum ether to further improve its optical purity (> 99:1 er value) before subjecting to the following steps.

General procedure for the enantioselective synthesis of 7 (11, 18)

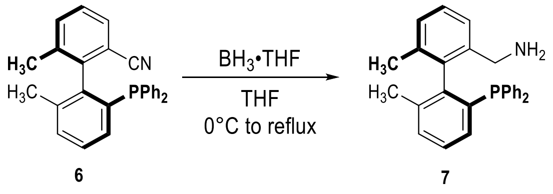

To a cooled solution of **6** (0.26 mmol, 100.0 mg) in dry THF (15.0 mL) at 0 ℃ was added borane-tetrahydrofuran (0.91 mmol, 1 M in THF, 3.6 mL) dropwisely. The

solution was then raised to room temperature and stirred for 1h. After that the reaction was gradually heated to 70 ℃ over 30 min and stirred under reflux for 30 min. Then the mixture was cooled to 0 ℃ and MeOH (5.0 mL) was slowly added to quench the reaction. The resulting solution was stirred at room temperature for 20 min and concentrated under reduced pressure. The residue was dissolved in THF (20.0 mL) and 1 M HCl (5.0 mL) was added. The mixture was stirred for 5 min at room temperature and then heated to reflux with stirring for 2 min. The solution was then cooled to room temperature and poured into saturated aqueous $NaHCO_3$ (20.0 mL). After removing the organic solvent in vacuo, the aqueous mixture was extracted with CH_2Cl_2 (3 × 20.0 mL) and dried over Na_2SO_4. After concentration, the residue was purified by column chromatography eluting with Et_3N / MeOH / CH_2Cl_2 (1:10:100) to give the product **7** in 80% yield (81.0 mg).

General procedure for the enantioselective synthesis of (8)

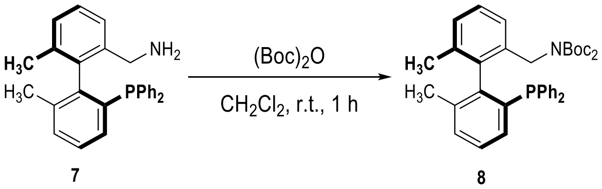

A mixture of **7** (0.05 mmol, 20.0 mg), $(Boc)_2O$ (0.10 mmol, 22.1 mg), *N*, *N*-dimethylpyridin-4-amine (0.001 mmol, 1.3 mg) was stirred in CH_2Cl_2 (1.0 mL) at room temperature for 1 h. Then the mixture was concentrated in vacuo and the resulting residue was purified via column chromatography on silica gel (20:1 petroleum ether / EtOAc) to give **8** in 94% yield (28.4 mg, > 99:1 er).

General procedure for the enantioselective synthesis of (9)

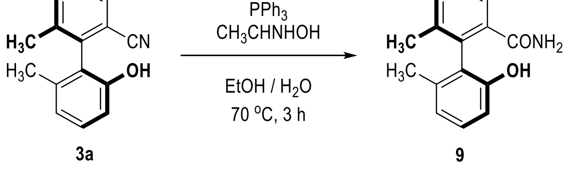

A mixture of **3a** (0.20 mmol, 44.7 mg), acetaldehyde oxime (1.60 mmol, 94.4 mg), $Pd(OAc)_2$ (0.02 mmol, 0.9 mg), and PPh_3 (0.02 mmol, 1.0 mg) was dissolved in EtOH /

H_2O (0.8 mL / 0.2 mL) and stirred at 70 °C for 3 h. Then the mixture was cooled to room temperature and concentrated in vacuo. The resulting residue was purified via column chromatography on silica gel (3:1 petroleum ether / EtOAc) to give **9** in 93% yield (47.8 mg, 96:4 er).

General procedure for the enantioselective synthesis of (10)

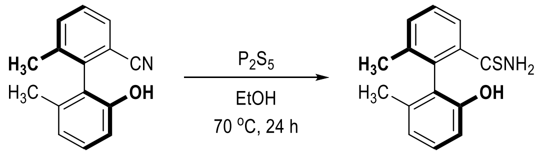

A solution of P_2S_5 (0.60 mmol, 133.4 mg) in ethanol (2.0 mL) was stirred at room temperature for 1 h. Then **3a** (0.20 mmol, 44.7 mg) was added and the reaction mixture was stirred at 70 °C for 24 h. After removing the solvent, the residue was purified via column chromatography (5:1 petroleum ether / EtOAc) to give **10** in 83% yield (42.8 mg, 98:2 er).

General procedure for the enantioselective synthesis of (12)

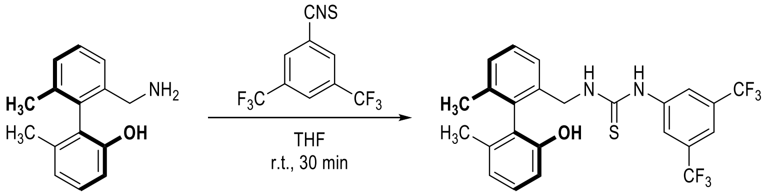

To a solution of **9** (0.18 mmol, 40.0 mg) in THF (2.0 mL) was added 3,5-bis(trifluoromethyl)phenyl isothiocyanate (0.18 mmol, 47.7 mg). The reaction was stirred for 30 min at room temperature and then concentrated in vacuo. The residue was subjected to column chromatography on silica gel (6:1 petroleum ether / EtOAc) to give the product **11** in 90% yield (79.0 mg, 98:2 er).

General procedure for the enantioselective synthesis of (13)

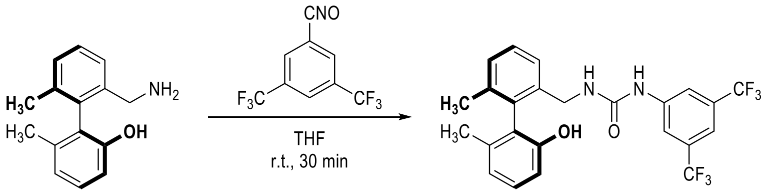

To a solution of **11** (0.18 mmol, 40.0 mg) in THF (2.0 mL) was added 3,5-bis(trifluoromethyl)phenyl isocyanate (0.18 mmol, 45.0 mg). The reaction was stirred for 30 min at room temperature and then concentrated in vacuo. The residue was subjected to column chromatography on silica gel (6:1 petroleum ether / EtOAc) to give the product **13** in 66% yield (56.0 mg, 98:2 er).

General procedure for the enantioselective synthesis of (14)

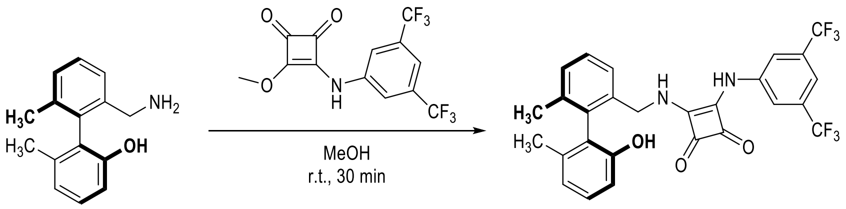

To a solution of **11** (0.10 mmol, 22.7 mg) in MeOH (1.0 mL) was added 3-((3,5-bis(trifluoromethyl)phenyl)amino)-4-methoxycyclobut-3-ene-1,2-dione (0.10 mmol, 33.9 mg). The reaction was stirred for 30 min at room temperature and then concentrated in vacuo. The residue was subjected to column chromatography on silica gel (3:1 petroleum ether / EtOAc) to give the product **14** in 95% yield (51.0 mg, 96:4 er).

General procedure for the enantioselective synthesis of (15)

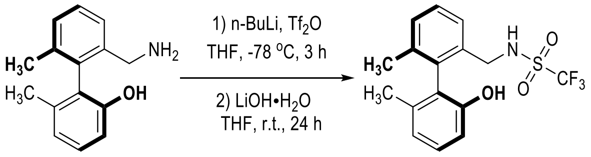

To a solution of amine **11** (0.43 mmol, 130.0 mg) in THF (8.0 mL) at −78 °C was added *n*-BuLi (0.87 mmol, 1.6 M in hexane, 540.0 μL). After stirring for 30 min at −78 °C,

trifluoromethanesulfonic anhydride (0.87 mmol) was slowly added into the mixture and the reaction was stirred at −78 °C for an additional 2 h. Then the reaction mixture was warmed to room temperature and quenched with 1 M HCl (10.0 mL). The resulting mixture was extracted with ethyl acetate (30.0 mL × 2), washed with brine (30.0 mL), dried over Na_2SO_4, and concentrated. The residue was dissolved in THF (10.0 mL) and a solution of LiOH H_2O (5.21 mmol, 219.0 mg) in H_2O (3.0 mL) was added. The mixture was stirred at room temperature for 24 h and then quenched with 1 M HCl (10.0 mL). The mixture was extracted with ethyl acetate (30.0 mL × 3), washed with brine (30.0 mL × 2), dried over Na_2SO_4, and concentrated in vacuo. The residue was purified via column chromatography on silica gel (6:1 petroleum ether / EtOAc) to give the product **15** in an overall 33.0% yield (67.8 mg, 97:3 er).

General procedure for the enantioselective synthesis of 16 (19)

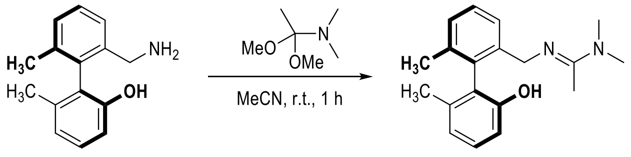

11 16

To a solution of **11** (0.10 mmol, 22.7 mg) in MeCN (1.0 mL) was added 1,1-dimethoxy-*N*, *N*-dimethylethan-1-amine (0.11 mmol, 33.9 mg). After being stirred at room temperature for 1 h, the mixture was concentrated in vacuo. The crude residue was purified by column chromatography eluting with Et_3N / MeOH / CH_2Cl_2 (1:10:100) to give the product **16** in 64% yield (19.0 mg).

General procedure for the enantioselective synthesis of 17 (20)

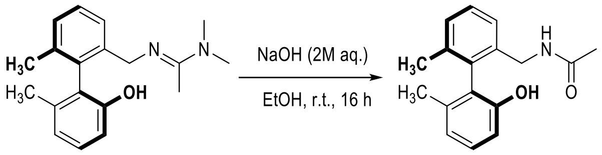

16 17

To a stirred solution of **16** (0.10 mmol, 29.6 mg) in EtOH (0.5 mL) was added 2 M aqueous solution of NaOH (0.5 mL). After 16 h the volatiles were removed and the aqueous mixture was extracted with CH_2Cl_2 (3 × 10.0 mL), dried over Na_2SO_4, and

concentrated. The residue was purified by column chromatography on silica gel (5:1 to 3:1 petroleum ether / EtOAc) to give the product **17** in 92% yield (25.9 mg, 98:2 er).

General procedure for the enantioselective synthesis of (23)

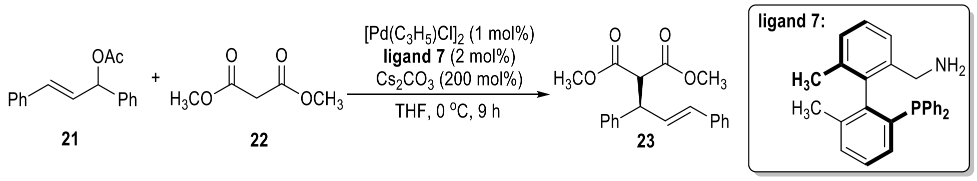

To a 4.0 mL oven-dried vial equipped with a magnetic stir bar was added chiral ligand **7** (0.002 mmol, 0.8 mg), $[Pd(C_3H_5)Cl]_2$ (0.001 mmol, 0.4 mg) and Cs_2CO_3 (65.2 mg, 0.2 mmol) in a glove box under N_2 atmosphere. The solution of the substrates **21** (0.15 mmol, 37.9 mg) and **22** (0.10 mmol, 13.2 mg) in dried THF (2.0 mL) was added via syringe. Then the reaction mixture was stirred at 0 ℃ for 9 h and then subjected to column chromatography on silica gel (20:1 petroleum ether / EtOAc) to give the product **23** in 85% yield (27.6 mg, 83:17 er).

General procedure for the enantioselective synthesis of (25)

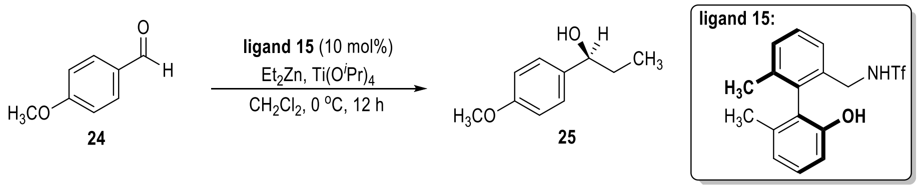

To a solution of ligand **15** (0.015 mmol, 5.4 mg) in dichloromethane (2.0 mL) was added $Ti(O^iPr)_4$ (0.60 mmol, 178.0 μL) at room temperature. After stirring for 15 min, aldehyde **24** (0.50 mmol, 60.7 μL) was added and the reaction mixture was cooled to 0 ^oC. Diethylzinc (0.90 mmol, 1.0 M in hexane, 0.9 mL) was slowly added into the solution and the reaction mixture was stirred for 12 h at 0 ^oC. Then the reaction was quenched with 1 M HCl solution (10.0 mL) and the resulting mixture was extracted with ethyl acetate (30.0 mL × 2), washed with brine (30.0 mL × 2), dried over $MgSO_4$, and concentrated in vacuo. The residue was purified via column chromatography on silica gel (5:1 petroleum ether / EtOAc) to give the product **25** in 95% yield (78.9 mg, 76:24 er).

General procedure for the enantioselective synthesis of (28)

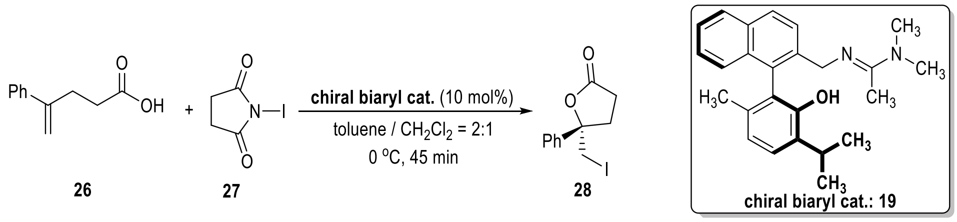

To a solution of **19** (0.10 mmol, 22.7 mg) in toluene / CH_2Cl_2 (1.0 mL / 0.5 mL) was added the carboxylic acid **26** (17.6 mg, 0.10 mmol) at 0 ℃. After stirring for 10 min, NIS **27** (27.0 mg, 0.12 mmol) was added and the reaction was stirred for an additional 45 min at 0 ℃. After concentration, the resulting residue was purified via column chromatography on silica gel (5:1 petroleum ether / EtOAc) to give the product **28** in 92% yield (27.8 mg, 74:26 er).

X-ray crystallography of compounds 3a, 11 and NHC-G

Good quality crystal of **3a** (white block crystal) was obtained by vaporization of a CH_2Cl_2 / petroleum ether solution of compound **3a**. A colorless block crystal of compound **11** was obtained by vaporization of a CH_2Cl_2 / petroleum ether solution. A colorless block crystal of NHC-**G** was obtained by vaporization of dichloromethane / MeOH solution.

CCDC: **2056374**, **2116213** and **2093769** contain the supplementary X-ray crystallographic data of **3a**, **11** and NHC-**G** respectively. These data can be obtained free of charge from The Cambridge Crystallographic Data Centre via www.ccdc.cam.ac.uk/data_request/cif.

Supplementary Table 3. X-ray crystallography of compounds 3a, 11 and NHC-G

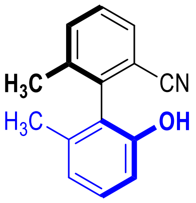<br>**3a**	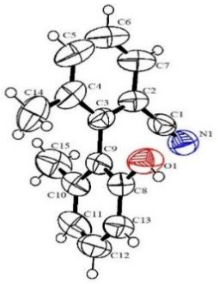<br>CCDC **2056374**
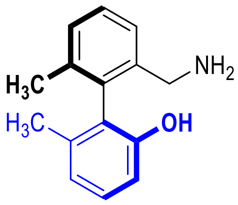<br>**11**	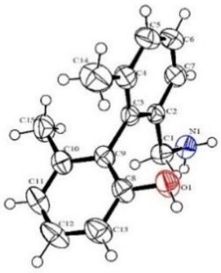<br>CCDC **2116213**
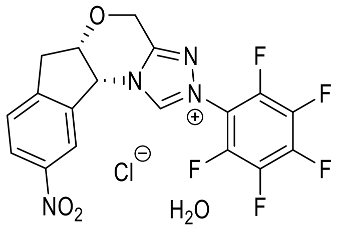<br>NHC-**G**	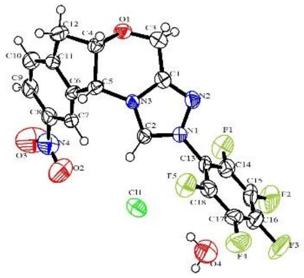<br>CCDC **2093769**

Density functional theory (DFT) calculations

Computational methods

Density functional theory (DFT) calculations were performed with *Gaussian 16* rev. B.01[2]. Geometry optimizations were performed using the M06-2X[3] functional with the Karlsruhe-family basis set of double-ζ valence def2-SVP[4,5] for all atoms. Minima and transition structures on the potential energy surface (PES) were confirmed using harmonic frequency analysis at the same level of theory, showing respectively zero and one imaginary frequency. Gibbs energies were evaluated at the reaction temperature of 30 ℃, using a quasi-RRHO treatment of vibrational entropies[6], using the Good Vibes code[7]. Vibrational entropies of frequencies below 100 cm^{-1} were obtained according to a free rotor description, using a smooth damping function to interpolate between the two limiting descriptions. The free energies were further corrected using standard concentration of 1 mol/L, which were used in solvation calculations.

Single point (SP) corrections were performed using the domain-based local pair natural orbital – coupled cluster with perturbative triple excitations (DLPNO-CCSD(T)) calculations[8,9] using ORCA version 5.0.1[10-12]. T_0 approximation which neglects the couplings between different triples by the off-diagonal Fock matrix elements, instead of the recently published iterative T_1 algorithm[13], was employed. The NormalPNO settings with $T_{cutPairs} = 10^{-4}$, $T_{CutDO} = 10^{-2}$, $T_{CutPNO} = 3.33\times10^{-7}$ and $T_{CutMKN} = 10^{-3}$ was used throughout. The TightSCF convergence with KDIIS algorithm[14] for SCF iterations were used. The complete basis set (CBS) extrapolation scheme of Helgaker et al[15-17], was performed using either the correlation-consistent double-/triple-ζ cc-pV(DT)Z basis set[18-20] or the aug-cc-pV(DT)Z[21-22] basis sets, which are augmented with diffuse functions. The auxiliary basis sets required for the integral evaluations in the DLPNO-CCSD(T) correlation energy calculations were generated automatically using the “AutoAux” command from the automated auxiliary basis set construction module[23] of ORCA. DEFGRID2 grid for integration was employed throughout.

For the basis sets augmented with diffuse functions, the aug-cc-pV(DT)Z basis set produces linear dependency errors due to the addition of diffuse functions using the

"AutoAux" command, in this case, DLPNO-CCSD(T) was run separately with aug-cc-pVDZ or aug-cc-pVTZ basis set with corresponding auxiliary basis sets aug-cc-pVD(T)Z/C[21,24] and the obtained values are extrapolated manually according to the following formulae:

$$E_{\mathrm{SCF}}^{(X)} = E_{\mathrm{SCF}}^{(\infty)} + A\exp(-\alpha\sqrt{X}) \qquad \text{Eq (1)}$$

$$E_{corr}^{(\infty)} = \frac{X^{\beta}E_{\mathrm{corr}}^{(X)} - Y^{\beta}E_{\mathrm{corr}}^{(Y)}}{X^{\beta} - Y^{\beta}} \qquad \text{Eq (2)}$$

for the extrapolation of HF energy (Eq (1)) and of correlation energy (Eq (2)) to the basis set limit, respectively. $E_{\mathrm{SCF}/corr}^{(X)}$ is the SCF/correlation energy calculated with basis set of cardinal number *X*, and $E_{\mathrm{SCF}/corr}^{(\infty)}$ is the basis set limit SCF/correlation energy and *A, α,* and *β* are constants. For correlation energy, *X* and *Y* are the cardinal numbers of the basis sets used for extrapolation (*X*=2, *Y*=3 herein). For Extrapolate(2/3, cc), *α*=4.42, and *β*=2.46 and for Extrapolate(2/3, aug-cc), *α*=4.3, and *β*=2.51.

The integral equation formalism variant of the polarizable continuum model (IEF-PCM) with the SMD implicit continuum solvation model[25] was included to account for the solvent effect of toluene. Unless otherwise stated, the final SMD (toluene)-DLPNO-CCSD(T)/ cc-pV(DT)Z//M06-2X/def2-SVP Gibbs energies are used for discussion throughout. *All Gibbs energy values in the text and figures are quoted in kcal mol^{-1}*. All molecular structures and molecular orbitals were visualized using *PyMOL* software[26].

Geometries of all optimized structures (in *.xyz* format with their associated energy in Hartrees) are included in a separate folder named *optimised_xyz_structures* with an associated README file. All these data have been deposited and uploaded to zenodo.org (DOI: 10.5281/zenodo.5573970) under open access.

Model system calculation

To initially explore the potential energy surface of this reaction and to increase computational efficiency, we carried out a model calculation in which a model NHC and a model imine is used (Supplementary Figure 3). Note that for the model NHC used,

the reaction centre is similar as the chiral NHC catalyst used in the reaction. For the imine simplification, we note that the methanesulfinate group has similar reactivity as *p*-toluenesulfinate group. We use this model reaction to determine the key steps for the overall transformation, from which we applied the full model to the key step to determine the stereoselectivity.

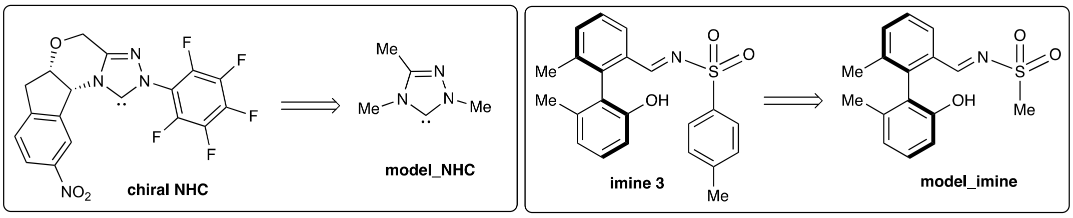

Supplementary Figure 3. Model NHC and model imine used for the calculation of Gibbs energy profile.

The full Gibbs energy profile for this model reaction is shown in Supplementary Figure 4. The Gibbs energies were calculated at SMD(Toluene)-DLPNO-CCSD(T)/CBS//M06-2X/def2-SVP, using complete basis set (CBS) extrapolation at (2/3,cc) or (2/3,aug-cc) (in square brackets) procedure as outlined in the computational methods section. The reaction proceeds with firstly the addition of NHC catalyst to the imine C=N bond, giving a highly exergonic adduct **model_INT2**, at -9.3 [-8.5] kcal mol^{-1}. This is followed by the loss of methanesulfinate anion, via transition state, **model_TS2**, at 23.9 [23.7] kcal mol^{-1}. The final deprotonation of imine intermediate via **model_TS3**, regenerates the NHC catalyst and yields the nitrile product. We note that the use of basis set augmented with diffuse functions (aug-cc-pVD(T)Z) gives similar energies (within 1 kcal mol^{-1}) as the basis set not augmented with diffuse functions (cc-pVD(T)Z), thus, for full system calculations, we use Extrapolate(2/3,cc) without diffuse functions for increased computational efficiency.

We herein focus on the steps of NHC addition and the loss of methanesulfinate since these steps are likely stereo-determining in the overall transformation of the full system as the regeneration of NHC catalyst via **model_TS3** through deprotonation is likely facile and simply carries the stereochemical information from previous steps forward. From the Gibbs energy profile in Supplementary Figure 4, we can see that the

NHC adduct, **model_INT2**, is the resting state of the catalytic cycle. The rate-limiting step is the loss of methanesulfinate, **model_TS2**, with an energetic span of 33.2 [32.2] kcal mol^{-1} (from **model_INT2** to **model_TS2**). Moreover, the addition of NHC, **model_TS1**, is reversible, as the subsequent loss of methanesulfinate has a barrier of 33.2 [32.2] kcal mol^{-1}, which is higher than the barrier for the reversible process of adduct dissociation (going from **model_INT2** to **model_INT1**) with a barrier of 24.4 [24.4] kcal mol^{-1}. We note that this rate-limiting barrier is very high and is not consistent with the good reactivity at ambient temperature used for the reaction. We further carried out investigation of the full system to determine the energetic span for the actual system used in the reaction (*vide infra*).

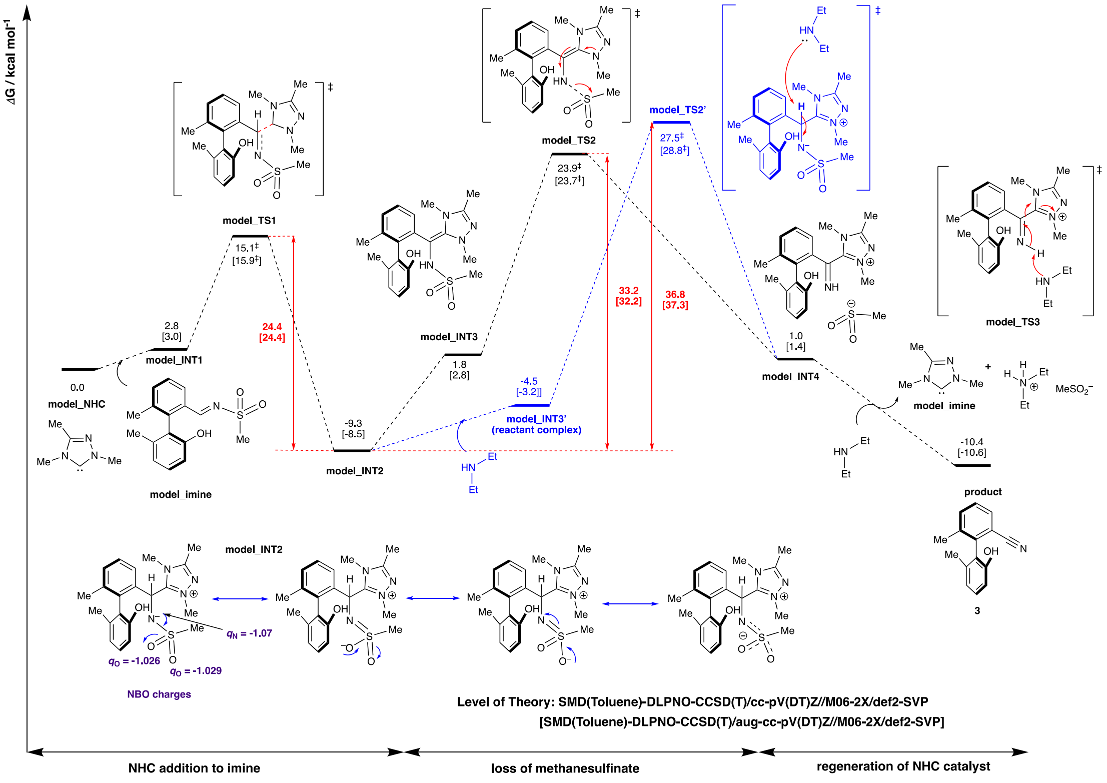

Supplementary Figure 5. Gibbs energy profile for the model reaction calculated at SMD(Toluene)-DLPNO-CCSD(T)/CBS//M06-2X/def2-SVP, using complete basis set (CBS) extrapolation at (2/3,cc) or (2/3,aug-cc) (in square brackets) procedure.

We also checked the alternative mechanism, in which the base-assisted deprotonation of the NHC-imine adduct via **model_TS2'** occurs to give the imine intermediate **model_INT4** directly, as proposed in a previous study of NHC-catalysed desulfonylation of tosylated aldimines[27]. However, this TS (**model_TS2'** at 27.5 [28.8]

kcal mol^{-1}) has an energetic span that is 3.6 [5.1] kcal mol^{-1} higher than the loss of methanesulfinate from the aza-Breslow intermediate (**model_TS2**). With these results, we focus on the step of loss of anion in the full system as both the rate-limiting and stereo-determining step.

Key steps and key transition state structures for the full reaction

<table>
<tr><th>major_TS2</th><th>minor_TS2</th></tr>
<tr><td>$\Delta G^{\ddagger}$ = 23.7 kcal mol^{-1}</td><td>$\Delta G^{\ddagger}$ = 27.2 kcal mol^{-1}</td></tr>
<tr><td>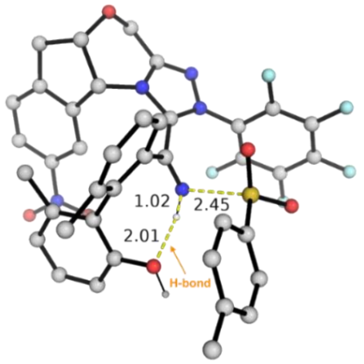
</td><td>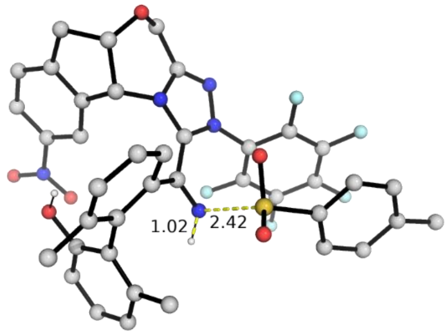
</td></tr>
<tr><td>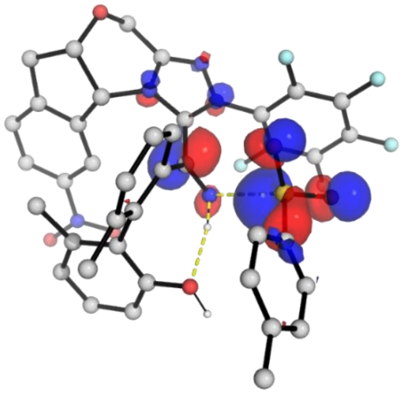</td><td>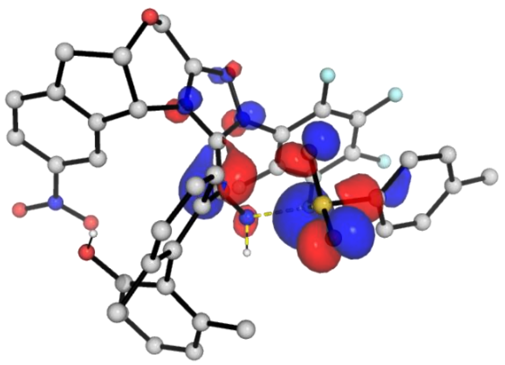</td></tr>
</table>

Supplementary Figure 6. DFT optimized transition state structures and their HOMO plots for the rate-determining step of loss of *p*-toluenesulfinate in the full reaction.

For the full reaction, we focus on the step of loss of toluenesulfinate from the Breslow intermediate as reflected by **model_TS2** in Supplementary Figure 5. Conformational sampling was carried out at the GFN2-xTB[28] level of theory using the *crest* program[29-31] from Grimme and co-workers. Note that since no TS structure could be located on the GFN2-xTB potential energy surface, we performed conformational

sampling on the aza-Breslow intermediate. A total of 104 conformers were located by the crest program, and these are sorted into 19 clusters of distinct conformers using the clustering_traj.py[32] with an RMSD cutoff of 1.0 Å (excluding H atoms). The 4 lowest energy structures were reoptimised at M06-2X/def2-SVP level of theory to yield the relevant TS structures for the rate- determining step of loss of toluenesulfinate. The lowest Gibbs energy structures for the TSs leading to both major and minor products, **major_TS2** and **minor_TS2**, respectively, are shown in Figure 6.

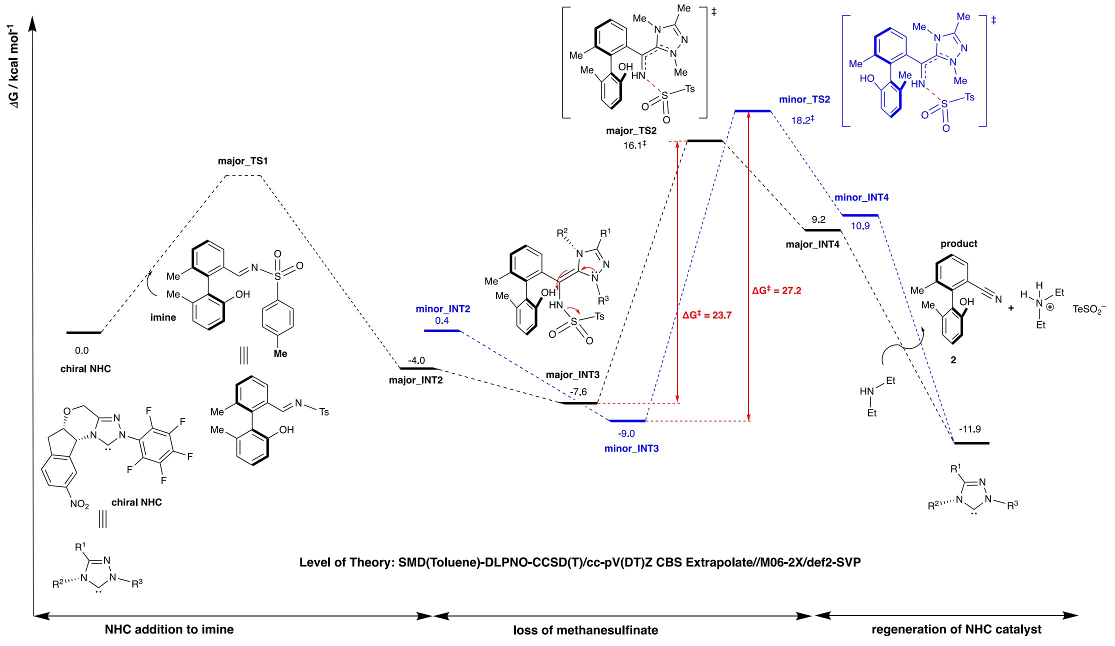

Supplementary Figure 6. Gibbs energy profile for the key steps of the full reaction calculated at SMD(Toluene)-DLPNO-CCSD(T)/cc-pV(DT)Z CBS Extrapolation//M06-2X/def2-SVP.

The Gibbs energy profile for the key step of the full system is shown in Supplementary Figure 6. The energetic span for the rate-determining TS leading to the major product is 23.7 kcal mol^{-1} and to the minor product is 27.2 kcal mol^{-1}. This barrier difference of 3.5 kcal mol^{-1} translates to an enantiomeric excess of 99%, at experimental temperature of 30°C, which is in good agreement with experimental observations. In addition, the energetic span of 23.7 kcal mol^{-1} is consistent with excellent reactivity at experimental temperature of 30°C. Their HOMO structures are

similar, and **major_TS2** is likely more favoured due to the hydrogen bonding formed between the OH group on the substrate and the amine group of the Breslow intermediate.

Rotational barriers for atropisomers

Supplementary Figure 7 shows the relaxed PES scan about the dihedral angles for the barriers of isomerisation of atropisomers of (a) **1a**, (b) **3a**, and (c) condensed imine. The barriers for isomerisation are all well over 50 kcal mol^{-1}, indicating that these atropisomers will not racemise easily at the reaction temperature of 30 ^{o}C.

(a)

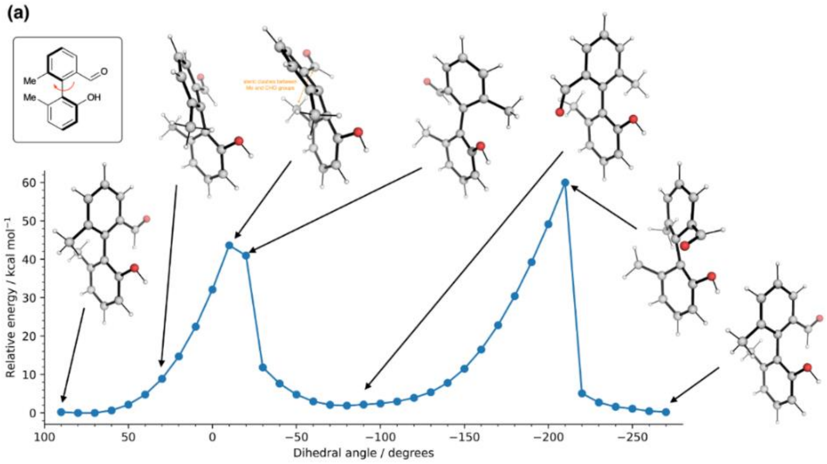

(b)

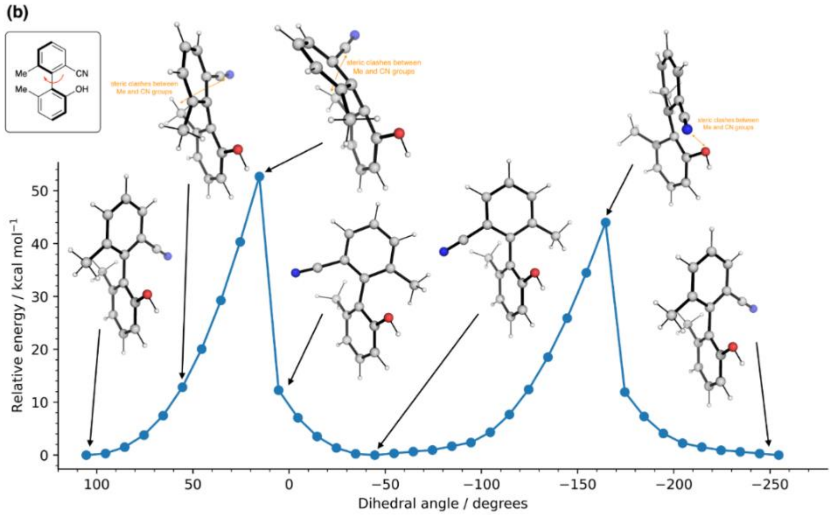

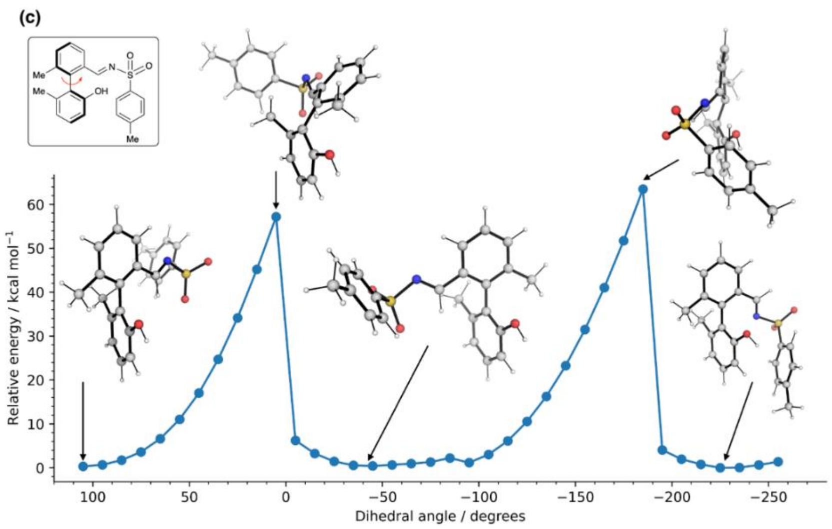

Supplementary Figure 7. Relaxed PES scan about the dihedral angles for the barriers of isomerisation of atropisomers of (a) substrate **1a**, (b) product **3a**, and (c) condensed imine

Optimised structures and absolute energies, zero-point energies

Geometries of all optimized structures (in *.xyz* format with their associated energy in Hartrees) have been deposited and uploaded to zenodo.org (DOI: 10.5281/zenodo. 5573970) under open access.

Absolute values (in Hartrees) for SCF energy, zero-point vibrational energy (ZPE), enthalpy and quasi-harmonic Gibbs free energy (at 30 ^{o}C/303.15 K) for optimised structures are given below. Single point corrections in SMD toluene using DLPNO-CCSD(T)/cc-pV(DT)Z CBS Extrapolate level of theory are also included (Supplementary Table 4). The individual energy values for (aug-)cc-pV(DT)Z basis sets and for extrapolated energies are included in Supplementary Table 5 and Supplementary Table 6.

Supplementary Table 4. Optimised structures and absolute energies, zero-point energies

Structure	E/au	ZPE/au	H/au	T.S/au	qh-G/au	SP SMD (toluene) DLPNO-CCSD(T) /cc-pV(DT)Z CBS Extrapolate
**model_system**						
**Et_2NH_2Ms**	-802.26423	0.209038	-802.04122	0.050974	-802.09067	-802.106771761
**model_imine**	-1297.3011	0.303744	-1296.9757	0.069216	-1297.0413	-1297.057584649
**model_NHC**	-359.72744	0.144296	-359.57359	0.039996	-359.61336	-359.734096841
**model_INT1**	-1657.0724	0.449095	-1656.592	0.089979	-1656.6768	-1656.808959381
**model_TS1**	-1657.0516	0.44998	-1656.5711	0.086668	-1656.6537	-1656.791717706
**model_INT2**	-1657.0875	0.452629	-1656.6046	0.086001	-1656.6865	-1656.833728768
**model_INT3**	-1657.0826	0.451871	-1656.6004	0.085618	-1656.682	-1656.815595236
**model_TS2**	-1657.0514	0.450388	-1656.5709	0.084953	-1656.6522	-1656.778899839
**model_INT4**	-1657.078	0.449421	-1656.5976	0.087806	-1656.681	-1656.813275761
**model_INT3'**	-1870.6262	0.604494	-1869.9829	0.106287	-1870.0821	-1870.382825950
**model_TS2'**	-1870.5768	0.600681	-1869.9385	0.101509	-1870.0344	-1870.330222484
**Full system**						
**NHC**	-1633.1919	0.262908	-1632.9043	0.07842	-1632.9774	-1633.365826
**substrate_1**	-729.63651	0.252446	-729.36729	0.056997	-729.4226	-729.5741718
**substrate_1-c2**	-729.63341	0.252251	-729.36439	0.0568	-729.41954	-729.5719554
**$TsNH_2$**	-874.78436	0.155925	-874.61621	0.048451	-874.66261	-874.5913381
**Et_2N**	-213.51354	0.150026	-213.35549	0.035622	-213.39093	-213.5363377
**product**	-708.58387	0.241686	-708.32566	0.056235	-708.38037	-708.4984566
**major_TS2**	-3161.3151	0.649318	-3160.6149	0.132663	-3160.737	-3161.1548
**major_TS2-c2**	-3161.3219	0.649753	-3160.6214	0.133526	-3160.7436	-3161.153644
**major_TS2-c3**	-3161.3099	0.649559	-3160.6099	0.132688	-3160.7315	-3161.15015
**major_TS2-c4**	-3161.3099	0.650099	-3160.609	0.134329	-3160.7313	-3161.144974
**minor_TS2**	-3161.3158	0.649746	-3160.6153	0.133319	-3160.7375	-3161.151609
**major_INT2**	-3161.3385	0.651677	-3160.6368	0.130192	-3160.756959	-3161.190316
**major_INT3**	-3161.3527	0.651003	-3160.6507	0.133176	-3160.772938	-3161.194197

**major_INT4**	-3161.3198	0.650182	-3160.618	0.135629	-3160.741952	-3161.165459
**minor_INT2**	-3161.3296	0.652316	-3160.6271	0.132009	-3160.748028	-3161.183229
**minor_INT3**	-3161.3596	0.651797	-3160.6571	0.132231	-3160.77859	-3161.197593
**minor_INT4**	-3161.3377	0.651058	-3160.6356	0.133459	-3160.758143	-3161.169332

Supplementary Table 5. Raw energy values obtained at SMD(toluene)-DLPNO-CCSD(T)/cc-pV(DT)Z basis sets and the complete basis set (CBS) extrapolation. Final single-point (SP) energy = Extrapolated SCF energy + Extrapolated correlation energy. α = 4.42 and β = 2.46 in the extrapolation of SCF and correlation energies. All values have the units of a.u.

**Structure**	**SCF with SMD correction**			**Correlation**			**Final SP Energy**
	**cc-pVDZ**	**cc-pVTZ**	**Extrapolated**	**cc-pVDZ**	**cc-pVTZ**	**Extrapolated**	
**model_system**							
**Et_2NH_2Ms**	-799.739799018	-799.905382516	-799.959232958	-1.567095981	-1.933459369	-2.147538803	-802.106771761
**model_imine**	-1292.350713901	-1292.651932034	-1292.749893061	-3.196967918	-3.898033504	-4.307691588	-1297.057584649
**model_NHC**	-357.922994234	-358.016208822	-358.046523720	-1.268622429	-1.533055357	-1.687573121	-359.734096841
**model_INT1**	-1650.270396404	-1650.656738374	-1650.782383055	-4.490820235	-5.460157409	-6.026576326	-1656.808959381
**model_TS1**	-1650.239943402	-1650.627614341	-1650.753691224	-4.499830286	-5.470707603	-6.038026482	-1656.791717706
**model_INT2**	-1650.267354080	-1650.656000373	-1650.782394457	-4.505104472	-5.481052457	-6.051334311	-1656.833728768
**model_INT3**	-1650.244898291	-1650.633466029	-1650.759834565	-4.511204910	-5.486096252	-6.055760671	-1656.815595236
**model_TS2**	-1650.205846635	-1650.580614833	-1650.702495535	-4.530162627	-5.506118084	-6.076404304	-1656.778899839
**model_INT4**	-1650.268556189	-1650.636595358	-1650.756287670	-4.511122175	-5.486840459	-6.056988091	-1656.813275761
**model_INT3'**	-1862.599799293	-1863.042477792	-1863.186444026	-5.373794166	-6.524173499	-7.196381924	-1870.382825950
**model_TS2'**	-1862.5208	-1862.9616	-1863.1049	-5.3995017	-6.5518600	-7.2252248	-1870.33022

	92530	54638	97631	67	53	54	2484
**Full System**							
**NHC**	-1626.335619693	-1626.792485702	-1626.941065945	-4.653597280	-5.771518237	-6.424760054	-1633.365826
**substrate_1**	-725.980592246	-726.162570798	-726.221753178	-2.520900897	-3.045737493	-3.352418578	-729.5741718
**substrate_1-c2**	-725.975835382	-726.158165793	-726.217462602	-2.522014761	-3.047457520	-3.354492809	-729.5719554
**$TsNH_2$**	-871.981523630	-872.180990155	-872.245859907	-1.715663631	-2.113189408	-2.345478158	-874.5913381
**Et_2N**	-212.337812151	-212.398958746	-212.418844611	-0.845185687	-1.017060424	-1.117493075	-213.5363377
**product**	-704.983965067	-705.157047882	-705.213337223	-2.481844577	-2.988854842	-3.285119355	-708.4984566
**major_TS2**	-3148.174727490	-3148.960491447	-3149.216034643	-8.774931548	-10.771877556	-11.938765637	-3161.1548
**major_TS2-c2**	-3148.189755503	-3148.972161576	-3149.226612733	-8.765892068	-10.761137393	-11.927031704	-3161.153644
**major_TS2-c3**	-3148.180046923	-3148.965769449	-3149.221299171	-8.767520697	-10.762886435	-11.928851106	-3161.15015
**major_TS2-c4**	-3148.168054423	-3148.951663987	-3149.206506538	-8.775440768	-10.771877222	-11.938467552	-3161.144974
**minor_TS2**	-3148.209500774	-3148.990916285	-3149.245045294	-8.755708696	-10.751434603	-11.917609735	-3161.151609
**major_INT2**	-3148.204604326	-3149.001066214	-3149.260088550	-8.768032799	-10.763943939	-11.930227310	-3161.190316
**major_INT3**	-3148.214708086	-3149.013755581	-3149.273618799	-8.757724218	-10.754051614	-11.920578217	-3161.194197
**major_INT4**	-3148.206734153	-3148.989408987	-3149.243947549	-8.758592967	-10.754960817	-11.921511059	-3161.165459
**minor_INT2**	-3148.203946802	-3149.001562434	-3149.260959986	-8.759615265	-10.755816345	-11.922269138	-3161.183229
**minor_INT3**	-3148.224743854	-3149.022932938	-3149.282516986	-8.753887927	-10.749163877	-11.915076083	-3161.197593
**minor_INT4**	-3148.225126187	-3149.002381899	-3149.255158075	-8.751976839	-10.747889320	-11.914173473	-3161.169332

Supplementary Table 6. Raw energy values obtained at SMD(toluene)-DLPNO-CCSD(T)/aug-cc-pV(DT)Z basis sets and the complete basis set (CBS) extrapolation. Final single-point (SP) energy = Extrapolated SCF energy + Extrapolated correlation energy. α = 4.3 and β = 2.51 in the extrapolation of SCF and correlation energies. All values have the units of a.u.

Structure	SCF with SMD correction			Correlation			Final SP Energy
	aug-cc-pVDZ	aug-cc-pVTZ	Extrapolated	aug-cc-pVDZ	aug-cc-pVTZ	Extrapolated	
**model_system**							
**Et_2NH_2Ms**	-799.7758716	-799.9109926	-799.9572291	-1.659181162	-1.971594274	-2.148411339	-802.1056405
**model_imine**	-1292.408135117	-1292.662054645	-1292.74894238804	-3.353900919	-3.965305314	-4.311343064947602	-1297.060285
**model_NHC**	-357.9392753 80	-358.0199364 05	-358.0475374 89756	-1.323241590	-1.557165410	-1.6895597356342993	-359.7370972
**model_INT1**	-1650.338229680	-1650.668620860	-1650.7816761 4242	-4.712067730	-5.555390570	-6.032687660539143	-1656.814364
**model_TS1**	-1650.310912401	-1650.640011619	-1650.75262480993	-4.727326343	-5.567849786	-6.043562496299464	-1656.796187
**model_INT2**	-1650.341098744	-1650.668968362	-1650.78116080085	-4.737187838	-5.579968453	-6.056958659386785	-1656.838119
**model_INT3**	-1650.315461981	-1650.645849460	-1650.75890347599	-4.740759689	-5.583729768	-6.0608272057004315	-1656.819731
**model_TS2**	-1650.280608887	-1650.594754261	-1650.70225045378	-4.763910398	-5.606086280	-6.082734224458418	-1656.784985
**model_INT4**	-1650.339200377	-1650.649982339	-1650.75632761881	-4.744216551	-5.585749661	-6.062033814522222	-1656.818361
**model_INT3'**	-1862.679457	-1863.055777	-1863.184548	-5.651295149	-6.642073905	-7.202826911	-1870.387375
**model_TS2'**	-1862.601845	-1862.975486	-1863.103341	-5.680425641	-6.670776173	-7.231286816275702	-1870.334628
**diethylamine**	-212.3490230 14	-212.4013444 25	-212.4192480 86645	-0.880787770	-1.032243922	-1.1179638589743786	-213.5372119
**product**	-705.0099687 46	-705.1631117 99	-705.2155152 29103	-2.583703204	-3.033408046	-3.2879283821122827	-708.5034436

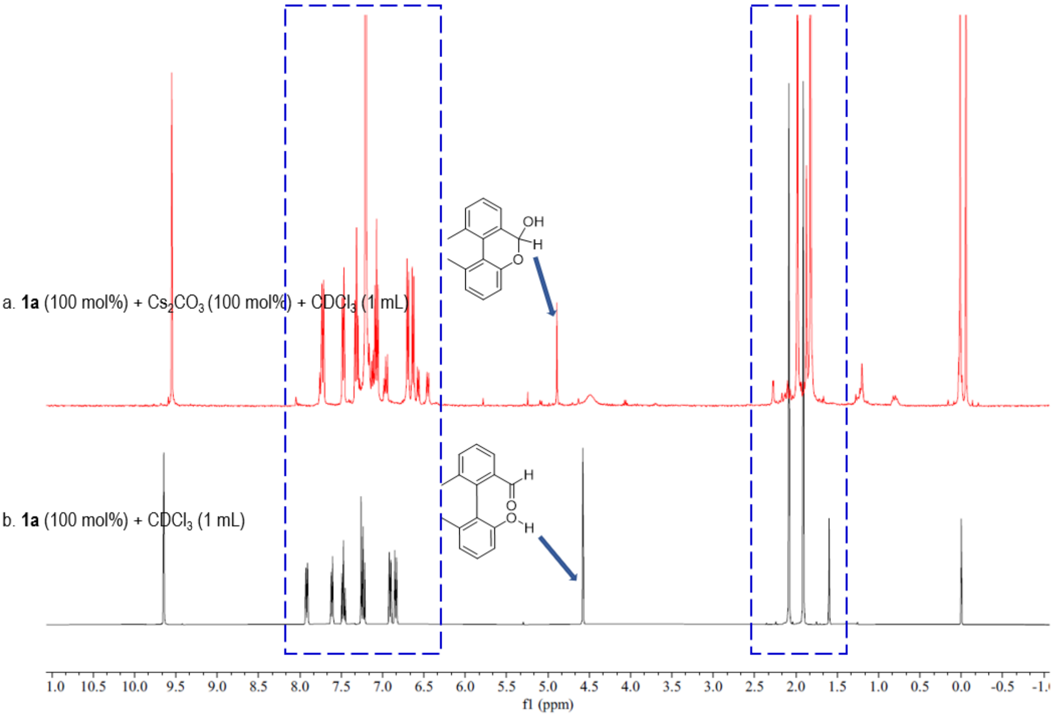

Supplementary Figure 8. Evidence of bridged hemiacetal **1a'** via ^{1}H NMR analysis

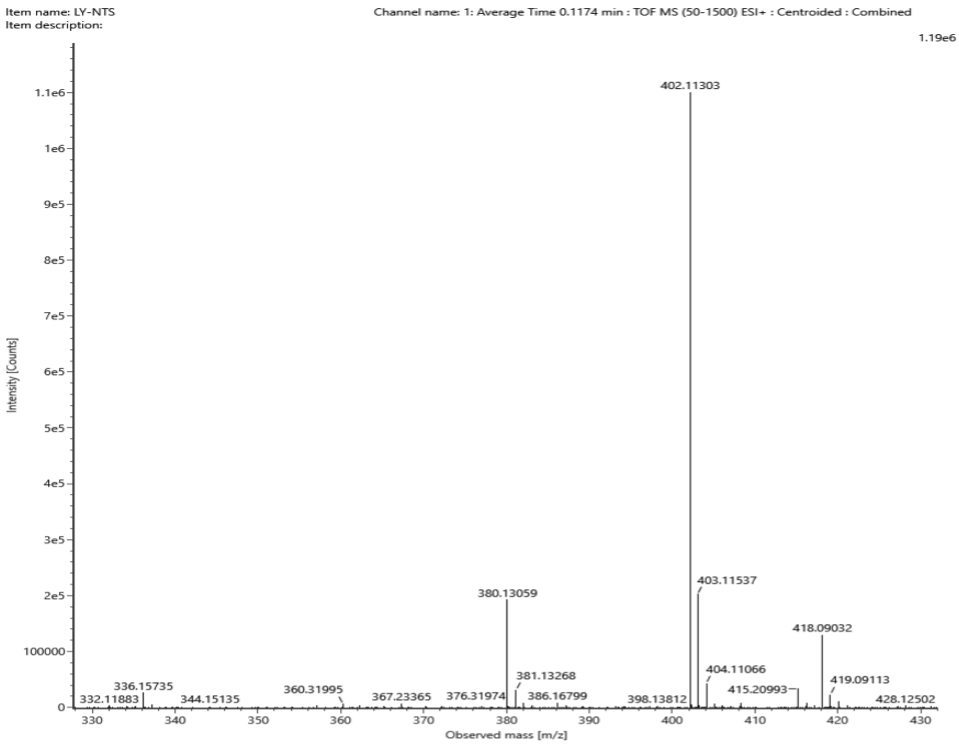

Supplementary Figure 9. Evidence of imine intermediate **4a.**

Characterization of substrates and products

2'-hydroxy-6,6'-dimethyl-[1,1'-biphenyl]-2-carbaldehyde (1a)

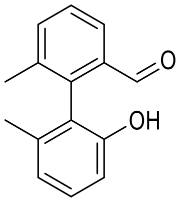

White solid, 17% yield over 4 steps, 1.4 g; m.p. 97-98 ^oC.

^{1}H NMR (400 MHz, $CDCl_3$) δ 9.63 (d, *J* = 0.8 Hz, 1H), 7.91 (d, *J* = 7.6 Hz, 1H), 7.61 (d, *J* = 7.6 Hz, 1H), 7.46 (t, *J* = 7.6 Hz, 1H), 7.23 (t, *J* = 8.0 Hz, 1H), 6.90 (d, *J* = 7.6 Hz, 1H), 6.84 (d, *J* = 8.4 Hz, 1H), 4.75 (s, 1H), 2.08 (s, 3H), 1.91 (s, 3H).

^{13}C NMR (101 MHz, $CDCl_3$) δ 192.9, 153.0, 139.4, 138.9, 137.9, 136.5, 134.8, 129.6, 128.8, 125.6, 122.6, 122.5, 113.2, 20.2, 19.2.

HRMS (ESI, m/z) calcd. for $C_{15}H_{14}O_2H^+$ $[M+H]^+$: 227.1067, found: 227.1073.

2'-hydroxy-4',6,6'-trimethyl-[1,1'-biphenyl]-2-carbaldehyde (1b)

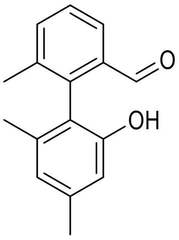

White solid, 22% yield over 4 steps, 720 mg; m.p. 75-76 ^oC.

^{1}H NMR (400 MHz, $CDCl_3$) δ 9.7 (d, *J* = 0.7 Hz, 1H), 7.9 (d, *J* = 7.2 Hz, 1H), 7.6 (d, *J* = 7.5 Hz, 1H), 7.5 (t, *J* = 7.7 Hz, 1H), 6.7 (s, 1H), 6.7 (s, 1H), 4.5 (s, 1H), 2.3 (s, 3H), 2.1 (s, 3H), 1.9 (s, 3H).

^{13}C NMR (101 MHz, $CDCl_3$) δ 193.2, 152.9, 139.7, 139.6, 139.2, 137.5, 136.4, 135.0, 128.6, 125.5, 123.4, 119.6, 113.8, 21.4, 20.2, 19.3.

HRMS (ESI, m/z) calcd. for $C_{16}H_{16}O_2H^+$ $[M+H]^+$: 241.1223, found: 241.1234.

6'-hydroxy-2',3',4',6-tetramethyl-[1,1'-biphenyl]-2-carbaldehyde (1c)

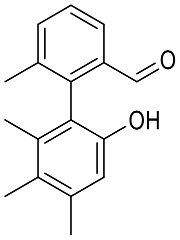

White solid, 6% yield over 4 steps, 220 mg; m.p. 124-125 ^oC.

^{1}H NMR (400 MHz, $CDCl_3$) δ 9.64 (d, *J* = 0.9 Hz, 1H), 7.90 (d, *J* = 7.7 Hz, 1H), 7.59 (d, *J* = 7.5 Hz, 1H), 7.45 (t, *J* = 7.6 Hz, 1H), 6.69 (s, 1H), 4.33 (s, 1H), 2.32 (s, 3H), 2.16 (s, 3H), 2.07 (s, 3H), 1.84 (s, 3H).

^{13}C NMR (101 MHz, $CDCl_3$) δ 192.8, 150.1, 140.1, 139.1, 137.9, 136.1, 135.5, 135.1, 128.5, 127.6, 125.3, 120.0, 114.4, 20.9, 19.3, 17.3, 15.3.

HRMS (ESI, m/z) calcd. for $C_{17}H_{18}O_2H^+$ $[M+H]^+$: 255.1380, found: 255.1382.

2'-hydroxy-3',4',6,6'-tetramethyl-[1,1'-biphenyl]-2-carbaldehyde (1d)

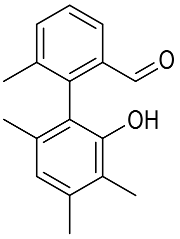

White solid, 11% yield over 4 steps, 390 mg; m.p.110-111 °C.

^{1}H NMR (400 MHz, $CDCl_3$) δ 9.62 (d, *J* = 0.8 Hz, 1H), 7.90 (dd, *J* = 7.7, 0.6 Hz, 1H), 7.60 (dd, *J* = 7.5, 0.6 Hz, 1H), 7.46 (t, *J* = 7.7 Hz, 1H), 6.73 (s, 1H), 4.38 (s, 1H), 2.30 (s, 3H), 2.17 (s, 3H), 2.08 (s, 3H), 1.83 (s, 3H).

^{13}C NMR (101 MHz, $CDCl_3$) δ 192.8, 150.1, 140.1, 139.1, 137.9, 136.1, 135.5, 135.1, 128.5, 127.6, 125.3, 120.0, 114.4, 20.9, 19.3, 17.3, 15.3.

HRMS (ESI, m/z) calcd. for $C_{17}H_{18}O_2H^+$ $[M+H]^+$: 255.1380, found: 255.1387.

2'-hydroxy-4',6'-dimethoxy-6-methyl-[1,1'-biphenyl]-2-carbaldehyde (1e)

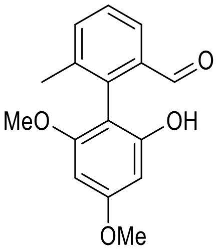

White solid, 14 % yield over 4 steps, 540 mg; m.p. 178-179 °C.

^{1}H NMR (400 MHz, Acetone-d_6) δ 8.36 (s, 1H), 7.57 (d, *J* = 7.7 Hz, 1H), 7.53 (d, *J* = 7.6 Hz, 1H), 7.36 (t, *J* = 7.7 Hz, 1H), 6.27 (s, 2H), 6.25 (d, *J* = 1.4 Hz, 2H), 3.80 (s, 3H), 3.71 (s, 3H), 2.13 (s, 3H).

^{13}C NMR (101 MHz, Acetone-d_6) δ 192.5, 161.7, 158.8, 156.0, 139.5, 138.8, 135.2, 134.9, 127.2, 123.5, 104.4, 93.7, 90.2, 55.1, 54.7, 18.7.

HRMS (ESI, m/z) calcd. for $C_{16}H_{16}O_4H^+$ $[M+H]^+$: 273.1121, found: 273.1131.

3'-chloro-6'-hydroxy-2',4',6-trimethyl-[1,1'-biphenyl]-2-carbaldehyde (1f)

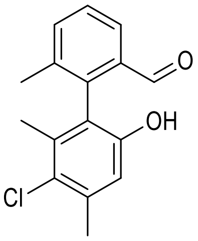

White solid, 8% yield over 4 steps, 320 mg; m.p. 125-126 °C.

^{1}H NMR (400 MHz, $CDCl_3$) δ 9.58 (s, 1H), 7.86 (d, *J* = 7.6 Hz, 1H), 7.58 (d, *J* = 7.4 Hz, 1H), 7.43 (t, *J* = 7.7 Hz, 1H), 6.76 (s, 1H), 5.18 (s, 1H), 2.39 (s, 3H), 2.06 (s, 3H), 1.96 (s, 3H).

^{13}C NMR (101 MHz, $CDCl_3$) δ 192.7, 151.0, 139.1, 138.9, 137.6, 136.4, 135.4, 134.7, 128.8, 126.9, 125.6, 121.5, 115.5, 21.0, 19.2, 18.1.

HRMS (ESI, m/z) calcd. for $C_{16}H_{15}ClO_2H^+$ $[M+H]^+$: 275.0833, found: 275.0835.

2'-hydroxy-3',6,6'-trimethyl-[1,1'-biphenyl]-2-carbaldehyde (1g)

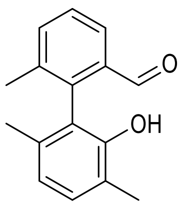

White solid, 15% yield over 4 steps, 490 mg; m.p. 109-110 ^{o}C.

^{1}H NMR (400 MHz, $CDCl_3$) δ 9.61 (d, *J* = 0.6 Hz, 1H), 7.91 (d, *J* = 7.7 Hz, 1H), 7.61 (d, *J* = 7.5 Hz, 1H), 7.46 (t, *J* = 7.7 Hz, 1H), 7.10 (d, *J* = 7.6 Hz, 1H), 6.81 (d, *J* = 7.6 Hz, 1H), 4.45 (s, 1H), 2.26 (s, 3H), 2.07 (s, 3H), 1.86 (s, 3H).

^{13}C NMR (101 MHz, $CDCl_3$) δ 192.5, 150.8, 139.0, 139.0, 136.3, 135.0, 134.8, 130.7, 128.8, 125.5, 121.8, 121.8, 121.5, 19.9, 19.1, 15.8.

HRMS (ESI, m/z) calcd. for $C_{16}H_{16}O_2H^+$ [M+H]$^+$: 241.1223, found: 241.1230.

2'-hydroxy-3'-methoxy-6,6'-dimethyl-[1,1'-biphenyl]-2-carbaldehyde (1h)

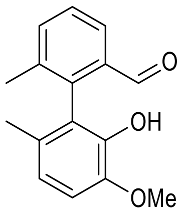

White solid, 11% yield over 4 steps, 396 mg; m.p. 69-70 ^{o}C.

^{1}H NMR (400 MHz, $CDCl_3$) δ 9.65 (d, *J* = 0.9 Hz, 1H), 7.87 (d, *J* = 7.7 Hz, 1H), 7.54 (d, *J* = 7.5 Hz, 1H), 7.39 (t, *J* = 7.6 Hz, 1H), 6.86 – 6.77 (m, 2H), 5.76 (s, 1H), 3.88 (s, 3H), 2.07 (s, 3H), 1.85 (s, 3H).

^{13}C NMR (101 MHz, $CDCl_3$) δ 192.9, 144.6, 142.8, 140.6, 137.9, 135.6, 133.9, 129.3, 127.8, 124.6, 122.4, 120.6, 110.1, 55.9, 19.3, 19.0.

HRMS (ESI, m/z) calcd. for $C_{16}H_{16}O_3H^+$ [M+H]$^+$: 257.1172, found: 257.1174.

2'-hydroxy-3'-isopropyl-6,6'-dimethyl-[1,1'-biphenyl]-2-carbaldehyde (1i)

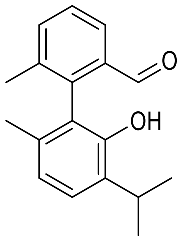

White solid, 9% yield over 4 steps, 340 mg; m.p. 86-87 ^{o}C.

^{1}H NMR (400 MHz, $CDCl_3$) δ 9.63 (d, *J* = 0.5 Hz, 1H), 7.93 (d, *J* = 7.6 Hz, 1H), 7.62 (d, *J* = 7.4 Hz, 1H), 7.48 (t, *J* = 7.7 Hz, 1H), 7.18 (d, *J* = 7.8 Hz, 1H), 6.87 (d, *J* = 7.9 Hz, 1H), 4.36 (s, 1H), 3.18 – 3.28 (m, 1H), 2.08 (s, 3H), 1.86 (s, 3H), 1.27 (d, *J* = 2.1 Hz, 3H), 1.25 (d, *J* = 2.1 Hz, 3H).

^{13}C NMR (101 MHz, $CDCl_3$) δ 192.6, 149.9, 139.3, 139.1, 136.5, 135.2, 134.5, 132.4, 129.1, 126.3, 125.7, 122.2, 121.9, 27.2, 22.8, 22.7, 20.0, 19.2.

HRMS (ESI, m/z) calcd. for $C_{18}H_{20}O_2H^+$ [M+H]$^+$: 269.1536, found: 269.1544.

3'-chloro-2'-hydroxy-6,6'-dimethyl-[1,1'-biphenyl]-2-carbaldehyde (1j)

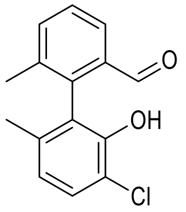

White solid, 9% yield over 4 steps, 320 mg; m.p. 77-78 ^oC.

^{1}H NMR (400 MHz, $CDCl_3$) δ 9.63 (d, *J* = 0.8 Hz, 1H), 7.89 (d, *J* = 7.7 Hz, 1H), 7.58 (d, *J* = 7.5 Hz, 1H), 7.45 (t, *J* = 7.6 Hz, 1H), 7.31 (d, *J* = 8.2 Hz, 1H), 6.87 (d, *J* = 8.2 Hz, 1H), 5.52 (s, 1H), 2.06 (s, 3H), 1.89 (s, 3H).

^{13}C NMR (101 MHz, $CDCl_3$) δ 192.3, 148.5, 139.5, 137.9, 137.2, 136.0, 134.0, 128.5, 128.4, 125.4, 124.2, 122.7, 117.6, 19.8, 19.1.

HRMS (ESI, m/z) calcd. for $C_{15}H_{13}ClO_2H^+$ $[M+H]^+$: 261.0677, found: 261.0683.

2'-hydroxy-6'-methoxy-6-methyl-[1,1'-biphenyl]-2-carbaldehyde (1k)

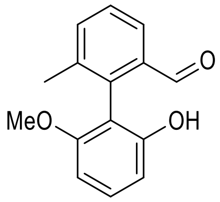

White solid, 9% yield over 4 steps, 420 mg; m.p. 155-156 ^oC.

^{1}H NMR (400 MHz, Acetone-d_6) δ 9.70 (s, 1H), 8.35 (s, 1H), 7.75 (d, *J* = 7.5 Hz, 1H), 7.56 (d, *J* = 7.4 Hz, 1H), 7.38 (t, *J* = 7.6 Hz, 1H), 7.28 (t, *J* = 8.3 Hz, 1H), 6.68 (t, *J* = 8.1 Hz, 2H), 3.68 (s, 3H), 2.10 (s, 3H).

^{13}C NMR (101 MHz, Acetone-d_6) δ 192.3, 158.2, 155.5, 138.9, 138.9, 135.0, 134.7, 129.9, 127.3, 123.6, 111.8, 108.5, 102.4, 55.2, 18.6.

HRMS (ESI, m/z) calcd. for $C_{15}H_{14}O_3H^+$ $[M+H]^+$: 243.1016, found: 243.1018.

2'-chloro-6'-hydroxy-6-methyl-[1,1'-biphenyl]-2-carbaldehyde (1l)

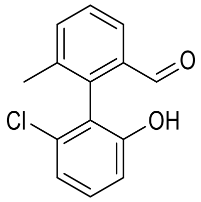

White solid, 20% yield over 4 steps, 1.6 g; m.p. 105-106 ^oC.

^{1}H NMR (400 MHz, $CDCl_3$) δ 9.66 (d, *J* = 0.8 Hz, 1H), 7.91 (d, *J* = 7.7 Hz, 1H), 7.63 (d, *J* = 7.5 Hz, 1H), 7.51 (t, *J* = 7.7 Hz, 1H), 7.29 (t, *J* = 8.1 Hz, 1H), 7.13 (dd, *J* = 8.1, 1.1 Hz, 1H), 6.95 (dd, *J* = 8.2, 1.1 Hz, 1H), 5.26 (s, 1H), 2.14 (s, 3H).

^{13}C NMR (101 MHz, $CDCl_3$) δ 192.1, 154.2, 139.1, 136.7, 136.3, 134.6, 134.3, 130.4, 129.3, 125.9, 122.3, 121.7, 114.2, 19.1.

HRMS (ESI, m/z) calcd. for $C_{14}H_{11}ClO_2H^+$ $[M+H]^+$: 247.0520, found: 247.0526.

2'-hydroxy-4',5,6,6'-tetramethyl-[1,1'-biphenyl]-2-carbaldehyde (1m)

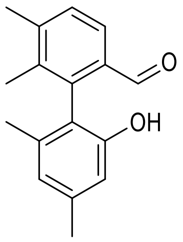

White solid, 10% yield over 4 steps, 340 mg; m.p. 137-138 °C.

^{1}H NMR (400 MHz, $CDCl_3$) δ 9.55 (d, *J* = 0.8 Hz, 1H), 7.82 (d, *J* = 7.9 Hz, 1H), 7.33 (d, *J* = 7.9 Hz, 1H), 6.72 (s, 1H), 6.66 (s, 1H), 4.68 (s, 1H), 2.41 (s, 3H), 2.33 (s, 3H), 1.98 (s, 3H), 1.85 (s, 3H).

^{13}C NMR (101 MHz, $CDCl_3$) δ 192.7, 152.8, 144.8, 139.4, 138.9, 137.5, 137.3, 133.1, 130.4, 125.3, 123.2, 119.9, 113.5, 21.3, 21.3, 20.1, 15.5.

HRMS (ESI, m/z) calcd. for $C_{17}H_{18}O_2H^+$ $[M+H]^+$: 255.1379, found: 255.1381.

2'-hydroxy-4,4',6,6'-tetramethyl-[1,1'-biphenyl]-2-carbaldehyde (1n)

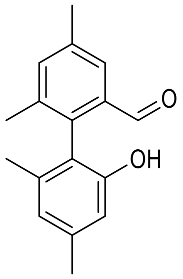

White solid, 10% yield over 4 steps, 367 mg; m.p. 134-135 °C.

^{1}H NMR (400 MHz, $CDCl_3$) δ 9.62 (s, 1H), 7.71 (s, 1H), 7.42 (s, 1H), 6.72 (s, 1H), 6.66 (s, 1H), 4.59 (s, 1H), 2.42 (s, 3H), 2.33 (s, 3H), 2.05 (s, 3H), 1.87 (s, 3H).

^{13}C NMR (101 MHz, $CDCl_3$) δ 193.1, 152.9, 139.6, 139.0, 138.7, 137.7, 137.4, 136.3, 135.0, 125.9, 123.4, 119.5, 113.6, 21.4, 21.2, 20.2, 19.2.

HRMS (ESI, m/z) calcd. for $C_{17}H_{18}O_2H^+$ $[M+H]^+$: 255.1380, found: 255.1385.

4-chloro-2'-hydroxy-4',6,6'-trimethyl-[1,1'-biphenyl]-2-carbaldehyde (1o)

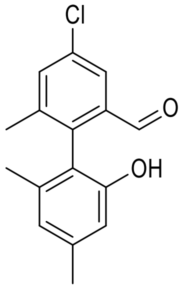

White solid, 10% yield over 4 steps, 318 mg; m.p. 46-47 °C.

^{1}H NMR (400 MHz, $CDCl_3$) δ 9.51 (s, 1H), 7.78 (d, *J* = 2.1 Hz, 1H), 7.52 (d, *J* = 1.7 Hz, 1H), 6.70 (s, 1H), 6.58 (s, 1H), 5.94 (s, 1H), 2.29 (s, 3H), 2.05 (s, 3H), 1.84 (s, 3H).

^{13}C NMR (101 MHz, $CDCl_3$) δ 192.6, 153.0, 141.2, 139.8, 139.3, 137.5, 135.8, 135.6, 134.4, 124.9, 123.3, 118.6, 113.9, 21.3, 20.0, 19.1.

HRMS (ESI, m/z) calcd. for $C_{16}H_{15}ClO_2H^+$ $[M+H]^+$: 275.0833, found: 275.0823.

2'-hydroxy-6-methoxy-4',6'-dimethyl-[1,1'-biphenyl]-2-carbaldehyde (1p)

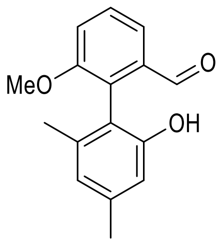

White solid, 17% yield over 4 steps, 580 mg; m.p. 130-131 ^oC.

<u>^{1}H NMR</u> (400 MHz, Acetone-d_6) δ 9.66 (d, J = 0.7 Hz, 1H), 7.98 (s, 1H), 7.55 – 7.46 (m, 2H), 7.36 (dd, J = 7.8, 1.5 Hz, 1H), 6.66 (d, J = 6.2 Hz, 2H), 3.78 (s, 3H), 2.27 (s, 3H), 1.92 (s, 3H).

<u>^{13}C NMR</u> (101 MHz, Acetone-d_6) δ 191.9, 157.8, 154.8, 138.5, 138.4, 135.4, 130.8, 128.7, 122.0, 118.0, 117.4, 116.3, 113.3, 55.3, 20.4, 19.3.

<u>HRMS</u> (ESI, m/z) calcd. for $C_{16}H_{16}O_3H^+$ $[M+H]^+$: 257.1172, found 257.1174.

6-ethyl-2'-hydroxy-6'-methyl-[1,1'-biphenyl]-2-carbaldehyde (1q)

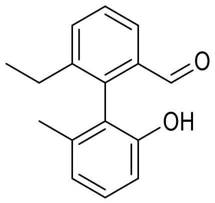

Colourless oil, 8% yield over 4 steps, 720 mg.

<u>^{1}H NMR</u> (400 MHz, $CDCl_3$) δ 9.57 (d, J = 0.6 Hz, 1H), 7.88 (dd, J = 7.7, 1.2 Hz, 1H), 7.63 (d, J = 7.6 Hz, 1H), 7.47 (t, J = 7.7 Hz, 1H), 7.22 (t, J = 7.9 Hz, 1H), 6.88 (d, J = 7.6 Hz, 1H), 6.83 (d, J = 8.1 Hz, 1H), 5.21 (s, 1H), 2.39 (q, J = 7.6 Hz, 2H), 1.90 (s, 3H), 1.07 (t, J = 7.6 Hz, 3H).

<u>^{13}C NMR</u> (101 MHz, $CDCl_3$) δ 193.4, 153.7, 145.0, 139.2, 138.4, 135.2, 135.0, 129.9, 129.3, 125.9, 122.7, 122.7, 113.5, 26.0, 20.7, 15.1.

<u>HRMS</u> (ESI, m/z) calcd. for $C_{16}H_{16}O_2H^+$ $[M+H]^+$: 241.1223, found: 241.1232.

6-ethyl-2'-hydroxy-6'-methoxy-[1,1'-biphenyl]-2-carbaldehyde (1r)

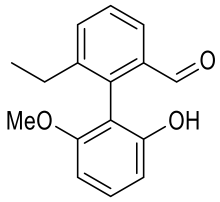

White solid, 12% yield over 4 steps, 970 mg; m.p. 111-113 ^oC.

<u>^{1}H NMR</u> (400 MHz, Acetone-d_6) δ 9.68 (d, J = 0.7 Hz, 1H), 8.34 (s, 1H), 7.76 (dd, J = 7.7, 1.3 Hz, 1H), 7.61 (dd, J = 7.6, 1.2 Hz, 1H), 7.44 (t, J = 7.6 Hz, 1H), 7.28 (t, J = 8.3 Hz, 1H), 6.68 (dd, J = 8.6, 4.7 Hz, 2H), 3.68 (s, 3H), 2.45 (q, J = 7.6 Hz, 2H), 1.04 (t, J = 7.6 Hz, 3H).

<u>^{13}C NMR</u> (101 MHz, Acetone-d_6) δ 192.3, 158.4, 155.7, 144.8, 138.3, 134.8, 133.7, 129.9, 127.6, 123.7, 111.6, 108.4, 102.3, 55.1, 25.7, 14.2.

<u>HRMS</u> (ESI, m/z) calcd. for $C_{16}H_{16}O_3H^+$ $[M+H]^+$: 257.1172, found: 257.1174.

2'-hydroxy-4,6,6'-trimethyl-[1,1'-biphenyl]-2-carbaldehyde (1s)

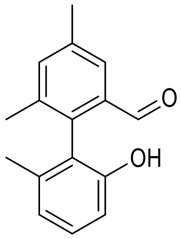

White solid, 14% yield over 4 steps, 1.2 g; m.p. 120-121 °C.

^{1}H NMR (400 MHz, $CDCl_3$) δ 9.58 (s, 1H), 7.70 (s, 1H), 7.42 (s, 1H), 7.20 (t, *J* = 7.9 Hz, 1H), 6.88 (d, *J* = 7.5 Hz, 1H), 6.82 (d, *J* = 8.1 Hz, 1H), 5.09 (s, 1H), 2.41 (s, 3H), 2.04 (s, 3H), 1.90 (s, 3H).

^{13}C NMR (101 MHz, $CDCl_3$) δ 193.0, 153.1, 138.6, 138.6, 137.9, 137.4, 136.4, 134.5, 129.3, 125.8, 122.5, 122.2, 112.9, 21.1, 20.1, 19.0.

HRMS (ESI, m/z) calcd. for $C_{16}H_{16}O_2H^+$ $[M+H]^+$: 241.1223, found: 241.1231.

2'-hydroxy-6'-methoxy-4,6-dimethyl-[1,1'-biphenyl]-2-carbaldehyde (1t)

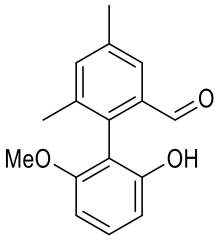

White solid, 10% yield over 4 steps, 850 mg; m.p. 181-183 °C.

^{1}H NMR (400 MHz, Acetone-*d*$_6$) δ 9.67 (s, 1H), 8.29 (s, 1H), 7.56 (s, 1H), 7.38 (s, 1H), 7.26 (t, *J* = 8.3 Hz, 1H), 6.67 (dd, *J* = 12.9, 4.6 Hz, 2H), 3.68 (s, 3H), 2.38 (s, 3H), 2.06 (s, 3H).

^{13}C NMR (101 MHz, Acetone-*d*$_6$) δ 192.3, 158.3, 155.6, 138.8, 136.9, 136.0, 136.0, 134.6, 129.7, 123.9, 111.9, 108.5, 102.4, 55.1, 20.1, 18.5.

HRMS (ESI, m/z) calcd. for $C_{16}H_{16}O_3H^+$ $[M+H]^+$: 257.1172, found: 257.1174.

1-(2-hydroxy-4,6-dimethylphenyl)-2-naphthaldehyde (1u)

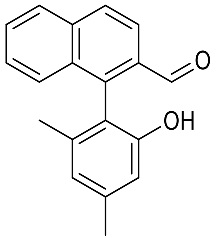

White solid, 23% yield over 4 steps, 750 mg; m.p. 142-143 °C.

^{1}H NMR (400 MHz, $CDCl_3$) δ 9.87 (d, *J* = 0.7 Hz, 1H), 8.10 (d, *J* = 8.6 Hz, 1H), 7.98 (dd, *J* = 11.1, 8.5 Hz, 2H), 7.70 – 7.59 (m, 2H), 7.47 – 7.52(m, 1H), 6.83 (s, 1H), 6.75 (s, 1H), 4.70 (s, 1H), 2.42 (s, 3H), 1.86 (s, 3H).

^{13}C NMR (101 MHz, $CDCl_3$) δ 192.8, 153.6, 140.9, 140.1, 138.6, 136.6, 132.2, 132.2, 129.3, 129.1, 128.6, 127.5, 126.5, 123.4, 122.4, 118.0, 113.8, 21.4, 20.1.

HRMS (ESI, m/z) calcd. for $C_{19}H_{16}O_2H^+$ $[M+H]^+$: 277.1223, found: 277.1231.

1-(2-hydroxy-4,6-dimethoxyphenyl)-2-naphthaldehyde (1v)

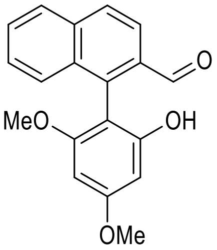

White solid, 18% yield over 4 steps, 660 mg; m.p. 172-173 °C.

^{1}H NMR (400 MHz, Acetone-*d*$_6$) δ 9.96 (s, 1H), 8.34 (s, 1H), 7.98 (d, *J* = 8.0 Hz, 3H), 7.69 (d, *J* = 8.5 Hz, 1H), 7.65 – 7.60 (m, 1H), 7.48 (m, 1H), 6.38 – 6.34 (m, 2H), 3.87 (s, 3H), 3.61 (s, 3H).

^{13}C NMR (101 MHz, Acetone-*d*$_6$) δ 192.4, 162.2, 159.6, 156.9, 140.4, 136.5, 133.3, 132.3, 128.5, 128.2, 127.9, 127.1, 126.5, 121.7, 102.8, 93.8, 90.3, 55.2, 54.8.

HRMS (ESI, m/z) calcd. for $C_{19}H_{16}O_4H^+$ [M+H]$^+$: 309.1121, found: 309.1125.

1-(2-hydroxy-3,4,6-trimethylphenyl)-2-naphthaldehyde (1w)

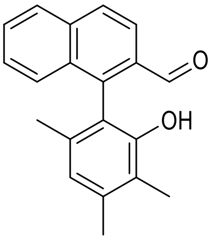

White solid, 13% yield over 4 steps, 440 mg; m.p. 167-168 °C.

^{1}H NMR (400 MHz, $CDCl_3$) δ 9.86 (d, *J* = 0.6 Hz, 1H), 8.12 (d, *J* = 8.6 Hz, 1H), 7.99 (dd, *J* = 15.5, 8.4 Hz, 2H), 7.65 – 7.69 (m, 1H), 7.62 (d, *J* = 8.4 Hz, 1H), 7.48 – 7.52 (m, 1H), 6.83 (s, 1H), 4.40 (s, 1H), 2.39 (s, 3H), 2.23 (s, 3H), 1.82 (s, 3H).

^{13}C NMR (101 MHz, $CDCl_3$) δ 192.6, 151.5, 140.8, 138.5, 136.7, 134.8, 132.4, 132.3, 129.4, 129.3, 128.6, 127.6, 126.5, 123.7, 122.5, 120.2, 117.9, 20.1, 19.8, 11.7.

HRMS (ESI, m/z) calcd. for $C_{20}H_{18}O_2H^+$ [M+H]$^+$: 291.1380, found: 291.1392.

1-(2-hydroxy-3-isopropyl-6-methylphenyl)-2-naphthaldehyde (1x)

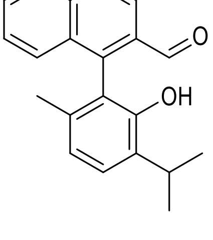

White solid, 25% yield over 4 steps, 920 mg; m.p. 178-179 °C.

^{1}H NMR (400 MHz, $CDCl_3$) δ 9.82 (d, *J* = 0.6 Hz, 1H), 8.11 (d, *J* = 8.6 Hz, 1H), 8.00 (d, *J* = 8.7 Hz, 1H), 7.96 (d, *J* = 8.2 Hz, 1H), 7.69 – 7.63 (m, 1H), 7.57 (d, *J* = 8.4 Hz, 1H), 7.52 – 7.46 (m, 1H), 7.28 (d, *J* = 7.9 Hz, 1H), 6.95 (d, *J* = 7.9 Hz, 1H), 4.39 (s, 1H), 3.20 – 3.30 (m, 1H), 1.82 (s, 3H), 1.32 – 1.26 (m, 6H).

^{13}C NMR (101 MHz, $CDCl_3$) δ 192.6, 150.9, 140.7, 136.8, 135.8, 132.5, 132.5, 132.3, 129.6, 129.5, 128.8, 127.9, 126.7, 126.6, 122.7, 122.2, 120.6, 27.2, 22.9, 22.8, 20.1.

HRMS (ESI, m/z) calcd. for $C_{21}H_{20}O_2H^+$ [M+H]$^+$: 305.1536, found: 305.1543.

1-(2-hydroxy-3,6-dimethylphenyl)-2-naphthaldehyde (1y)

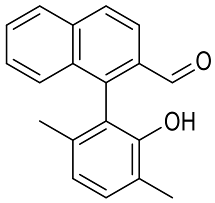

White solid, 11% yield over 4 steps, 350 mg; m.p. 162-163 °C.

^{1}H NMR (400 MHz, $CDCl_3$) δ 9.82 (d, *J* = 0.7 Hz, 1H), 8.10 (d, *J* = 8.6 Hz, 1H), 7.97 (dd, *J* = 16.2, 8.4 Hz, 2H), 7.63 – 7.67 (m, 1H), 7.56 (d, *J* = 8.4 Hz, 1H), 7.46 – 7.50 (m, 1H), 7.20 (d, *J* = 7.7 Hz, 1H), 6.88 (d, *J* = 7.7 Hz, 1H), 4.40 (s, 1H), 2.29 (s, 3H), 1.83 (s, 3H).

^{13}C NMR (101 MHz, $CDCl_3$) δ 192.6, 151.9, 140.7, 136.8, 136.2, 132.4, 132.2, 131.3, 129.6, 129.5, 128.8, 127.8, 126.6, 122.7, 122.0, 121.8, 120.6, 20.1, 16.0.

HRMS (ESI, m/z) calcd. for $C_{19}H_{16}O_2H^+$ $[M+H]^+$: 277.1223, found: 277.1231.

2'-methoxy-6,6'-dimethyl-[1,1'-biphenyl]-2-carbaldehyde (1z)

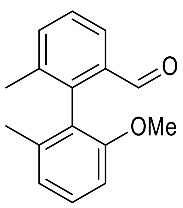

White solid, 97% yield, 310 mg; m.p. 40-41 °C.

^{1}H NMR (400 MHz, $CDCl_3$) δ 9.6 (d, *J* = 0.9 Hz, 1H), 7.9 (d, *J* = 6.9 Hz, 1H), 7.5 (d, *J* = 7.1 Hz, 1H), 7.4 (t, *J* = 7.6 Hz, 1H), 7.3 (t, *J* = 8.0 Hz, 1H), 6.9 (d, *J* = 7.7 Hz, 1H), 6.8 (d, *J* = 8.3 Hz, 1H), 3.7 (s, 3H), 2.0 (s, 3H), 1.9 (s, 3H).

^{13}C NMR (101 MHz, $CDCl_3$) δ 193.2, 156.9, 141.7, 138.0, 137.8, 135.6, 134.1, 129.0, 127.6, 124.8, 124.6, 122.5, 108.1, 55.6, 20.0, 19.2.

HRMS (ESI, m/z) calcd. for $C_{16}H_{16}O_2H^+$ $[M+H]^+$: 241.1223, found: 241.1218.

(5a*R*,10b*S*)-2-(2,3,4,5,6-pentafluorophenyl)-9-nitro-5a,10b-dihydro-4H,6H-indeno[2,1-*b*][1,2,4]triazolo[4,3-*d*][1,4]oxazin-2-ium chloride (NHC-**G**)

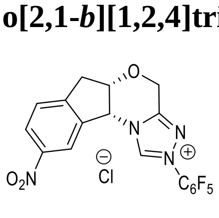

White solid, 20% yield, 800 mg; m.p. 200-201 °C.

^{1}H NMR (400 MHz, DMSO-d_6) δ 12.17 (s, 1H), 8.52 (s, 1H), 8.29 (dd, *J* = 8.4, 2.2 Hz, 1H), 7.73 (d, *J* = 8.4 Hz, 1H), 6.40 (d, *J* = 4.1 Hz, 1H), 5.38 (d, *J* = 16.3 Hz, 1H), 5.13 (d, *J* = 16.3 Hz, 1H), 5.07 (t, *J* = 4.5 Hz, 1H), 3.64 (dd, *J* = 18.0, 4.9 Hz, 1H), 3.30 (d, *J* = 17.9 Hz, 1H).

^{13}C NMR (101 MHz, DMSO-d_6) δ 151.2, 149.5, 147.7, 147.6, δ 144.1 – 143.4 (m), 141.7 – 140.7 (m), 140.0 – 139.2 (m), 137.8, 137.3 – 136.7 (m), 127.1, 125.4, 121.1, 77.6, 61.7, 60.2, 37.6.

^{19}F NMR (377 MHz, DMSO-d_6) δ -145.2 – -145.5 (m, 2F), -148.2 (t, 1F, J = 23.2 Hz), -159.7 – -160.1 (m, 2F).

HRMS (ESI, m/z) calcd. for $C_{18}H_{10}ClF_5N_4O_3H^+$ $[M+H]^+$:460.0362, found: 460.0352;

(*S*)-2'-hydroxy-6,6'-dimethyl-[1,1'-biphenyl]-2-carbonitrile (3a)

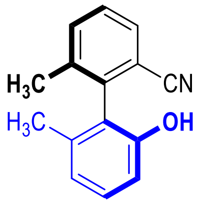

White solid, 96% yield, 21.6 mg; m.p. 170-171 °C.

$[\alpha]^{25}_D$ = +33.0 (c = 0.5 in MeOH);

^{1}H NMR (400 MHz, $CDCl_3$) δ 7.62 (d, J = 7.7 Hz, 1H), 7.58 – 7.53 (m, 1H), 7.39 (t, J = 7.7 Hz, 1H), 7.21 (t, J = 7.9 Hz, 1H), 6.91 (d, J = 7.6 Hz, 1H), 6.76 (d, J = 8.1 Hz, 1H), 4.84 (s, 1H), 2.11 (s, 3H), 1.98 (s, 3H).

^{13}C NMR (101 MHz, $CDCl_3$) δ 192.9, 153.0, 139.4, 138.9, 137.9, 136.5, 134.8, 129.6, 128.8, 125.6, 122.6, 122.5, 113.2, 20.2, 19.2.

HRMS (ESI, m/z) calcd. for $C_{15}H_{13}NOH^+$ $[M+H]^+$: 224.1070, found: 224.1073;

HPLC analysis: 98:2 er (IB column, 25 °C, n-hexane / i-PrOH = 96 / 4, 0.4 mL / min, λ = 254 nm), Rt (major) = 32.9 min, Rt (minor) = 40.9 min.

(*S*)-2'-hydroxy-4',6,6'-trimethyl-[1,1'-biphenyl]-2-carbonitrile (3b)

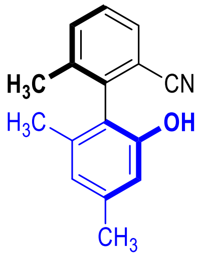

White solid, 97% yield, 23.0 mg; m.p. 82-83 °C.

$[\alpha]^{25}_D$ = +17.4 (c = 1.0 in $CHCl_3$).

^{1}H NMR (400 MHz, $CDCl_3$) δ 7.62 (d, J = 7.7 Hz, 1H), 7.55 (d, J = 7.7 Hz, 1H), 7.39 (t, J = 7.7 Hz, 1H), 6.74 (s, 1H), 6.62 (s, 1H), 4.54 (s, 1H), 2.32 (s, 3H), 2.11 (s, 3H), 1.95 (s, 3H).

^{13}C NMR (101 MHz, $CDCl_3$) δ 152.1, 140.0, 139.8, 139.5, 136.9, 134.6, 130.9, 128.4, 123.7, 121.0, 118.0, 114.6, 113.9, 21.3, 19.7, 19.5.

HRMS (ESI, m/z) calcd. for $C_{16}H_{15}NOH^+$ $[M+H]^+$: 238.1226, found: 238.1228.

HPLC analysis: 98:2 er (IB column, 25 °C, n-hexane / i-PrOH = 96 / 4, 0.4 mL / min, λ = 254 nm), Rt (major) = 31.4 min, Rt (minor) = 40.0 min.

(*S*)-6'-hydroxy-2',3',4',6-tetramethyl-[1,1'-biphenyl]-2-carbonitrile (3c)

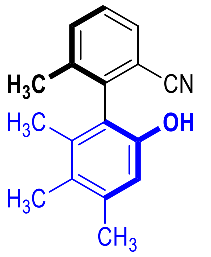

White solid, 99% yield, 24.9 mg; m.p. 223-224 °C.

[α]$^{25}_{D}$ = +23.4 (*c* = 0.5 in MeOH).

^{1}H NMR (400 MHz, $CDCl_3$) δ 7.62 (d, *J* = 7.7 Hz, 1H), 7.54 (d, *J* = 7.7 Hz, 1H), 7.38 (t, *J* = 7.7 Hz, 1H), 6.64 (s, 1H), 4.33 (s, 1H), 2.29 (s, 3H), 2.15 (s, 3H), 2.10 (s, 3H), 1.89 (s, 3H).

^{13}C NMR (101 MHz, $CDCl_3$) δ 149.6, 140.9, 139.5, 138.2, 135.1, 134.5, 130.8, 128.2, 127.8, 121.6, 118.1, 114.7, 114.7, 20.9, 19.8, 16.9, 15.3.

HRMS (ESI, m/z) calcd. for $C_{17}H_{17}NOH^+$ $[M+H]^+$: 252.1383, found: 252.1387.

HPLC analysis: 92:8 er (IB column, 25 °C, *n*-hexane / *i*-PrOH = 96 / 4, 0.4 mL / min, λ = 254 nm), Rt (major) = 31.9 min, Rt (minor) = 38.6 min.

(*S*)-2'-hydroxy-3',4',6,6'-tetramethyl-[1,1'-biphenyl]-2-carbonitrile (3d)

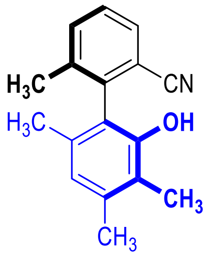

White solid, 96% yield, 24.1 mg; m.p. 116-117 °C.

[α]$^{25}_{D}$ = +33.3 (*c* = 0.5 in MeOH).

^{1}H NMR (400 MHz, $CDCl_3$) δ 7.64 (d, *J* = 7.7 Hz, 1H), 7.56 (d, *J* = 7.6 Hz, 1H), 7.40 (t, *J* = 7.7 Hz, 1H), 6.74 (s, 1H), 4.28 (s, 1H), 2.29 (s, 3H), 2.16 (s, 3H), 2.10 (s, 3H), 1.91 (s, 3H).

^{13}C NMR (101 MHz, $CDCl_3$) δ 149.9, 139.8, 139.6, 138.1, 134.6, 133.0, 131.0, 128.4, 123.9, 120.8, 119.8, 117.7, 114.7, 20.0, 19.6, 19.1, 11.5.

HRMS (ESI, m/z) calcd. for $C_{17}H_{17}NOH^+$ $[M+H]^+$: 252.1383, found: 252.1386.

UPLC analysis: 96:4 er (IB-U column, 25 °C, *n*-hexane / *i*-PrOH = 96 / 4, 0.3 mL / min, λ = 254 nm), Rt (major) = 9.4 min, Rt (minor) = 5.3 min.

(*R*)-2'-hydroxy-4',6'-dimethoxy-6-methyl-[1,1'-biphenyl]-2-carbonitrile (3e)

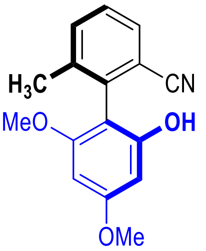

White solid, 78% yield, 20.1 mg; m.p. 90-91 °C.

[α]$^{25}_{D}$ = +22.8 (*c* = 0.5 in MeOH).

^{1}H NMR (400 MHz, Acetone-d_6) δ 8.36 (s, 1H), 7.57 (d, *J* = 7.7 Hz, 1H), 7.53 (d, *J* = 7.6 Hz, 1H), 7.36 (t, *J* = 7.7 Hz, 1H), 6.25 (d, *J* = 1.4 Hz, 2H), 3.80 (s, 3H), 3.71 (s, 3H), 2.13 (s, 3H).

^{13}C NMR (101 MHz, Acetone-d_6) δ 161.8, 158.6, 155.8, 139.9, 138.9, 133.6, 129.8, 127.4, 118.5, 115.4, 106.4, 93.9, 90.3, 55.1, 54.6, 19.1.

HRMS (ESI, m/z) calcd. for $C_{16}H_{15}NO_3H^+$ $[M+H]^+$: 270.1125, found: 270.1127.

HPLC analysis: 95:5 er (IB column, 25 °C, *n*-hexane / *i*-PrOH = 96 / 4, 0.4 mL / min, λ = 254 nm), Rt (major) = 30.2 min, Rt (minor) = 28.3 min.

(*S*)-3'-chloro-6'-hydroxy-2',4',6-trimethyl-[1,1'-biphenyl]-2-carbonitrile (3f)

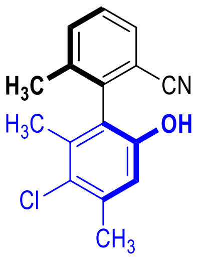

White solid, 98% yield, 26.8 mg; m.p. 160-161 °C.

$[\alpha]^{25}_D$ = +18.0 (*c* = 0.4 in MeOH).

^{1}H NMR (400 MHz, $CDCl_3$) δ 7.64 (d, *J* = 7.7 Hz, 1H), 7.57 (d, *J* = 7.7 Hz, 1H), 7.42 (t, *J* = 7.7 Hz, 1H), 6.74 (s, 1H), 4.57 (s, 1H), 2.39 (s, 3H), 2.10 (s, 3H), 2.02 (s, 3H).

^{13}C NMR (101 MHz, $CDCl_3$) δ 150.4, 139.4, 139.4, 138.0, 135.1, 134.8, 131.0, 128.7, 127.1, 122.8, 117.7, 115.7, 114.5, 21.1, 19.7, 17.7.

HRMS (ESI, m/z) calcd. for $C_{16}H_{14}ClNOH^+$ $[M+H]^+$: 272.0837, found: 272.0845.

UPLC analysis: 86:14 er (IB-U column, 25 °C, *n*-hexane / *i*-PrOH = 97 / 3, 0.4 mL / min, λ = 254 nm), Rt (major) = 8.5 min, Rt (minor) = 6.5 min.

(*S*)-2'-hydroxy-3',6,6'-trimethyl-[1,1'-biphenyl]-2-carbonitrile (3g)

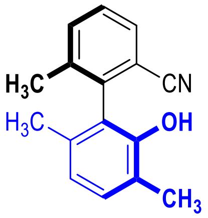

White solid, 97% yield, 23.0 mg; m.p. 110-111 °C.

$[\alpha]^{25}_D$ = +28.6 (*c* = 0.5 in MeOH).

^{1}H NMR (400 MHz, $CDCl_3$) δ 7.64 (d, *J* = 7.7 Hz, 1H), 7.56 (d, *J* = 7.7 Hz, 1H), 7.40 (t, *J* = 7.7 Hz, 1H), 7.10 (d, *J* = 7.7 Hz, 1H), 6.82 (d, *J* = 7.7 Hz, 1H), 4.39 (s, 1H), 2.25 (s, 3H), 2.09 (s, 3H), 1.94 (s, 3H).

^{13}C NMR (101 MHz, $CDCl_3$) δ 150.4, 139.9, 139.5, 134.7, 134.4, 131.1, 128.6, 123.4, 122.2, 121.3, 117.8, 114.5, 19.6, 19.4, 15.8.

HRMS (ESI, m/z) calcd. for $C_{16}H_{15}NOH^+$ $[M+H]^+$: 238.1226, found: 238.1227.

UPLC analysis: 95:5 er (IB-U column, 25 °C, *n*-hexane / *i*-PrOH = 95 / 5, 0.5 mL / min, λ = 254 nm), Rt (major) = 9.2 min, Rt (minor) = 4.2 min.

(*S*)-2'-hydroxy-3'-methoxy-6,6'-dimethyl-[1,1'-biphenyl]-2-carbonitrile (3h)

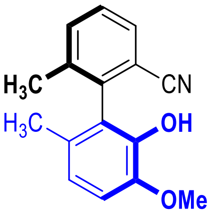

White solid, 85% yield, 21.6 mg; m.p. 83-84 ^{o}C.

[α]$^{25}_{D}$ = +38.4 (*c* = 0.5 in MeOH).

^{1}H NMR (400 MHz, $CDCl_3$) δ 7.60 (d, *J* = 7.6 Hz, 1H), 7.52 (d, *J* = 7.7 Hz, 1H), 7.36 (t, *J* = 7.7 Hz, 1H), 6.83 (q, *J* = 8.3 Hz, 2H), 5.62 (s, 1H), 3.91 (s, 3H), 2.10 (s, 3H), 1.94 (s, 3H).

^{13}C NMR (101 MHz, $CDCl_3$) δ 144.8, 142.8, 140.9, 138.8, 134.4, 130.6, 129.0, 128.1, 123.7, 121.2, 118.4, 113.9, 110.7, 56.2, 19.8, 19.1.

HRMS (ESI, m/z) calcd. for $C_{16}H_{15}NO_2H^+$ [M+H]$^+$: 254.1176, found: 254.1177;

HPLC analysis: 90:10 er (AD-H column, 25 ^{o}C, *n*-hexane / *i*-PrOH = 95 / 5, 0.4 mL / min, *λ* = 254 nm), Rt (major) = 40.5 min, Rt (minor) = 49.9 min.

(*S*)-2'-hydroxy-3'-isopropyl-6,6'-dimethyl-[1,1'-biphenyl]-2-carbonitrile (3i)

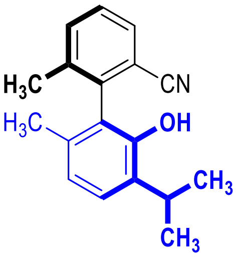

White solid, 94% yield, 25.0 mg; m.p. 74-75 ^{o}C.

[α]$^{25}_{D}$ = +49.0 (*c* = 0.5 in MeOH).

^{1}H NMR (400 MHz, $CDCl_3$) δ 7.68 (d, *J* = 7.7 Hz, 1H), 7.60 (d, *J* = 7.7 Hz, 1H), 7.44 (t, *J* = 7.7 Hz, 1H), 7.20 (d, *J* = 7.9 Hz, 1H), 6.91 (d, *J* = 7.9 Hz, 1H), 4.34 (s, 1H), 3.17 – 3.27 (m, 1H), 2.12 (s, 3H), 1.96 (s, 3H), 1.31 – 1.26 (m, 6H).

^{13}C NMR (101 MHz, $CDCl_3$) δ 149.3, 139.7, 139.7, 134.8, 133.9, 132.1, 131.2, 128.7, 126.4, 123.4, 122.4, 117.6, 114.8, 27.1, 22.7, 22.5, 19.6, 19.3.

HRMS (ESI, m/z) calcd. for $C_{18}H_{19}NOH^+$ [M+H]$^+$: 266.1540, found: 266.1541.

UPLC analysis: 96:4 er (IB-U column, 25 ^{o}C, *n*-hexane / *i*-PrOH = 97 / 3, 0.3 mL / min, *λ* = 254 nm), Rt (major) = 6.8 min, Rt (minor) = 4.4 min.

(*S*)-3'-chloro-2'-hydroxy-6,6'-dimethyl-[1,1'-biphenyl]-2-carbonitrile (3j)

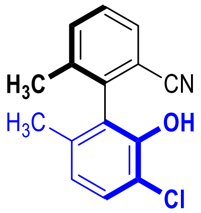

White solid, 77% yield, 19.9 mg; m.p. 223-224 °C.

[α]$^{25}_{D}$ = +54.1 (*c* = 0.5 in MeOH).

^{1}H NMR (400 MHz, $CDCl_3$) δ 7.61 (d, *J* = 7.7 Hz, 1H), 7.54 (d, *J* = 7.7 Hz, 1H), 7.39 (t, *J* = 7.7 Hz, 1H), 7.30 (d, *J* = 8.3 Hz, 1H), 6.87 (d, *J* = 8.3 Hz, 1H), 5.51 (s, 1H), 2.09 (s, 3H), 1.97 (s, 3H).

^{13}C NMR (101 MHz, $CDCl_3$) δ 148.3, 140.0, 138.6, 136.8, 134.5, 130.6, 128.9, 128.3, 125.1, 123.0, 118.0, 117.7, 113.6, 19.7, 19.3.

HRMS (ESI, m/z) calcd. for $C_{15}H_{12}ClNOH^+$ $[M+H]^+$: 258.0680, found: 258.0681.

HPLC analysis: 86:14 er (IB column, 25 °C, *n*-hexane / *i*-PrOH = 96 / 4, 0.4 mL / min, λ = 254 nm), Rt (major) = 40.2 min, Rt (minor) = 27.9 min.

(*R*)-2'-hydroxy-6'-methoxy-6-methyl-[1,1'-biphenyl]-2-carbonitrile (3k)

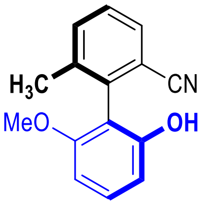

White solid, 83% yield, 19.9 mg; m.p. 223-224 °C.

[α]$^{25}_{D}$ = +64.3 (*c* = 0.5 in MeOH).

^{1}H NMR (400 MHz, Acetone-d_6) δ 8.41 (s, 1H), 7.60 (dd, *J* = 7.7, 0.6 Hz, 1H), 7.57 – 7.53 (m, 1H), 7.38 (t, *J* = 7.7 Hz, 1H), 7.25 (t, *J* = 8.3 Hz, 1H), 6.69 – 6.62 (m, 2H), 3.71 (s, 3H), 2.12 (s, 3H).

^{13}C NMR (101 MHz, Acetone-d_6) δ 157.9, 155.2, 139.4, 138.9, 133.7, 130.1, 129.9, 127.6, 118.4, 114.8, 113.6, 108.7, 102.6, 55.2, 19.1.

HRMS (ESI, m/z) calcd. for $C_{15}H_{13}NO_2H^+$ $[M+H]^+$: 240.1020, found: 240.1020.

UPLC analysis: 93:7 er (IB column, 25 °C, *n*-hexane / *i*-PrOH = 95 / 5, 0.5 mL / min, λ = 254 nm), Rt (major) = 4.7 min, Rt (minor) = 10.8 min.

(*R*)-2'-chloro-6'-hydroxy-6-methyl-[1,1'-biphenyl]-2-carbonitrile (3l)

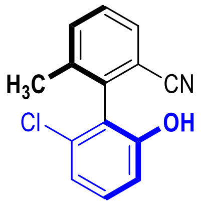

White solid, 89% yield, 21.6 mg; m.p. 175-176 °C.

[α]$^{25}_{D}$ = +38.5 (*c* = 0.5 in MeOH).

^{1}H NMR (400 MHz, $CDCl_3$) δ 7.64 (d, *J* = 7.7 Hz, 1H), 7.57 (d, *J* = 7.6 Hz, 1H), 7.43 (t, *J* = 7.7 Hz, 1H), 7.25 (dd, *J* = 10.7, 5.6 Hz, 1H),

7.13 (d, J = 0.9 Hz, 1H), 6.84 (dd, J = 8.2, 0.9 Hz, 1H), 5.12 (s, 1H), 2.15 (s, 3H).

^{13}C NMR (101 MHz, $CDCl_3$) δ 154.0, 139.5, 138.0, 134.7, 134.0, 130.7, 130.7, 128.9, 123.5, 121.8, 117.8, 114.5, 114.1, 19.6.

HRMS (ESI, m/z) calcd. for $C_{14}H_{10}ClNOH^+$ $[M+H]^+$: 244.0524, found: 244.0524.

UPLC analysis: 95:5 er (IB column, 25 °C, *n*-hexane / *i*-PrOH = 95 / 5, 0.5 mL / min, λ = 254 nm), Rt (major) = 3.4 min, Rt (minor) = 4.7 min.

(*S*)-2'-hydroxy-4',5,6,6'-tetramethyl-[1,1'-biphenyl]-2-carbonitrile (3m)

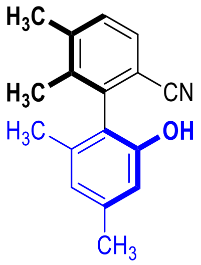

White solid, 82% yield, 20.7 mg; m.p. 167-168 °C.

$[\alpha]^{25}_D$ = +2.7 (c = 0.5 in MeOH).

^{1}H NMR (400 MHz, $CDCl_3$) δ 7.51 (d, J = 7.9 Hz, 1H), 7.26 (d, J = 7.9 Hz, 1H), 6.72 (s, 1H), 6.59 (s, 1H), 4.77 (s, 1H), 2.39 (s, 3H), 2.30 (s, 3H), 2.00 (s, 3H), 1.93 (s, 3H).

^{13}C NMR (101 MHz, $CDCl_3$) δ 152.4, 143.1, 139.7, 139.6, 138.0, 137.0, 130.5, 129.9, 123.5, 121.6, 118.4, 113.9, 111.9, 21.3, 21.2, 19.6, 16.2.

HRMS (ESI, m/z) calcd. for $C_{17}H_{17}NOH^+$ $[M+H]^+$: 252.1382, found: 252.1388.

HPLC analysis: 97:3 er (AD-H column, 25 °C, *n*-hexane / *i*-PrOH = 95 / 5, 0.4 mL / min, λ = 254 nm), Rt (major) = 37.3 min, Rt (minor) = 27.9 min.

(*S*)-2'-hydroxy-4,4',6,6'-tetramethyl-[1,1'-biphenyl]-2-carbonitrile (3n)

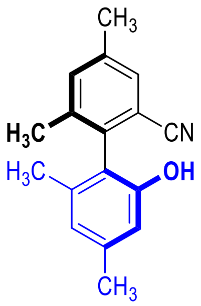

White solid, 95% yield, 23.8 mg; m.p. 76-77 °C.

$[\alpha]^{25}_D$ = +3.1 (c = 0.4 in MeOH).

^{1}H NMR (400 MHz, $CDCl_3$) δ 7.43 (s, 1H), 7.36 (s, 1H), 6.73 (s, 1H), 6.61 (s, 1H), 4.49 (s, 1H), 2.40 (s, 3H), 2.31 (s, 3H), 2.07 (s, 3H), 1.94 (s, 3H).

^{13}C NMR (101 MHz, $CDCl_3$) δ 152.3, 139.7, 139.2, 138.5, 137.1, 136.7, 135.6, 131.3, 123.6, 120.8, 118.1, 114.4, 113.8, 21.3, 20.9, 19.6, 19.5.

HRMS (ESI, m/z) calcd. for $C_{17}H_{17}NOH^+$ $[M+H]^+$: 252.1382, found: 252.1387.

HPLC analysis: 97:3 er (IB column, 25 °C, *n*-hexane / *i*-PrOH = 96 / 4, 0.4 mL / min, λ = 254 nm), Rt (major) = 27.6 min, Rt (minor) = 46.2 min.

(*S*)-4-chloro-2'-hydroxy-4',6,6'-trimethyl-[1,1'-biphenyl]-2-carbonitrile (3o)

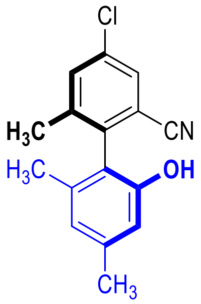

White solid, 62% yield, 16.6 mg; m.p. 70-71 ^{o}C.

[α]$^{25}_{D}$ = +10.3 (*c* = 0.4 in MeOH).

^{1}H NMR (400 MHz, $CDCl_3$) δ 7.59 (d, *J* = 1.9 Hz, 1H), 7.53 (d, *J* = 1.6 Hz, 1H), 6.74 (s, 1H), 6.59 (s, 1H), 4.53 (s, 1H), 2.32 (s, 3H), 2.10 (s, 3H), 1.95 (s, 3H).

^{13}C NMR (101 MHz, $CDCl_3$) δ 152.1, 141.3, 140.1, 139.0, 137.1, 134.5, 133.9, 130.2, 123.8, 120.0, 116.8, 115.8, 114.0, 21.3, 19.7, 19.4.

HRMS (ESI, m/z) calcd. for $C_{16}H_{14}ClNOH^{+}$ $[M+H]^{+}$: 272.0837, found: 272.0840.

HPLC analysis: 94:6 er (IB column, 25 ^{o}C, *n*-hexane / *i*-PrOH = 96 / 4, 0.4 mL / min, *λ* = 254 nm), Rt (major) = 21.1 min, Rt (minor) = 23.0 min.

(*R*)-2'-hydroxy-6-methoxy-4',6'-dimethyl-[1,1'-biphenyl]-2-carbonitrile (3p)

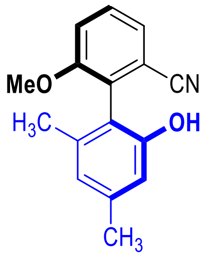

White solid, 97% yield, 24.7 mg; m.p. 138-139 ^{o}C.

[α]$^{25}_{D}$ = +33.4 (*c* = 0.5 in $CHCl_3$).

^{1}H NMR (400 MHz, $CDCl_3$) δ 7.42 (t, *J* = 8.0 Hz, 1H), 7.35 (dd, *J* = 7.7, 0.9 Hz, 1H), 7.20 (d, *J* = 8.3 Hz, 1H), 6.72 (s, 1H), 6.55 (s, 1H), 4.96 (s, 1H), 3.76 (s, 3H), 2.28 (s, 3H), 1.99 (s, 3H).

^{13}C NMR (101 MHz, $CDCl_3$) δ 157.7, 152.8, 139.8, 137.9, 129.8, 129.6, 125.0, 123.5, 118.3, 117.8, 115.6, 115.6, 114.1, 56.1, 21.4, 19.6.

HRMS (ESI, m/z) calcd. for $C_{16}H_{15}NO_2H^{+}$ $[M+H]^{+}$: 254.1175, found: 254.1174.

UPLC analysis: 98:2 er (IB-U column, 25 ^{o}C, *n*-hexane / *i*-PrOH = 70 / 30, 0.8 mL / min, *λ* = 254 nm), Rt (major) = 8.9 min, Rt (minor) = 6.7 min.

(*S*)-6-ethyl-2'-hydroxy-6'-methyl-[1,1'-biphenyl]-2-carbonitrile (3q)

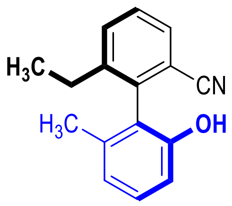

White solid, 82% yield, 19.4 mg; m.p. 82-83 ^{o}C.

[α]$^{25}_{D}$ = -14.7 (*c* = 0.5 in MeOH).

^{1}H NMR (400 MHz, $CDCl_3$) δ 7.64 (dd, *J* = 13.2, 7.7 Hz, 2H), 7.47 (t, *J* = 7.8 Hz, 1H), 7.24 (t, *J* = 7.9 Hz, 1H), 6.94 (d, *J* = 7.6

Hz, 1H), 6.79 (d, J = 8.1 Hz, 1H), 4.83 (s, 1H), 2.44 (q, J = 7.6 Hz, 2H), 2.01 (s, 3H), 1.10 (t, J = 7.6 Hz, 3H).

^{13}C NMR (101 MHz, $CDCl_3$) δ 152.6, 145.1, 139.3, 137.5, 133.1, 130.9, 129.7, 128.7, 123.7, 122.6, 117.9, 114.4, 113.3, 26.2, 19.7, 14.4.

HRMS (ESI, m/z) calcd. for $C_{16}H_{15}NOH^+$ $[M+H]^+$: 238.1226, found: 238.1227.

HPLC analysis: 93:7 er (IB column, 25 ^oC, *n*-hexane / *i*-PrOH = 96 / 4, 0.4 mL / min, λ = 254 nm), Rt (major) = 27.3 min, Rt (minor) = 34.0 min.

(*R*)-6-ethyl-2'-hydroxy-6'-methoxy-[1,1'-biphenyl]-2-carbonitrile (3r)

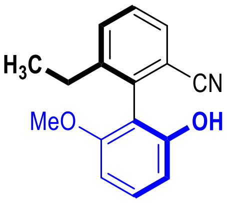

White solid, 91% yield, 22.9 mg; m.p. 124-125 ^oC.

$[\alpha]^{25}_D$ = +3.9 (c = 0.5 in MeOH).

^{1}H NMR (400 MHz, Acetone-d_6) δ 8.39 (s, 1H), 7.63 – 7.57 (m, 2H), 7.42 (t, J = 7.7 Hz, 1H), 7.25 (t, J = 8.3 Hz, 1H), 6.68 – 6.62 (m, 2H), 3.71 (s, 3H), 2.46 (q, J = 7.6 Hz, 2H), 1.03 (t, J = 7.6 Hz, 3H).

^{13}C NMR (101 MHz, Acetone-d_6) δ 158.1, 155.4, 145.2, 138.4, 132.3, 130.1, 129.9, 127.9, 118.3, 114.9, 113.5, 108.6, 102.5, 55.2, 26.3, 14.0.

HRMS (ESI, m/z) calcd. for $C_{16}H_{15}NO_2H^+$ $[M+H]^+$: 254.1175, found: 254.1174.

HPLC analysis: 93:7 er (AD-H column, 25 ^oC, *n*-hexane / *i*-PrOH = 95 / 5, 0.4 mL / min, λ = 254 nm), Rt (major) = 36.9 min, Rt (minor) = 38.8 min.

(*S*)-2'-hydroxy-4,6,6'-trimethyl-[1,1'-biphenyl]-2-carbonitrile (3s)

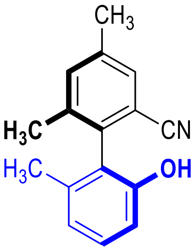

White solid, 99% yield, 22.1 mg; m.p. 130-131 ^oC.

$[\alpha]^{25}_D$ = -2.3 (c = 0.5 in MeOH).

^{1}H NMR (400 MHz, $CDCl_3$) δ 7.45 (s, 1H), 7.38 (s, 1H), 7.21 (t, J = 7.9 Hz, 1H), 6.91 (d, J = 7.6 Hz, 1H), 6.79 (d, J = 8.1 Hz, 1H), 4.56 (s, 1H), 2.41 (s, 3H), 2.07 (s, 3H), 1.99 (s, 3H).

^{13}C NMR (101 MHz, $CDCl_3$) δ 152.5, 139.0, 138.7, 137.5, 136.5, 135.7, 131.4, 129.6, 123.7, 122.6, 117.9, 114.1, 113.1, 20.9, 19.6, 19.6.

HRMS (ESI, m/z) calcd. for $C_{16}H_{15}NOH^+$ $[M+H]^+$: 238.1226, found: 238.1228.

HPLC analysis: 97:3 er (AD-H column, 25 °C, *n*-hexane / *i*-PrOH = 95 / 5, 0.4 mL / min, λ = 254 nm), Rt (major) = 56.2 min, Rt (minor) = 60.9 min.

(*R*)-2'-hydroxy-6'-methoxy-4,6-dimethyl-[1,1'-biphenyl]-2-carbonitrile (3t)

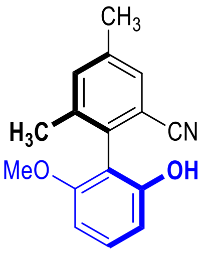

White solid, 90% yield, 22.7 mg; m.p. 100-101 °C.

$[\alpha]^{25}_{D}$ = -3.6 (*c* = 0.5 in MeOH).

^{1}H NMR (400 MHz, Acetone-d_6) δ 8.33 (s, 1H), 7.40 (s, 1H), 7.36 (d, *J* = 0.6 Hz, 1H), 7.23 (t, *J* = 8.3 Hz, 1H), 6.67 – 6.61 (m, 2H), 3.71 (s, 3H), 2.36 (s, 3H), 2.07 (s, 3H).

^{13}C NMR (101 MHz, Acetone-d_6) δ 158.1, 155.4, 139.1, 137.5, 135.9, 134.6, 130.1, 129.9, 118.5, 114.6, 113.5, 108.6, 102.5, 55.2, 19.8, 19.0.

HRMS (ESI, m/z) calcd. for $C_{16}H_{15}NO_2H^+$ $[M+H]^+$: 254.1175, found: 254.1178.

HPLC analysis: 93:7 er (AD-H column, 25 °C, *n*-hexane / *i*-PrOH = 95 / 5, 0.4 mL / min, λ = 254 nm), Rt (major) = 39.4 min, Rt (minor) = 42.8 min.

(*S*)-1-(2-hydroxy-4,6-dimethylphenyl)-2-naphthonitrile (3u)

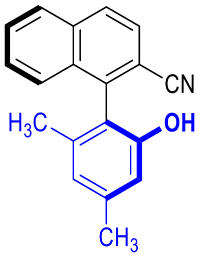

White solid, 98% yield, 27.1 mg; m.p. 163-164 °C.

$[\alpha]^{25}_{D}$ = +97.4 (*c* = 0.5 in MeOH).

^{1}H NMR (400 MHz, $CDCl_3$) δ 7.96 (dd, *J* = 8.3, 5.3 Hz, 2H), 7.74 (d, *J* = 8.6 Hz, 1H), 7.62 – 7.66 (m, 1H), 7.60 – 7.47 (m, 2H), 6.82 (s, 1H), 6.70 (s, 1H), 4.45 (d, *J* = 4.1 Hz, 1H), 2.38 (s, 3H), 1.90 (s, 3H).

^{13}C NMR (101 MHz, $CDCl_3$) δ 151.3, 141.5, 135.7, 135.2, 131.9, 131.7, 129.4, 129.3, 128.7, 128.4, 127.2, 126.7, 122.4, 122.3, 121.7, 118.3, 111.8, 19.7, 16.0.

HRMS (ESI, m/z) calcd. for $C_{19}H_{15}NOH^+$ $[M+H]^+$: 274.1226, found: 274.1233.

UPLC analysis: 93:7 er (IB-U column, 25 °C, *n*-hexane / *i*-PrOH = 96 / 4, 0.4 mL / min, λ = 254 nm), Rt (major) = 13.4 min, Rt (minor) = 10.3 min.

(*R*)-1-(2-hydroxy-4,6-dimethoxyphenyl)-2-naphthonitrile (3v)

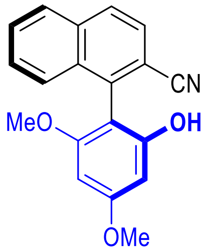

White solid, 97% yield, 30.0 mg; m.p. 166-167 ^{o}C.

[α]$^{25}_{D}$ = +18.5 (*c* = 0.5 in MeOH).

^{1}H NMR (400 MHz, Acetone-d_6) δ 8.39 (s, 1H), 8.01 (dd, *J* = 8.7, 1.8 Hz, 2H), 7.74 (d, *J* = 8.5 Hz, 1H), 7.66 – 7.61 (m, 2H), 7.56 – 7.50 (m, 1H), 6.34 (dd, *J* = 5.2, 2.2 Hz, 2H), 3.85 (s, 3H), 3.65 (s, 3H).

^{13}C NMR (101 MHz, Acetone-d_6) δ 162.3, 159.3, 156.5, 140.4, 135.0, 132.7, 128.3, 128.2, 128.2, 127.1, 127.1, 126.7, 118.7, 112.3, 105.0, 94.0, 90.5, 55.2, 54.7.

HRMS (ESI, m/z) calcd. for $C_{19}H_{15}NO_3H^+$ [M+H]$^+$: 306.1125, found: 306.1127.

HPLC analysis: 95:5 er (IB column, 25 ^{o}C, *n*-hexane / *i*-PrOH = 90 / 10, 0.5 mL / min, *λ* = 254 nm), Rt (major) = 42.4 min, Rt (minor) = 40.1 min.

(*S*)-1-(2-hydroxy-3,4,6-trimethylphenyl)-2-naphthonitrile (3w)

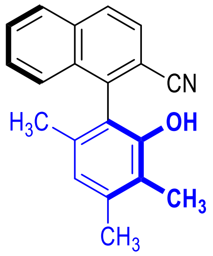

White solid, 98% yield, 28.0 mg; m.p. 177-178 ^{o}C.

[α]$^{25}_{D}$ = -21.6 (*c* = 0.5 in MeOH).

^{1}H NMR (400 MHz, $CDCl_3$) δ 7.96 (t, *J* = 7.9 Hz, 2H), 7.74 (d, *J* = 8.6 Hz, 1H), 7.63 – 7.67 (m, 1H), 7.49 – 7.57 (m, 2H), 6.82 (s, 1H), 4.24 (s, 1H), 2.34 (s, 3H), 2.19 (s, 3H), 1.87 (s, 3H).

^{13}C NMR (101 MHz, $CDCl_3$) δ 150.8, 141.5, 138.8, 135.1, 134.2, 131.9, 129.2, 129.2, 128.6, 128.2, 127.1, 126.6, 124.2, 120.1, 119.8, 118.2, 111.9, 20.3, 19.4, 11.7.

HRMS (ESI, m/z) calcd. for $C_{20}H_{17}NOH^+$ [M+H]$^+$: 288.1383, found: 288.1382.

HPLC analysis: 96:4 er (IB column, 25 ^{o}C, *n*-hexane / *i*-PrOH = 96 / 4, 0.4 mL / min, *λ* = 254 nm), Rt (major) = 62.0 min, Rt (minor) = 57.0 min.

(S)-1-(2-hydroxy-3-isopropyl-6-methylphenyl)-2-naphthonitrile (3x)

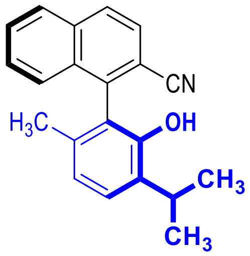

White solid, 94% yield, 28.5 mg; m.p. 138-139 °C.

$[\alpha]^{25}_{D}$ = +17.3 (c = 0.4 in MeOH).

^{1}H NMR (400 MHz, $CDCl_3$) δ 8.01 (t, J = 8.9 Hz, 2H), 7.79 (d, J = 8.5 Hz, 1H), 7.67 – 7.71 (m, 1H), 7.56 (dd, J = 4.6, 1.8 Hz, 2H), 7.31 (d, J = 7.9 Hz, 1H), 6.99 (d, J = 7.9 Hz, 1H), 4.34 (s, 1H), 3.19 – 3.30 (m, 1H), 1.92 (s, 3H), 1.34 (d, J = 1.3 Hz, 3H), 1.32 (d, J = 1.3 Hz, 3H).

^{13}C NMR (101 MHz, $CDCl_3$) δ 150.1, 141.2, 135.1, 135.0, 132.3, 131.7, 129.3, 129.2, 128.6, 128.3, 127.1, 126.8, 126.5, 122.5, 122.2, 118.0, 111.9, 27.1, 22.7, 22.7, 19.5.

HRMS (ESI, m/z) calcd. for $C_{21}H_{19}NOH^+$ $[M+H]^+$: 302.1540, found: 302.1538.

HPLC analysis: 90:10 er (AD-H column, 25 °C, n-hexane / i-PrOH = 96 / 4, 0.4 mL / min, λ = 254 nm), Rt (major) = 34.6 min, Rt (minor) = 53.3 min.

(S)-1-(2-hydroxy-3,6-dimethylphenyl)-2-naphthonitrile (3y)

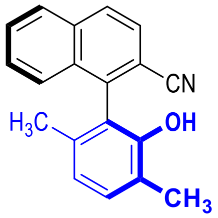

White solid, 98% yield, 26.8 mg; m.p. 134-135 °C.

$[\alpha]^{25}_{D}$ = +24.7 (c = 0.5 in MeOH).

^{1}H NMR (400 MHz, $CDCl_3$) δ 7.97 (t, J = 8.4 Hz, 2H), 7.75 (d, J = 8.6 Hz, 1H), 7.63 – 7.67 (m, 1H), 7.52 (dd, J = 4.8, 1.8 Hz, 2H), 7.19 (d, J = 7.7 Hz, 1H), 6.90 (d, J = 7.7 Hz, 1H), 4.34 (d, J = 2.4 Hz, 1H), 2.29 (s, 3H), 1.89 (s, 3H).

^{13}C NMR (101 MHz, $CDCl_3$) δ 151.3, 141.5, 135.7, 135.2, 131.9, 131.7, 129.4, 129.3, 128.7, 128.4, 127.2, 126.7, 122.4, 122.3, 121.7, 118.3, 111.8, 19.7, 16.0.

HRMS (ESI, m/z) calcd. for $C_{19}H_{15}NOH^+$ $[M+H]^+$: 274.1226, found: 274.1232.

HPLC analysis: 86:14 er (IB column, 25 °C, n-hexane / i-PrOH = 96 / 4, 0.4 mL / min, λ = 254 nm), Rt (major) = 31.6 min, Rt (minor) = 28.0 min.

(*S*)-2'-cyano-6,6'-dimethyl-[1,1'-biphenyl]-2-yl trifluoromethanesulfonate (5)

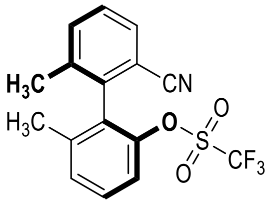

Colourless oil, 90% yield, 213.6 mg.

[α]$^{25}_{D}$ = +12.8 (*c* = 0.5 in $CHCl_3$).

^{1}H NMR (400 MHz, $CDCl_3$) δ 7.63 (d, *J* = 7.7 Hz, 1H), 7.56 (d, *J* = 7.4 Hz, 1H), 7.44 (td, *J* = 7.8, 3.4 Hz, 2H), 7.39 (d, *J* = 7.2 Hz, 1H), 7.29 (d, *J* = 8.1 Hz, 1H), 2.11 (d, *J* = 4.2 Hz, 6H).

^{13}C NMR (101 MHz, $CDCl_3$) δ 146.9, 139.7, 138.8, 137.4, 134.7, 130.8, 130.7, 130.4, 130.3, 129.1, 119.1, 118.3 (q, *J* = 321.2 Hz), 117.5, 114.0, 19.7, 19.6.

^{19}F NMR (377 MHz, $CDCl_3$) δ -74.6.

HRMS (ESI, m/z) calcd. for $C_{16}H_{12}F_3NO_3SNa^+$ [M+Na] $^+$: 378.0382, found 378.0385.

HPLC analysis: 97:3 er (IE column, 25 °C, *n*-hexane / *i*-PrOH = 98 /2, 0.4 mL / min, *λ* = 254 nm), Rt (major) = 25.4 min, Rt (minor) = 24.1 min.

(*S*)-2'-(diphenylphosphanyl)-6,6'-dimethyl-[1,1'-biphenyl]-2-carbonitrile (6)

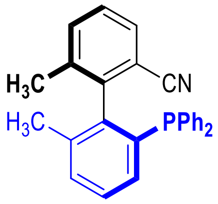

White solid, 73% yield, 170.7 mg; m.p. 129-130 °C.

[α]$^{25}_{D}$ = +21.6 (*c* = 0.5 in $CHCl_3$).

^{1}H NMR (400 MHz, $CDCl_3$) δ 7.47 (d, *J* = 7.6 Hz, 1H), 7.41 (d, *J* = 7.6 Hz, 1H), 7.35 – 7.26 (m, 9H), 7.18 – 7.22 (m, 4H), 7.03 – 7.06 (m, 1H), 1.97 (s, 3H), 1.81 (s, 3H).

^{13}C NMR (101 MHz, CDCl3) δ 143.8 (d, *J* = 7.3 Hz), 142.9 (d, *J* = 31.4 Hz), 138.4 (d, *J* = 2.2 Hz), 137.4 (d, *J* = 11.3 Hz), 136.4 (dd, *J* = 11.2, 7.8 Hz), 136.2 (d, *J* = 5.9 Hz), 134.3 – 133.5 (m), 134.2, 132.0, 131.2, 130.3, (d, *J* = 12.0 Hz), 128.7 (d, *J* = 12.0 Hz), 128.5 (d, *J* = 9.4 Hz), 128.5 – 128.3 (m), 127.9, 118.1, 114.2 (d, *J* = 3.0 Hz), 19.9 (d, *J* = 2.7 Hz), 19.8 (d, *J* = 3.7 Hz).

^{31}P NMR (162 MHz, $CDCl_3$) δ -13.94.

HRMS (ESI, m/z) calcd. for $C_{27}H_{22}NPH^+$ [M+H] $^+$: 392.1563, found 392.1562.

HPLC analysis: 97:3 er (ID column, 25 °C, *n*-hexane / *i*-PrOH = 90 / 10, 0.4 mL / min, *λ* = 254 nm), Rt (major) = 14.0 min, Rt (minor) = 18.5 min.

(*S*)-(2'-(diphenylphosphanyl)-6,6'-dimethyl-[1,1'-biphenyl]-2-yl)methanamine (7)

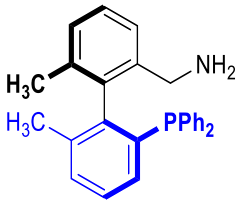

White solid, 80% yield, 81.0 mg; m.p. 96-97 °C.

[α]^{25}$_D$ = +71.9 (*c* = 0.5 in $CHCl_3$).

^{1}H NMR (400 MHz, $CDCl_3$) δ 7.4 – 7.3 (m, 6H), 7.3 – 7.2 (m, 6H), 7.1 (td, *J* = 7.6, 1.7 Hz, 2H), 7.1 – 7.0 (m, 2H), 3.4 (d, *J* = 14.8 Hz, 1H), 3.2 (d, *J* = 14.9 Hz, 1H), 2.9 (s, 2H), 1.9 (s, 3H), 1.6 (s, 3H).

^{13}C NMR (101 MHz, $CDCl_3$) δ 144.8 (d, *J* = 32.2 Hz), 138.8 (d, *J* = 1.6 Hz), 138.2 (d, *J* = 7.9 Hz), 137.0 (d, *J* = 9.5 Hz), 136.7, 136.5, 136.5, 134.1 (d, *J* = 7.2 Hz), 133.9 (d, *J* = 7.0 Hz), 131.8, 131.0, 128.7, 128.6 (d, *J* = 1.9 Hz), 128.6 – 128.1 (m), 127.6, 124.4, 43.5 (d, *J* = 2.8 Hz),20.0 (d, *J* = 2.7 Hz), 20.0 (d, *J* = 3.3 Hz).

^{31}P NMR (162 MHz, CDCl3) δ -14.41.

HRMS (ESI, m/z) calcd. for $C_{27}H_{26}NPH^+$ $[M+H]^+$: 396.1876, found 396.18730.

tert-butyl (*S*)-((2'-(diphenylphosphanyl)-6,6'-dimethyl-[1,1'-biphenyl]-2-yl)methyl)carbamate (8)

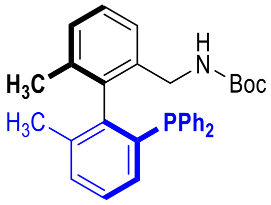

White solid, 80% yield, 170.7 mg.

[α]^{25}$_D$ = +93.3 (*c* = 0.2 in $CHCl_3$).

^{1}H NMR (400 MHz, $CDCl_3$) δ 7.36 – 7.25 (m, 8H), 7.21 (td, *J* = 7.5, 1.9 Hz, 3H), 7.18 – 7.09 (m, 4H), 7.07 – 7.00 (m, 1H), 3.96 – 4.03 (m, 1H), 3.33 – 3.23 (m, 1H), 1.90 (s, 3H), 1.71 (s, 3H), 1.41 (s, 9H).

^{13}C NMR (101 MHz, $CDCl_3$) δ 156.0, δ 144.6 (d, *J* = 32.0 Hz), 138.4 (d, *J* = 7.9 Hz), 136.9, 136.8, 136.7, 136.6 – 136.3 (m), 134.3 (d, *J* = 20.4 Hz), 133.9 (d, *J* = 20.3 Hz), 131.7, 131.0, 128.8 (d, *J* = 6.6 Hz), 128.5, 128.4 (dd, *J* = 18.2, 5.7 Hz), 79.0, 42.3, 28.5, 20.1 (d, *J* = 3.7 Hz), 19.9 (d, *J* = 2.5 Hz).

^{31}P NMR (162 MHz, CDCl3) δ -14.52.

HRMS (ESI, m/z) calcd. for $C_{32}H_{34}NO_2PNa^+$ $[M+Na]^+$: 518.2219, found 518.2213.

HPLC analysis: 97:3 er (AS-H column, 25 °C, *n*-hexane / *i*-PrOH = 98 / 2, 0.5 mL / min, *λ* = 254 nm), Rt (major) = 12.3 min, Rt (minor) = 10.2 min.

(*S*)-2'-hydroxy-6,6'-dimethyl-[1,1'-biphenyl]-2-carboxamide (9)

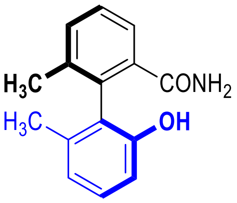

White solid, 93% yield, 47.8 mg; m.p. 129-130 °C.

[α]$^{25}_{D}$ = +37.3 (*c* = 0.4 in MeOH).

^{1}H NMR (400 MHz, Acetone-d_6) δ 8.09 (s, 1H), 7.48 (d, *J* = 7.6 Hz, 1H), 7.38 (d, *J* = 7.5 Hz, 1H), 7.31 (t, *J* = 7.6 Hz, 1H), 7.12 (t, *J* = 7.8 Hz, 1H), 6.82 (s, 1H), 6.80 (s, 1H), 6.47 (d, *J* = 30.0 Hz, 2H), 1.95 (s, 3H), 1.86 (s, 3H).

^{13}C NMR (101 MHz, Acetone-d_6) δ 172.2, 155.1, 138.6, 138.6, 138.4, 135.7, 132.2, 129.5, 128.3, 128.1, 126.4, 122.8, 114.8, 20.2, 20.1.

HRMS (ESI, m/z) calcd. for $C_{15}H_{15}NO_2Na^+$ [M+Na]$^+$: 264.0995, found: 264.0999.

UPLC analysis: 96:4 er (IC-U column, 25 °C, *n*-hexane / *i*-PrOH = 90 / 10, 0.5 mL / min, *λ* = 254 nm), Rt (major) = 8.0 min, Rt (minor) = 7.0 min.

(*S*)-2'-hydroxy-6,6'-dimethyl-[1,1'-biphenyl]-2-carbothioamide (10)

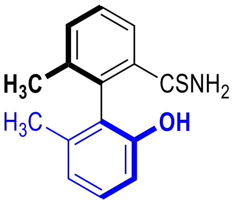

A yellow solid, 83% yield, 42.8 mg; m.p. 118-119 °C.

[α]$^{25}_{D}$ = -95.6 (*c* = 0.5 in MeOH).

^{1}H NMR (400 MHz, $CDCl_3$) δ 7.61 (dd, *J* = 7.1, 2.1 Hz, 1H), 7.42 (s, 1H), 7.37 – 7.30 (m, 2H), 7.16 (q, *J* = 7.8 Hz, 2H), 6.87 (dt, *J* = 7.7, 1.0 Hz, 1H), 6.76 (d, *J* = 8.1 Hz, 1H), 5.35 (s, 1H), 1.95 (d, *J* = 4.6 Hz, 6H).

^{13}C NMR (101 MHz, $CDCl_3$) δ 205.1, 152.2, 142.6, 138.1, 137.7, 131.8, 130.7, 129.1, 128.3, 126.7, 125.8, 123.3, 113.2, 19.9, 19.8.

HRMS (ESI, m/z) calcd. for $C_{15}H_{15}NOSH^+$ [M+H]$^+$: 258.0947, found: 258.0939.

UPLC analysis: 99:1 er (IA-U column, 25 °C, *n*-hexane / *i*-PrOH = 95 / 5, 0.4 mL / min, *λ* = 254 nm), Rt (major) = 4.9 min, Rt (minor) = 5.8 min.

(*S*)-2'-(aminomethyl)-6,6'-dimethyl-[1,1'-biphenyl]-2-ol (11)

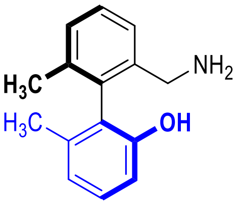

White solid, 71% yield, 72.1 mg; m.p. 161-162 °C.

[α]$^{25}_{D}$ = +107.0 (*c* = 0.5 in $CHCl_3$).

^{1}H NMR (400 MHz, $CDCl_3$) δ 7.24 (dd, *J* = 5.0, 2.5 Hz, 2H),

7.18 – 7.10 (m, 2H), 6.85 (d, *J* = 7.5 Hz, 1H), 6.73 (d, *J* = 8.0 Hz, 1H), 4.18 (s, 3H), 3.57 (d, *J* = 12.6 Hz, 1H), 3.41 (d, *J* = 12.4 Hz, 1H), 1.94 (s, 3H), 1.84 (s, 3H).

^{13}C NMR (101 MHz, $CDCl_3$) δ 154.2, 139.8, 138.0, 137.1, 136.6, 129.7, 128.5, 128.0, 127.9, 126.1, 122.1, 115.8, 45.0, 19.9, 19.8.

HRMS (ESI, m/z) calcd. for $C_{15}H_{17}NONa^+$ $[M+Na]^+$: 250.1202, found: 250.1199.

(*S*)-1-(3,5-bis(trifluoromethyl)phenyl)-3-((2'-hydroxy-6,6'-dimethyl-[1,1'-biphenyl]-2-yl)methyl)thiourea (12)

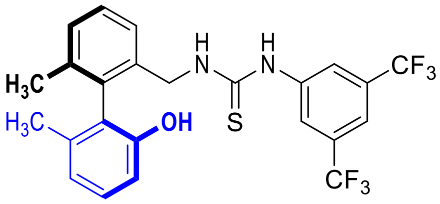

White solid, 90% yield, 79.0 mg; m.p. 67-68 °C.

$[\alpha]^{25}_D$ = -142.2 (*c* = 0.5 in $CHCl_3$).

^{1}H NMR (400 MHz, $CDCl_3$) δ 7.93 (s, 1H), 7.67 (d, *J* = 15.8 Hz, 3H), 7.43 (d, *J* = 7.1 Hz, 1H), 7.35 – 7.26 (m, 2H), 7.10 (t, *J* = 7.9 Hz, 1H), 6.82 (d, *J* = 7.6 Hz, 1H), 6.61 (d, *J* = 8.1 Hz, 1H), 6.41 (s, 1H), 4.70 (s, 2H), 4.17 (dd, *J* = 14.6, 4.7 Hz, 1H), 1.96 (s, 3H), 1.86 (s, 3H).

^{13}C NMR (101 MHz, $CDCl_3$) δ 180.3, 151.7, 138.8, 138.2, 137.3, 135.6, 134.2, 132.7 (q, *J* = 32.3 Hz), 130.5, 129.2, 128.9, 127.6, 125.2, 124.5, 123.0, 122.8 (q, *J* = 272.9 Hz), 119.5, 113.0, 47.5, 19.6, 19.6.

^{19}F NMR (377 MHz, $CDCl_3$) δ -63.01.

HRMS (ESI, m/z) calcd. for $C_{24}H_{20}F_6N_2OSNa^+$ $[M+Na]^+$: 521.1093, found: 521.1091.

HPLC analysis: 98:2 er (IB column, 25 °C, *n*-hexane / *i*-PrOH = 90 / 10, 0.5 mL / min, *λ* = 254 nm), Rt (major) = 37.2 min, Rt (minor) = 23.5 min.

(*S*)-1-(3,5-bis(trifluoromethyl)phenyl)-3-((2'-hydroxy-6,6'-dimethyl-[1,1'-biphenyl]-2-yl)methyl)urea (13)

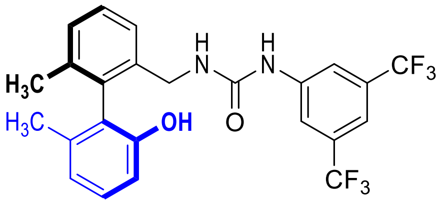

White solid, 66% yield, 56.0 mg; m.p. 178-179 °C.

$[\alpha]^{25}_D$ = -92.6 (*c* = 0.5 in $CHCl_3$);

^{1}H NMR (400 MHz, $CDCl_3$) δ 7.62 (s, 2H), 7.38 (s, 1H), 7.32 (s, 1H), 7.30 – 7.22 (m, 3H), 7.11 (t, *J* =

7.8 Hz, 1H), 6.85 (d, J = 7.5 Hz, 1H), 6.72 (d, J = 8.1 Hz, 1H), 5.55 (t, J = 5.7 Hz, 1H), 5.44 (s, 1H), 4.08 (dd, J = 15.0, 6.4 Hz, 1H), 3.91 (dd, J = 14.9, 4.9 Hz, 1H), 1.93 (s, 3H), 1.85 (s, 3H).

^{13}C NMR (101 MHz, $CDCl_3$) δ 155.0, 152.0, 140.2, 138.1, 137.3, 137.1, 134.0, 132.0 (q, J = 33.2 Hz), 130.0, 129.0, 128.7, 126.2, 125.5, 123.1 (q, J = 273.0 Hz), 122.9, 118.8, 116.0, 113.2, 42.8, 19.6, 19.5.

^{19}F NMR (377 MHz, $CDCl_3$) δ -63.32.

HRMS (ESI, m/z) calcd. for $C_{24}H_{20}F_6N_2O_2Na^+$ [M+Na] $^+$: 505.1321, found: 505.1325.

HPLC analysis: 98:2 er (IB column, 25 ^{o}C, *n*-hexane / *i*-PrOH = 85 / 15, 0.5 mL / min, λ = 254 nm), Rt (major) = 32.0 min, Rt (minor) = 17.3 min.

(*S*)-3-((3,5-bis(trifluoromethyl)phenyl)amino)-4-(((2'-hydroxy-6,6'-dimethyl-[1,1'-biphenyl]-2-yl)methyl)amino)cyclobut-3-ene-1,2-dione (14)

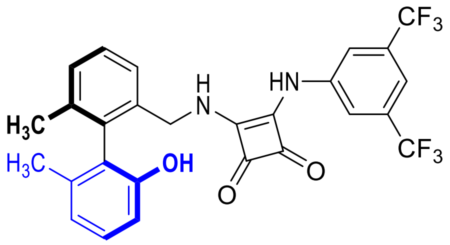

White solid, 95% yield, 51.0 mg; m.p. 138-139 ^{o}C.

$[\alpha]^{25}_D$ = -62.5 (*c* = 0.8 in $CHCl_3$).

^{1}H NMR (400 MHz, $CDCl_3$) δ 9.75 (s, 1H), 7.88 (s, 1H), 7.70 (s, 2H), 7.39 (s, 1H), 7.23 – 7.28 (m, 3H), 7.01 (t, J = 7.8 Hz, 1H), 6.71 (dd, J = 22.5, 7.8 Hz, 2H), 6.63 (s, 1H), 4.54 – 4.68 (m, 2H), 1.98 (s, 3H), 1.85 (s, 3H).

^{13}C NMR (101 MHz, $CDCl_3$) δ 183.4, 179.9, 169.3, 162.3, 153.1, 139.3, 138.8, 137.4, 135.5, 135.2, 132.6 (q, J = 33.5 Hz), 130.7, 128.8, 128.4, 126.8, 124.9, 124.3 – 121.4 (m), 122.8 (q, J = 272.9 Hz), 122.4, 116.9, 113.3, 48.1, 19.7, 19.5.

^{19}F NMR (377 MHz, $CDCl_3$) δ -63.34.

HRMS (ESI, m/z) calcd. for $C_{27}H_{20}F_6N_2O_3Na^+$ [M+Na] $^+$: 557.1270, found: 557.1273.

HPLC analysis: 96:4 er (IB column, 25 ^{o}C, *n*-hexane / *i*-PrOH = 90 / 10, 1.0 mL / min, λ = 254 nm), Rt (major) = 32.1 min, Rt (minor) = 26.4 min.

(*S*)-1,1,1-trifluoro-*N*-((2'-hydroxy-6,6'-dimethyl-[1,1'-biphenyl]-2-yl)methyl)methanesulfonamide (15)

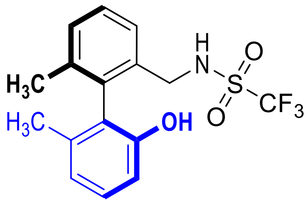

Colourless oil, 33% yield, 59.5 mg.

[α]$^{25}_{D}$ = -15.7 (*c* = 0.4 in $CHCl_3$).

^{1}H NMR (400 MHz, $CDCl_3$) δ 7.37 (s, 3H), 7.23 (d, *J* = 7.9 Hz, 1H), 6.95 (d, *J* = 6.7 Hz, 1H), 6.85 (d, *J* = 8.1 Hz, 1H), 5.25 (s, 1H), 4.72 (s, 1H), 4.16 (d, *J* = 13.6 Hz, 1H), 3.96 (d, *J* = 13.5 Hz, 1H), 2.01 (s, 3H), 1.90 (s, 3H).

^{13}C NMR (101 MHz, $CDCl_3$) δ 151.7, 138.4, 137.3, 134.9, 134.4, 131.1, 129.4, 129.1, 127.6, 124.7, 123.3, 119.6 (q, *J* = 321.2 Hz), 113.4, 47.0, 19.7, 19.6.

^{19}F NMR (377 MHz, $CDCl_3$) δ -77.31.

HRMS (ESI, m/z) calcd. for $C_{16}H_{16}F_3NO_3SNa^+$ [M+Na] $^+$: 382.0695, found: 382.0689.

HPLC analysis: 96:4 er (AD-H column, 25 °C, *n*-hexane / *i*-PrOH = 97 / 3, 0.4 mL / min, *λ* = 254 nm), Rt (major) = 27.7 min, Rt (minor) = 38.7 min.

(*S*,*E*)-*N'*-((2'-hydroxy-6,6'-dimethyl-[1,1'-biphenyl]-2-yl)methyl)-*N*,*N*-dimethylacetimidamide (16)

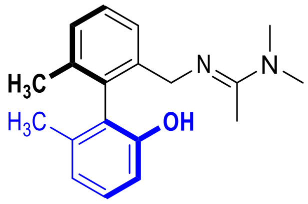

White solid, 64% yield, 83.5 mg; m.p. 170-171 °C.

[α]$^{25}_{D}$ = -241.6 (*c* = 0.5 in $CHCl_3$).

^{1}H NMR (400 MHz, $CDCl_3$) δ 8.69 (s, 1H), 7.35 (d, *J* = 7.5 Hz, 1H), 7.22 (dd, *J* = 18.5, 7.6 Hz, 2H), 7.09 (q, *J* = 7.5 Hz, 2H), 6.78 (d, *J* = 7.5 Hz, 1H), 4.32 (d, *J* = 9.7 Hz, 1H), 4.14 (d, *J* = 14.3 Hz, 1H), 3.04 (s, 6H), 1.93 (s, 3H), 1.85 (s, 3H), 1.67 (s, 3H).

^{13}C NMR (101 MHz, $CDCl_3$) δ 162.5, 154.6, 137.8, 137.3, 136.7, 133.5, 130.1, 128.8, 127.8, 127.7, 125.2, 121.5, 113.9, 47.8, 41.5, 40.6, 19.7, 19.6, 14.3.

HRMS (ESI, m/z) calcd. for $C_{19}H_{24}N_2OH^+$ [M+H] $^+$: 297.1961, found: 297.1970.

(*S*)-*N*-((2'-hydroxy-6,6'-dimethyl-[1,1'-biphenyl]-2-yl)methyl)acetamide (17)

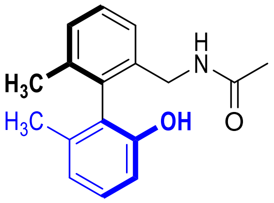

Colourless oil, 92% yield, 24.9 mg.

[α]$^{25}_{D}$ = -7.8 (*c* = 0.2 in CHCl3).

^{1}H NMR (400 MHz, $CDCl_3$) δ 7.25 (d, *J* = 2.3 Hz, 3H), 7.16 (t, *J* = 7.8 Hz, 1H), 6.91 – 6.79 (m, 2H), 6.29 (s, 1H), 6.10 (t, *J* = 5.5 Hz, 1H), 4.19 (dd, *J* = 14.4, 6.7 Hz, 1H), 3.84 (dd, *J* = 14.4, 4.4 Hz, 1H), 1.96 (s, 3H), 1.87 (s, 3H), 1.84 (s, 3H).

^{13}C NMR (101 MHz, $CDCl_3$) δ 170.2, 152.8, 137.9, 137.2, 137.0, 135.0, 129.8, 128.8, 128.4, 126.8, 125.7, 122.3, 113.4, 42.5, 23.0, 19.7, 19.7.

HRMS (ESI, m/z) calcd. for $C_{17}H_{19}NO_2Na^+$ $[M+Na]^+$: 292.1308, found: 292.1309.

HPLC analysis: 98:2 er (AD-H column, 25 °C, *n*-hexane / *i*-PrOH = 90 / 10, 1.0 mL / min, *λ* = 254 nm), Rt (major) = 7.0 min, Rt (minor) = 8.3 min.

(*S*)-2-(2-(aminomethyl)naphthalen-1-yl)-6-isopropyl-3-methylphenol (18)

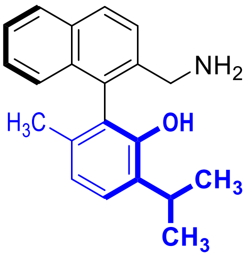

White solid, 63% yield, 154.9 mg; m.p. 97-98 °C.

[α]$^{25}_{D}$ =+17.2 (*c* = 2.0 in $CHCl_3$).

^{1}H NMR (400 MHz, $CDCl_3$) δ 7.88 (t, *J* = 9.1 Hz, 2H), 7.53 (d, *J* = 8.4 Hz, 1H), 7.43 – 7.47 (m, 1H), 7.33 – 7.37 (m, 1H), 7.32 – 7.20 (m, 2H), 6.92 (d, *J* = 7.8 Hz, 1H), 3.85 (d, *J* = 12.3 Hz, 1H), 3.68 (d, *J* = 12.3 Hz, 4H), 3.35 – 3.43 (m, 1H), 1.73 (s, 3H), 1.28 (dd, *J* = 6.9, 1.2 Hz, 6H).

^{13}C NMR (101 MHz, $CDCl_3$) δ 151.8, 137.3, 135.3, 135.1, 134.0, 133.4, 132.9, 128.6, 128.0, 126.8, 126.7, 126.6, 125.9, 125.9, 125.4, 122.1, 45.3, 27.1, 23.1, 22.7, 19.8.

HRMS (ESI, m/z) calcd. for $C_{21}H_{23}NONa^+$ $[M+Na]^+$: 328.1672, found: 328.1669.

(*S,E*)-*N'*-((1-(2-hydroxy-3-isopropyl-6-methylphenyl)naphthalen-2-yl)methyl)-*N*, *N*-dimethylacetimidamide (19)

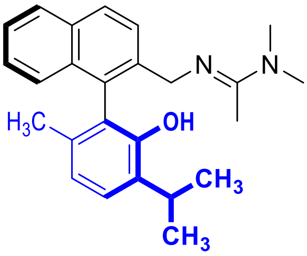

White solid, 76% yield, 92.0 mg; m.p. 270-271 °C.

[α]$^{25}_{D}$ = -43.5 (*c* = 0.3 in $CHCl_3$).

^{1}H NMR (400 MHz, $CDCl_3$) δ 7.94 – 7.78 (m, 3H), 7.44 – 7.48 (m, 1H), 7.38 – 7.28 (m, 3H), 7.22 (d, *J* = 7.8 Hz, 1H),

6.91 (d, J = 7.8 Hz, 1H), 4.54 (d, J = 15.3 Hz, 1H), 4.40 (d, J = 15.3 Hz, 1H), 3.42 – 2.95 (m, 7H), 1.76 (d, J = 4.3 Hz, 6H), 1.24 (dd, J = 6.9, 4.7 Hz, 6H).

^{13}C NMR (101 MHz, $CDCl_3$) δ 163.3, 150.9, 135.2, 133.4, 133.1, 132.9, 132.5, 132.2, 129.1, 128.3, 127.0, 126.9, 126.5, 126.0, 125.4, 123.4, 122.1, 46.7, 41.5, 26.9, 22.9, 22.9, 22.7, 19.6, 14.6.

HRMS (ESI, m/z) calcd. for $C_{25}H_{30}N_2OH^+$ $[M+H]^+$: 375.2431, found: 375.2432.

(*S*)-*N*-((1-(2-hydroxy-3-isopropyl-6-methylphenyl)naphthalen-2-yl)methyl)acetamide (20)

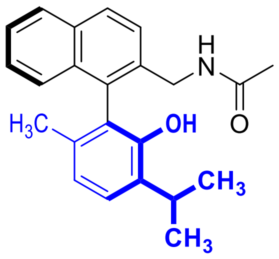

Colourless oil, 97% yield, 33.7 mg.

[α]$^{25}_{D}$ = -7.3 (c = 0.4 in $CHCl_3$).

^{1}H NMR (400 MHz, $CDCl_3$) δ 7.84 (d, J = 8.7 Hz, 2H), 7.53 (dd, J = 8.5, 3.5 Hz, 1H), 7.44 – 7.48 (m, 1H), 7.38 – 7.29 (m, 2H), 7.23 (d, J = 7.9 Hz, 1H), 6.92 (d, J = 7.9 Hz, 1H), 6.16 (t, J = 5.9 Hz, 1H), 4.26 – 4.32 (m, 1H), 3.99 – 4.05 (m, 1H), 3.26 – 3.36 (m, 1H), 1.82 (s, 3H), 1.76 (s, 3H), 1.30 – 1.25 (m, 6H).

^{13}C NMR (101 MHz, $CDCl_3$) δ 170.3, 150.4, 135.4, 135.1, 133.3, 133.2, 132.5, 132.0, 129.0, 128.2, 127.0, 126.6, 126.2, 125.9, 125.3, 124.1, 122.2, 42.2, 27.1, 23.0, 22.9, 22.7, 19.6.

HRMS (ESI, m/z) calcd. for $C_{23}H_{25}NO_2Na^+$ $[M+Na]^+$: 370.1778, found: 370.1769.

HPLC analysis: 96:4 er (AD-H column, 25 °C, *n*-hexane / *i*-PrOH = 80 / 20, 0.5 mL / min, λ = 254 nm), Rt (major) = 8.3 min, Rt (minor) = 9.1 min.

Dimethyl (*S*,*E*)-2-(1,3-diphenylallyl)malonate (23)

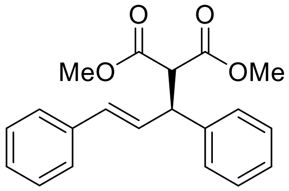

Colourless oil, 85% yield, 27.6 mg.

[α]$^{25}_{D}$ = -8.8 (c = 1.0 in $CHCl_3$).

^{1}H NMR (400 MHz, $CDCl_3$) δ 7.4 – 7.2 (m, 10H), 6.5 (d, J = 15.8 Hz, 1H), 6.3 (dd, J = 15.7, 8.6 Hz, 1H), 4.3 (dd, J = 11.1, 9.1 Hz, 1H), 4.0 (d, J = 10.9 Hz, 1H), 3.7 (s, 3H), 3.5 (s, 3H).

^{13}C NMR (101 MHz, $CDCl_3$) δ 168.3, 167.8, 140.2, 136.9, 131.9, 129.1, 128.8, 128.5, 127.9, 127.6, 127.2, 126.4, 57.7, 52.7, 52.5, 49.2.

HRMS (ESI, m/z) calcd. for $C_{20}H_{20}O_4Na^+$ [M+Na]$^+$: 347.1254, found: 347.1247.

HPLC analysis: 83:17 er (AD-H column, 25 °C, *n*-hexane / *i*-PrOH = 95 / 5, 1.0 mL / min, λ = 254 nm), Rt (major) = 22.0 min, Rt (minor) = 15.9 min.

(*S*)-1-(4-methoxyphenyl)propan-1-ol (25)

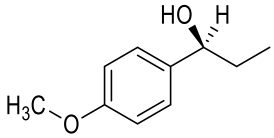

Colourless oil, 95% yield, 78.9 mg.

$[\alpha]^{25}_D$ = -16.8 (*c* = 1.5 in $CHCl_3$).

^{1}H NMR (400 MHz, $CDCl_3$) δ 7.23 (dd, *J* = 9.0, 2.5 Hz, 2H), 6.89 – 6.83 (m, 2H), 4.49 (t, *J* = 6.7 Hz, 1H), 3.78 (s, 3H), 2.13 (s, 1H), 1.86 – 1.64 (m, 2H), 0.87 (t, *J* = 7.4 Hz, 3H).

^{13}C NMR (101 MHz, $CDCl_3$) δ 159.0, 136.8, 127.2, 113.8, 75.6, 55.3, 31.8, 10.2.

HRMS (ESI, m/z) calcd. for $C_{10}H_{14}O_2Na^+$ [M+Na]$^+$: 189.0886, found: 189.0892.

HPLC analysis: 76:24 er (OD-H column, 25 °C, *n*-hexane / *i*-PrOH = 97 / 3, 1.0 mL / min, λ = 254 nm), Rt (major) = 20.5 min, Rt (minor) = 17.8 min.

(*R*)-5-(iodomethyl)-5-phenyldihydrofuran-2(3H)-one (28)

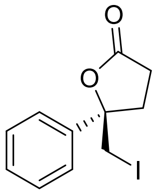

Colourless oil, 92% yield, 27.8 mg.

$[\alpha]^{25}_D$ = +4.5 (*c* = 1.0 in $CHCl_3$).

^{1}H NMR (400 MHz, $CDCl_3$) δ 7.4 – 7.3 (m, 5H), 3.6 (d, *J* = 1.4 Hz, 2H), 2.8 – 2.6 (m, 3H), 2.6 – 2.5 (m, 1H).

^{13}C NMR (101 MHz, $CDCl_3$) δ 175.4, 140.6, 128.9, 128.6, 124.9, 86.0, 34.0, 29.2, 16.3.

HRMS (ESI, m/z) calcd. for $C_{11}H_{11}IO_2Na^+$ [M+Na]$^+$: 324.9696, found: 324.9698.

HPLC analysis: 74:26 er (OD-H column, 25 °C, *n*-hexane / *i*-PrOH = 95 / 5, 1.0 mL / min, λ = 254 nm), Rt (major) = 25.5 min, Rt (minor) = 21.4 min.

Supplementary Figures - NMR and HPLC spectra

Supplementary Figure 10 ^{1}H NMR (400 MHz, $CDCl_3$) of **1a**

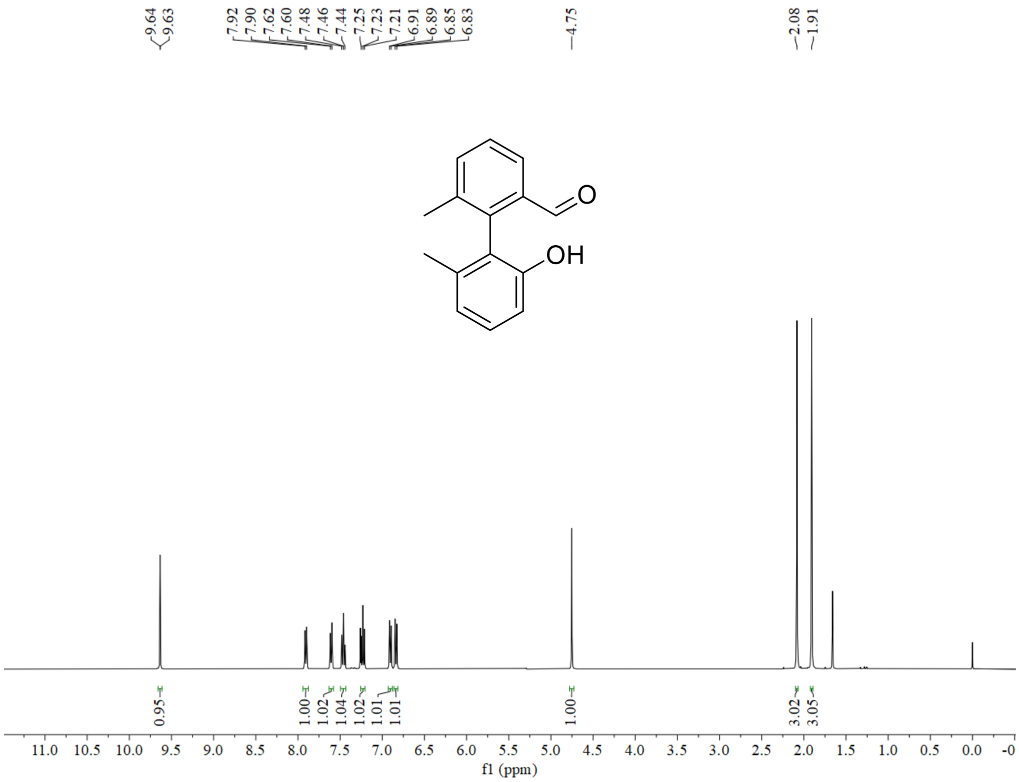

Supplementary Figure 11 ^{13}C NMR (400 MHz, $CDCl_3$) of **1a**

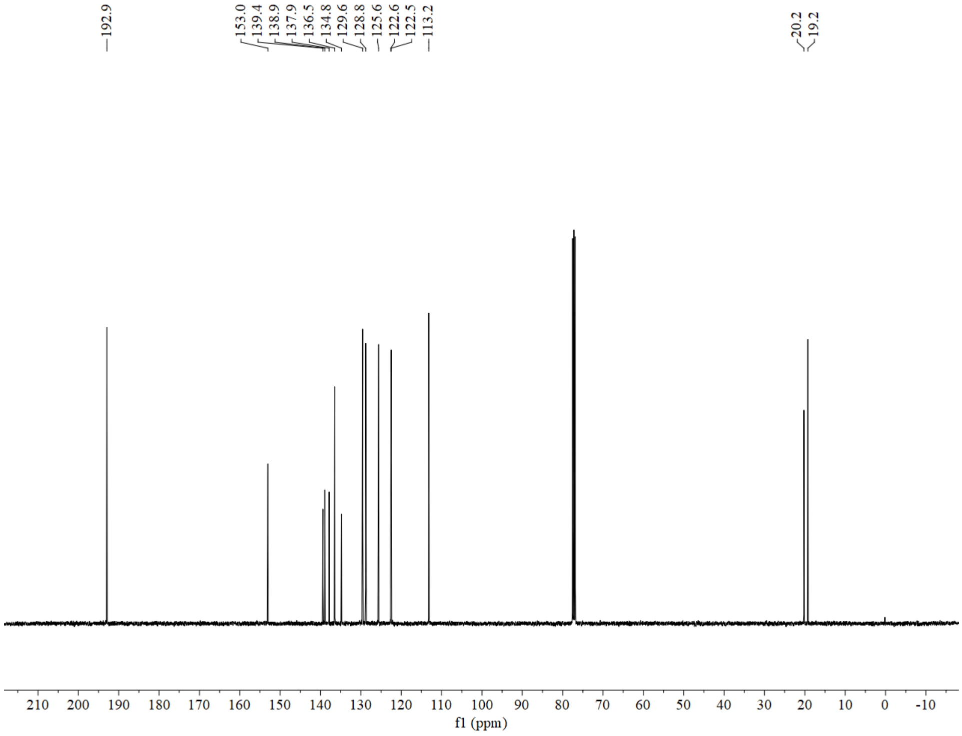

Supplementary Figure 12 ^{1}H NMR (400 MHz, $CDCl_3$) of **1b**

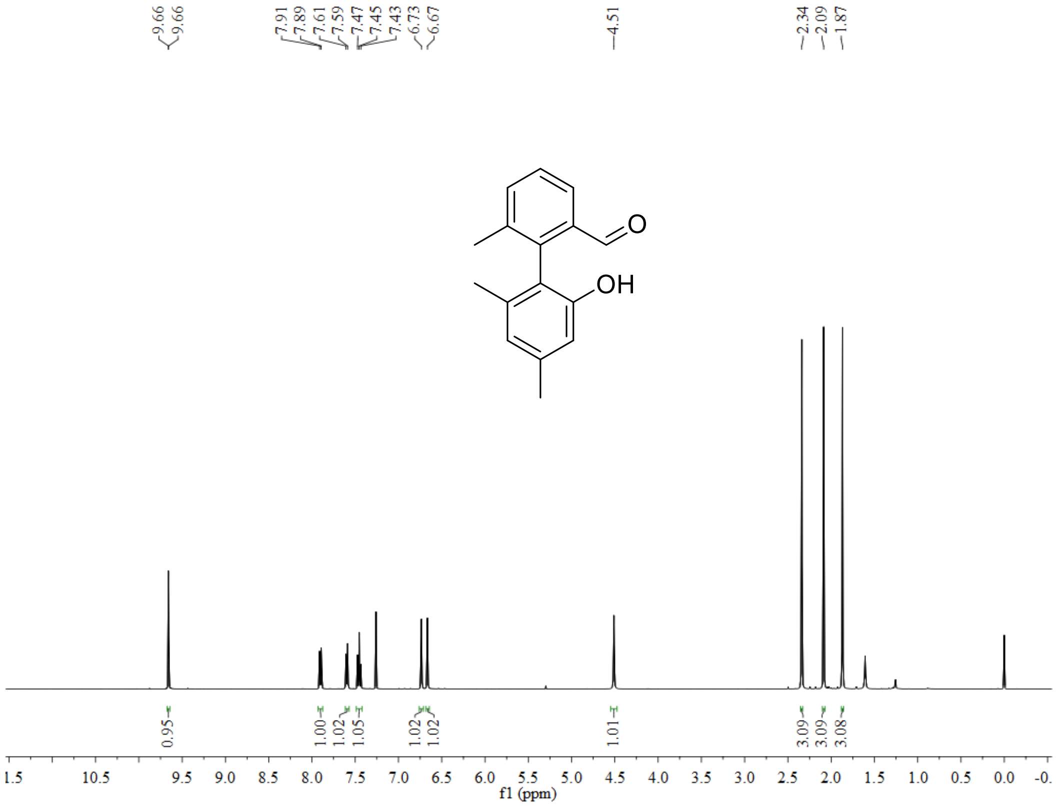

Supplementary Figure 13 ^{13}C NMR (400 MHz, $CDCl_3$) of **1b**

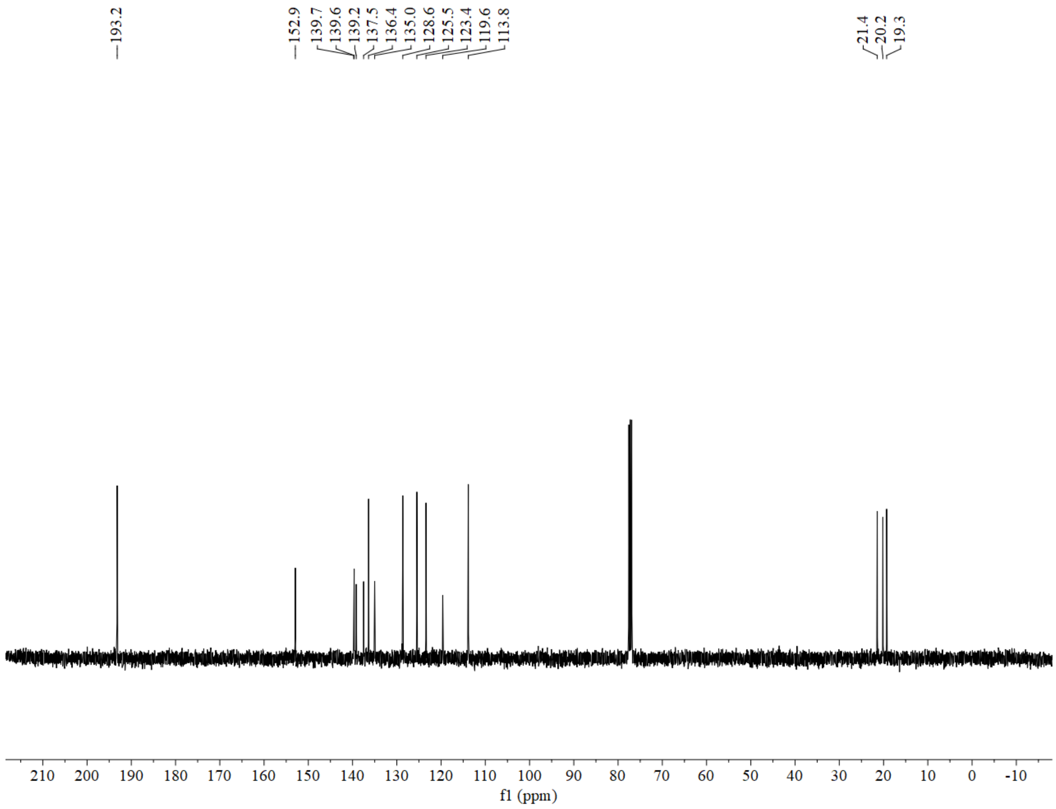

Supplementary Figure 14 ^{1}H NMR (400 MHz, $CDCl_3$) of **1c**

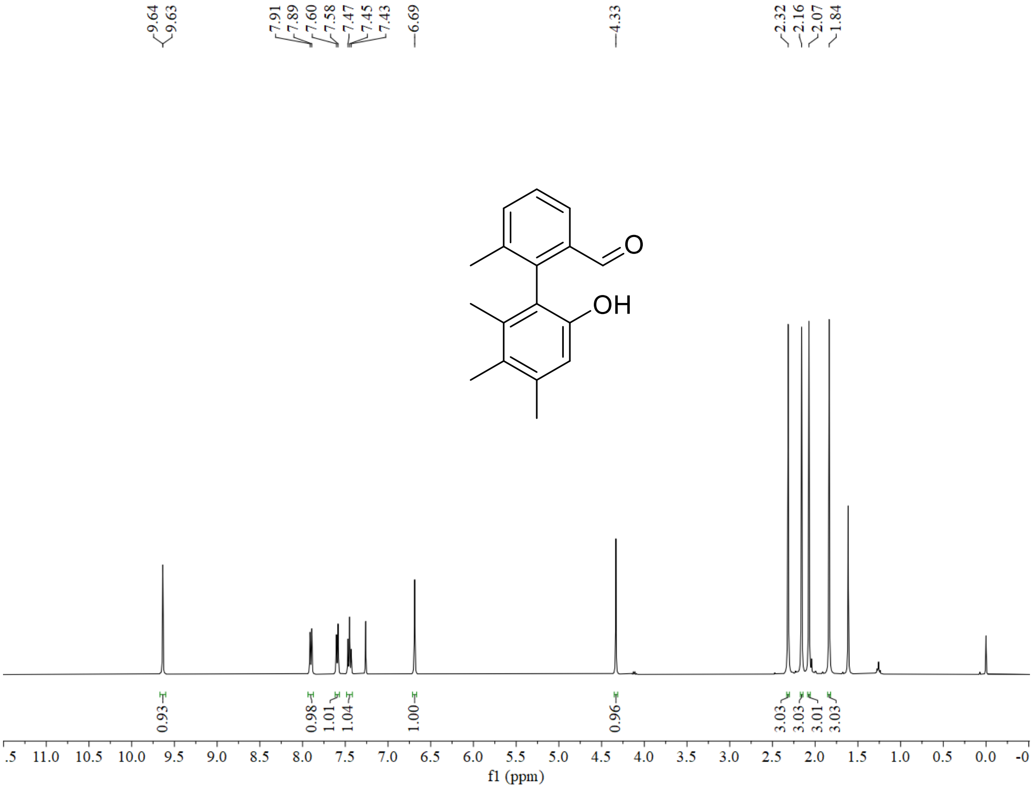

Supplementary Figure 15 ^{13}C NMR (400 MHz, $CDCl_3$) of **1c**

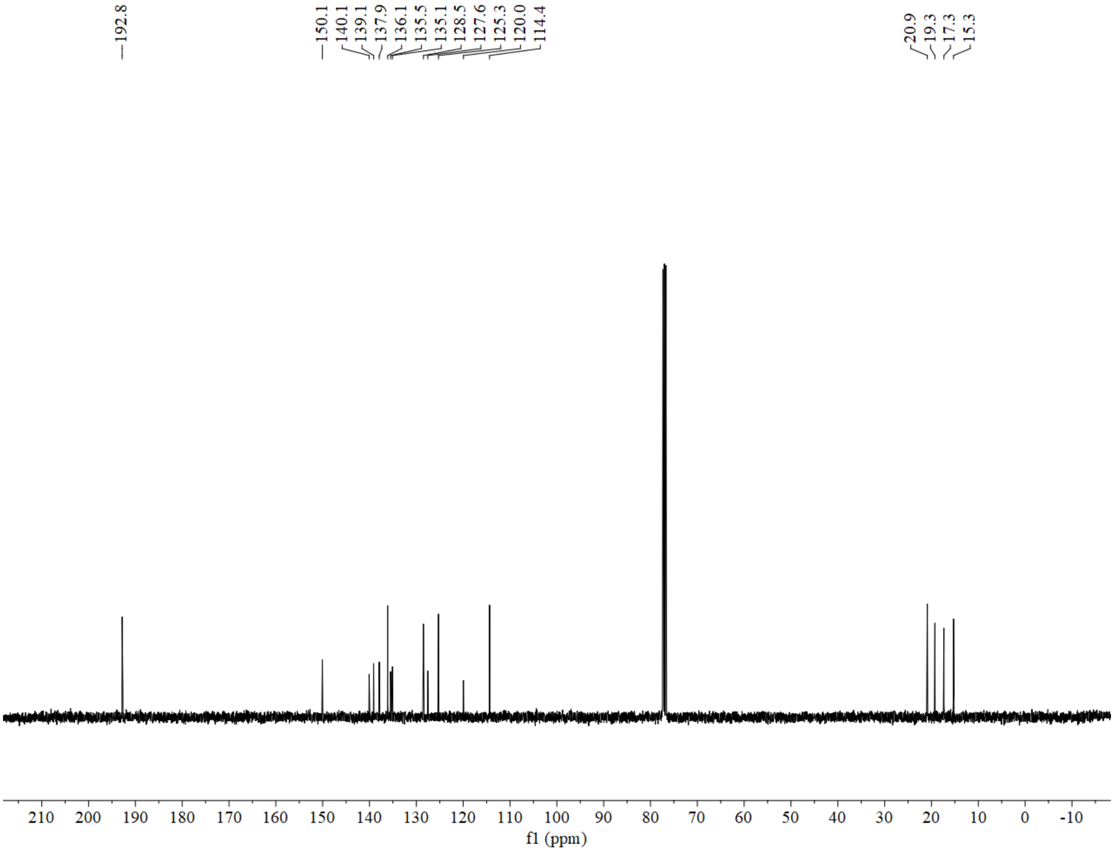

Supplementary Figure 16 ^{1}H NMR (400 MHz, $CDCl_3$) of **1d**

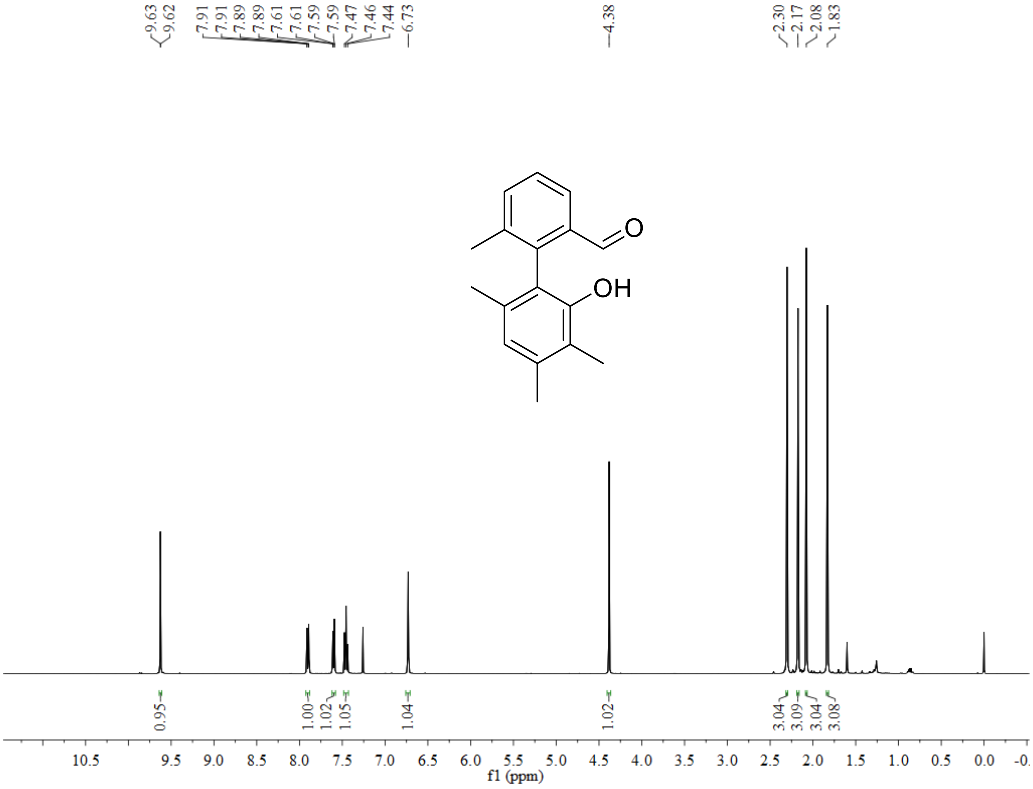

Supplementary Figure 17 ^{13}C NMR (400 MHz, $CDCl_3$) of **1d**

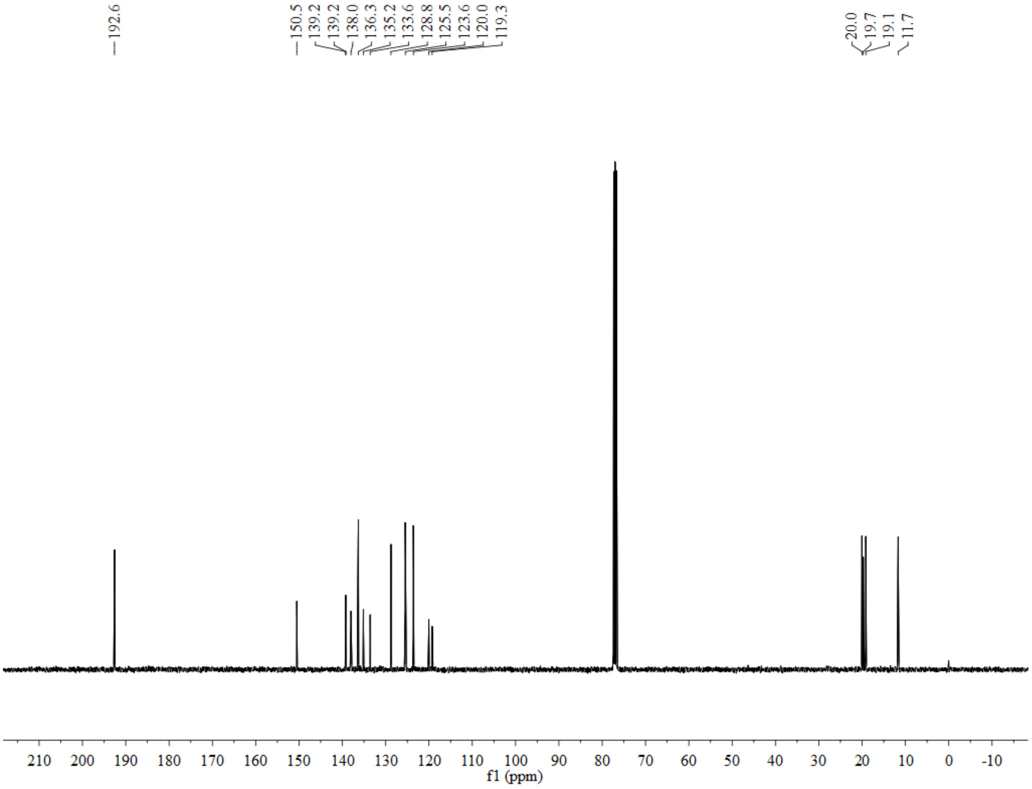

Supplementary Figure 18 ^{1}H NMR (400 MHz, Acetone-d_6) of **1e**

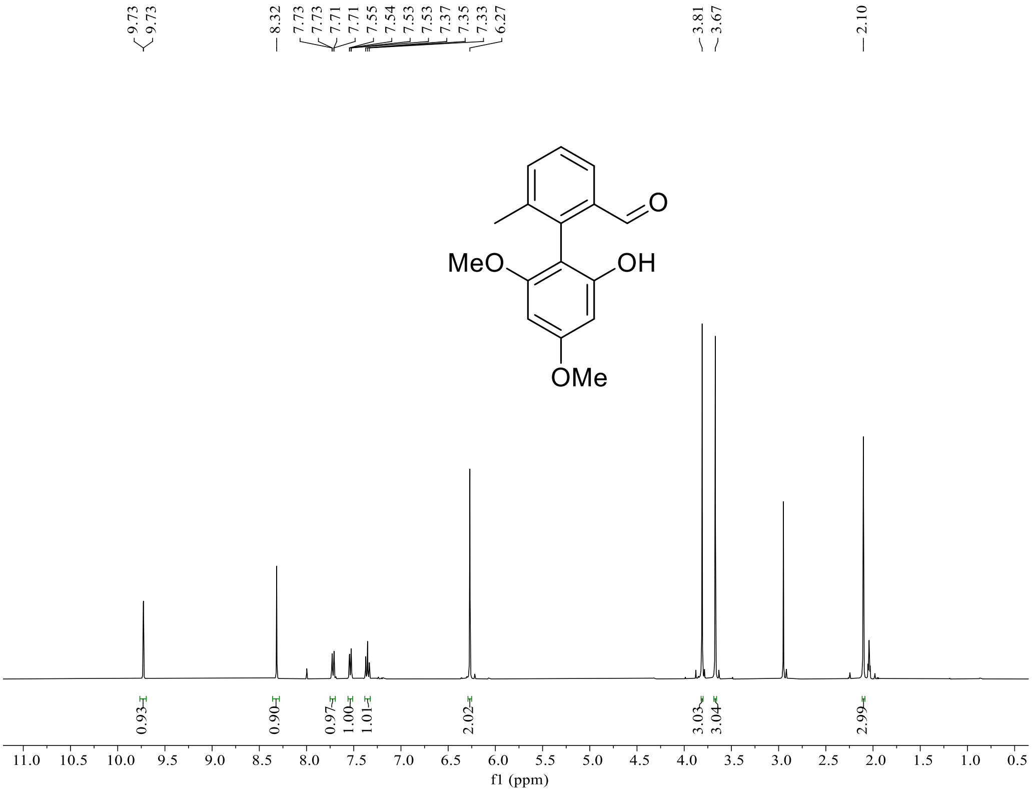

Supplementary Figure 19 ^{13}C NMR (400 MHz, Acetone-d_6) of **1e**

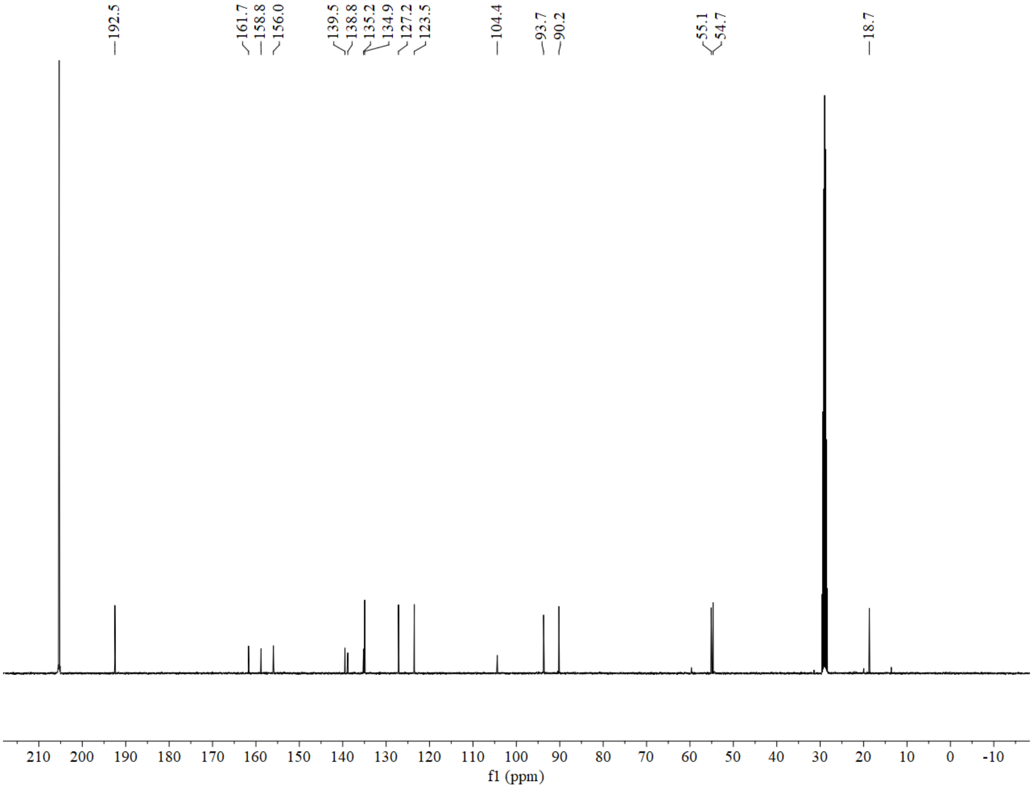

Supplementary Figure 20 ^{1}H NMR (400 MHz, $CDCl_3$) of **1f**

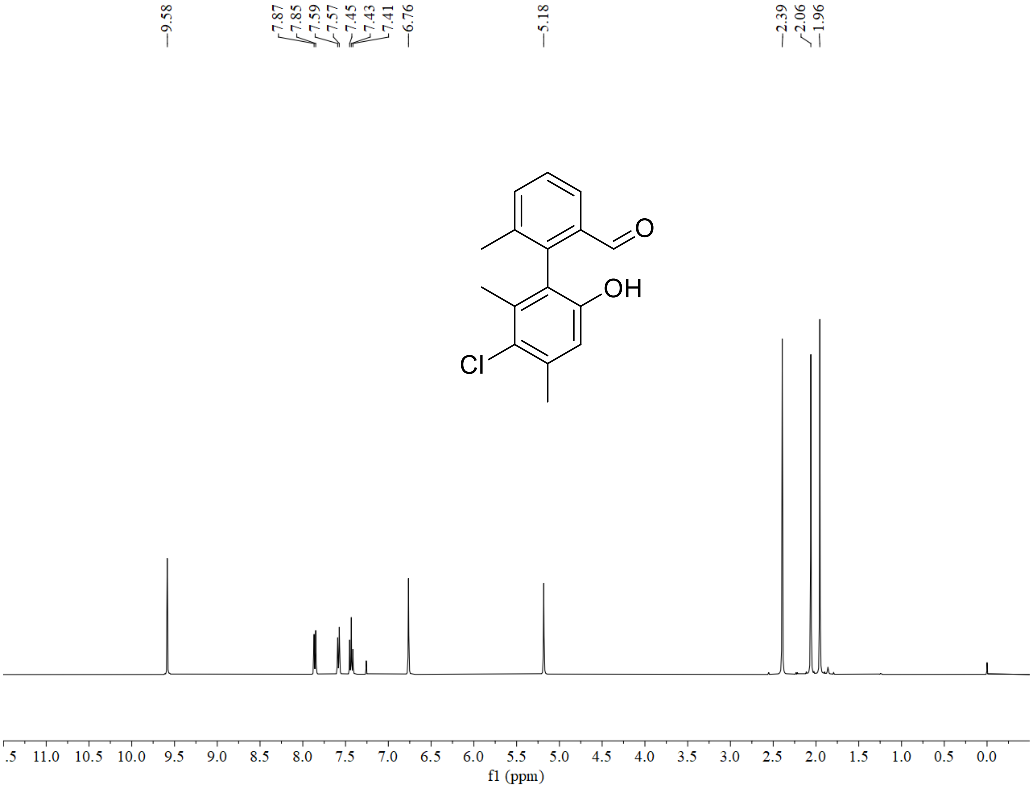

Supplementary Figure 21 ^{13}C NMR (400 MHz, $CDCl_3$) of **1f**

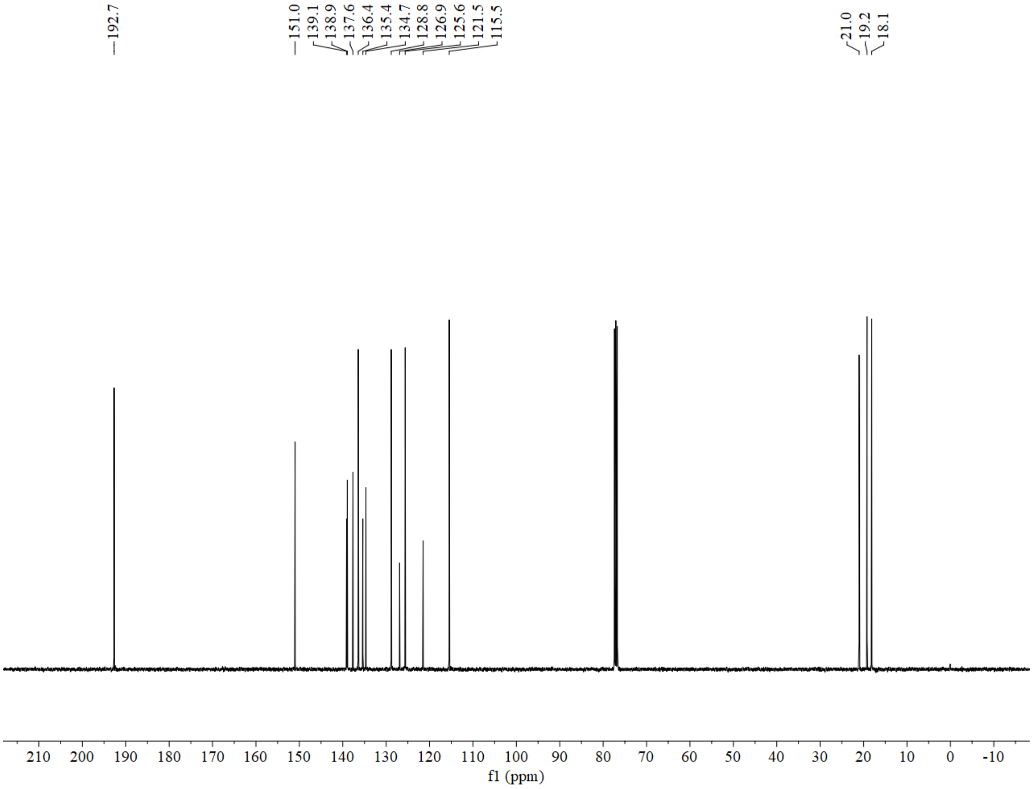

Supplementary Figure 22 ^{1}H NMR (400 MHz, $CDCl_3$) of **1g**

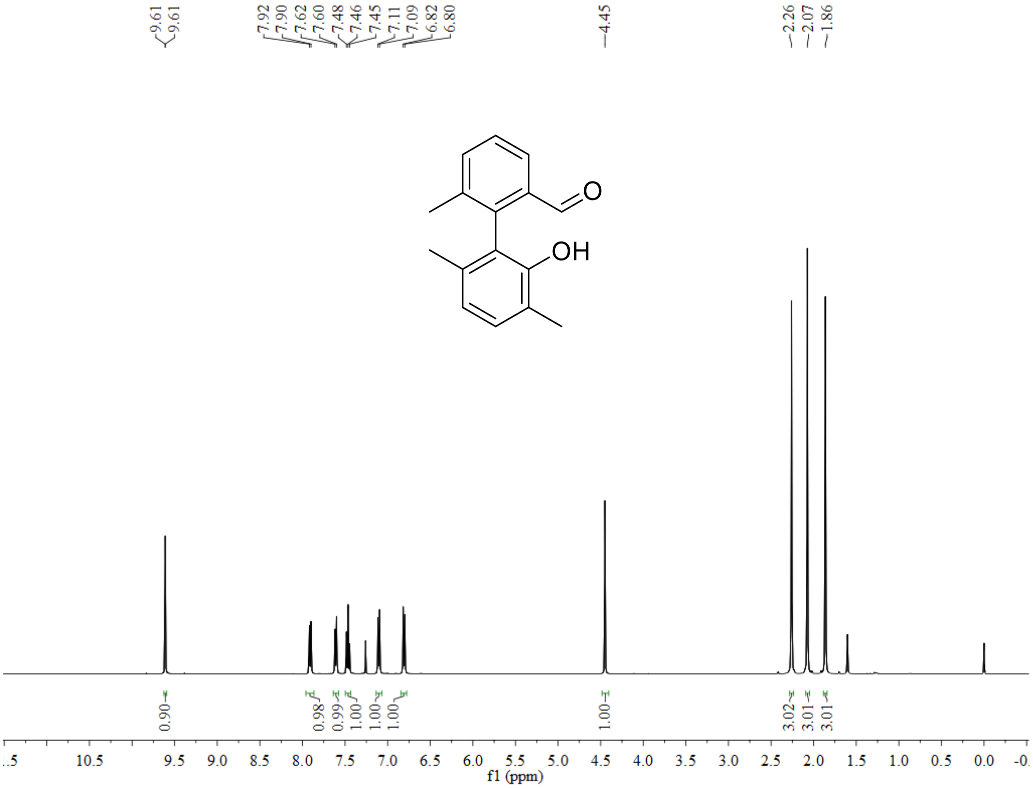

Supplementary Figure 23 ^{13}C NMR (400 MHz, $CDCl_3$) of **1g**

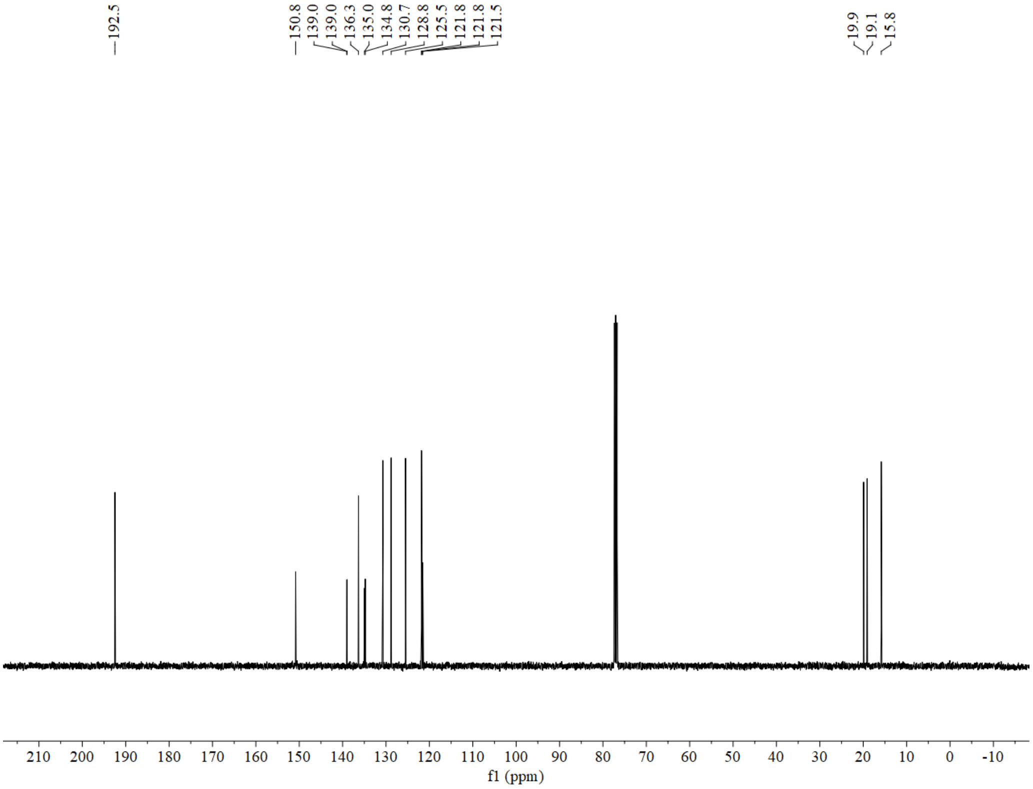

Supplementary Figure 24 ^{1}H NMR (400 MHz, $CDCl_3$) of **1h**

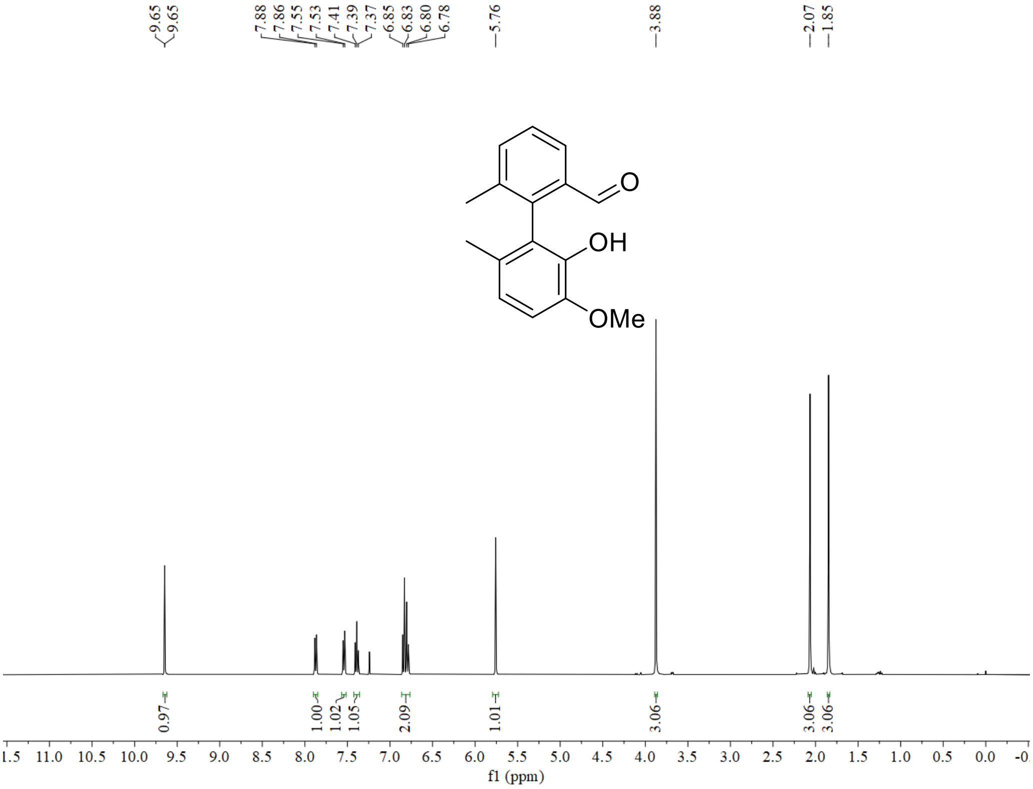

Supplementary Figure 25 ^{13}C NMR (400 MHz, $CDCl_3$) of **1h**

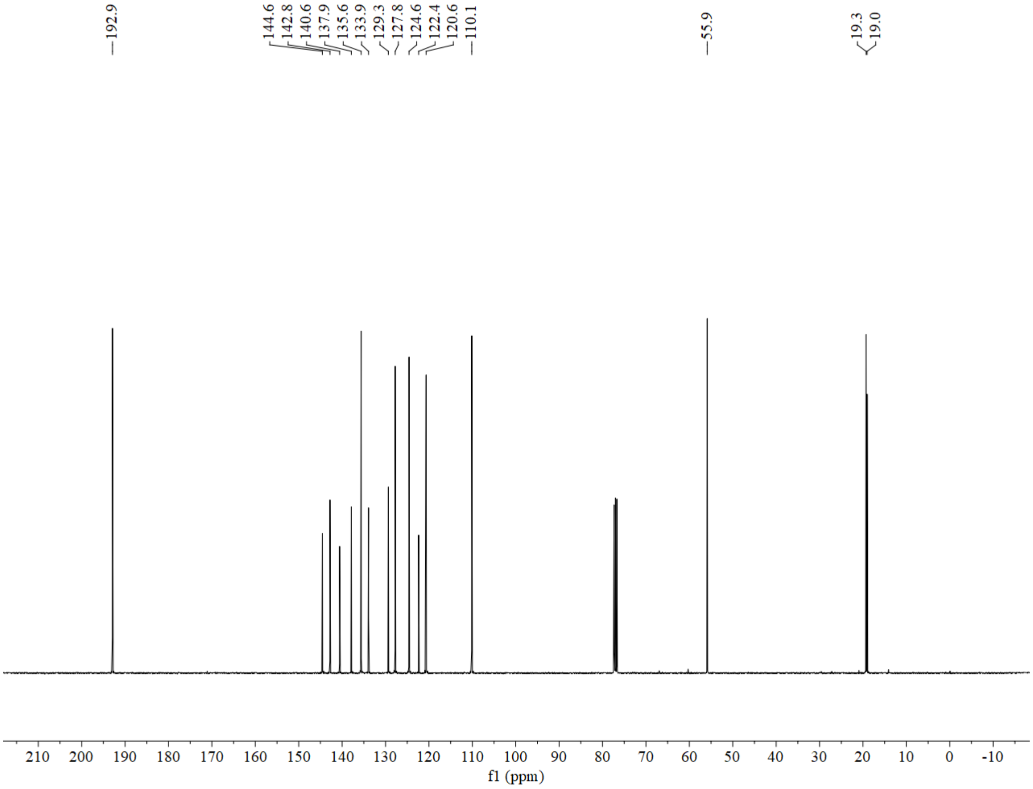

Supplementary Figure 26 ^{1}H NMR (400 MHz, $CDCl_3$) of **1i**

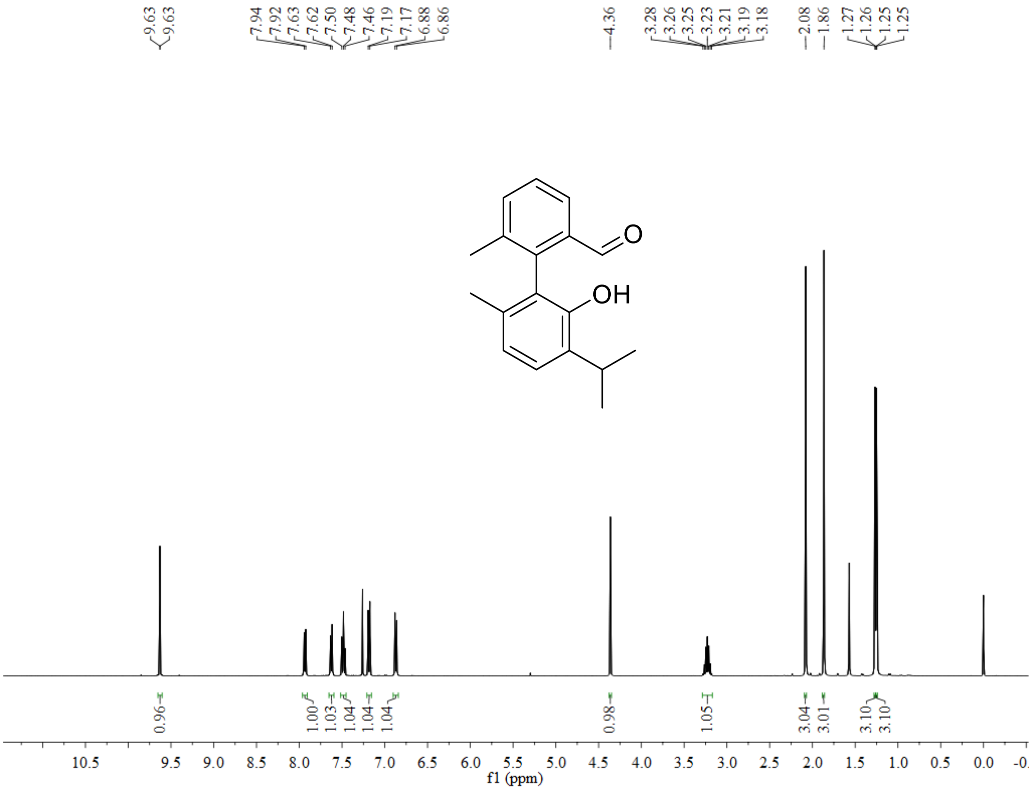

Supplementary Figure 27 ^{13}C NMR (400 MHz, $CDCl_3$) of **1i**

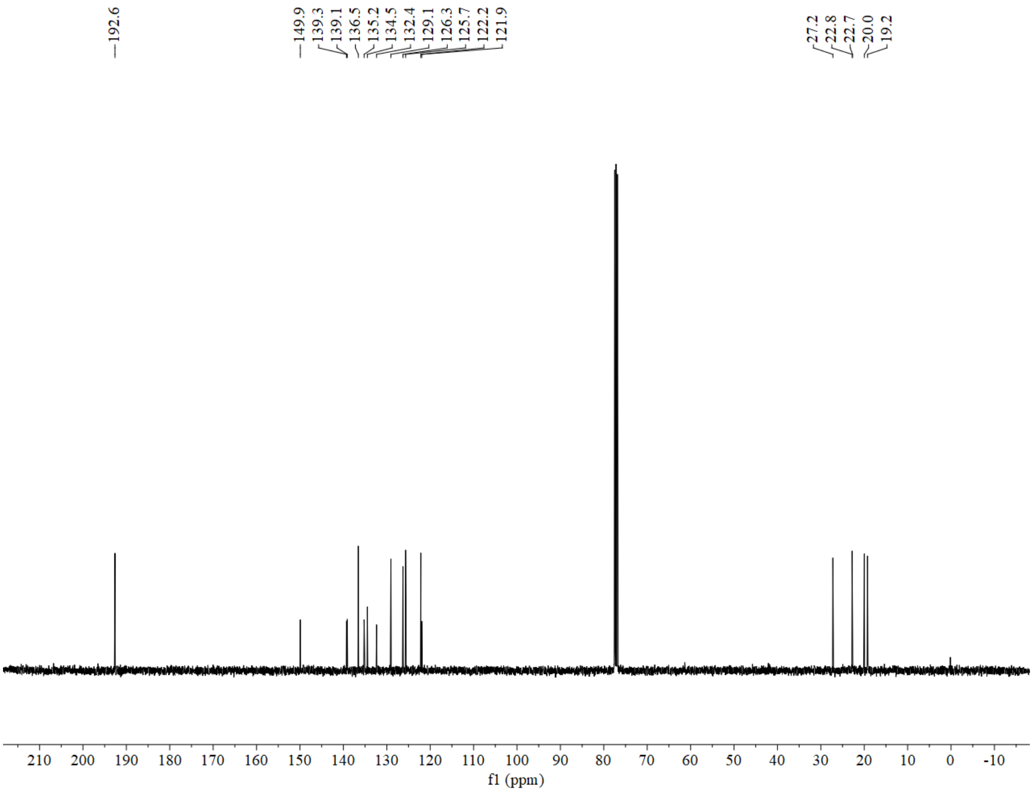

Supplementary Figure 28 ^{1}H NMR (400 MHz, $CDCl_3$) of **1j**

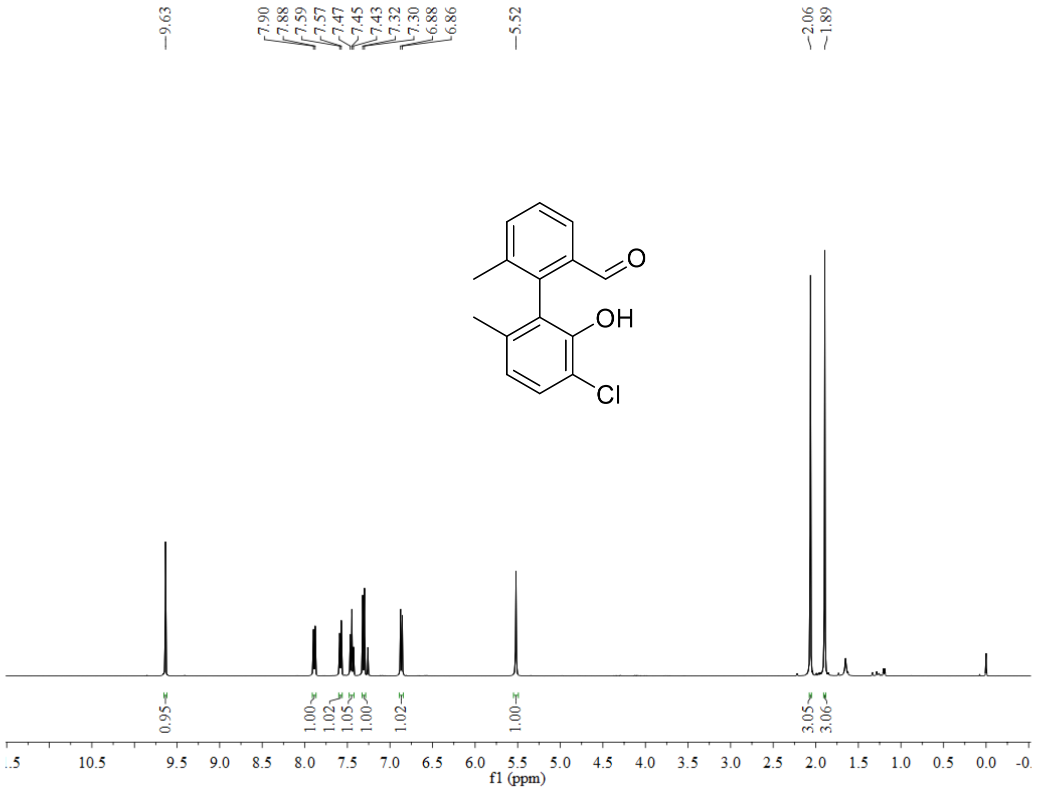

Supplementary Figure 29 ^{13}C NMR (400 MHz, $CDCl_3$) of **1j**

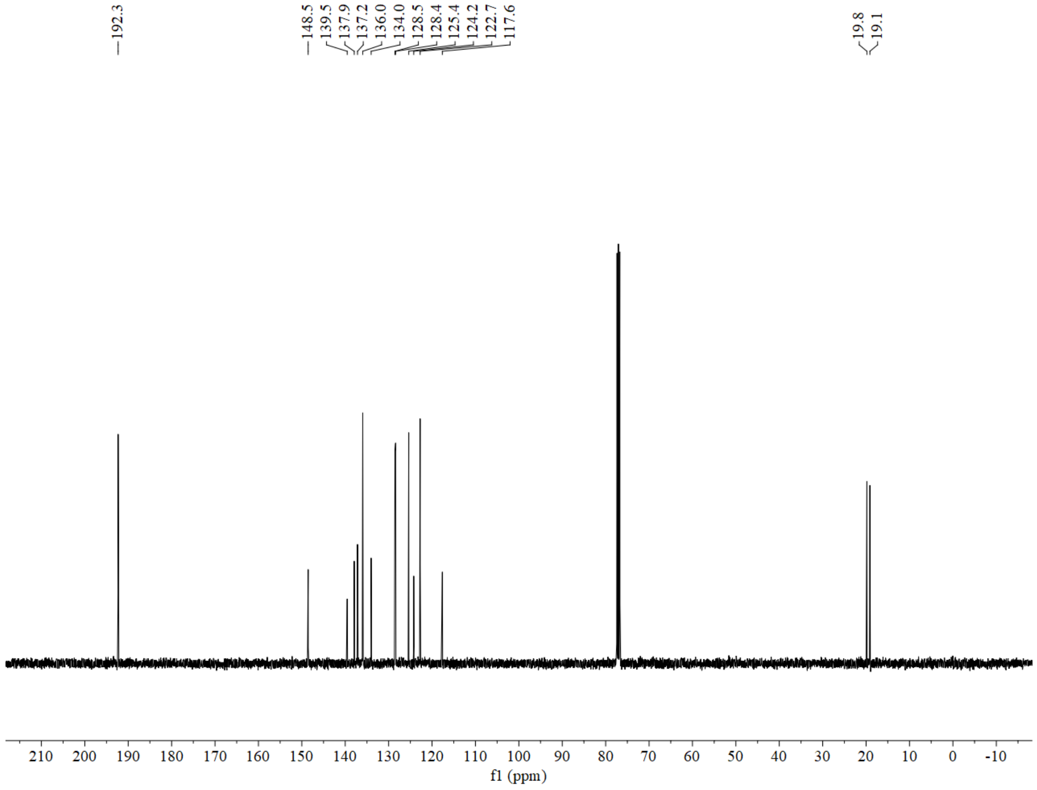

Supplementary Figure 30 ^{1}H NMR (400 MHz, Acetone-d_6) of **1k**

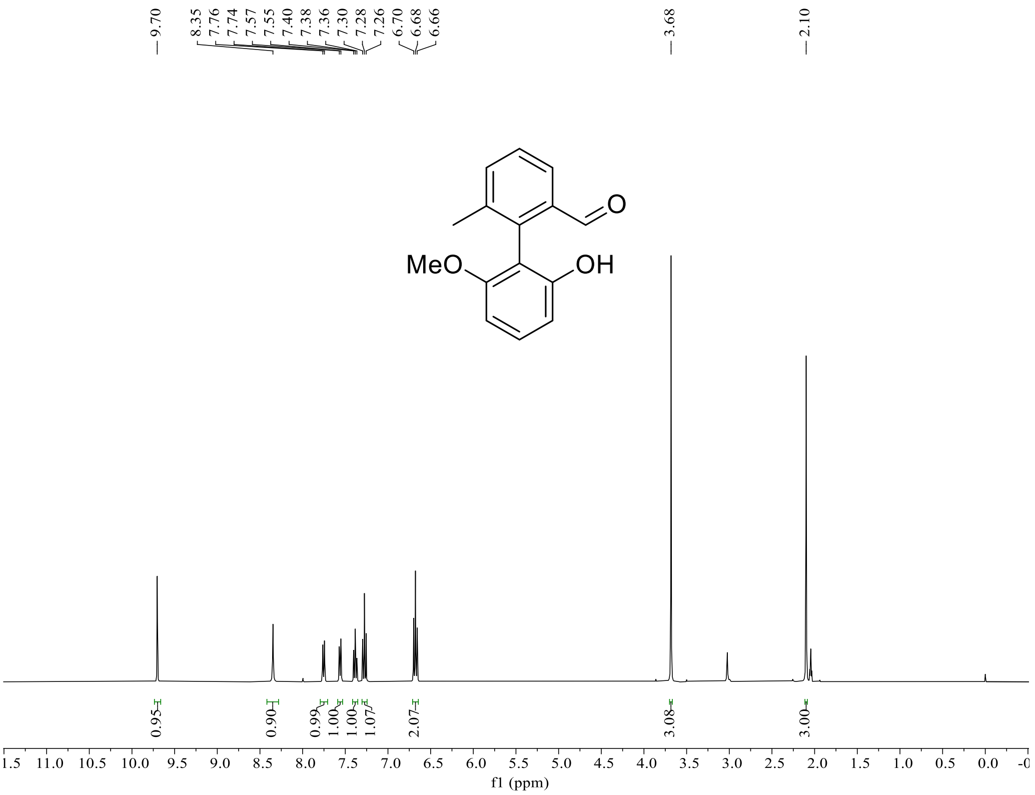

Supplementary Figure 31 ^{13}C NMR (400 MHz, Acetone-d_6) of **1k**

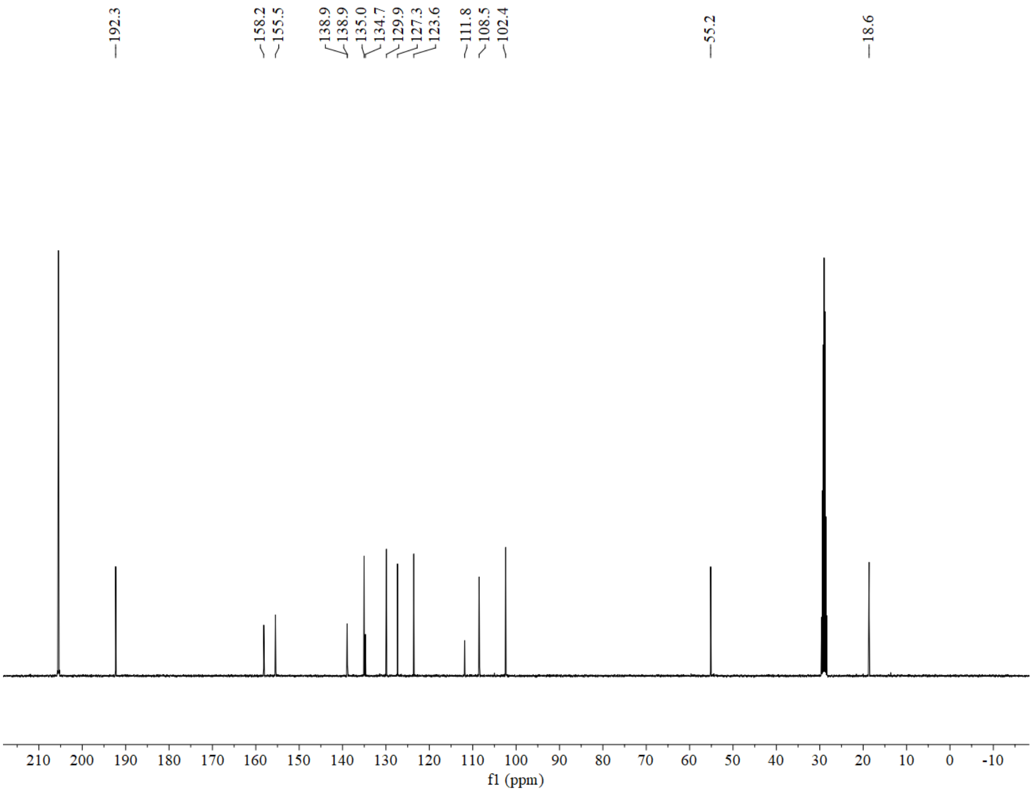

Supplementary Figure 32 ^{1}H NMR (400 MHz, $CDCl_3$) of **1l**

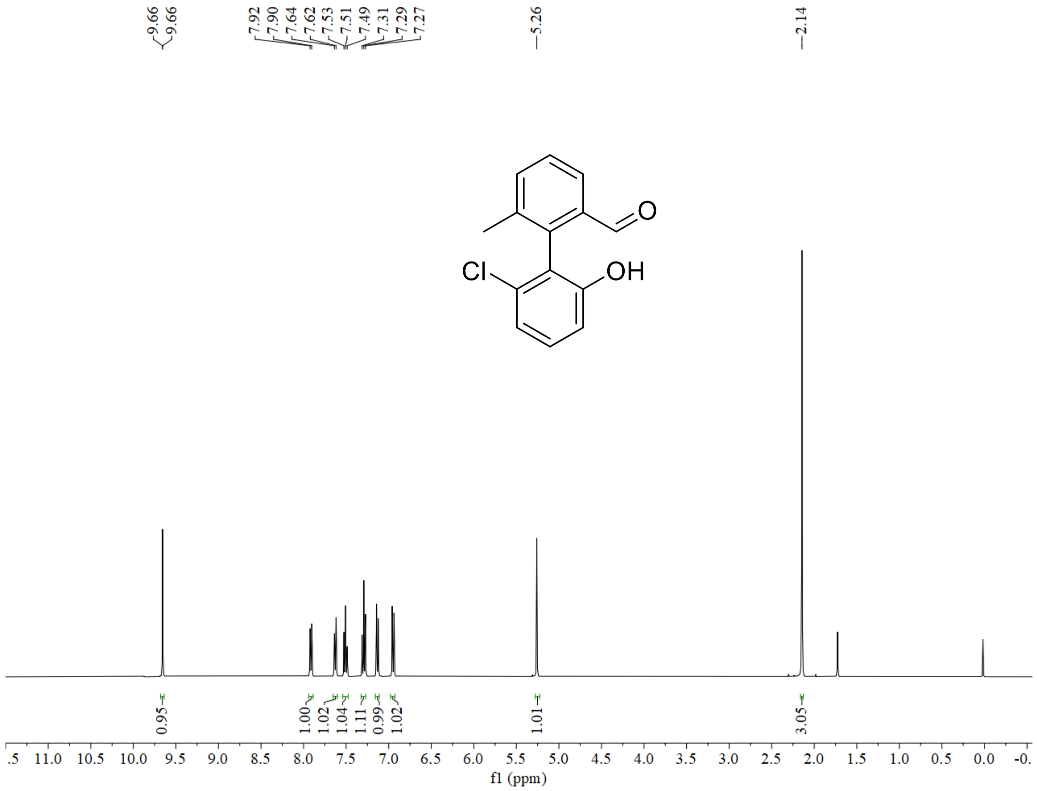

Supplementary Figure 33 ^{13}C NMR (400 MHz, $CDCl_3$) of **1l**

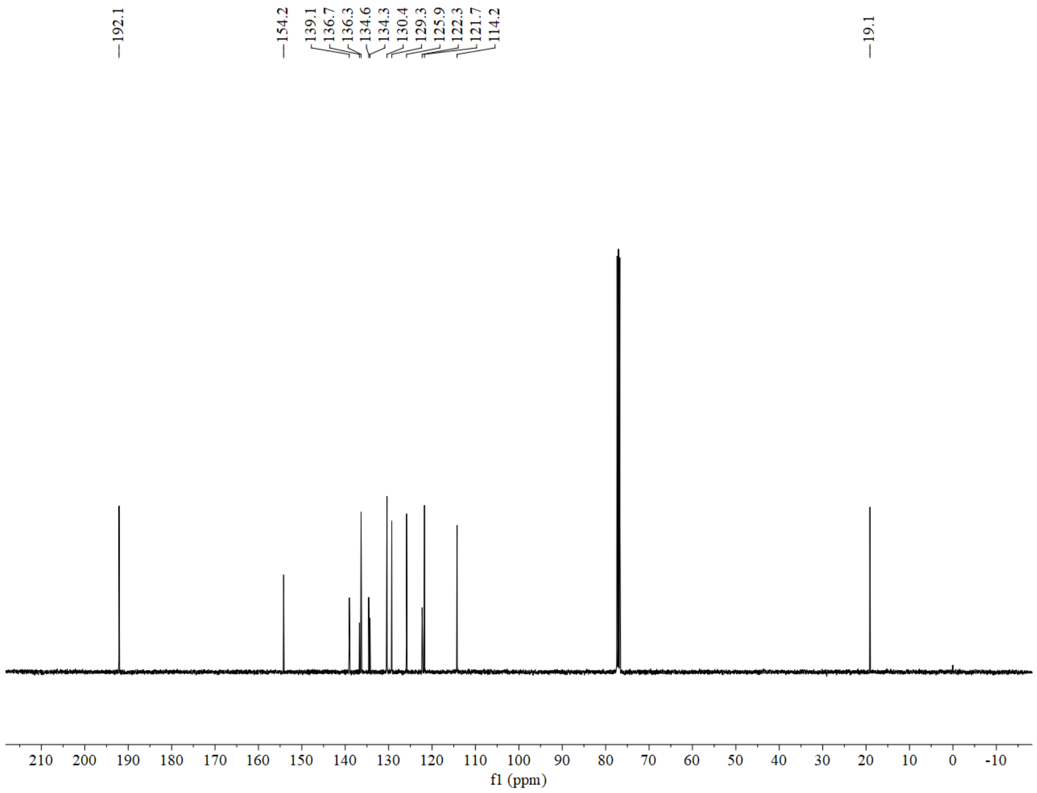

Supplementary Figure 34 ^{1}H NMR (400 MHz, $CDCl_3$) of **1m**

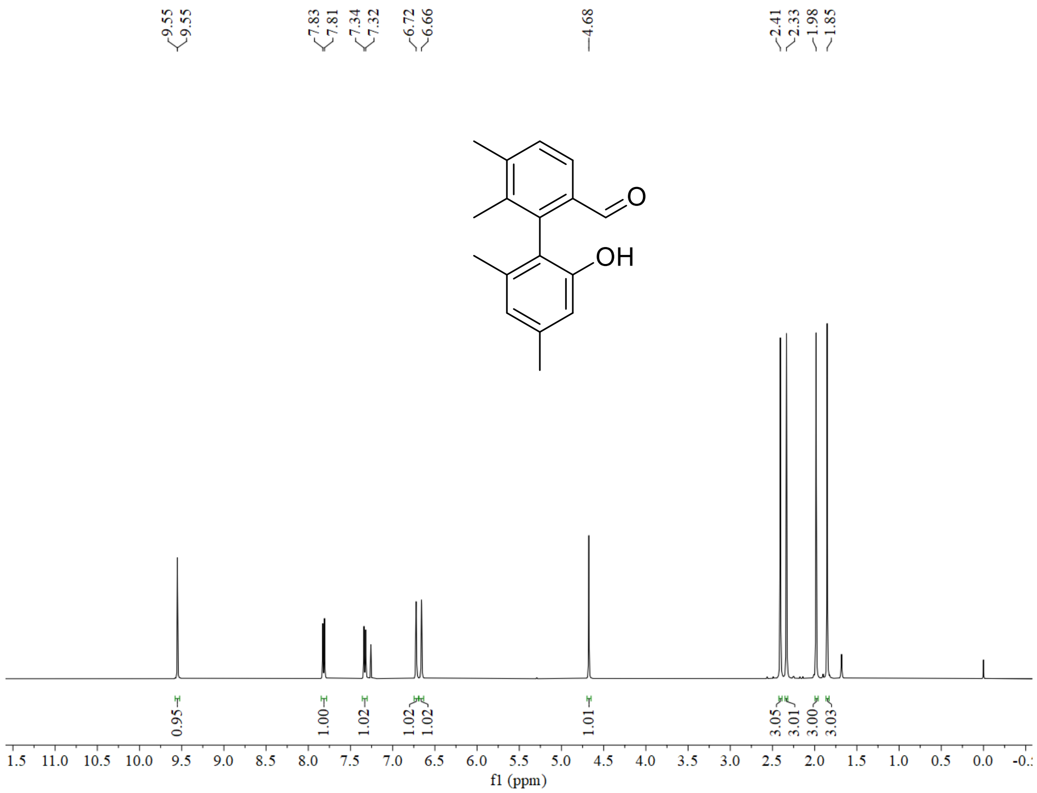

Supplementary Figure 35 ^{13}C NMR (400 MHz, $CDCl_3$) of **1m**

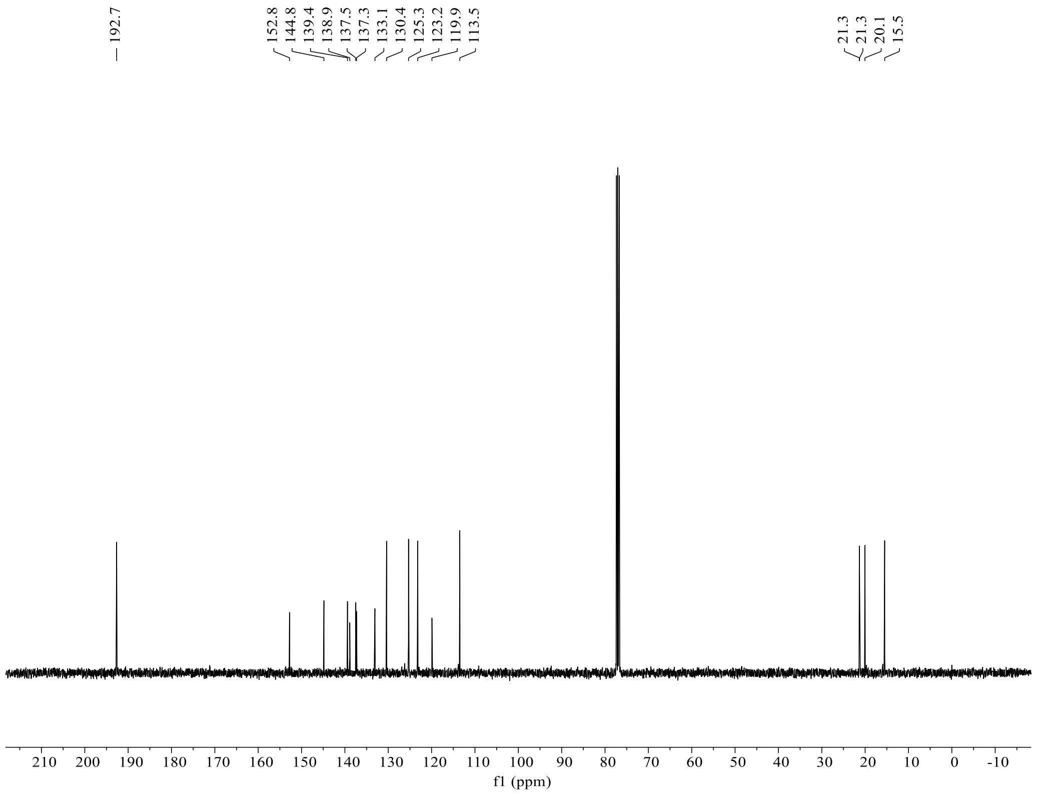

Supplementary Figure 36 ^{1}H NMR (400 MHz, $CDCl_3$) of **1n**

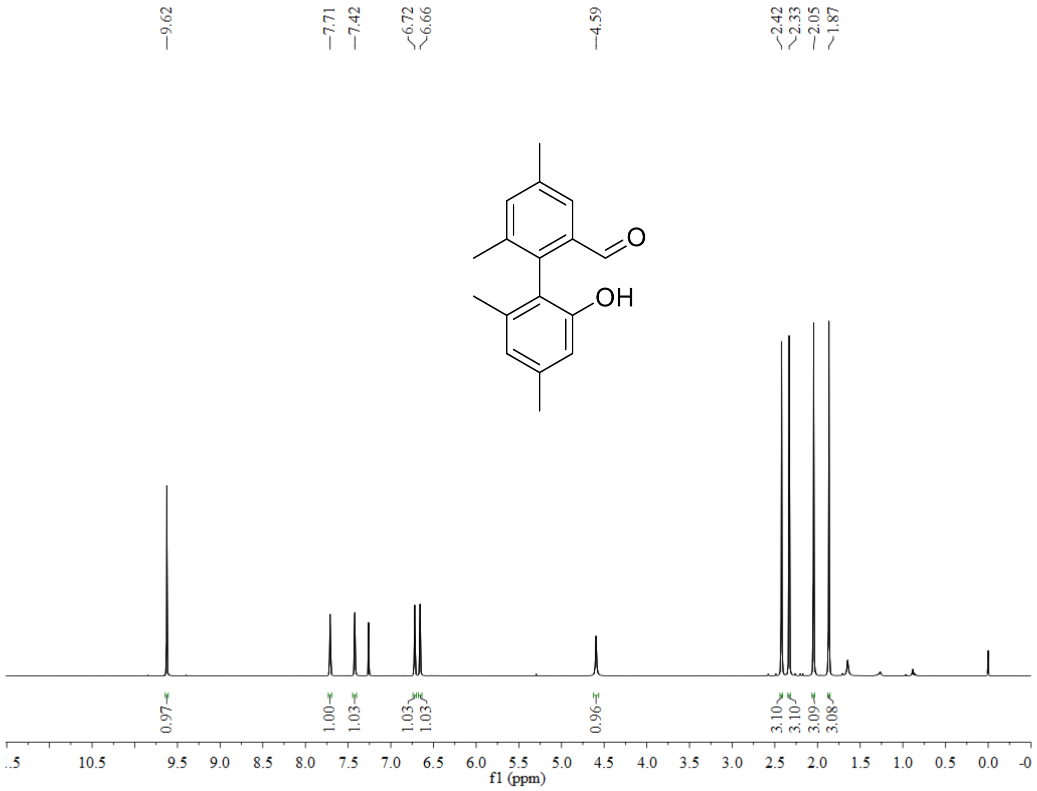

Supplementary Figure 37 ^{13}C NMR (400 MHz, $CDCl_3$) of **1n**

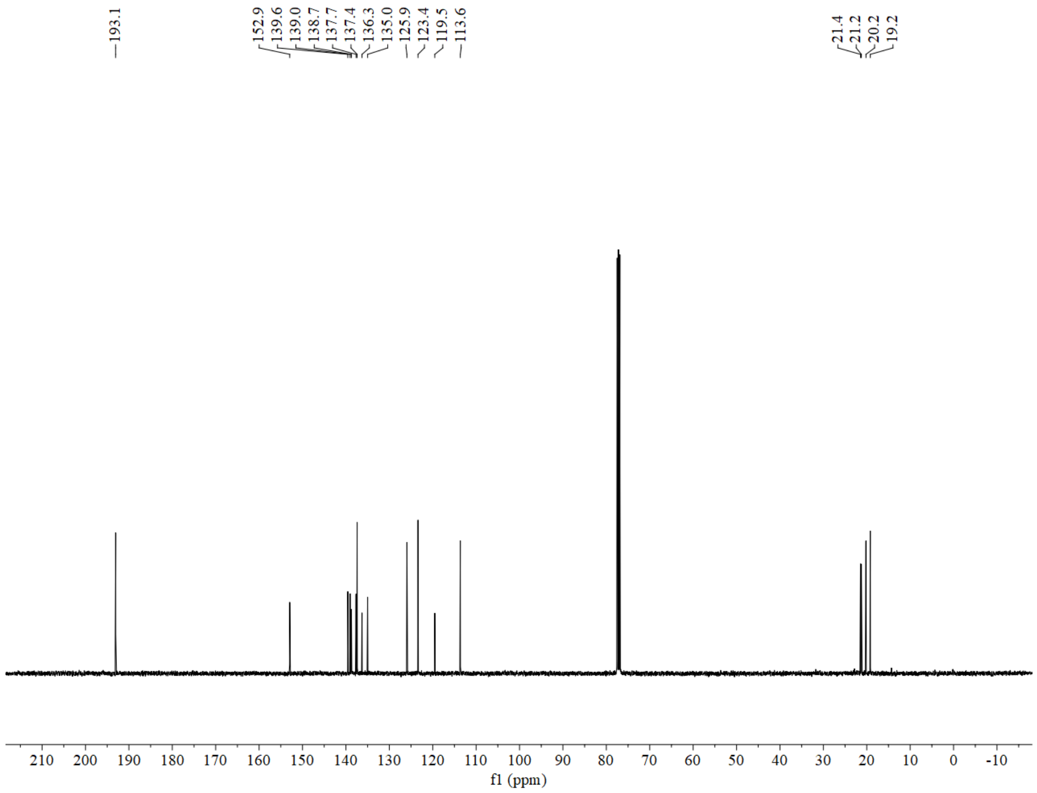

Supplementary Figure 38 ^{1}H NMR (400 MHz, $CDCl_3$) of **1o**

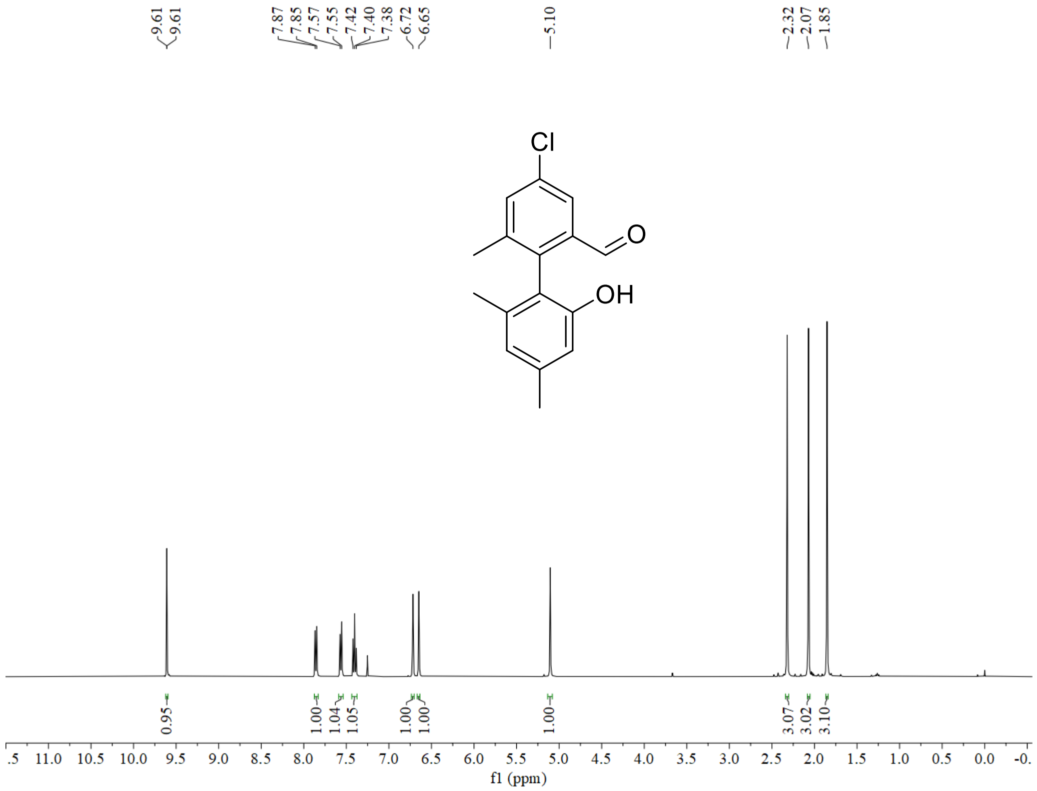

Supplementary Figure 39 ^{13}C NMR (400 MHz, $CDCl_3$) of **1o**

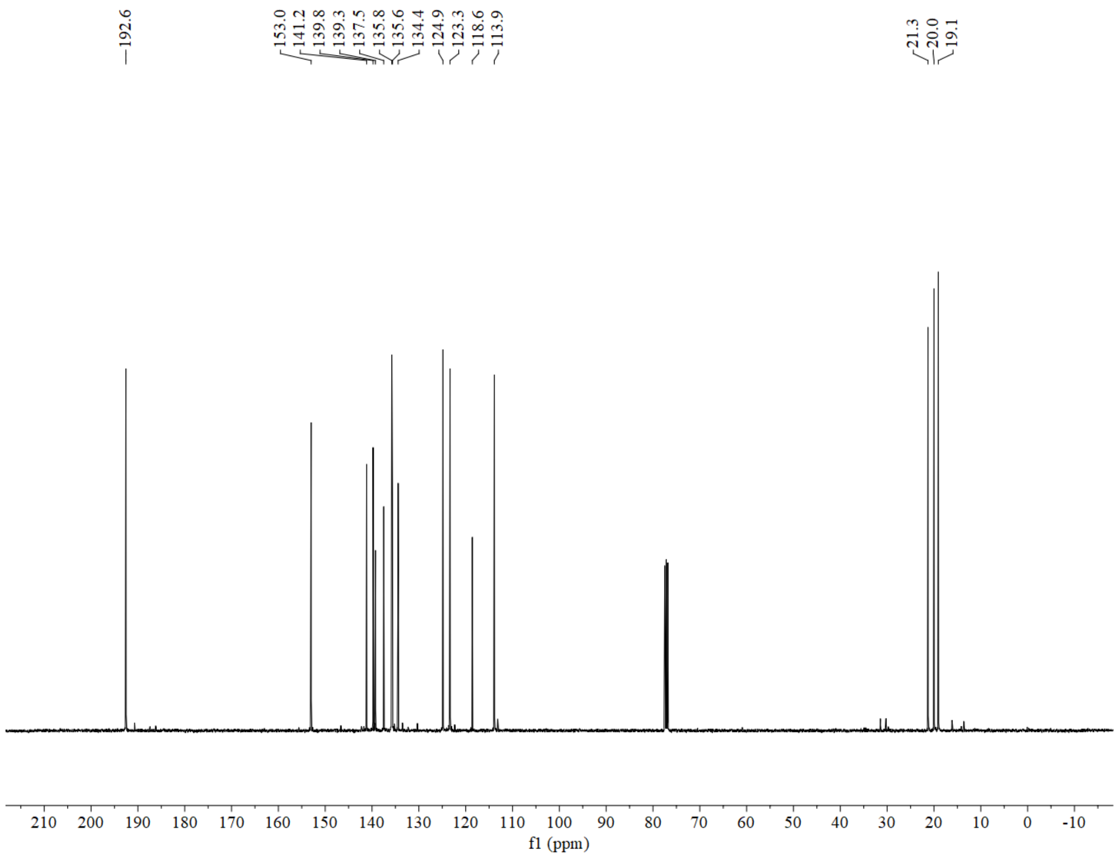

Supplementary Figure 40 ^{1}H NMR (400 MHz, Acetone-d_6) of **1p**

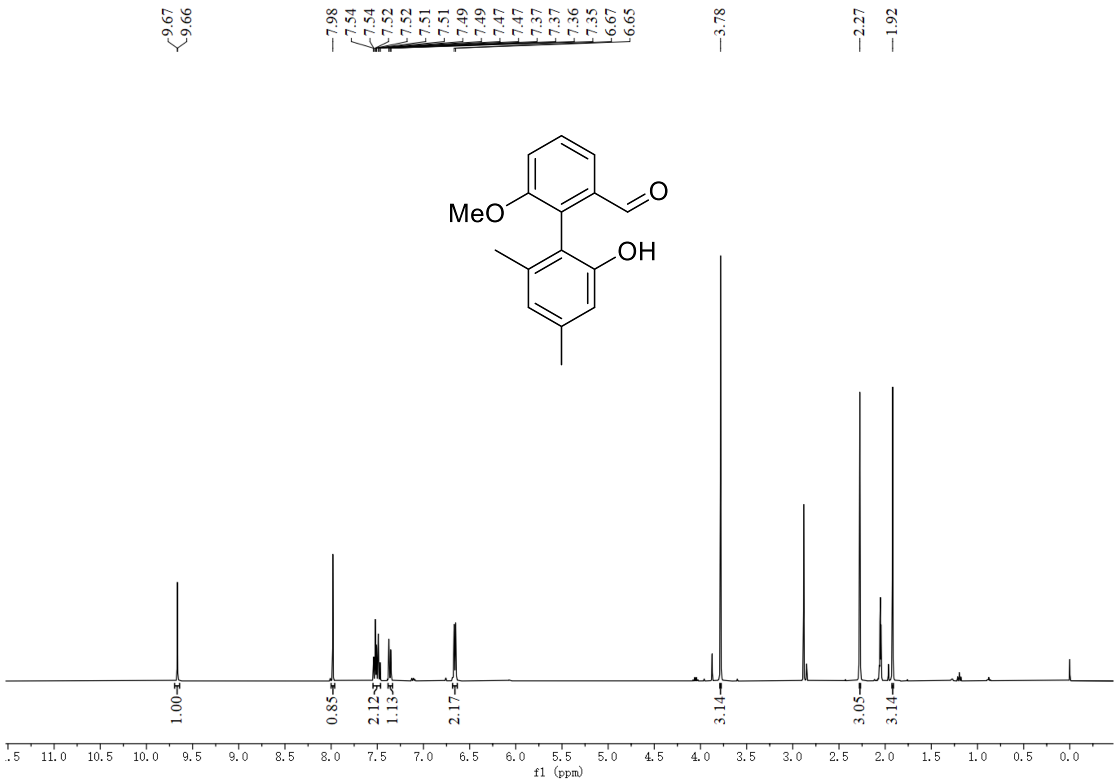

Supplementary Figure 41 ^{13}C NMR (400 MHz, Acetone-d_6) of **1p**

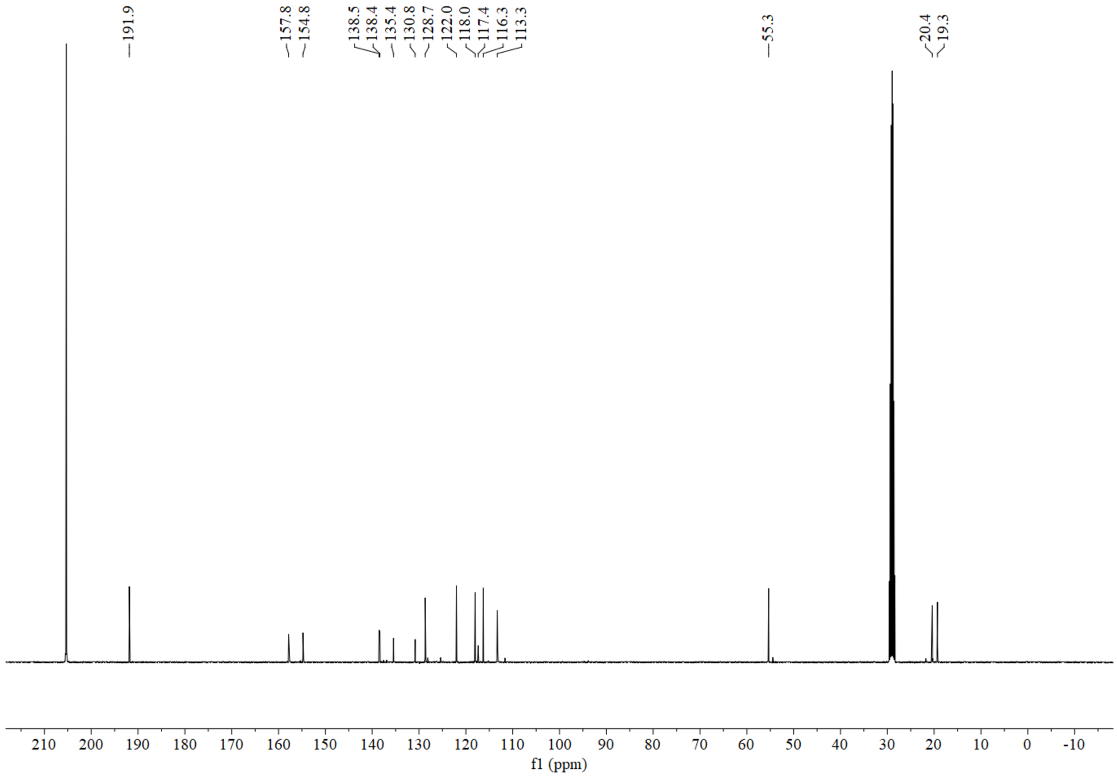

Supplementary Figure 42 ^{1}H NMR (400 MHz, $CDCl_3$) of **1q**

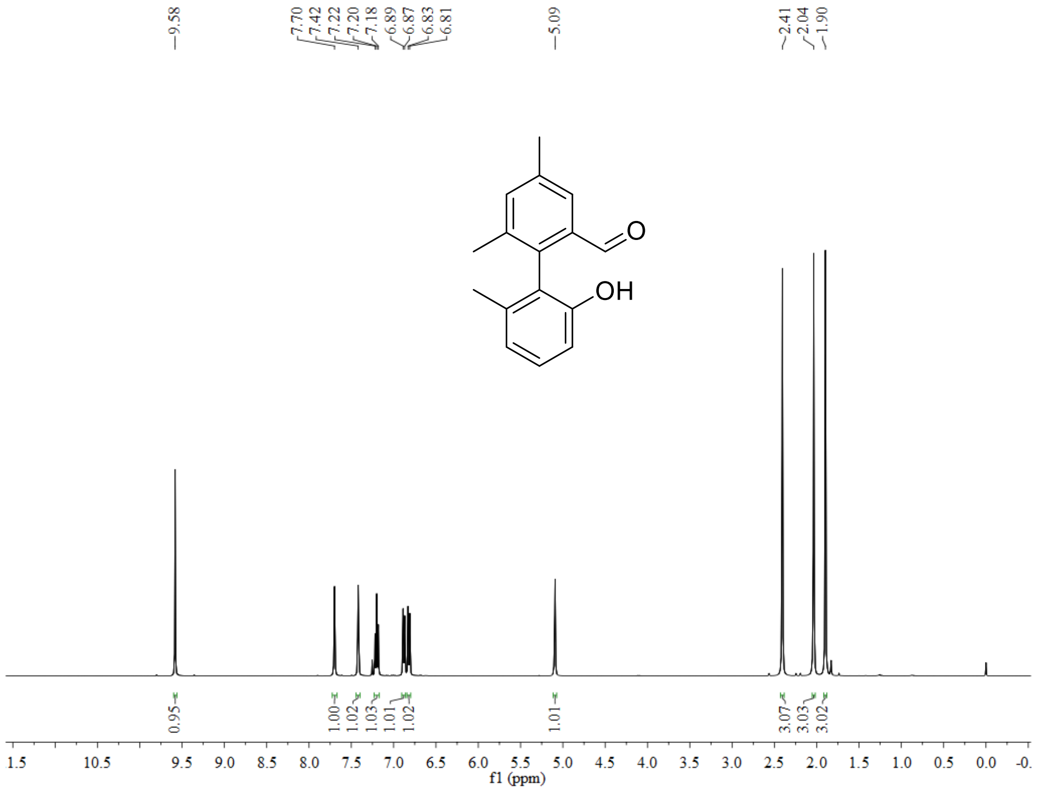

Supplementary Figure 43 ^{13}C NMR (400 MHz, $CDCl_3$) of **1q**

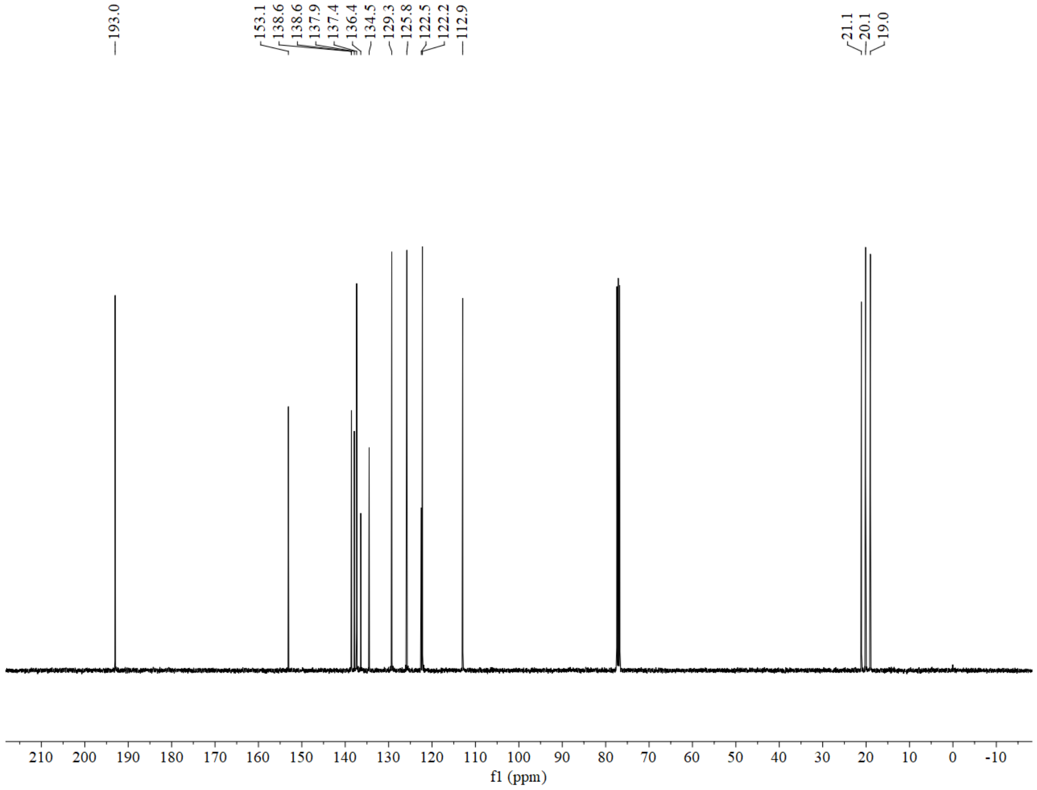

Supplementary Figure 44 ^{1}H NMR (400 MHz, Acetone-d_6) of **1r**

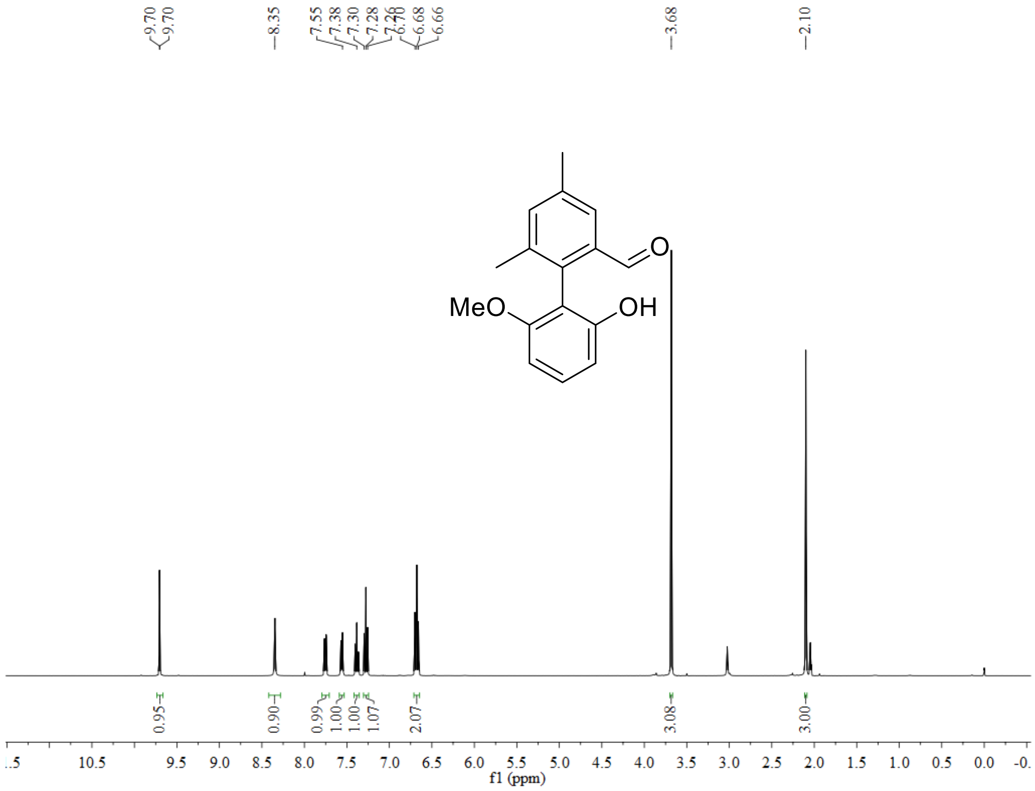

Supplementary Figure 45 ^{13}C NMR (400 MHz, Acetone-d_6) of **1r**

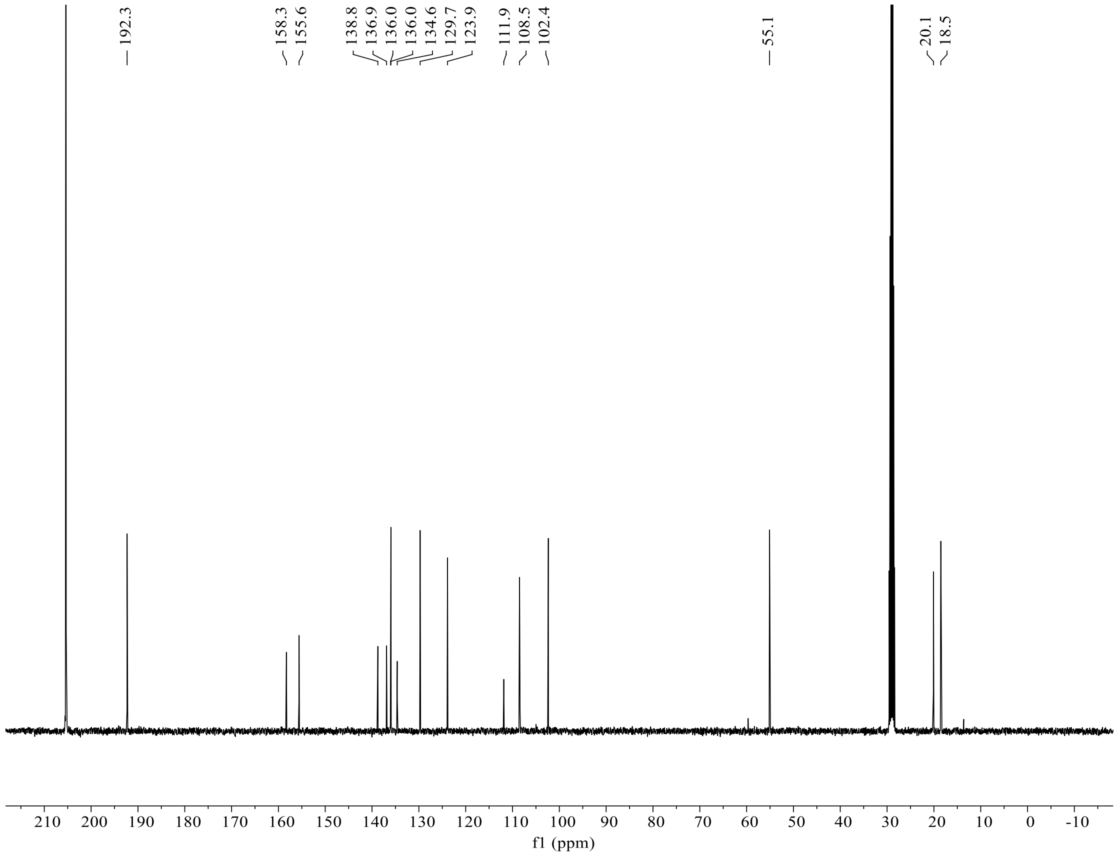

Supplementary Figure 46 ^{1}H NMR (400 MHz, $CDCl_3$) of **1s**

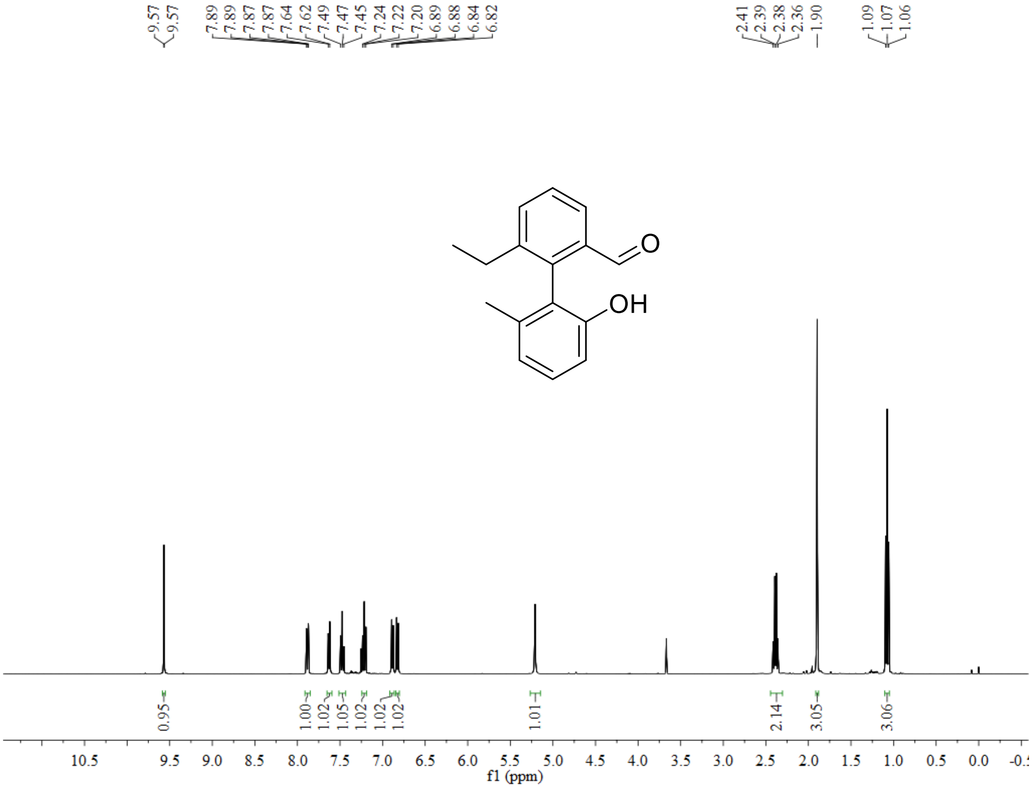

Supplementary Figure 47 ^{13}C NMR (400 MHz, $CDCl_3$) of **1s**

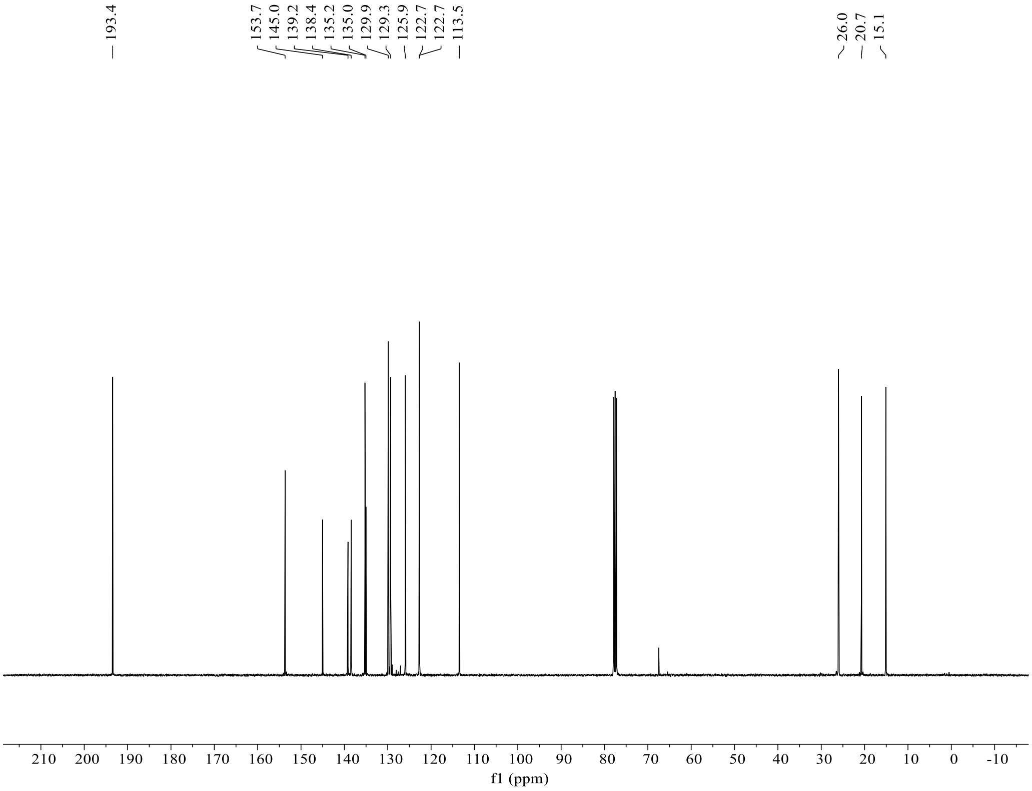

Supplementary Figure 48 ^{1}H NMR (400 MHz, Acetone-d_6) of **1t**

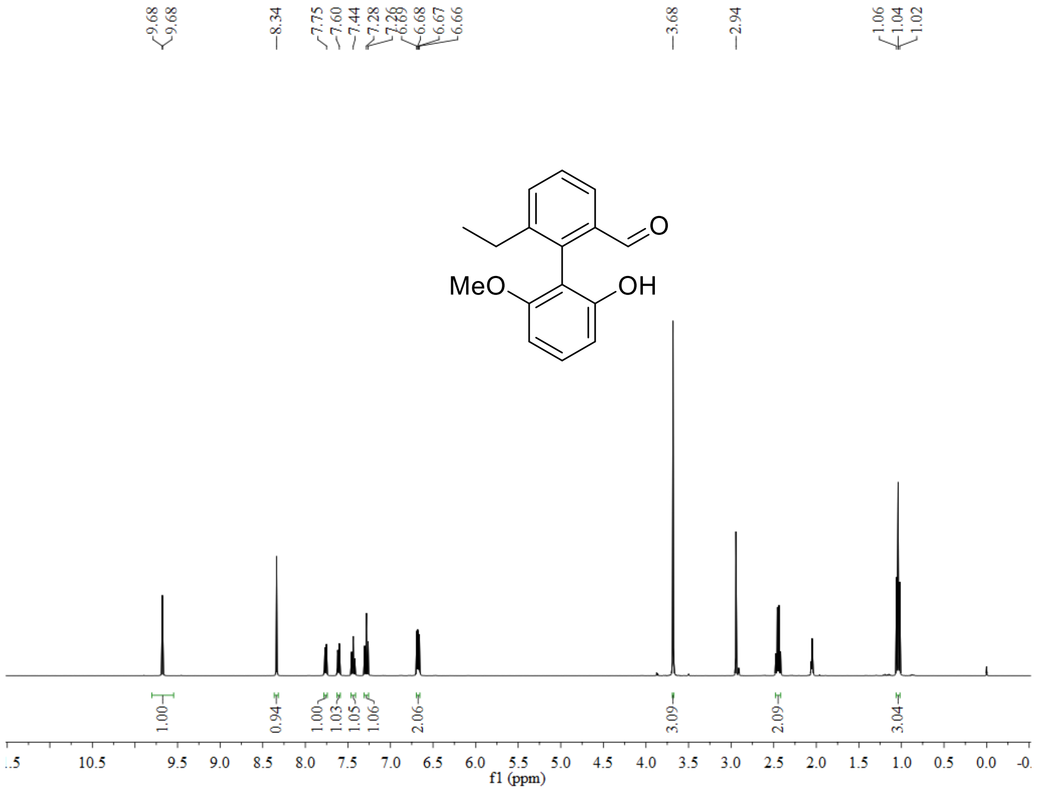

Supplementary Figure 49 ^{13}C NMR (400 MHz, Acetone-d_6) of **1t**

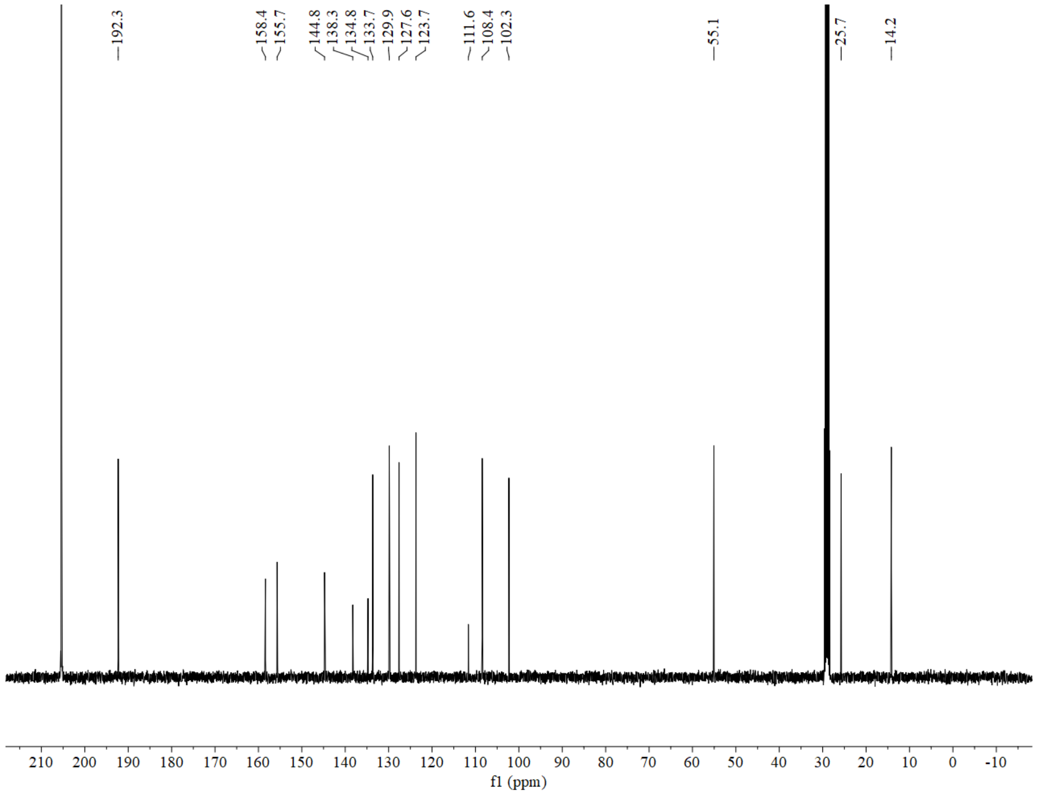

Supplementary Figure 50 ^{1}H NMR (400 MHz, $CDCl_3$) of **1u**

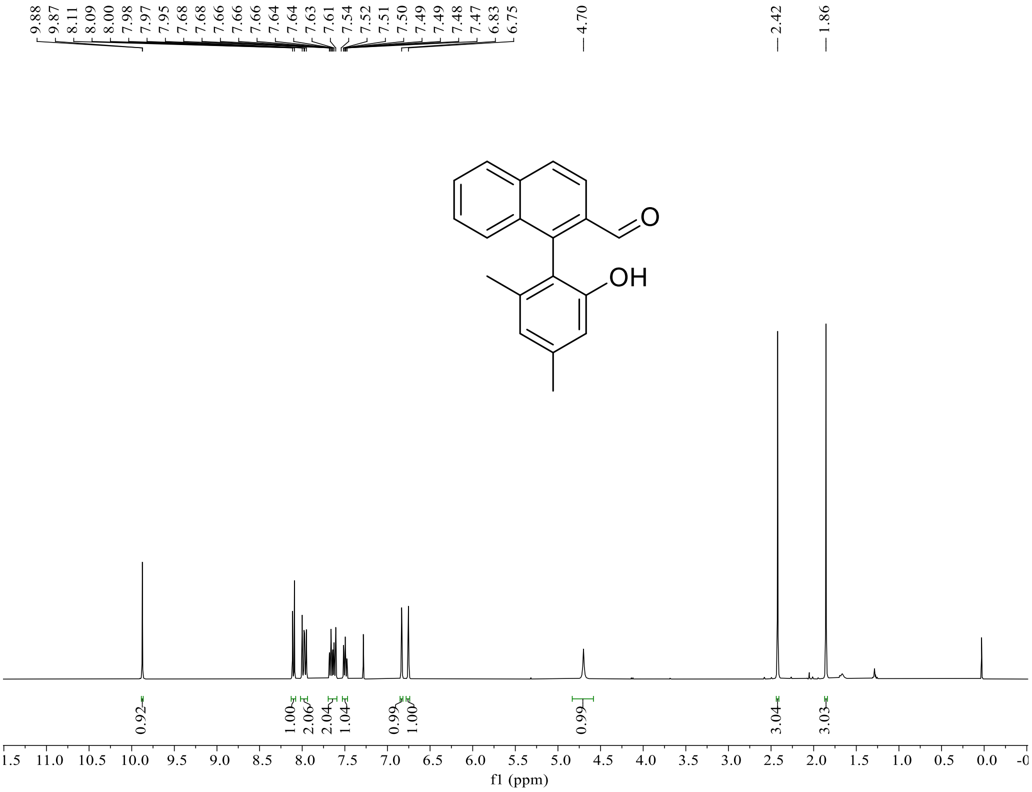

Supplementary Figure 51 ^{13}C NMR (400 MHz, $CDCl_3$) of **1u**

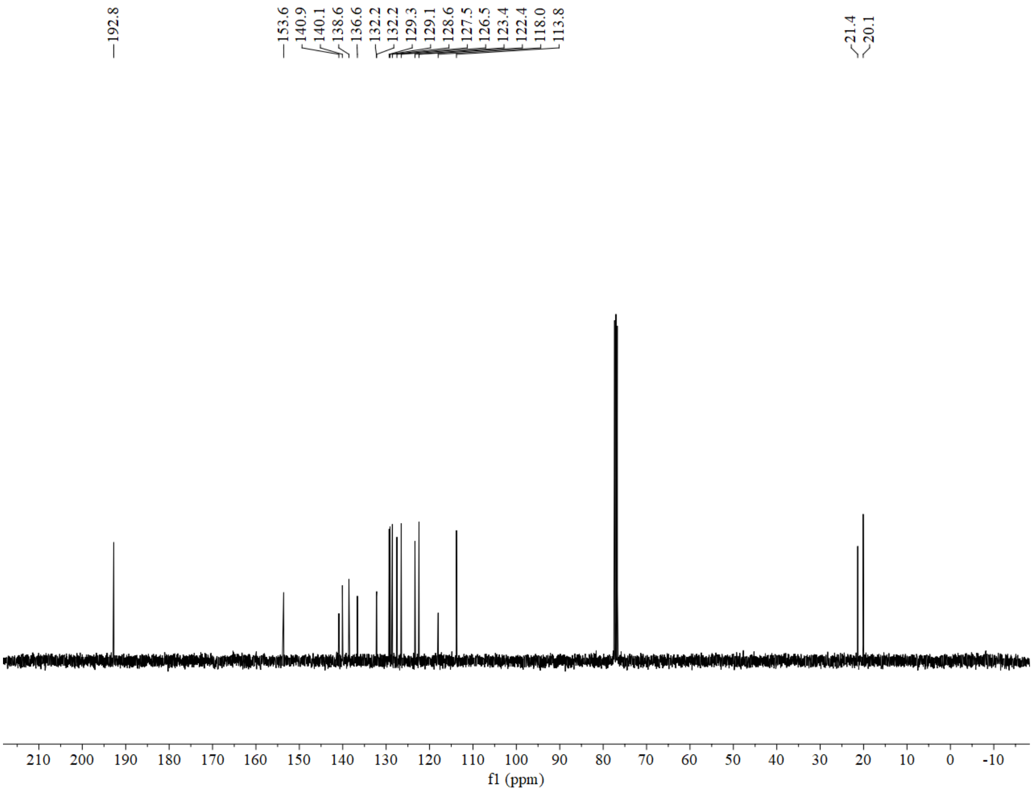

Supplementary Figure 52 ^{1}H NMR (400 MHz, Acetone-d_6) of **1v**

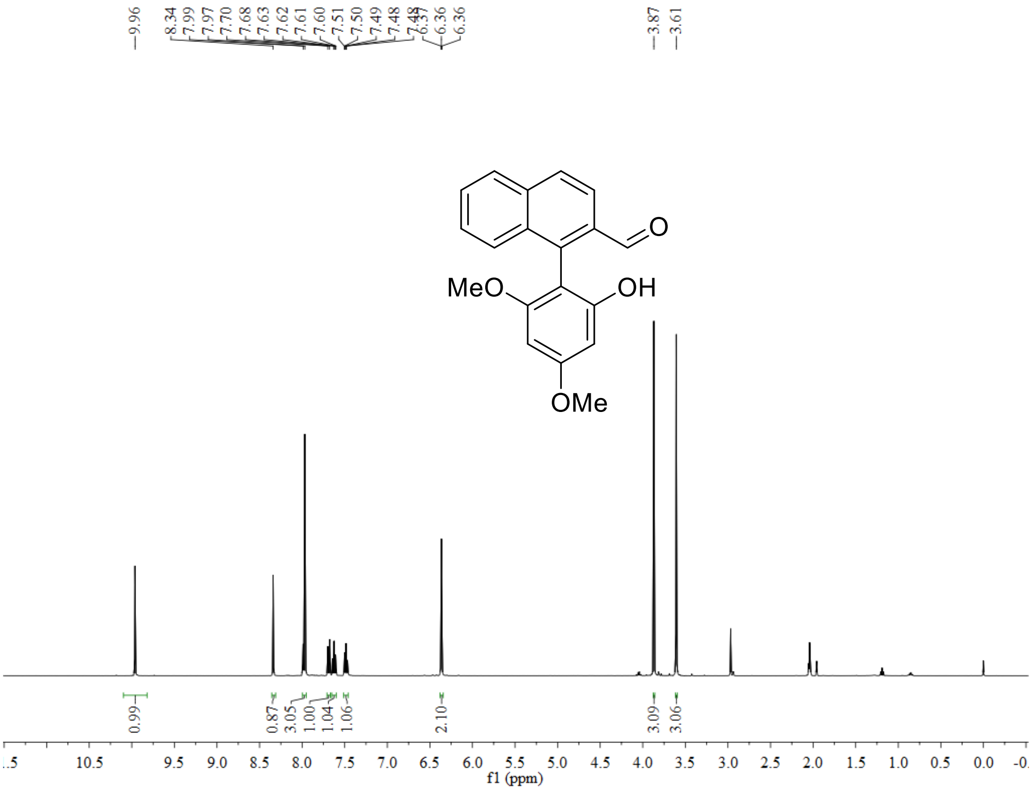

Supplementary Figure 53 ^{13}C NMR (400 MHz, Acetone-d_6) of **1v**

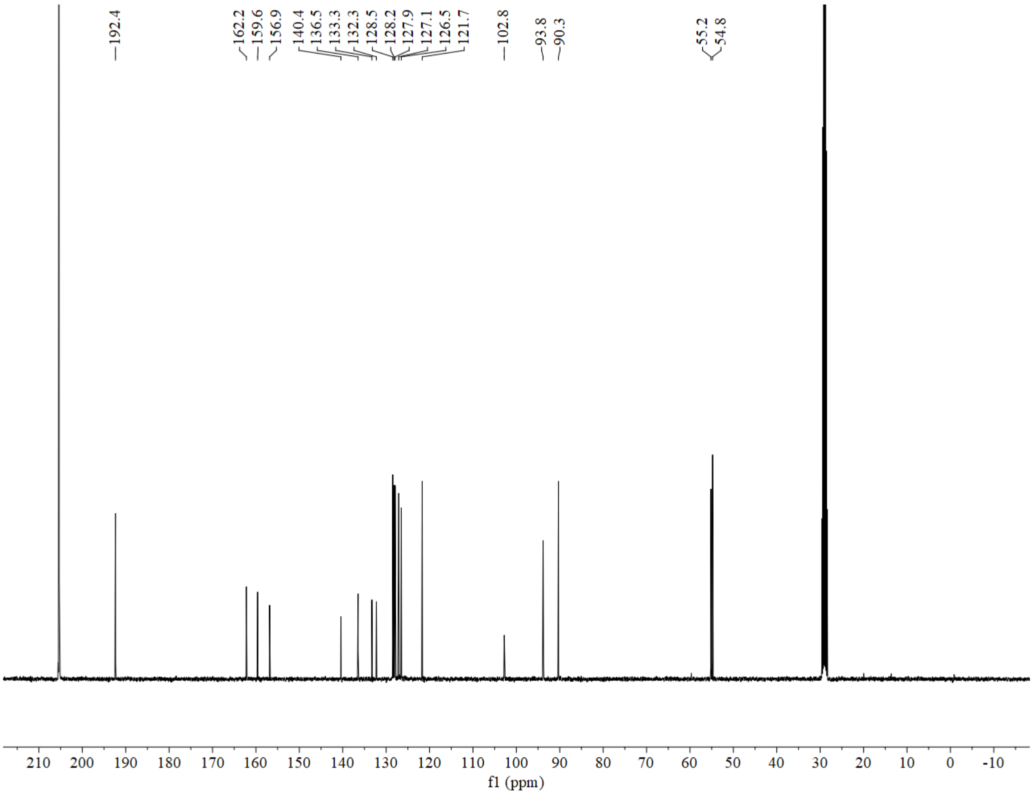

Supplementary Figure 54 ^{1}H NMR (400 MHz, $CDCl_3$) of **1w**

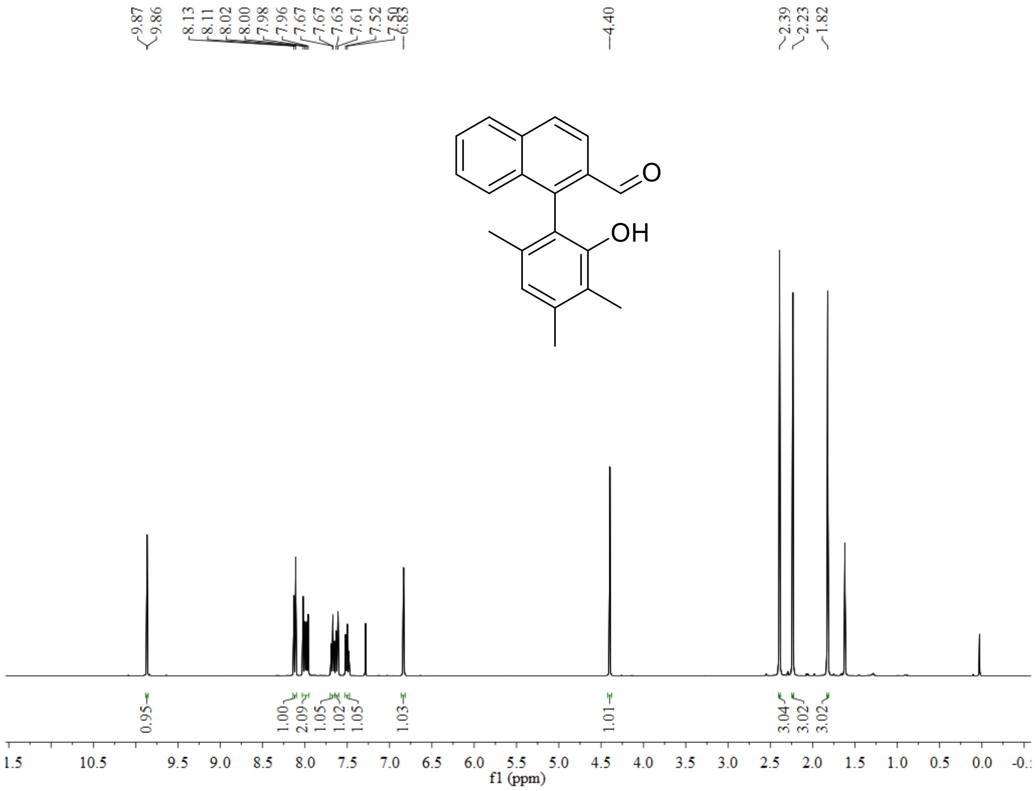

Supplementary Figure 55 ^{13}C NMR (400 MHz, $CDCl_3$) of **1w**

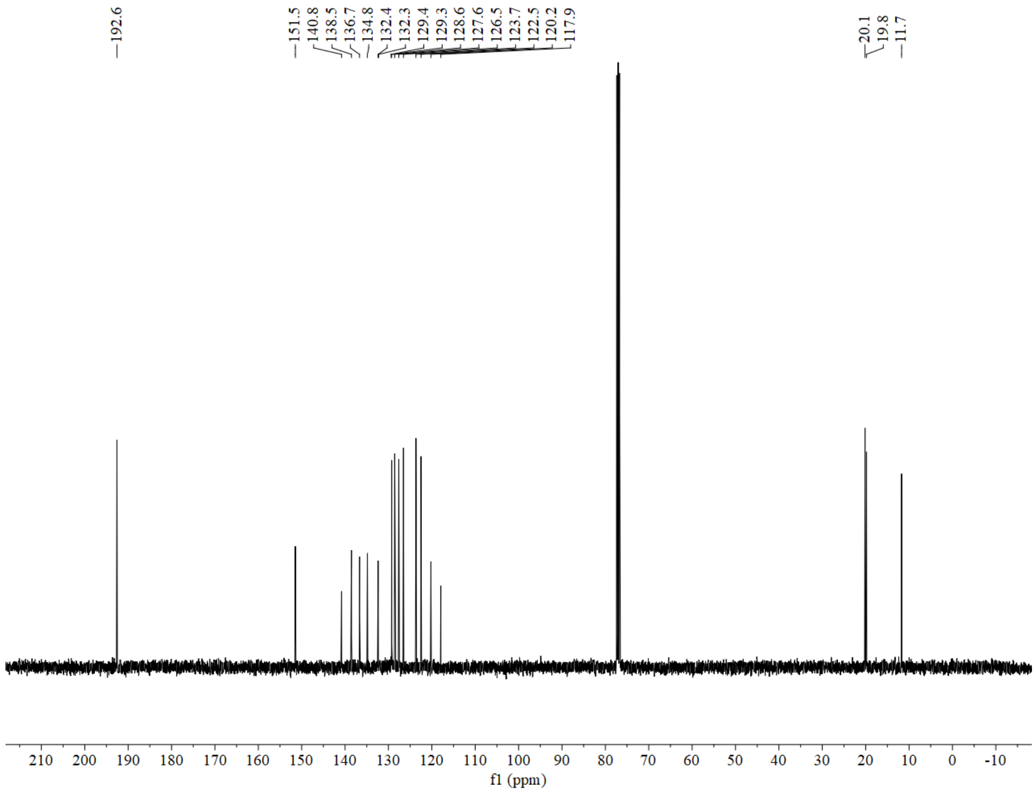

Supplementary Figure 56 ^{1}H NMR (400 MHz, $CDCl_3$) of **1x**

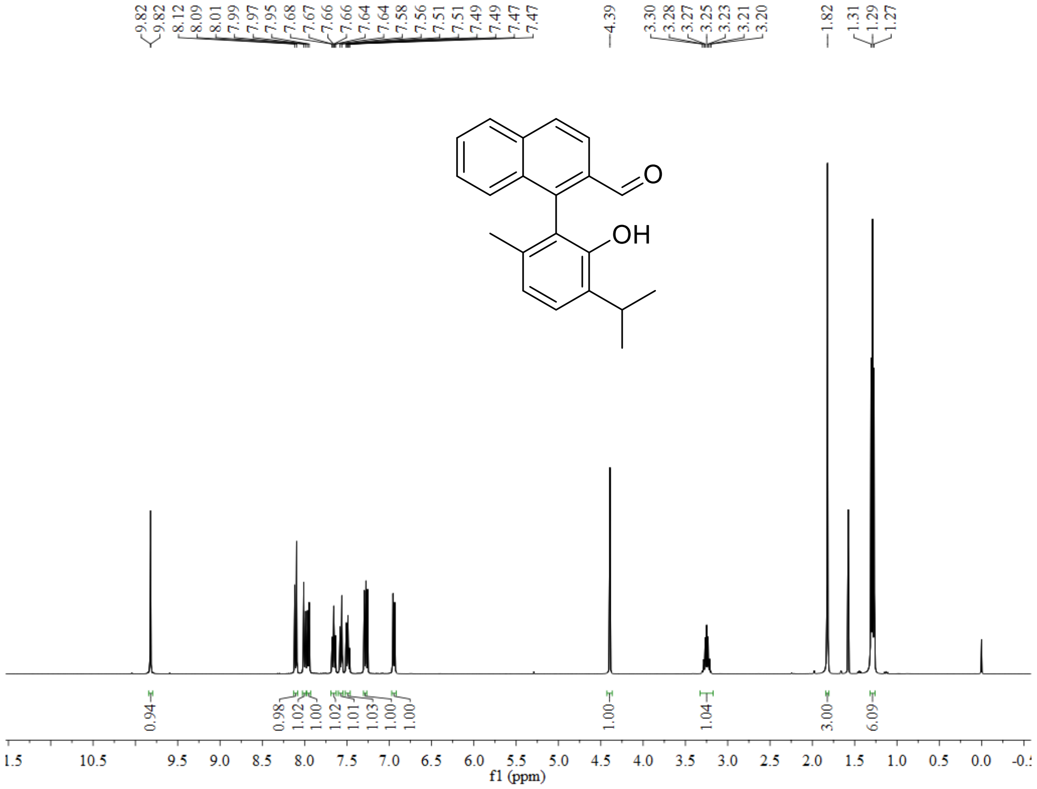

Supplementary Figure 57 ^{13}C NMR (400 MHz, $CDCl_3$) of **1x**

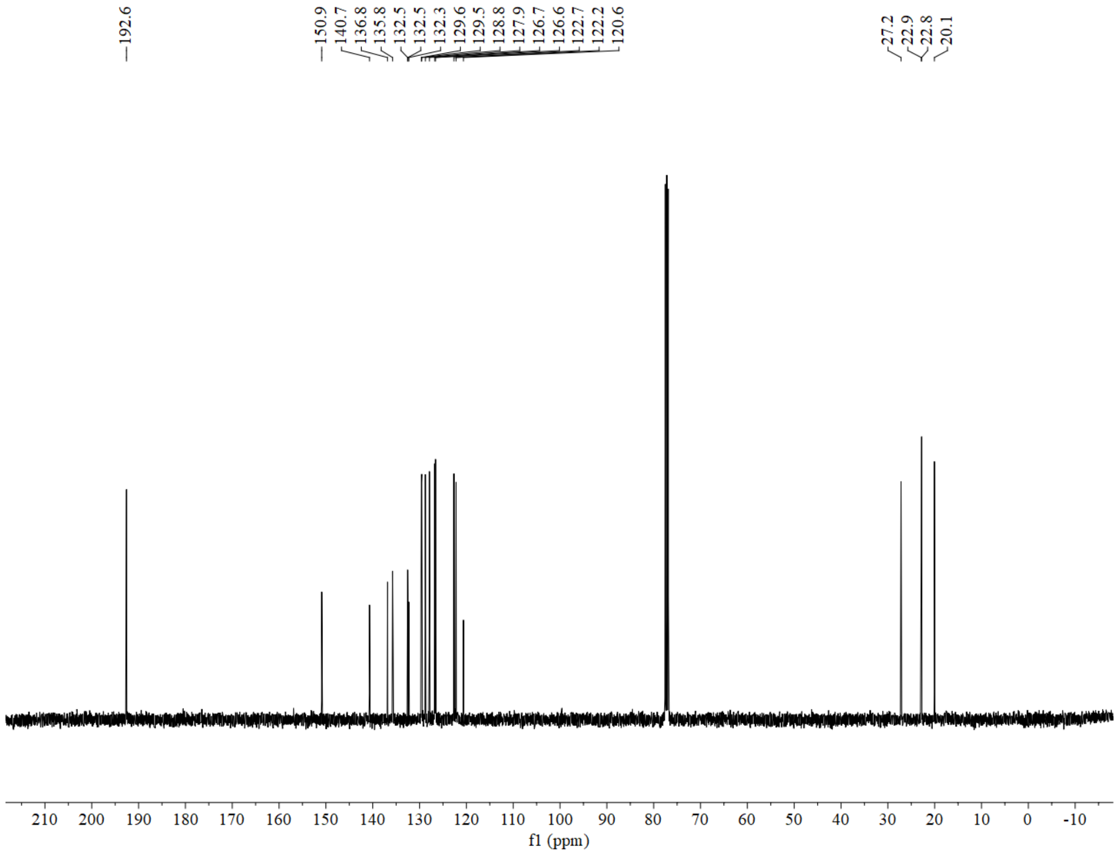

Supplementary Figure 58 ^{1}H NMR (400 MHz, $CDCl_3$) of **1y**

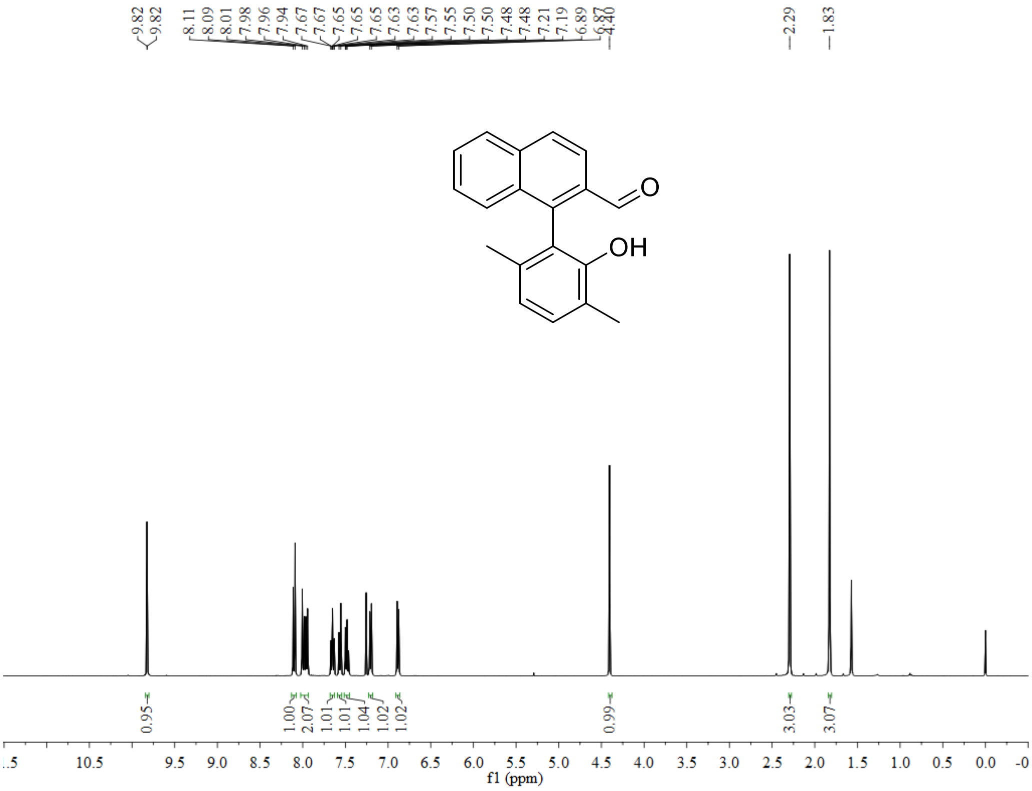

Supplementary Figure 59 ^{13}C NMR (400 MHz, $CDCl_3$) of **1y**

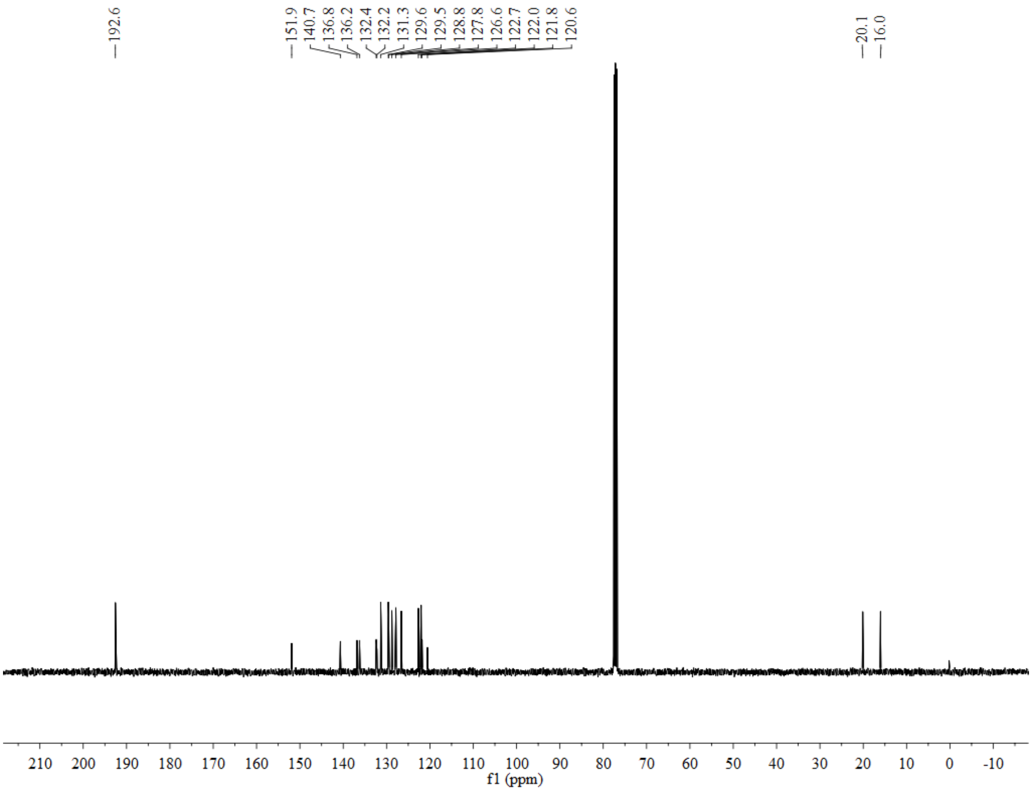

Supplementary Figure 60 ^{1}H NMR (400 MHz, $CDCl_3$) of **1z**

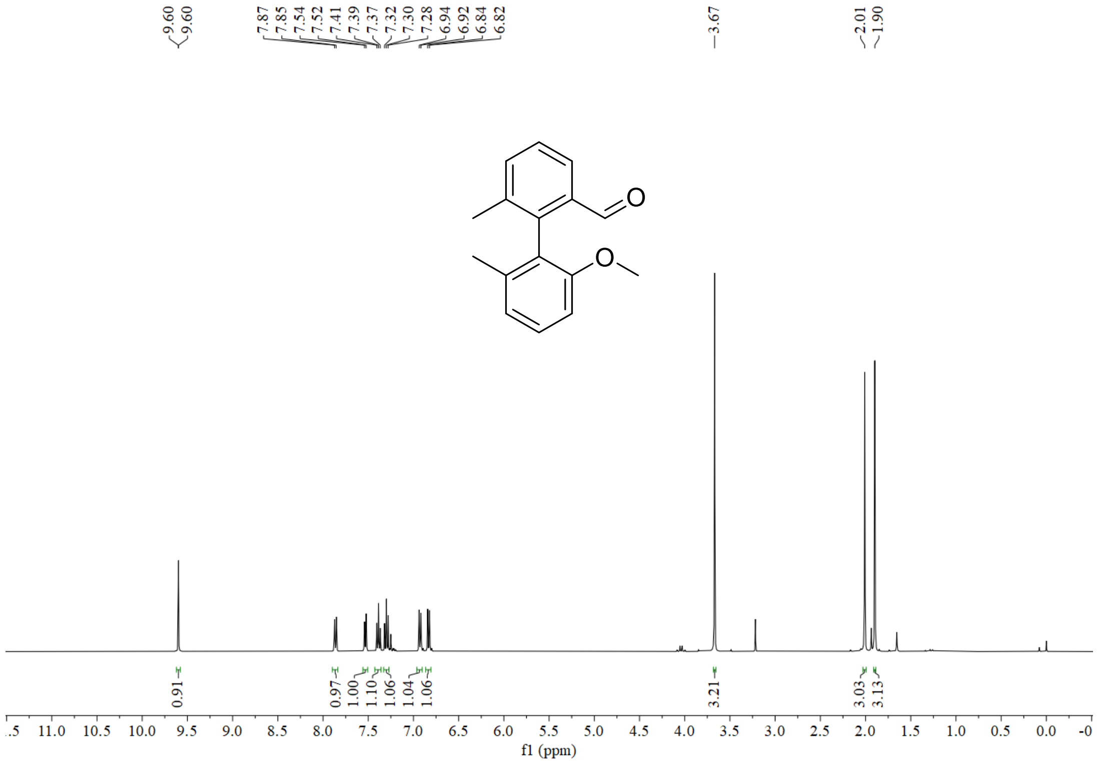

Supplementary Figure 61 ^{13}C NMR (400 MHz, $CDCl_3$) of **1z**

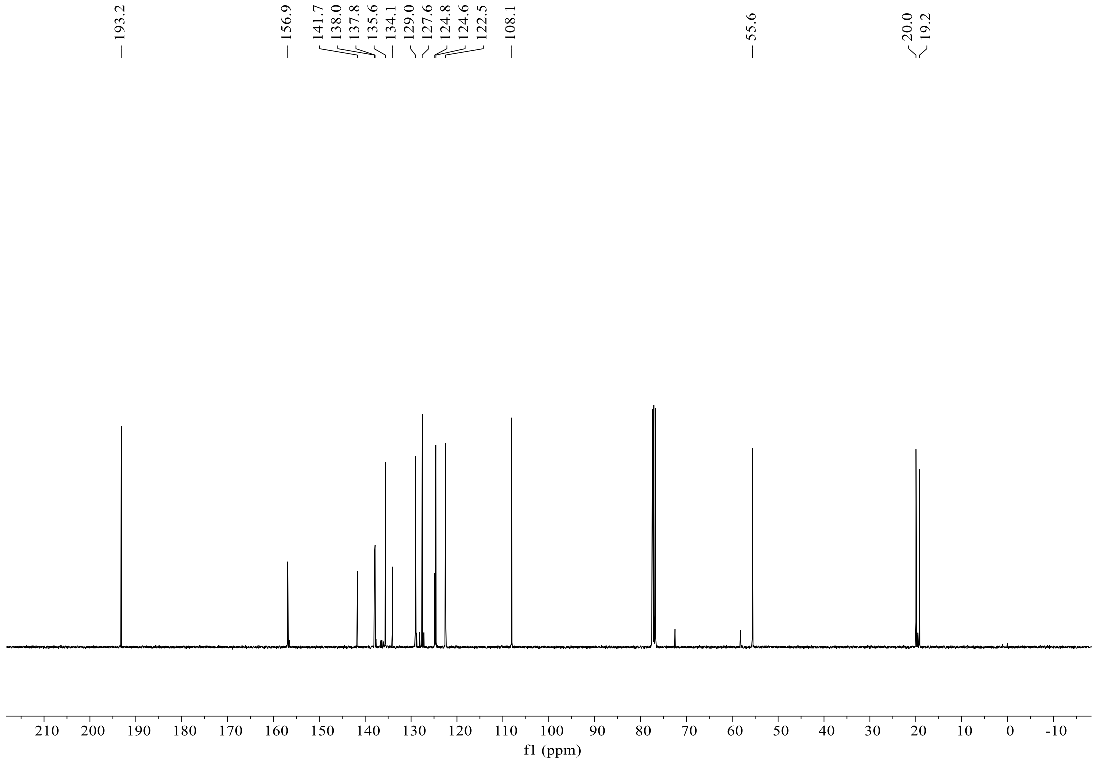

Supplementary Figure 62 ^{1}H NMR (400 MHz, DMSO-d_6) of NHC-**G**

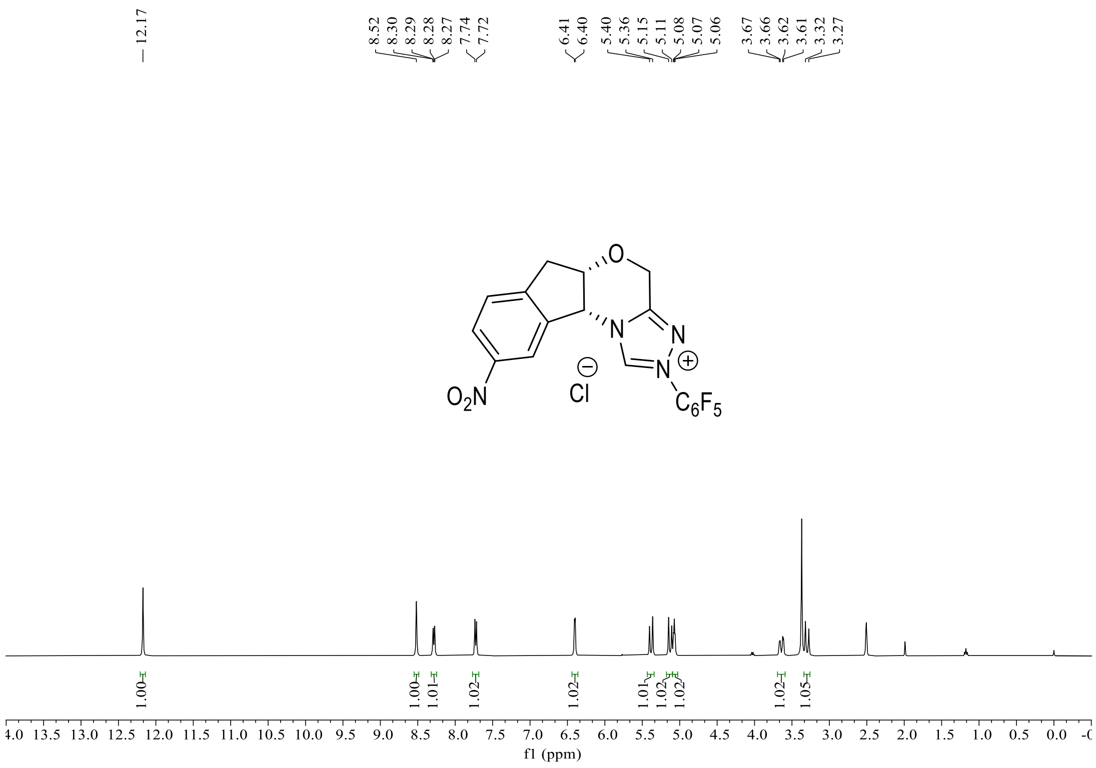

Supplementary Figure 63 ^{13}C NMR (400 MHz, DMSO-d_6) of NHC-**G**

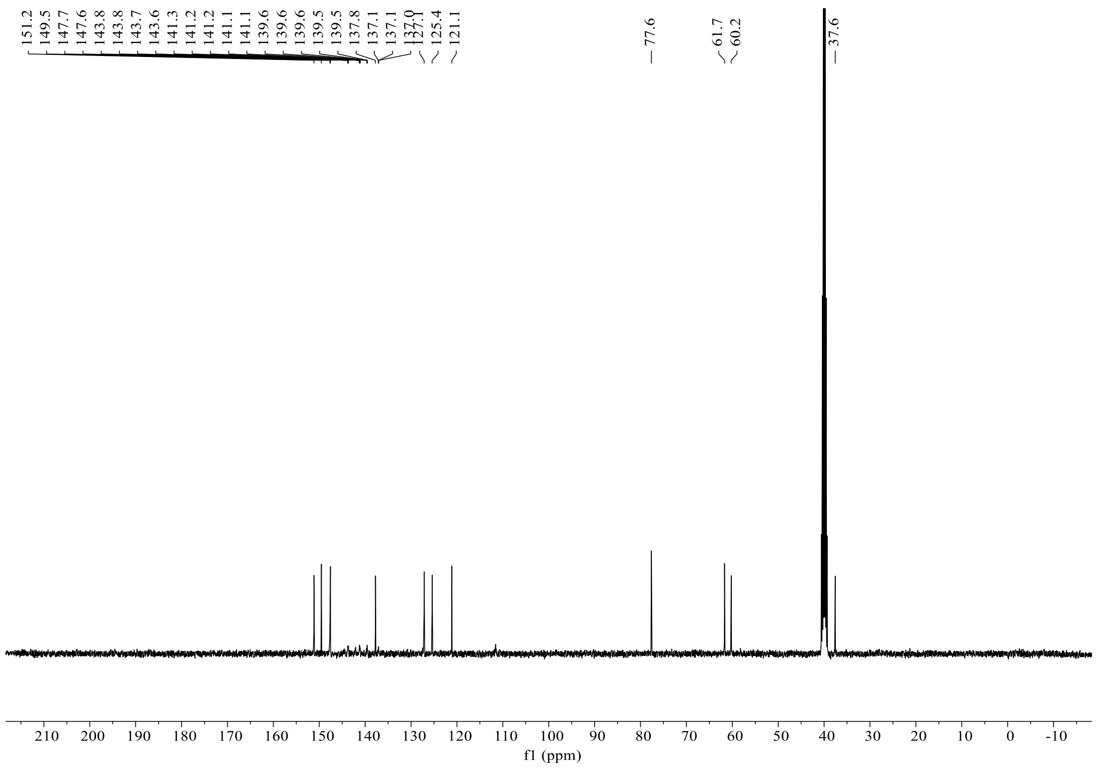

Supplementary Figure 64 ^{19}F NMR (400 MHz, DMSO-d_6) of NHC-**G**

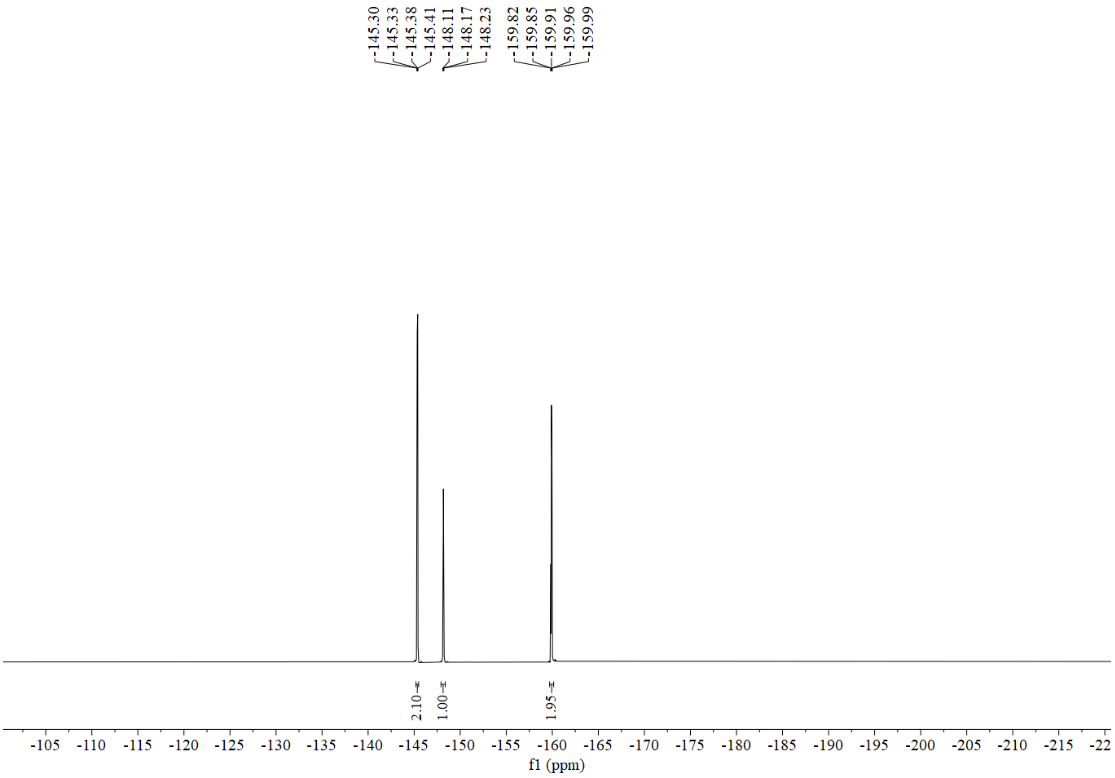

Supplementary Figure 65 ^{1}H NMR (400 MHz, $CDCl_3$) of **3a**

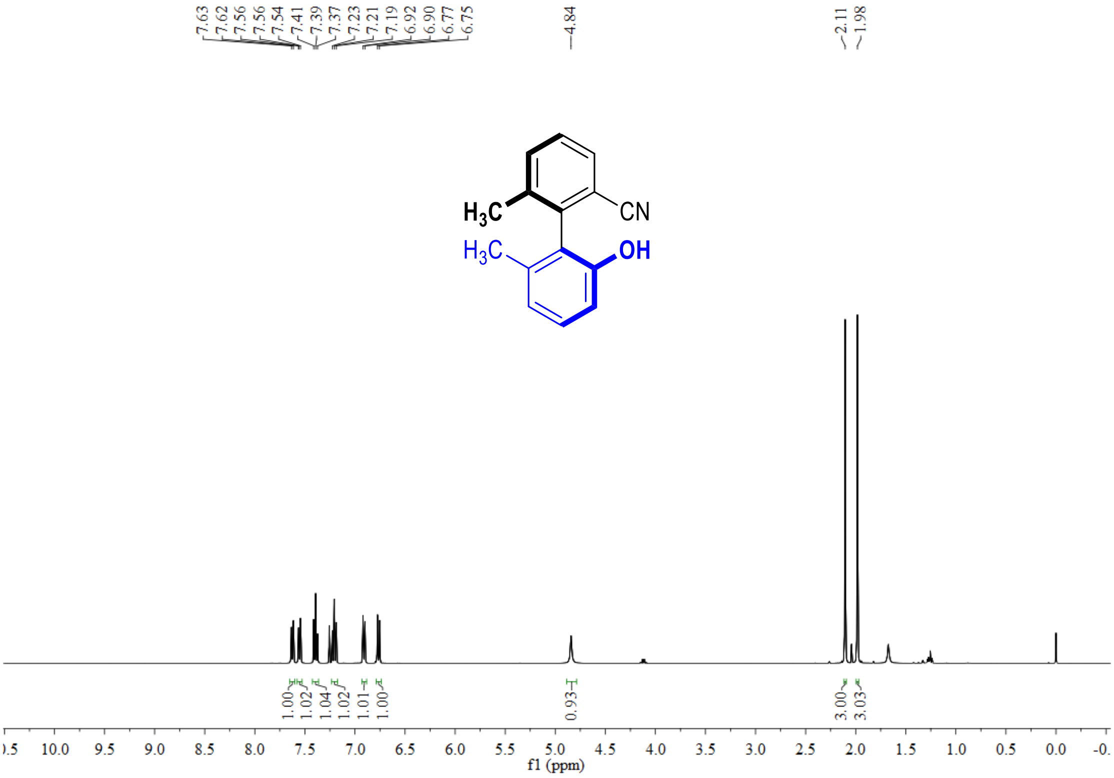

Supplementary Figure 66 ^{13}C NMR (400 MHz, $CDCl_3$) of **3a**

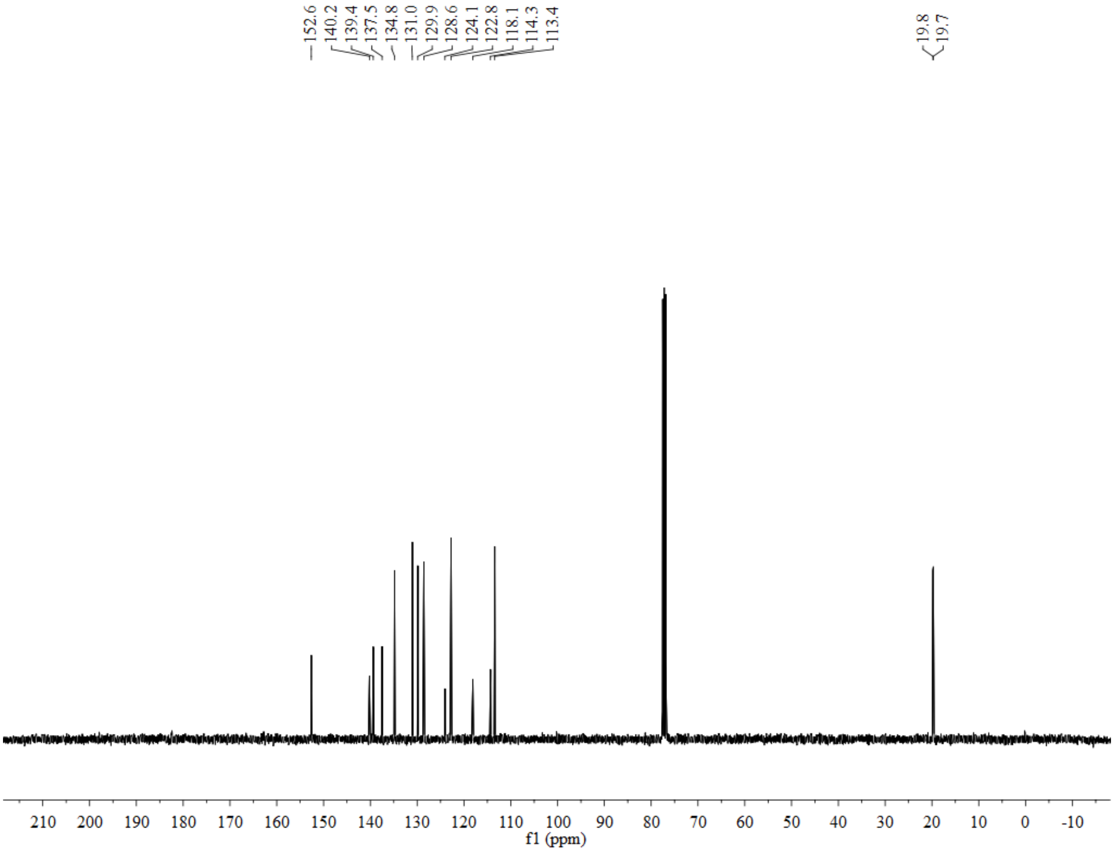

Supplementary Figure 67 HPLC spectra of racemic **3a**

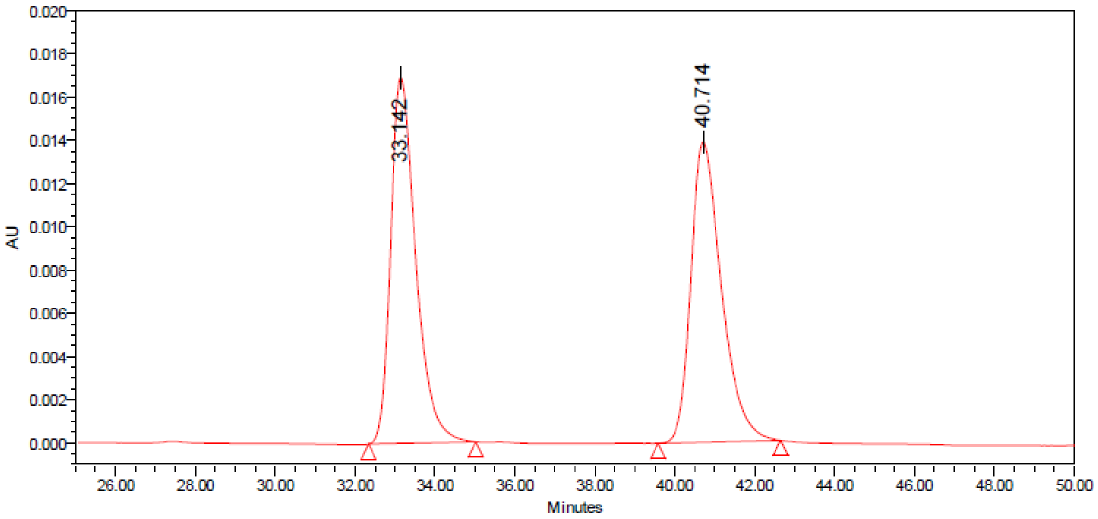

	RT	Area	% Area	Height
1	33.142	740902	49.98	16931
2	40.714	741420	50.02	13887

Supplementary Figure 68 HPLC spectra of (*S*)- **3a**

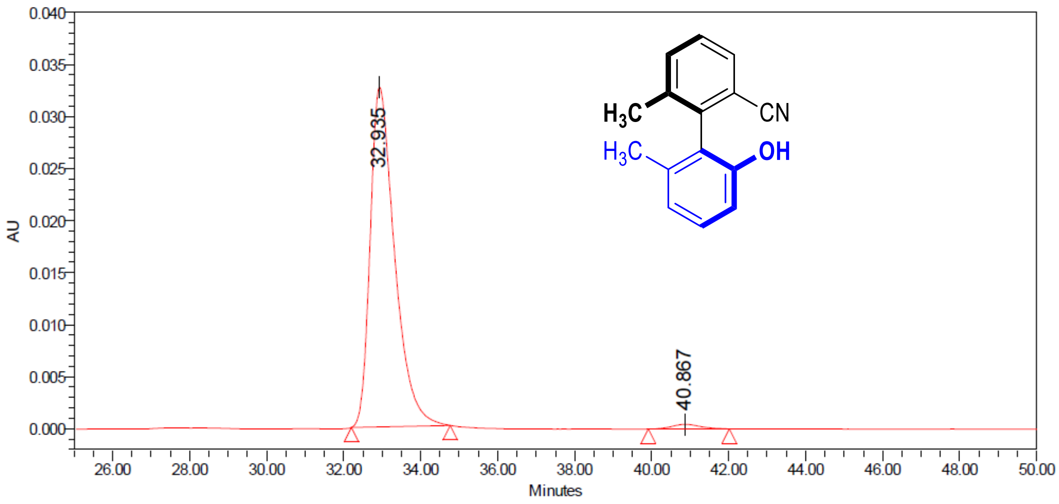

	RT	Area	% Area	Height
1	32.935	1443303	98.51	32648
2	40.867	21893	1.49	433

Supplementary Figure 69 ^{1}H NMR (400 MHz, $CDCl_3$) of **3b**

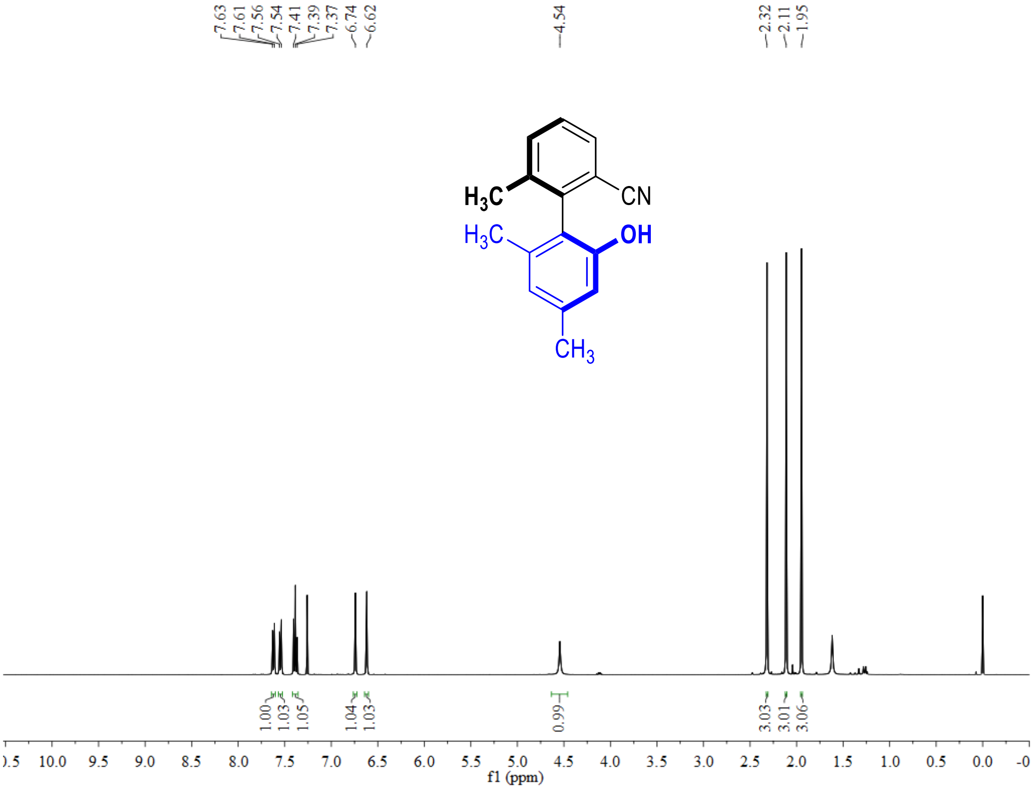

Supplementary Figure 70 ^{13}C NMR (400 MHz, $CDCl_3$) of **3b**

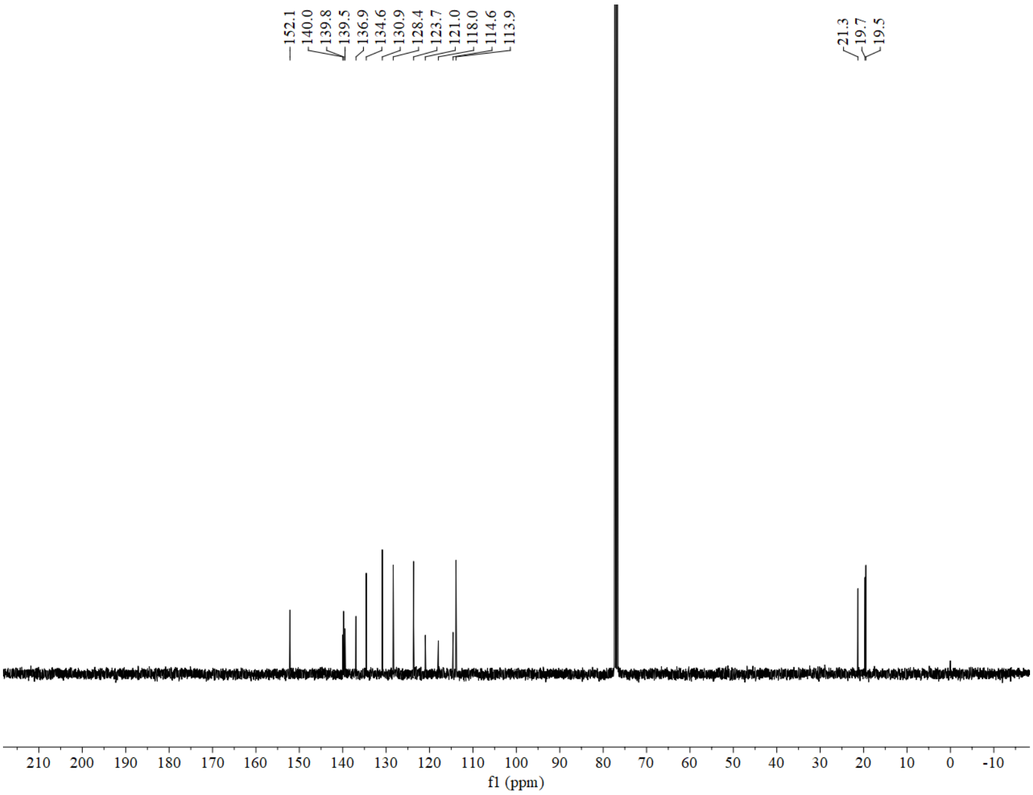

Supplementary Figure 71 HPLC spectra of racemic **3b**

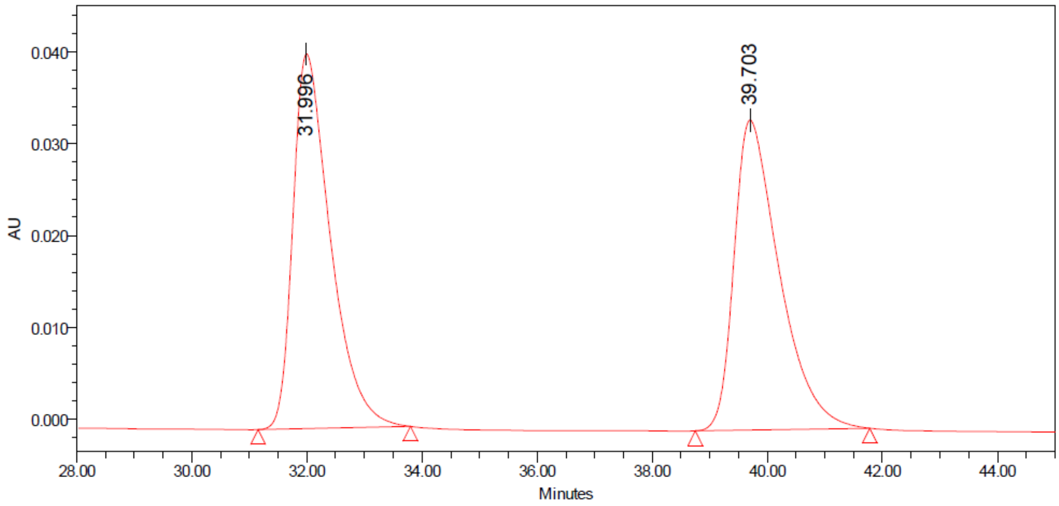

	RT	Area	% Area	Height
1	31.996	1844019	50.00	40759
2	39.703	1843925	50.00	33728

Supplementary Figure 72 HPLC spectra of (*S*)- **3b**

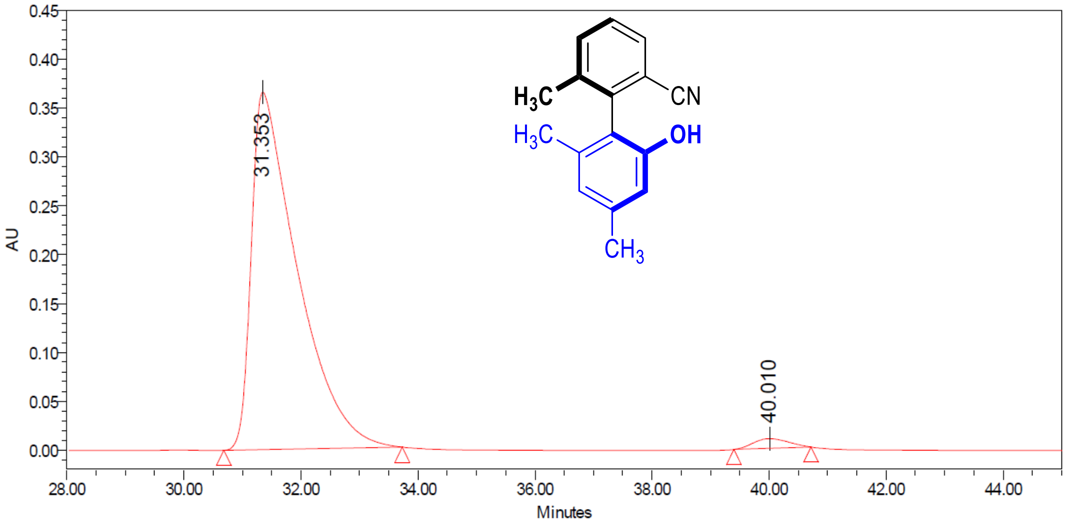

	RT	Area	% Area	Height
1	31.353	19950039	97.96	365865
2	40.010	415451	2.04	9975

Supplementary Figure 73 ^{1}H NMR (400 MHz, $CDCl_3$) of **3c**

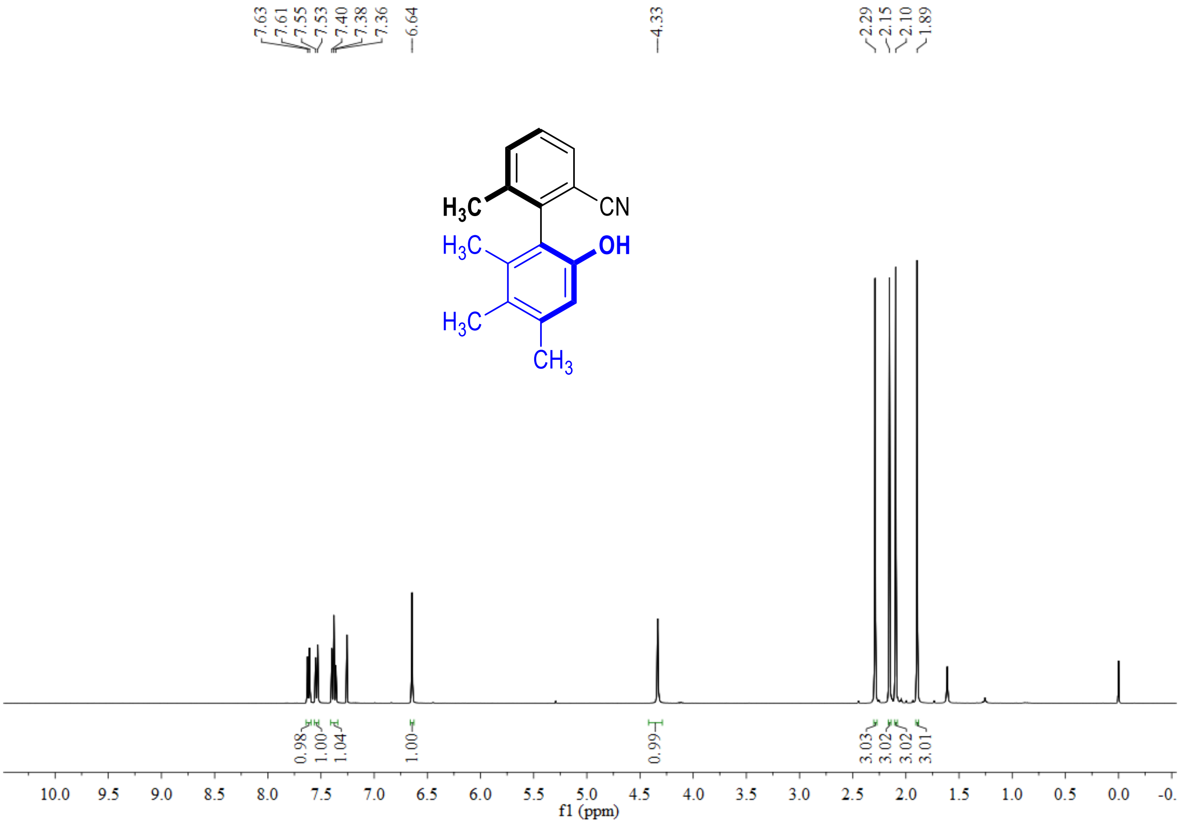

Supplementary Figure 74 ^{13}C NMR (400 MHz, $CDCl_3$) of **3c**

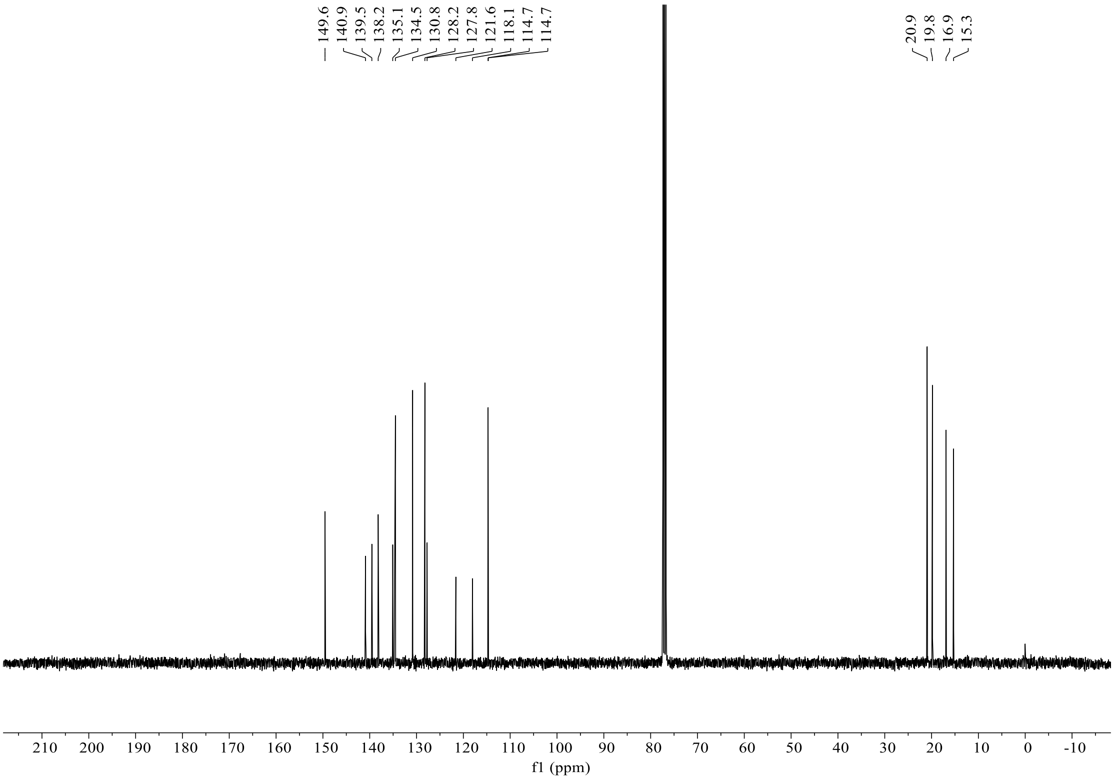

Supplementary Figure 75 HPLC spectra of racemic **3c**

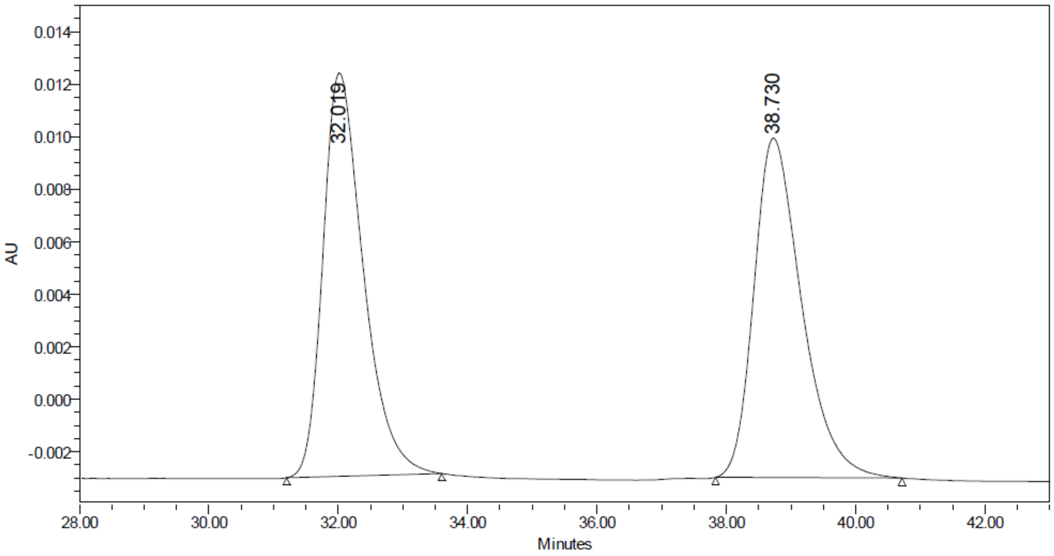

	RT	Area	% Area	Height
1	32.019	666307	50.07	15367
2	38.730	664569	49.93	12930

Supplementary Figure 76 HPLC spectra of (*S*)- **3c**

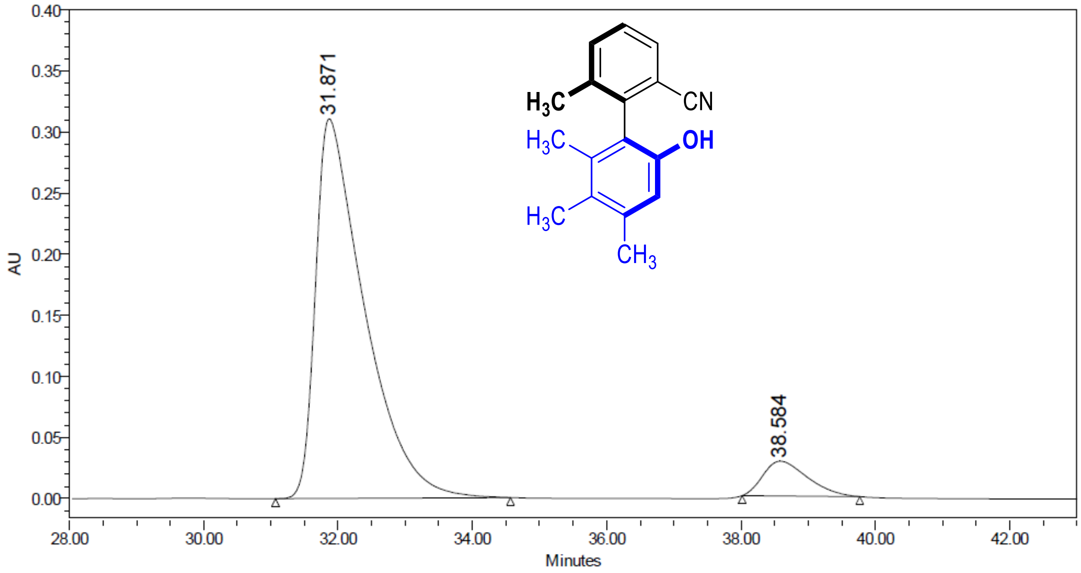

	RT	Area	% Area	Height
1	31.871	15742403	92.29	310796
2	38.584	1314778	7.71	28448

Supplementary Figure 77 ^{1}H NMR (400 MHz, $CDCl_3$) of **3d**

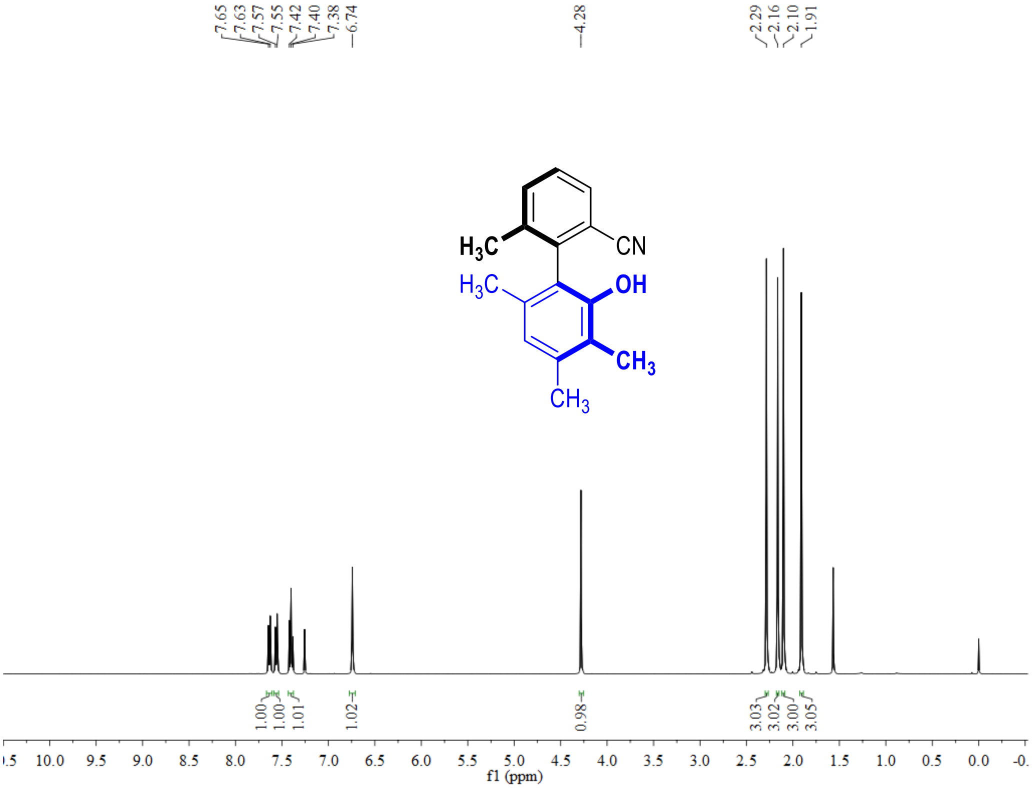

Supplementary Figure 78 ^{13}C NMR (400 MHz, $CDCl_3$) of **3d**

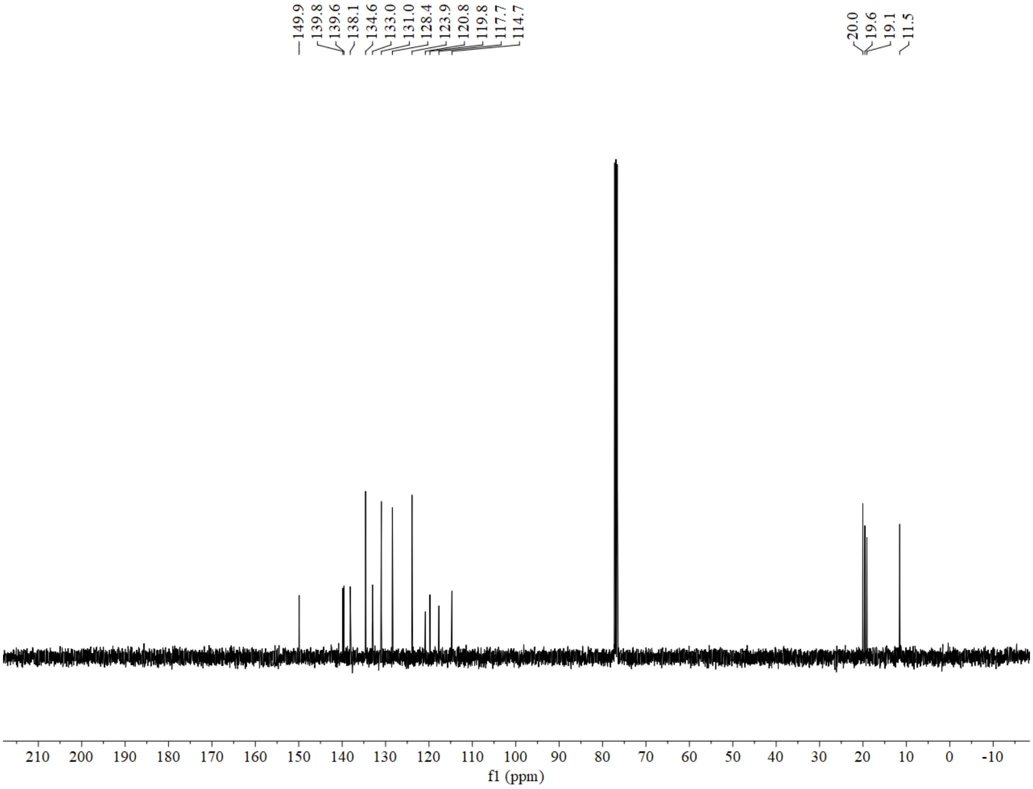

Supplementary Figure 79 HPLC spectra of racemic **3d**

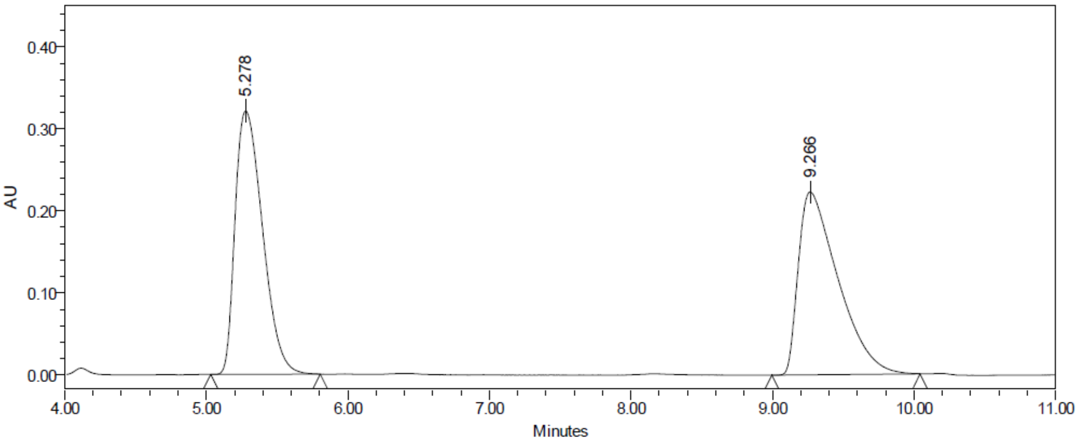

	RT	Area	% Area	Height
1	5.278	4334651	49.98	321012
2	9.266	4338978	50.02	222819

Supplementary Figure 80 HPLC spectra of (*S*)- **3d**

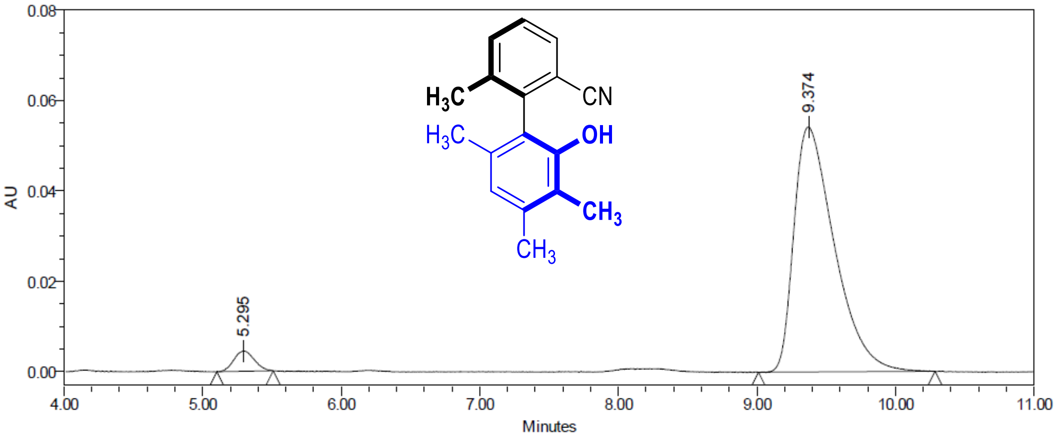

	RT	Area	% Area	Height
1	5.295	45796	4.00	4504
2	9.374	1100407	96.00	54160

Supplementary Figure 81 ^{1}H NMR (400 MHz, Acetone-d_6) of **3e**

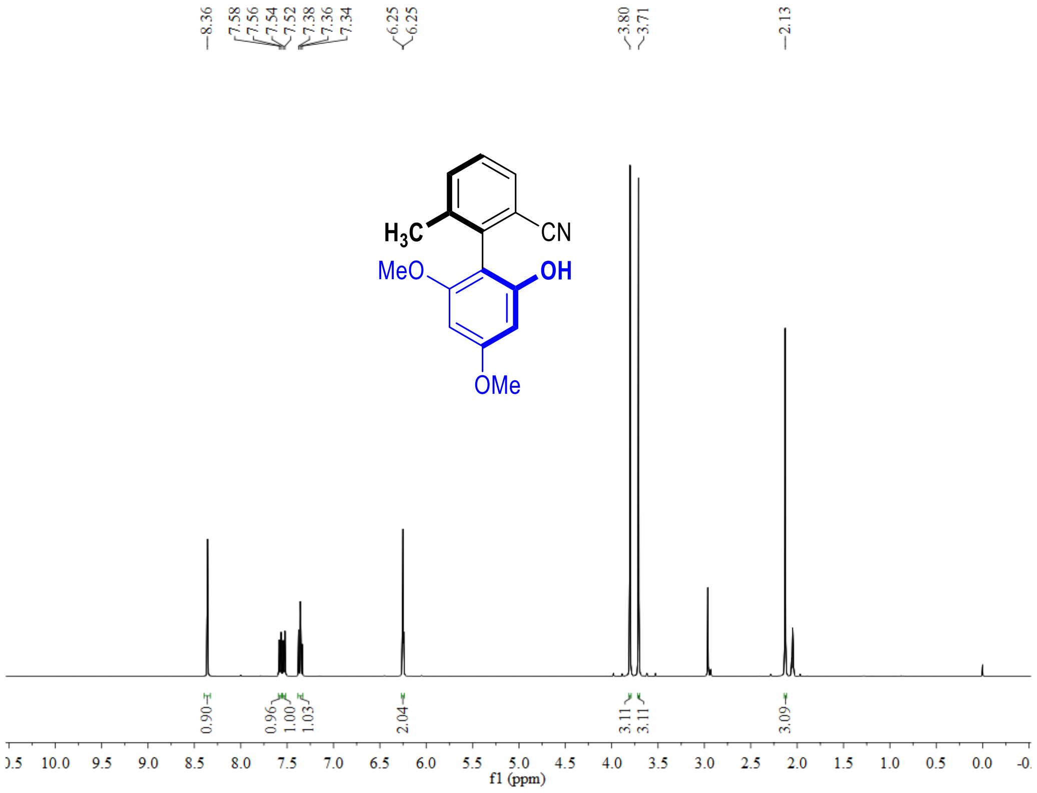

Supplementary Figure 82 ^{13}C NMR (400 MHz, Acetone-d_6) of **3e**

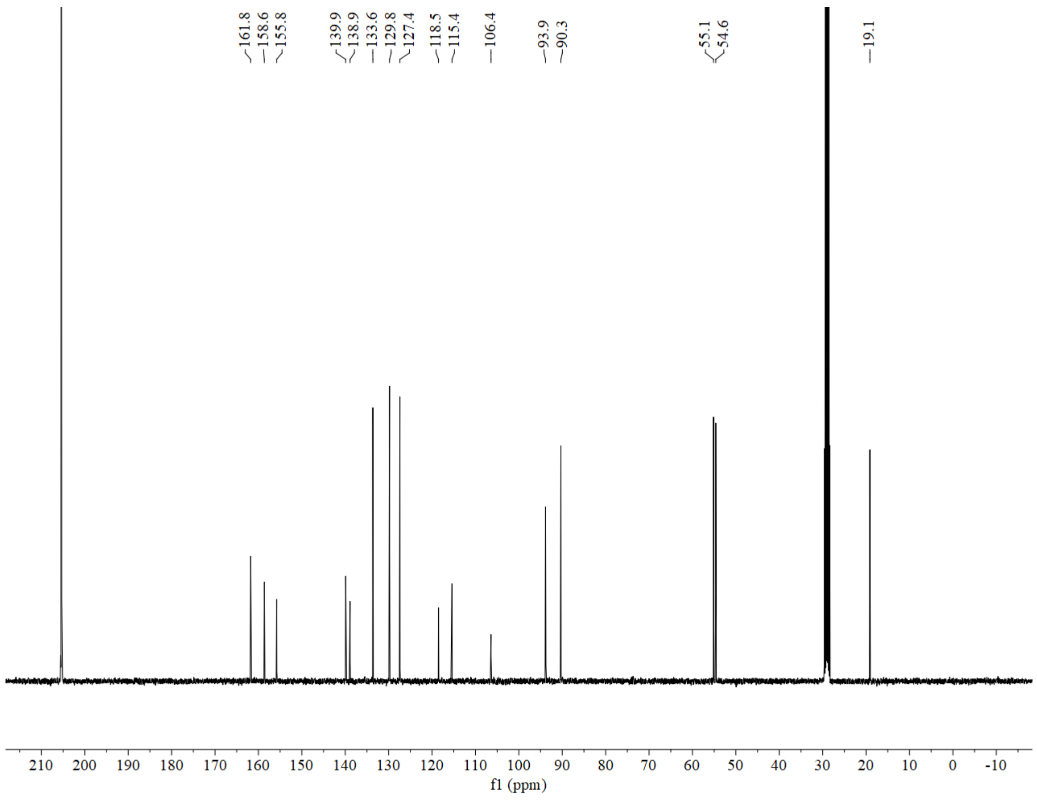

Supplementary Figure 83 HPLC spectra of racemic **3e**

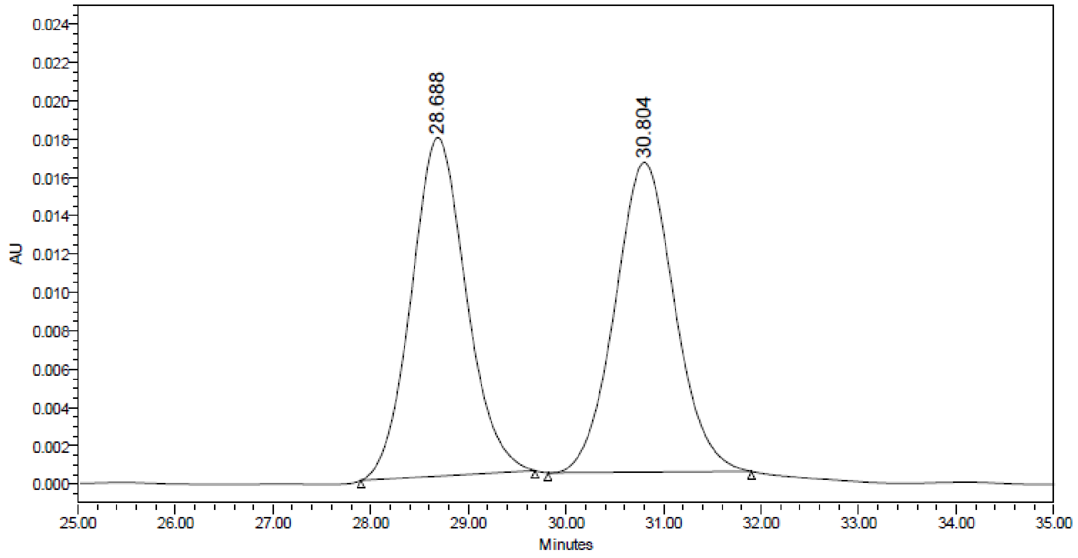

	RT	Area	% Area	Height
1	28.688	690958	50.14	17661
2	30.804	687204	49.86	16172

Supplementary Figure 84 HPLC spectra of (*R*)- **3e**

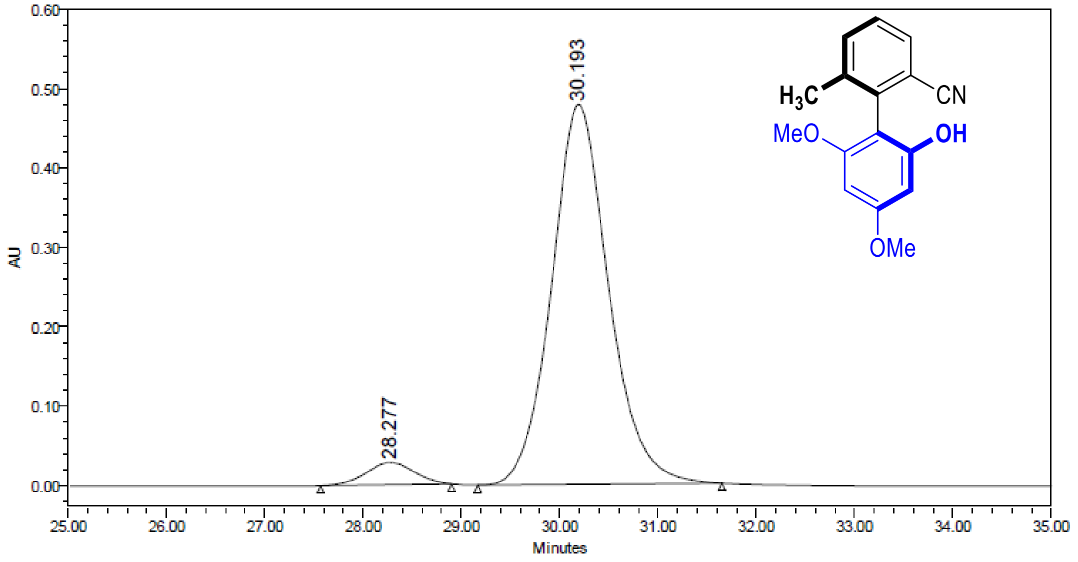

	RT	Area	% Area	Height
1	28.277	953463	4.70	28041
2	30.193	19315480	95.30	478442

Supplementary Figure 85 ^{1}H NMR (400 MHz, $CDCl_3$) of **3f**

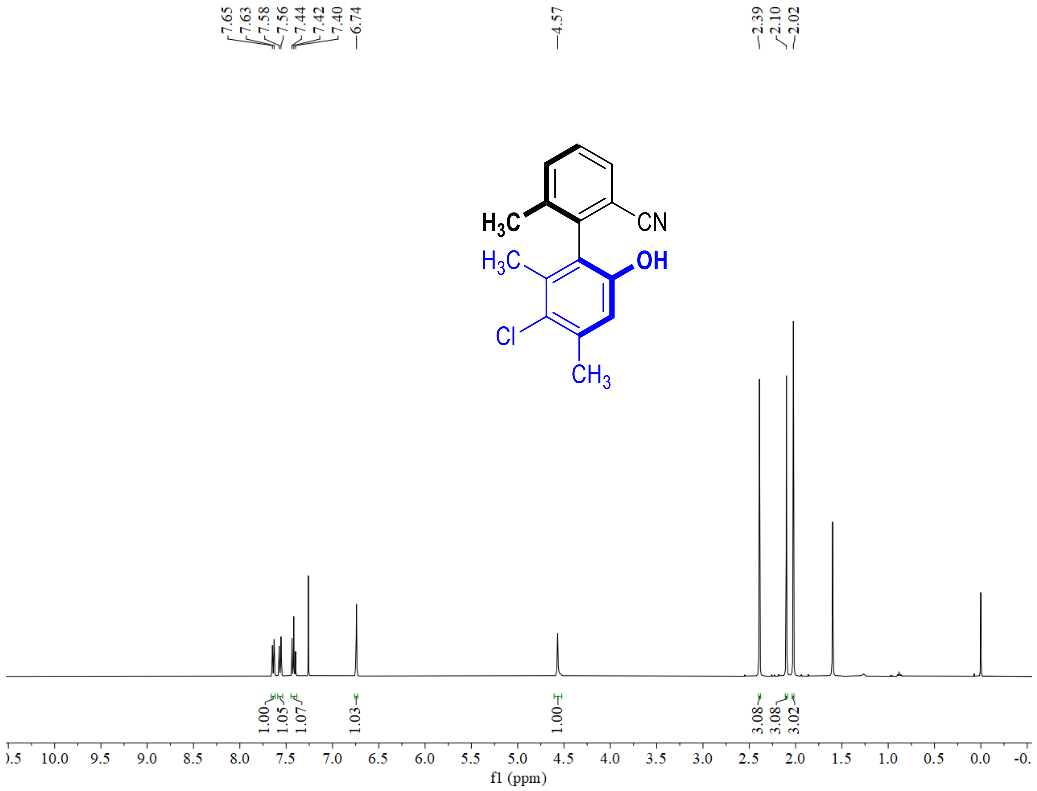

Supplementary Figure 86 ^{13}C NMR (400 MHz, $CDCl_3$) of **3f**

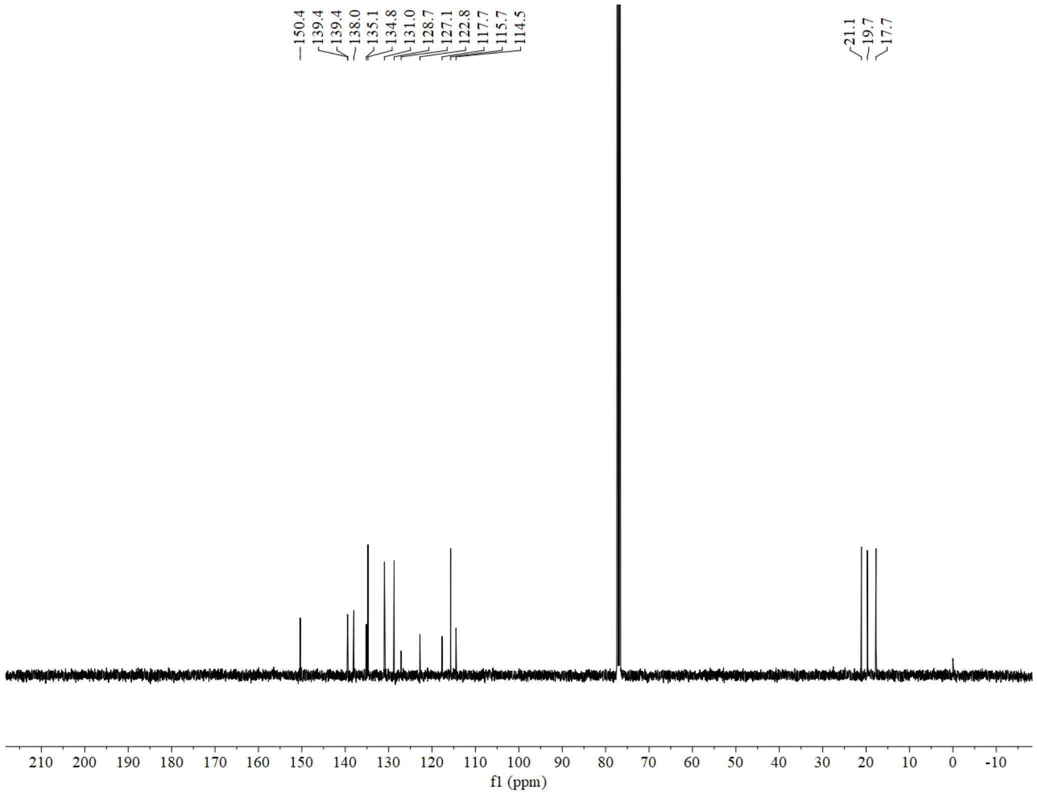

Supplementary Figure 87 HPLC spectra of racemic **3f**

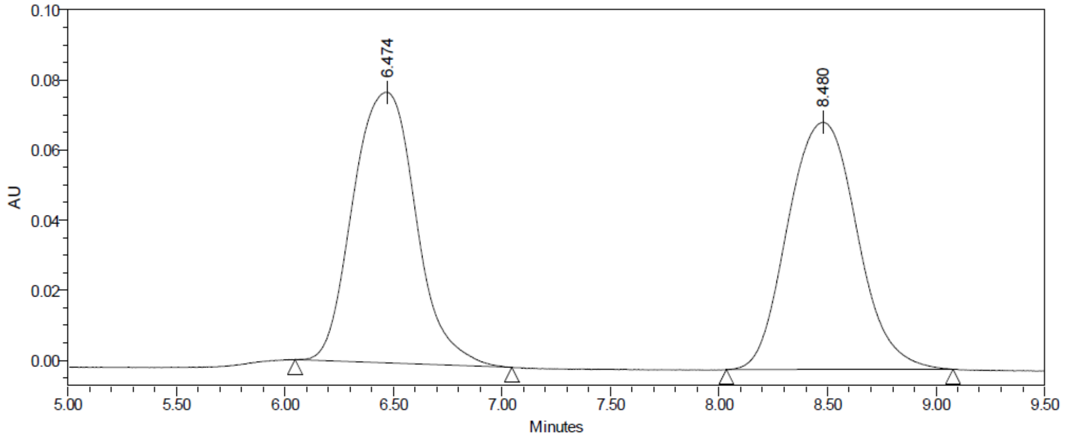

	RT	Area	% Area	Height
1	6.474	1558700	49.88	77203
2	8.480	1566083	50.12	70515

Supplementary Figure 88 HPLC spectra of (*S*)- **3f**

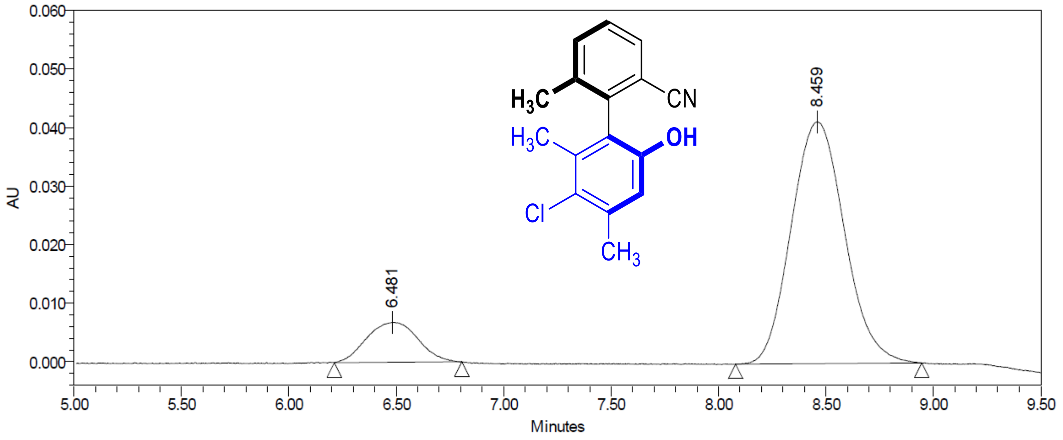

	RT	Area	% Area	Height
1	6.481	112746	13.65	6813
2	8.459	713047	86.35	41318

Supplementary Figure 89 ^{1}H NMR (400 MHz, $CDCl_3$) of **3g**

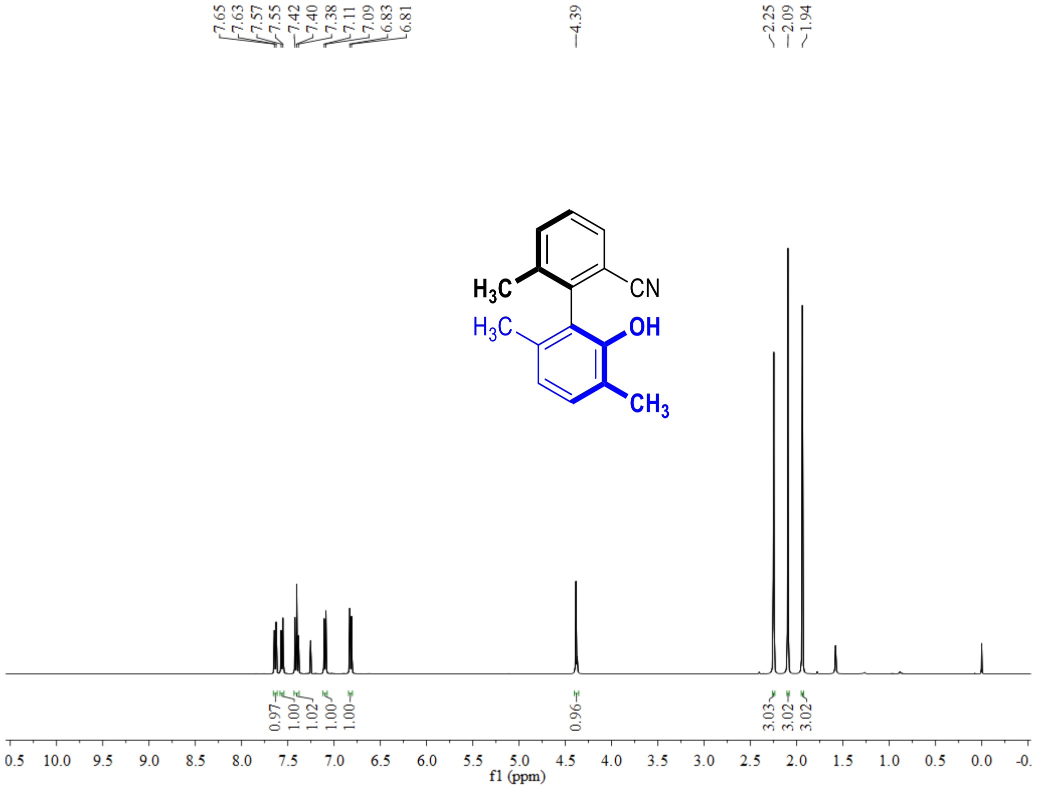

Supplementary Figure 90 ^{13}C NMR (400 MHz, $CDCl_3$) of **3g**

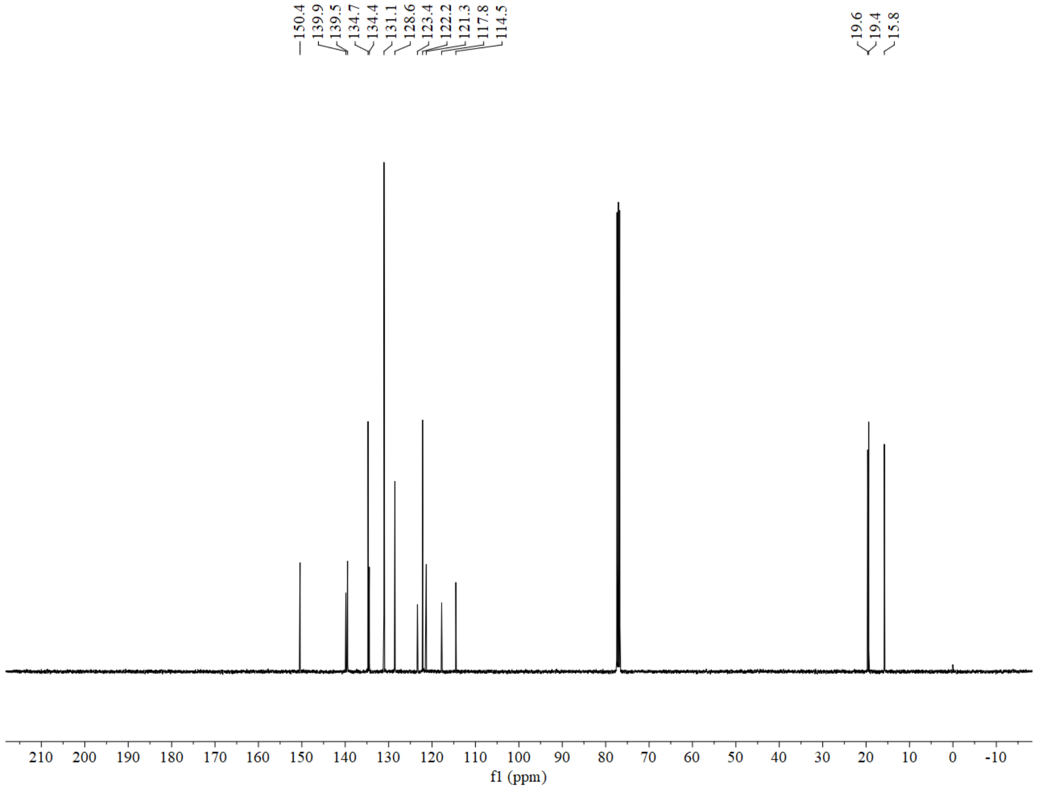

Supplementary Figure 91 HPLC spectra of racemic **3g**

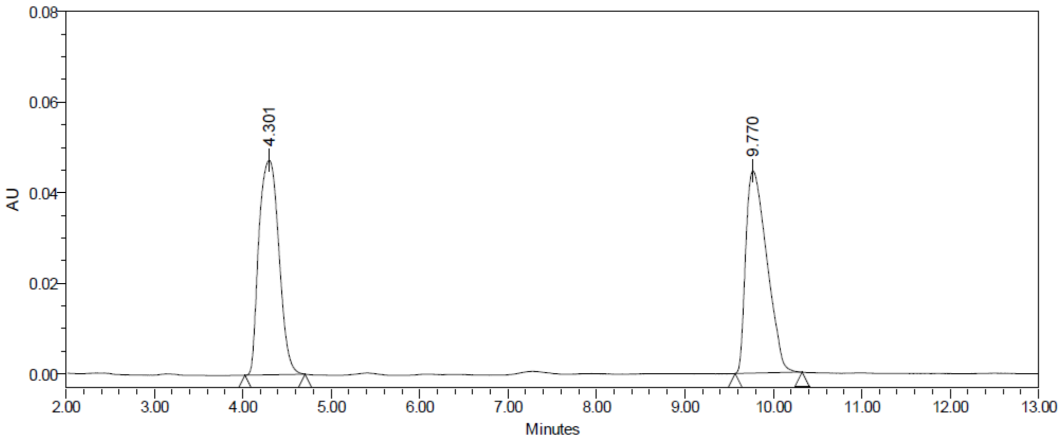

	RT	Area	% Area	Height
1	4.301	745669	50.19	47352
2	9.770	739943	49.81	44660

Supplementary Figure 92 HPLC spectra of (*S*)- **3g**

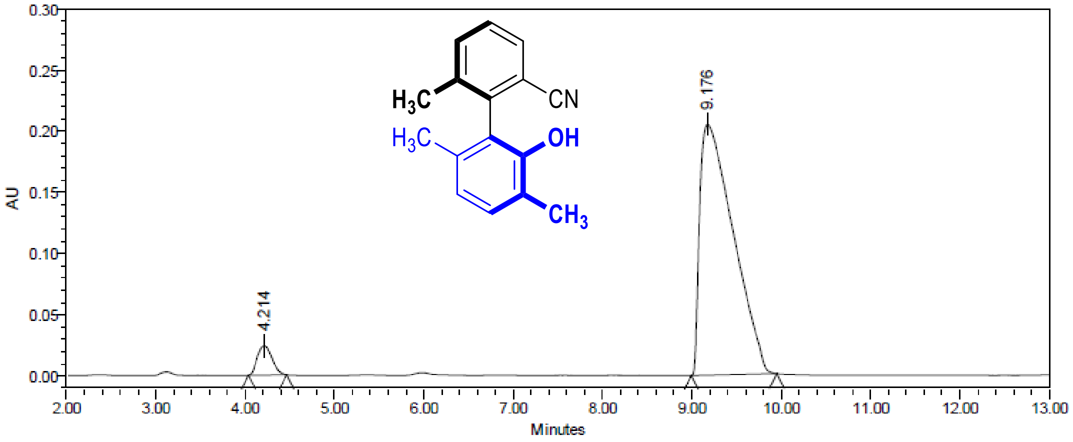

	RT	Area	% Area	Height
1	4.214	272548	4.85	23936
2	9.176	5342170	95.15	204854

Supplementary Figure 93 ^{1}H NMR (400 MHz, $CDCl_3$) of **3h**

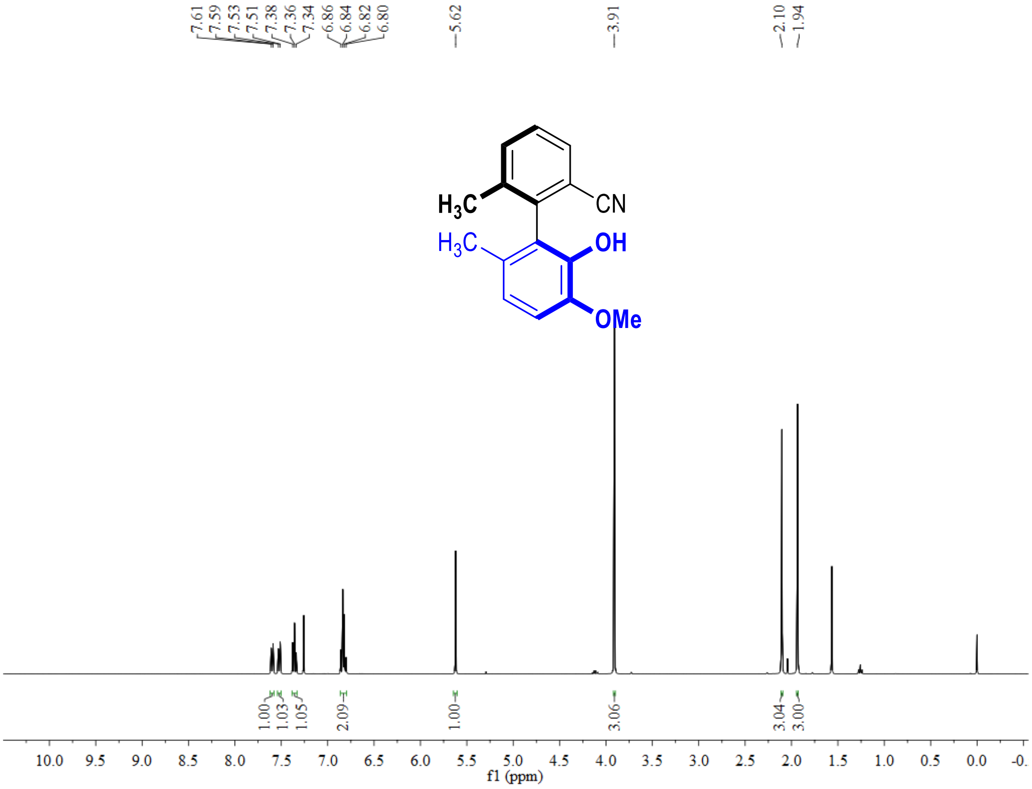

Supplementary Figure 94 ^{13}C NMR (400 MHz, $CDCl_3$) of **3h**

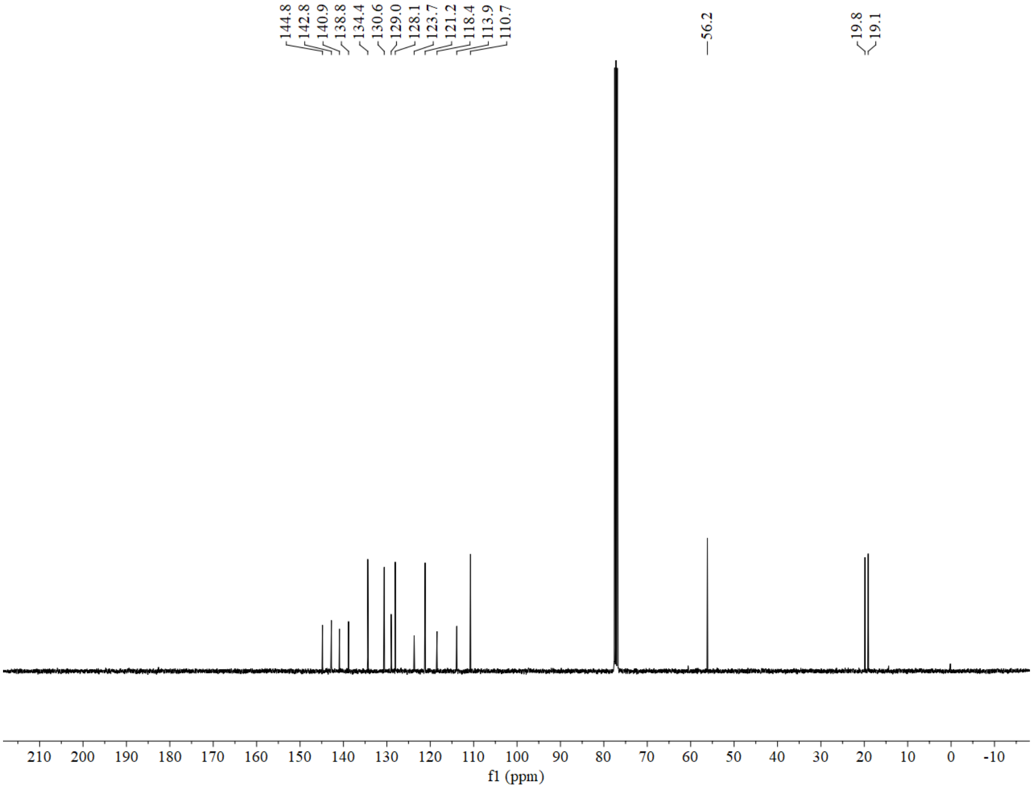

Supplementary Figure 95 HPLC spectra of racemic **3h**

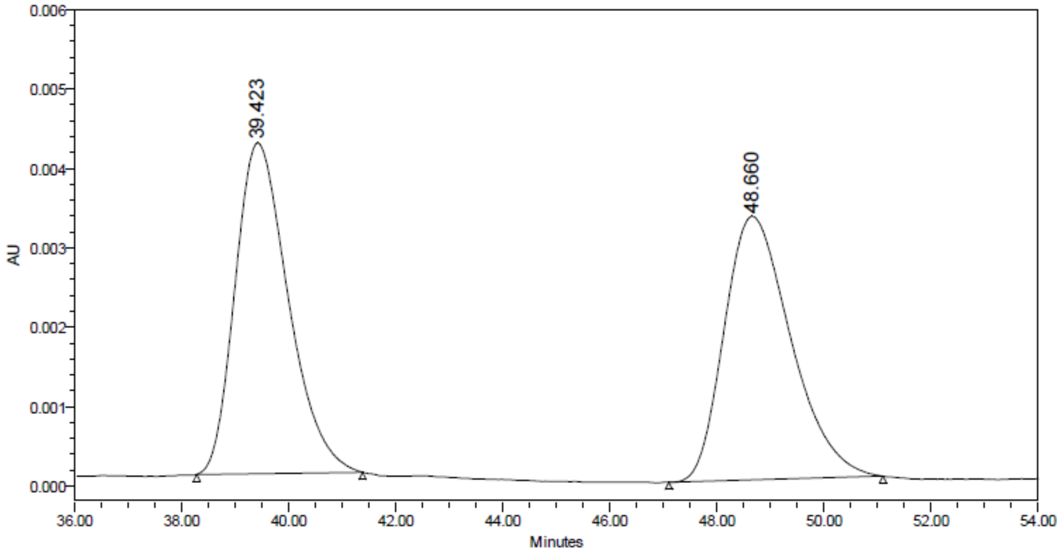

	RT	Area	% Area	Height
1	39.423	287608	50.08	4165
2	48.660	286722	49.92	3319

Supplementary Figure 96 HPLC spectra of (*S*)- **3h**

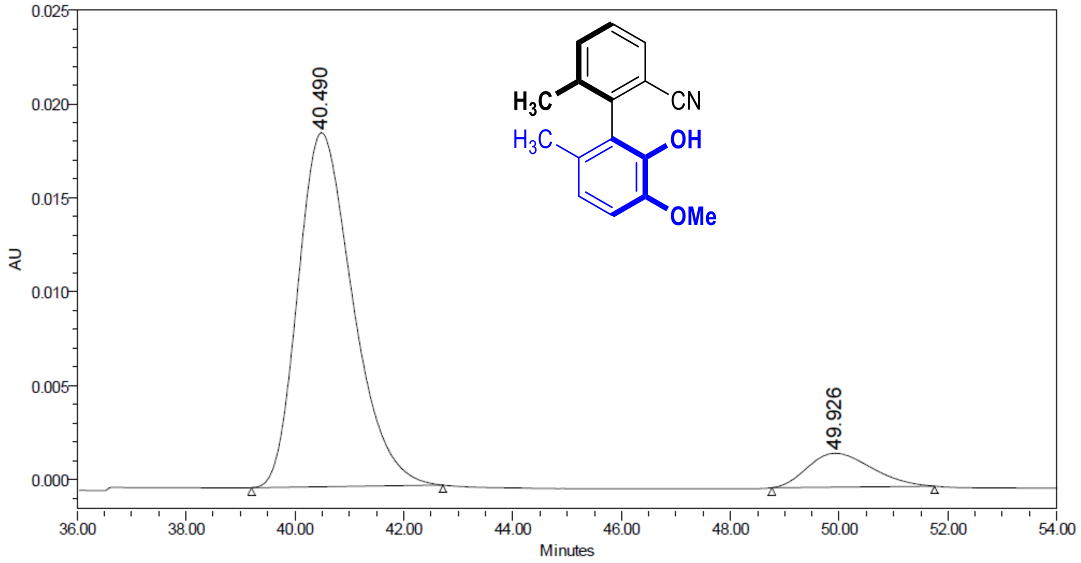

	RT	Area	% Area	Height
1	40.490	1316791	89.91	18849
2	49.926	147814	10.09	1804

Supplementary Figure 97 ^{1}H NMR (400 MHz, $CDCl_3$) of **3i**

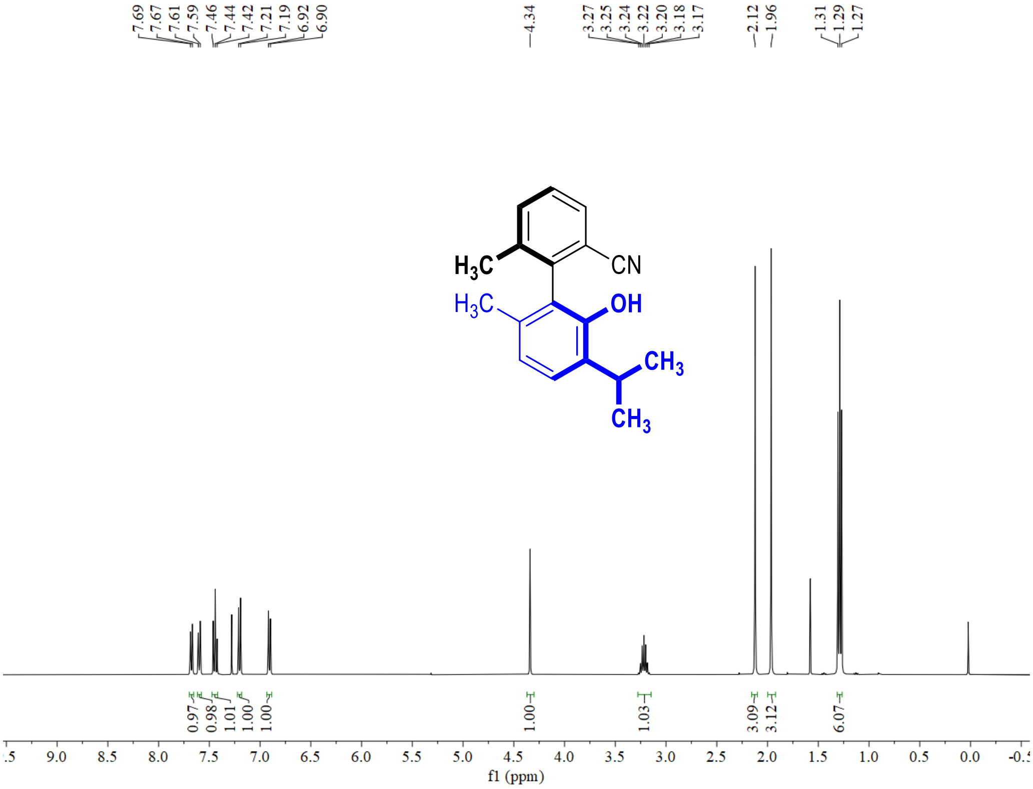

Supplementary Figure 98 ^{13}C NMR (400 MHz, $CDCl_3$) of **3i**

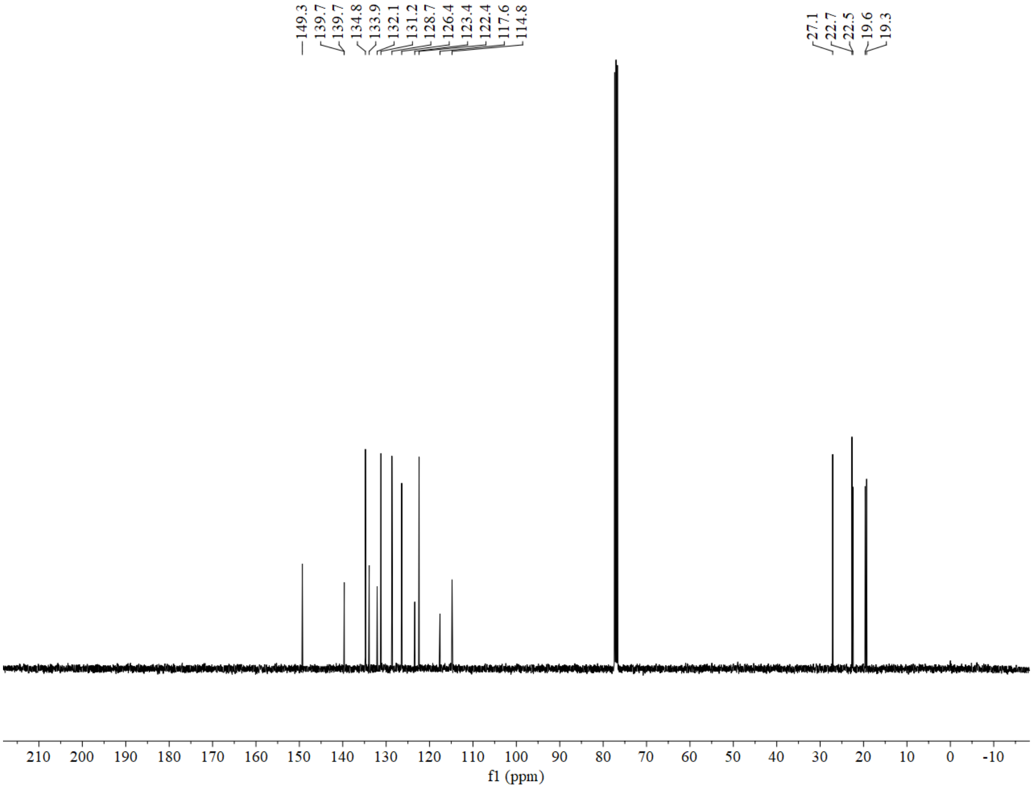

Supplementary Figure 99 HPLC spectra of racemic **3i**

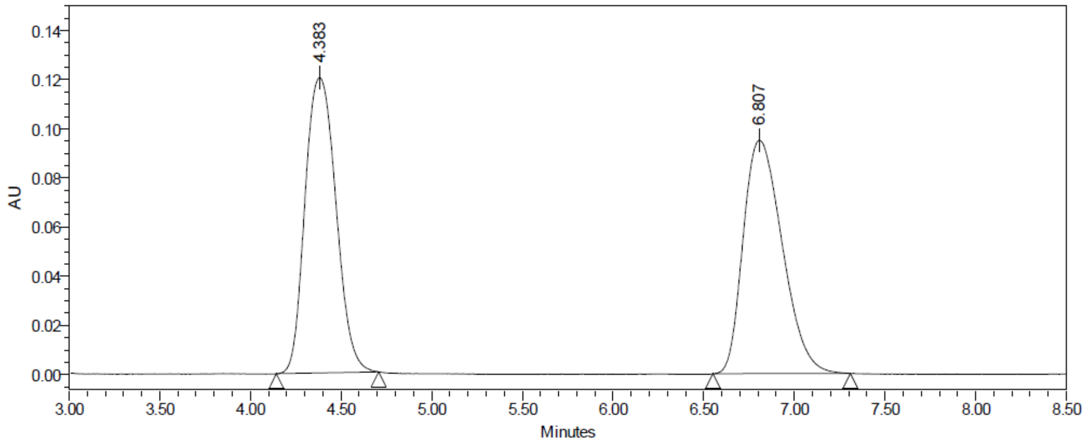

	RT	Area	% Area	Height
1	4.383	1428529	50.04	120312
2	6.807	1426027	49.96	95057

Supplementary Figure 100 HPLC spectra of (*S*)- **3i**

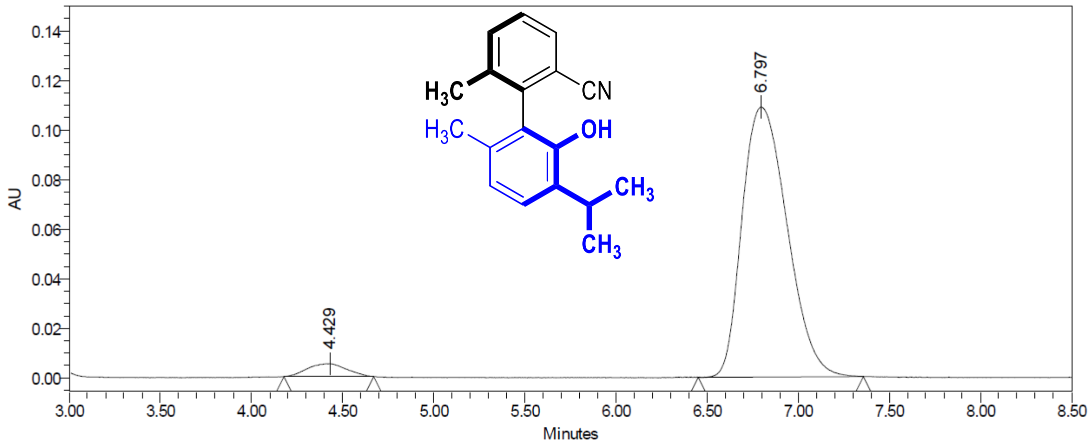

	RT	Area	% Area	Height
1	4.429	77438	3.97	5191
2	6.797	1874636	96.03	108955

Supplementary Figure 101 ^{1}H NMR (400 MHz, $CDCl_3$) of **3j**

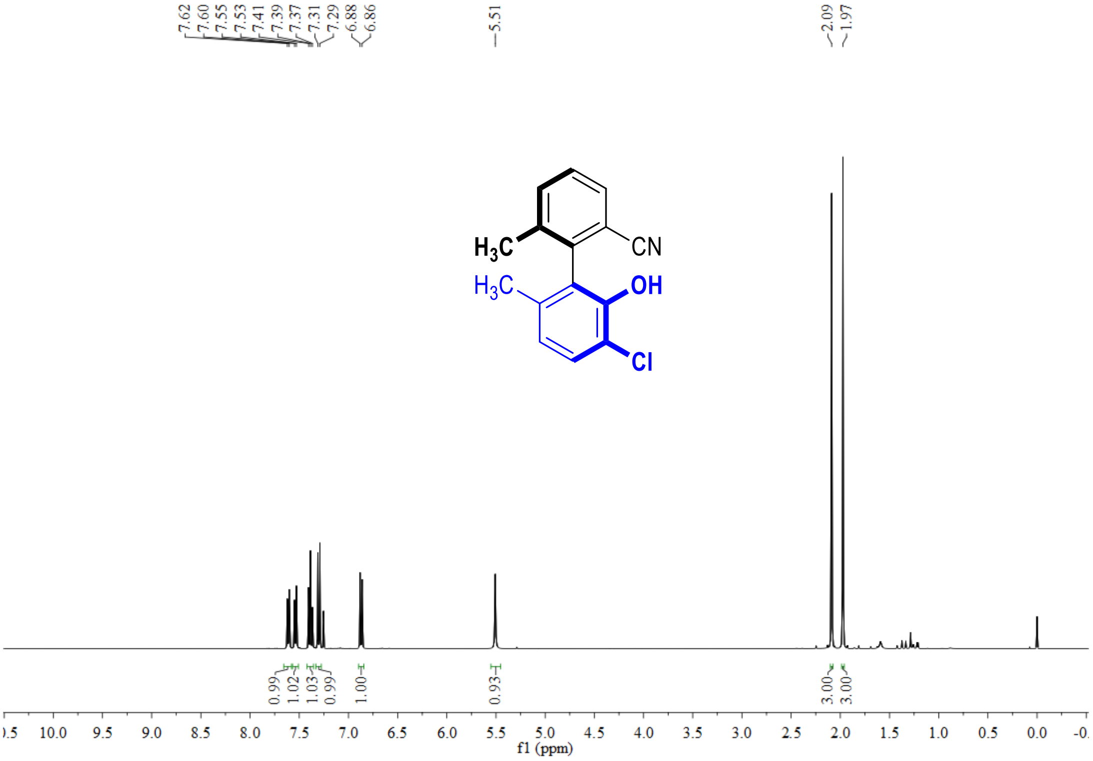

Supplementary Figure 102 ^{13}C NMR (400 MHz, $CDCl_3$) of **3j**

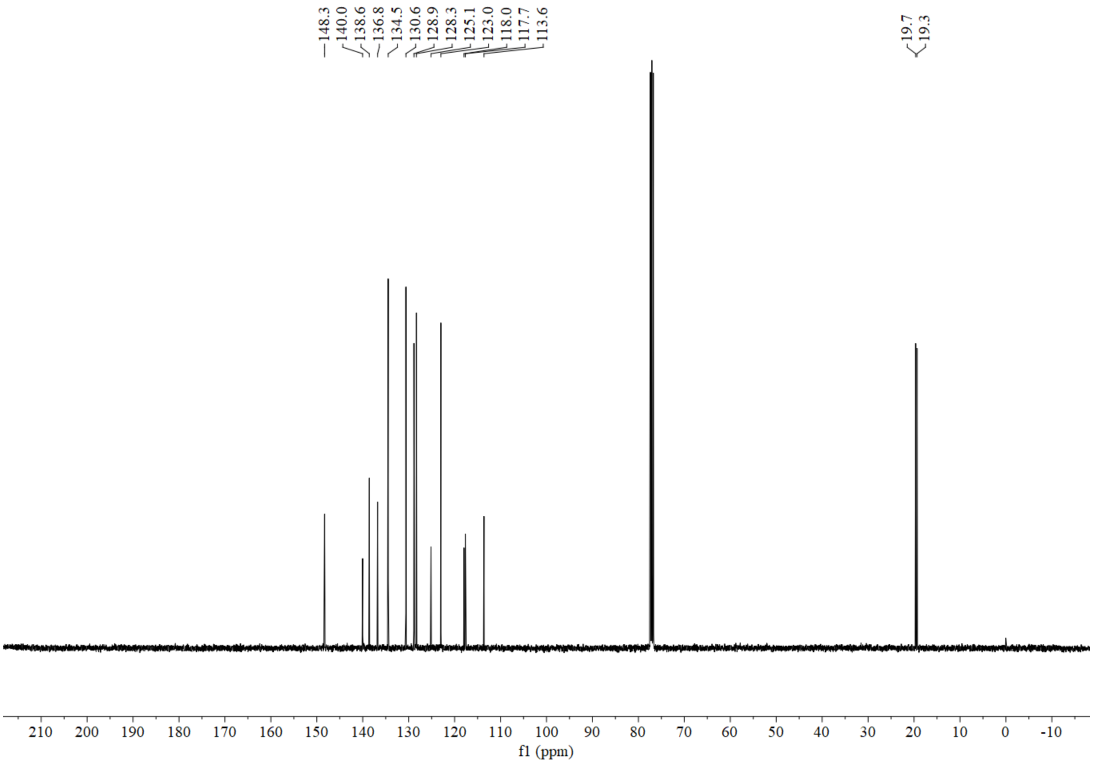

Supplementary Figure 103 HPLC spectra of racemic **3j**

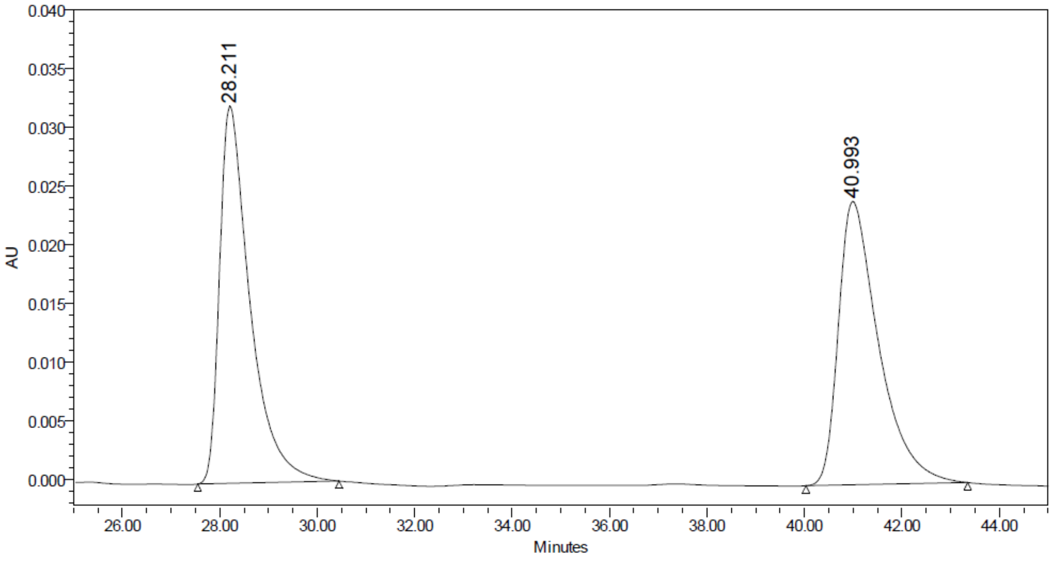

	RT	Area	% Area	Height
1	28.211	1395850	50.17	32097
2	40.993	1386423	49.83	24127

Supplementary Figure 104 HPLC spectra of (*S*)- **3j**

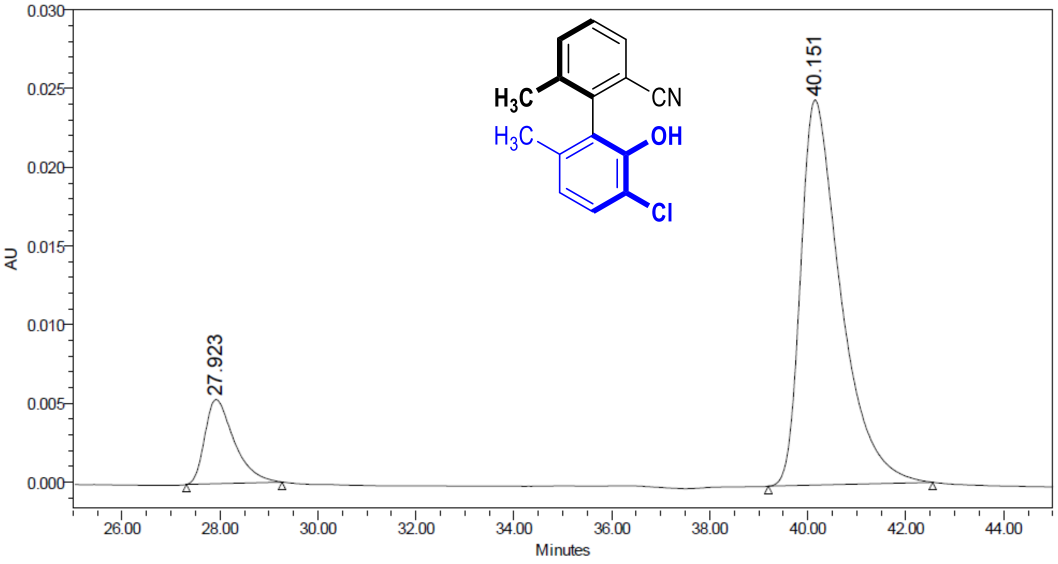

	RT	Area	% Area	Height
1	27.923	222644	13.80	5349
2	40.151	1390247	86.20	24456

Supplementary Figure 105 ^{1}H NMR (400 MHz, Acetone-d_6) of **3k**

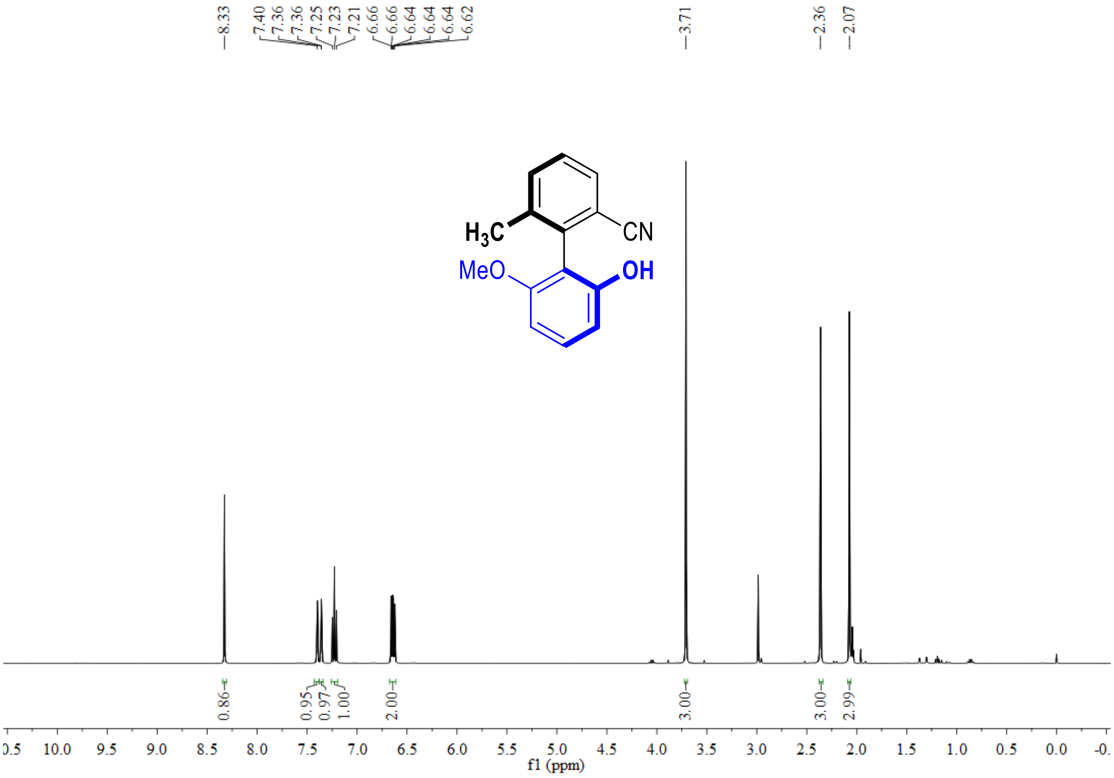

Supplementary Figure 106 ^{13}C NMR (400 MHz, Acetone-d_6) of **3k**

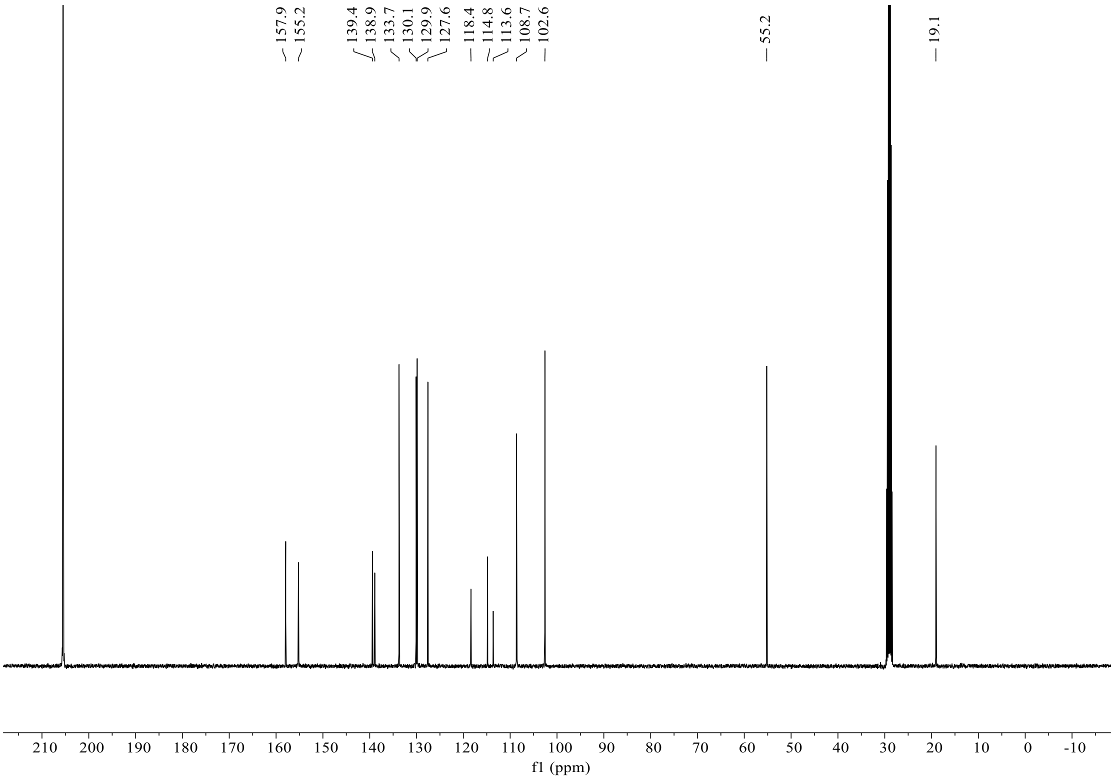

Supplementary Figure 107 HPLC spectra of racemic **3k**

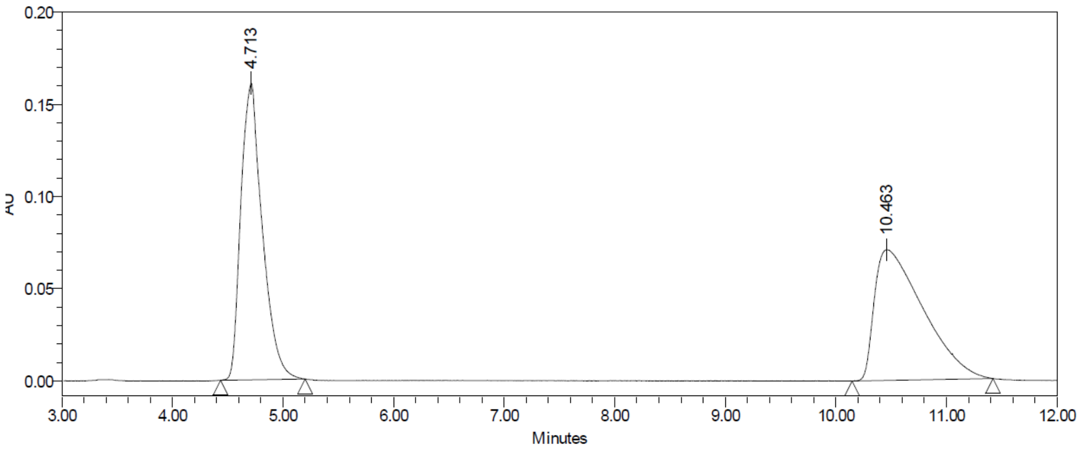

	RT	Area	% Area	Height
1	4.713	2163628	49.97	160782
2	10.463	2166354	50.03	70895

Supplementary Figure 108 HPLC spectra of (*R*)- **3k**

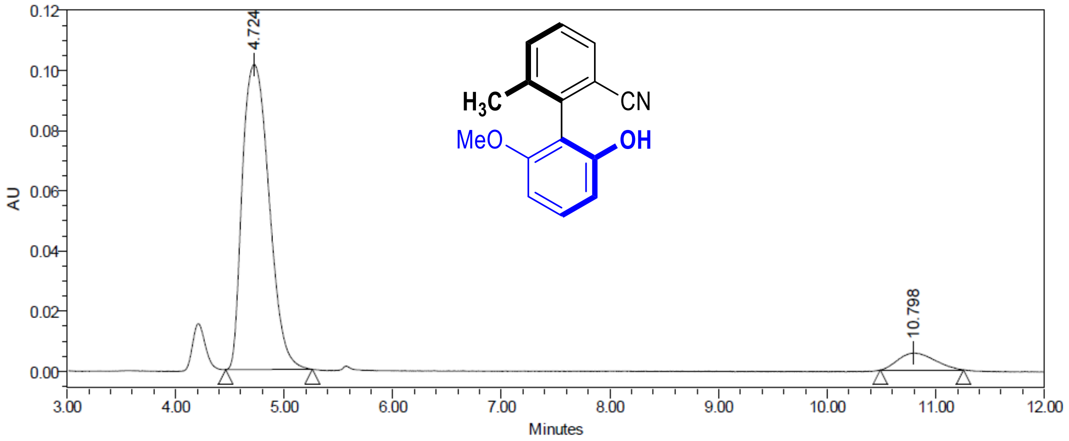

	RT	Area	% Area	Height
1	4.724	1738477	92.96	101357
2	10.798	131644	7.04	5639

Supplementary Figure 109 ^{1}H NMR (400 MHz, $CDCl_3$) of **3l**

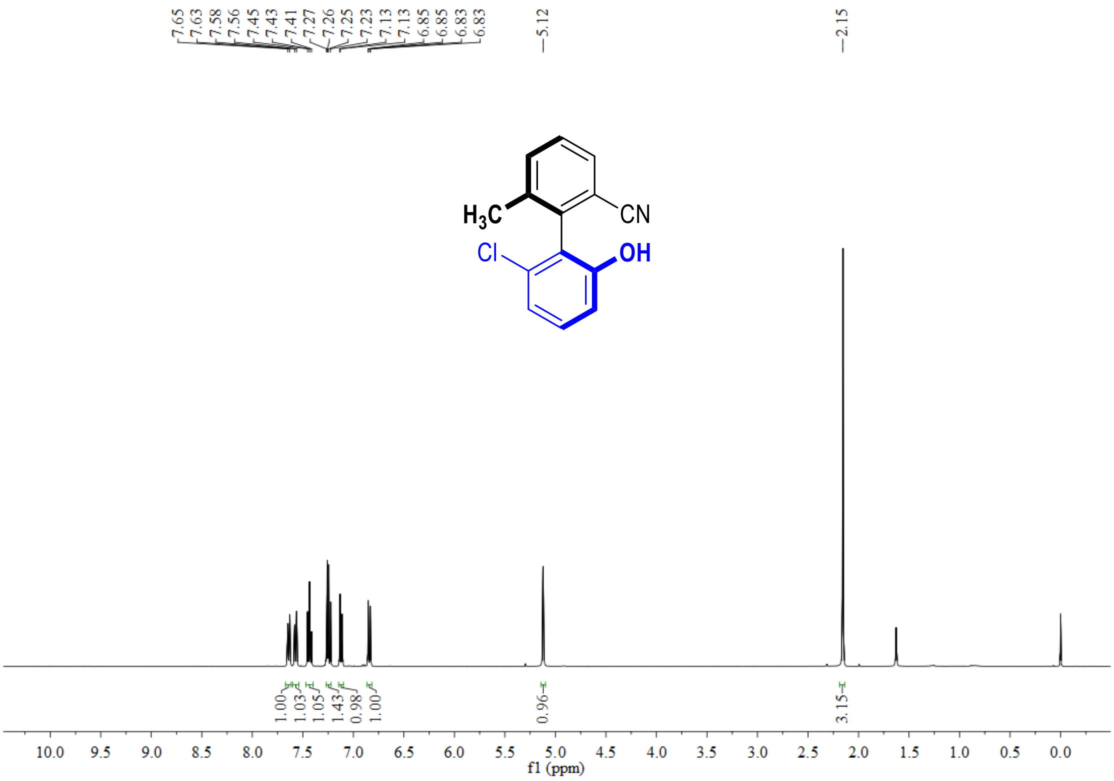

Supplementary Figure 110 ^{13}C NMR (400 MHz, $CDCl_3$) of **3l**

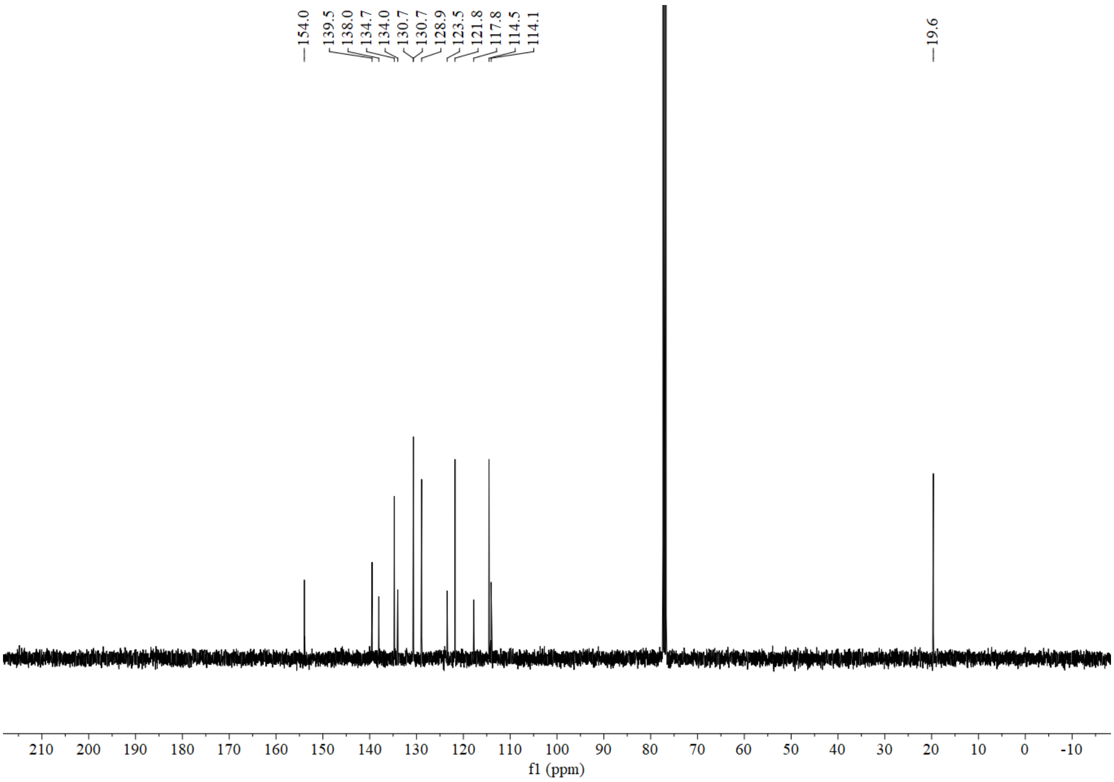

Supplementary Figure 111 HPLC spectra of racemic **3l**

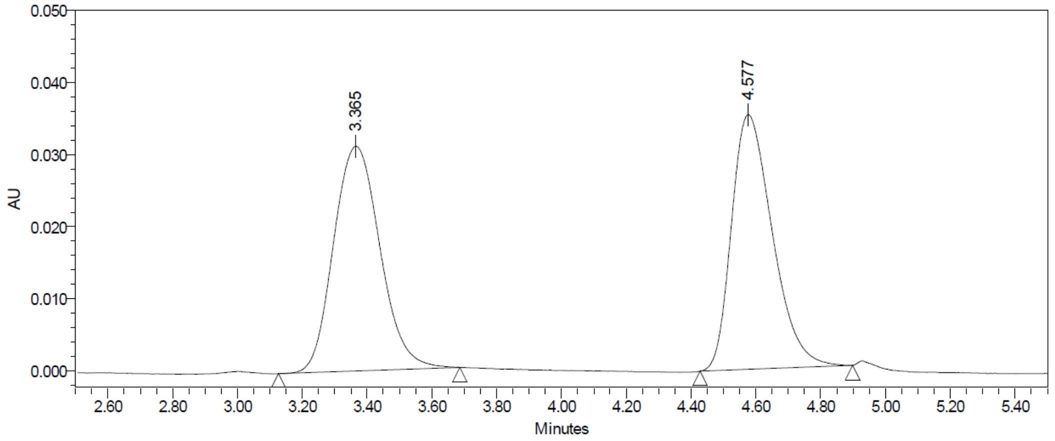

	RT	Area	% Area	Height
1	3.365	310535	50.05	31144
2	4.577	309911	49.95	35316

Supplementary Figure 112 HPLC spectra of (*R*)- **3l**

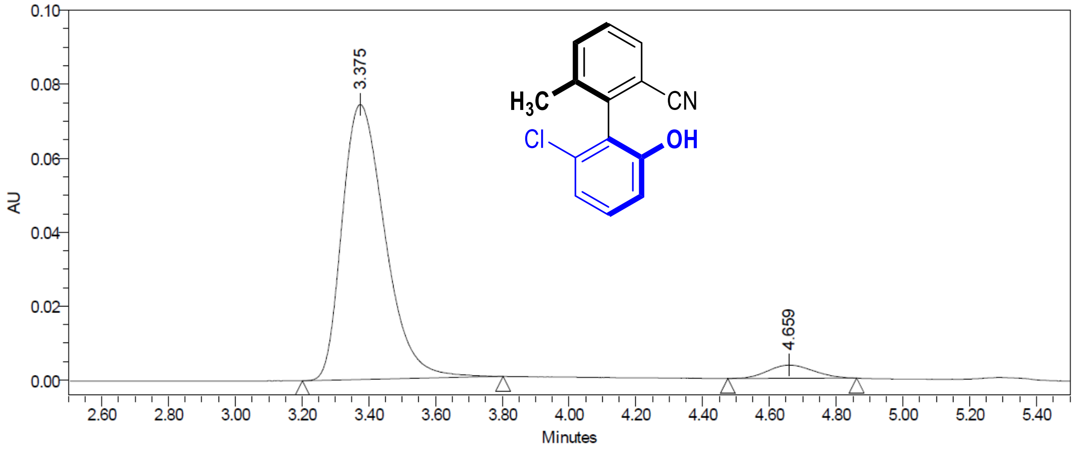

	RT	Area	% Area	Height
1	3.375	664385	95.16	74320
2	4.659	33828	4.84	3591

Supplementary Figure 113 ^{1}H NMR (400 MHz, $CDCl_3$) of **3m**

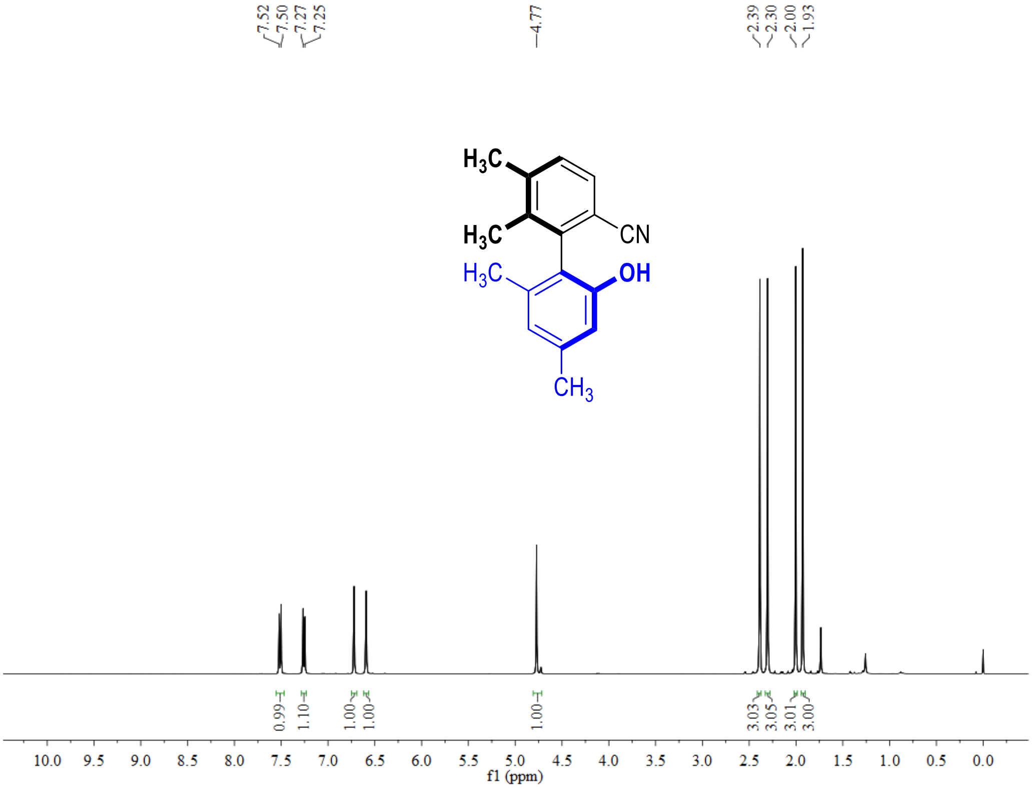

Supplementary Figure 114 ^{13}C NMR (400 MHz, $CDCl_3$) of **3m**

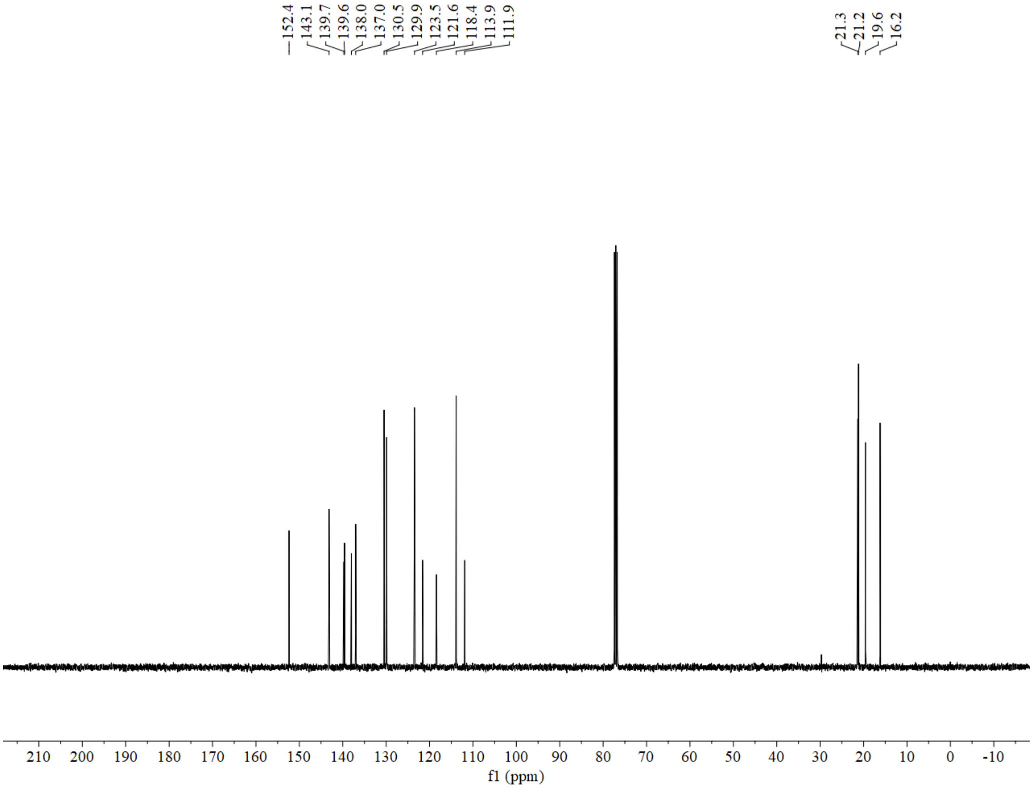

Supplementary Figure 115 HPLC spectra of racemic **3m**

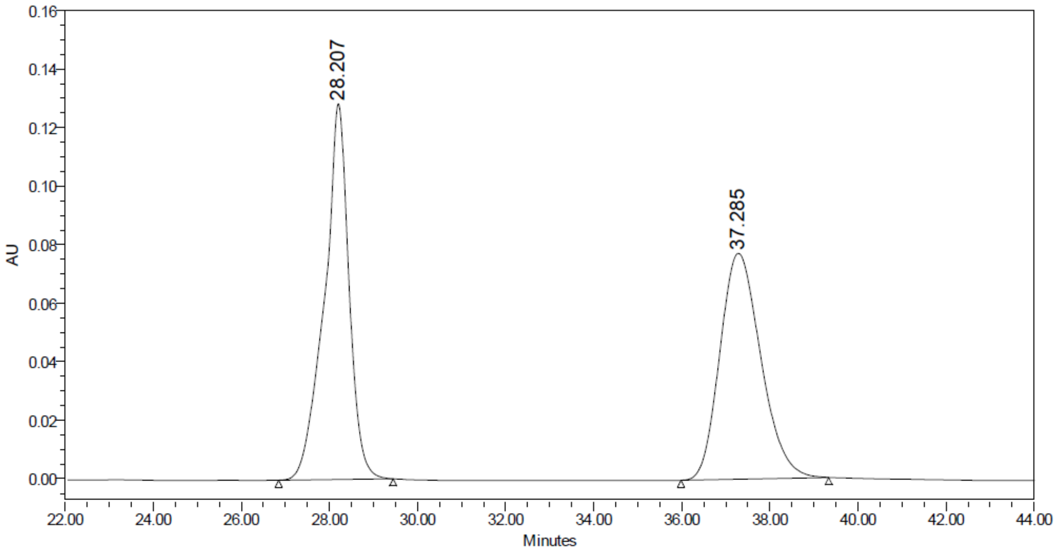

	RT	Area	% Area	Height
1	28.207	4991691	50.05	128446
2	37.285	4980740	49.95	77221

Supplementary Figure 116 HPLC spectra of (*S*)- **3m**

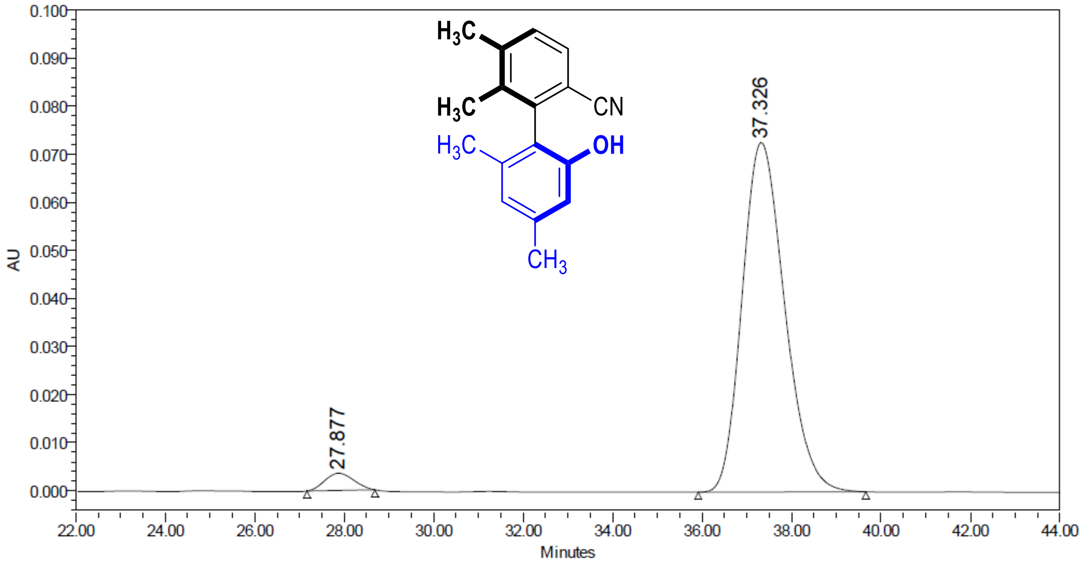

	RT	Area	% Area	Height
1	27.877	160448	3.24	3573
2	37.326	4794349	96.76	72668

Supplementary Figure 117 ^{1}H NMR (400 MHz, $CDCl_3$) of **3n**

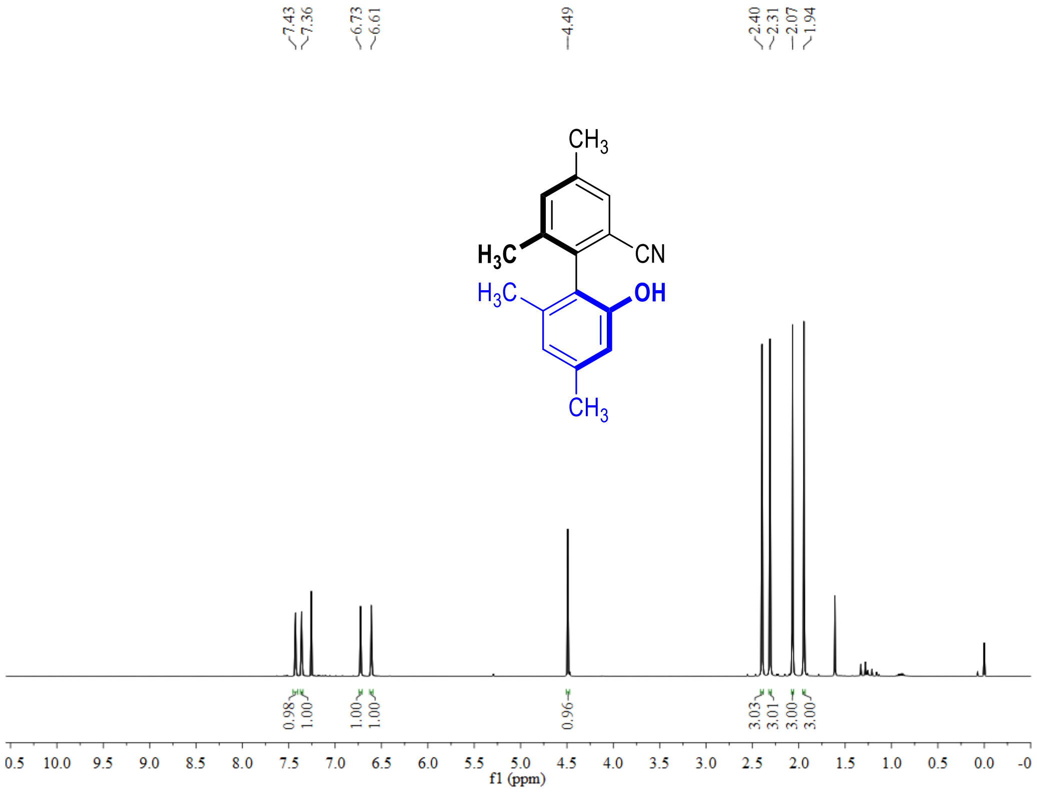

Supplementary Figure 118 ^{13}C NMR (400 MHz, $CDCl_3$) of **3n**

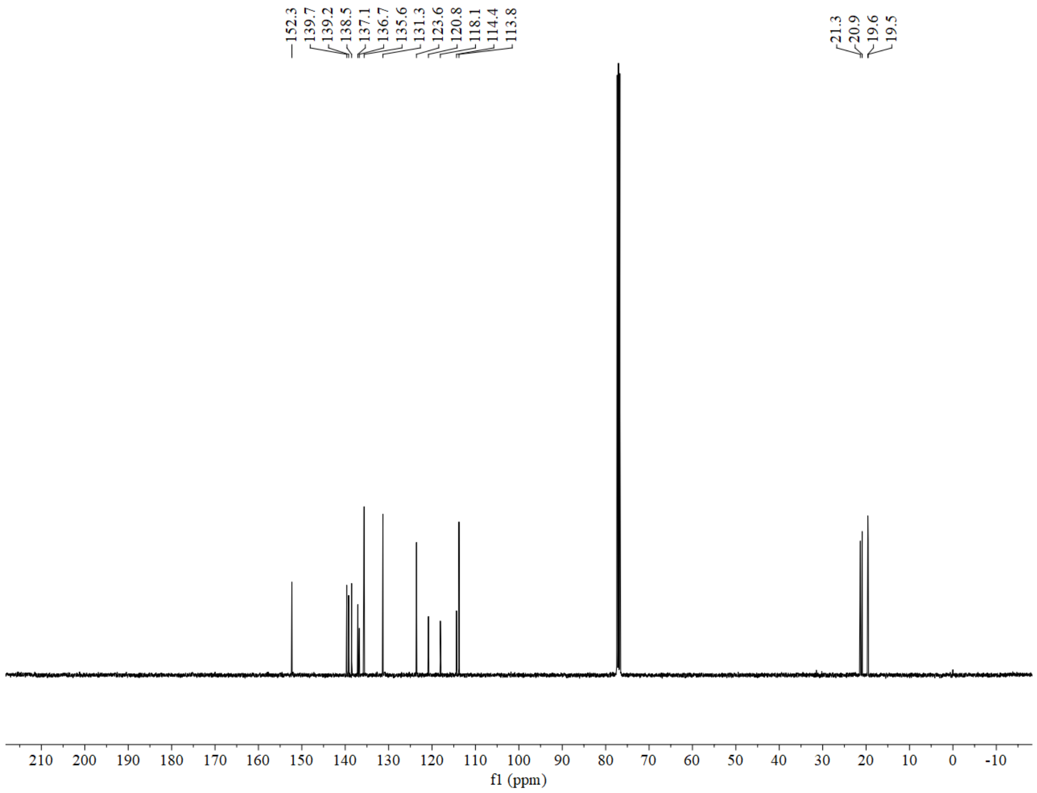

Supplementary Figure 119 HPLC spectra of racemic **3n**

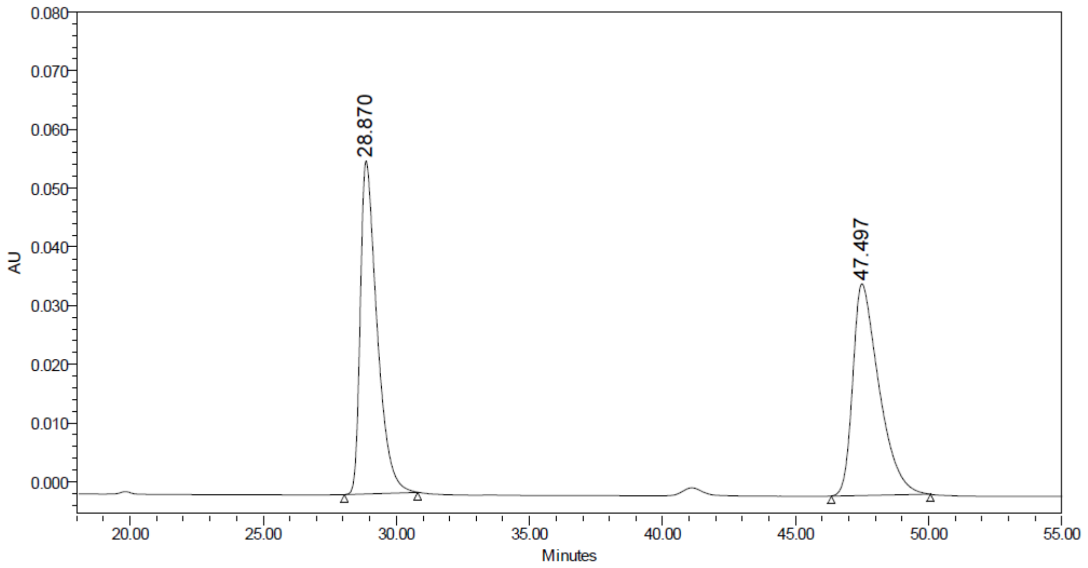

	RT	Area	% Area	Height
1	28.870	2476254	50.02	56712
2	47.497	2473977	49.98	36049

Supplementary Figure 120 HPLC spectra of (*S*)- **3n**

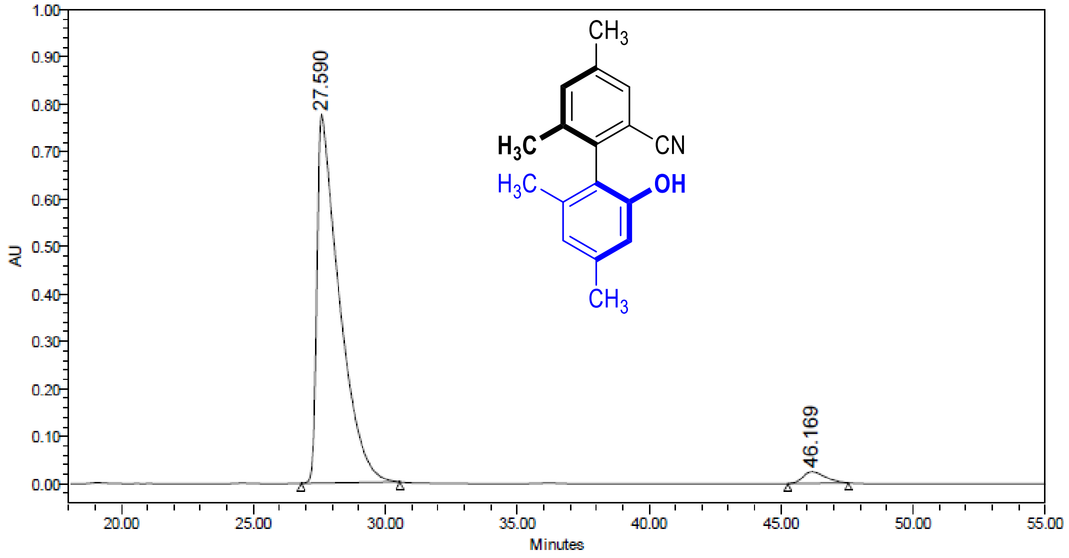

	RT	Area	% Area	Height
1	27.590	45611598	96.98	776306
2	46.169	1422760	3.02	24417

Supplementary Figure 121 ^{1}H NMR (400 MHz, $CDCl_3$) of **3o**

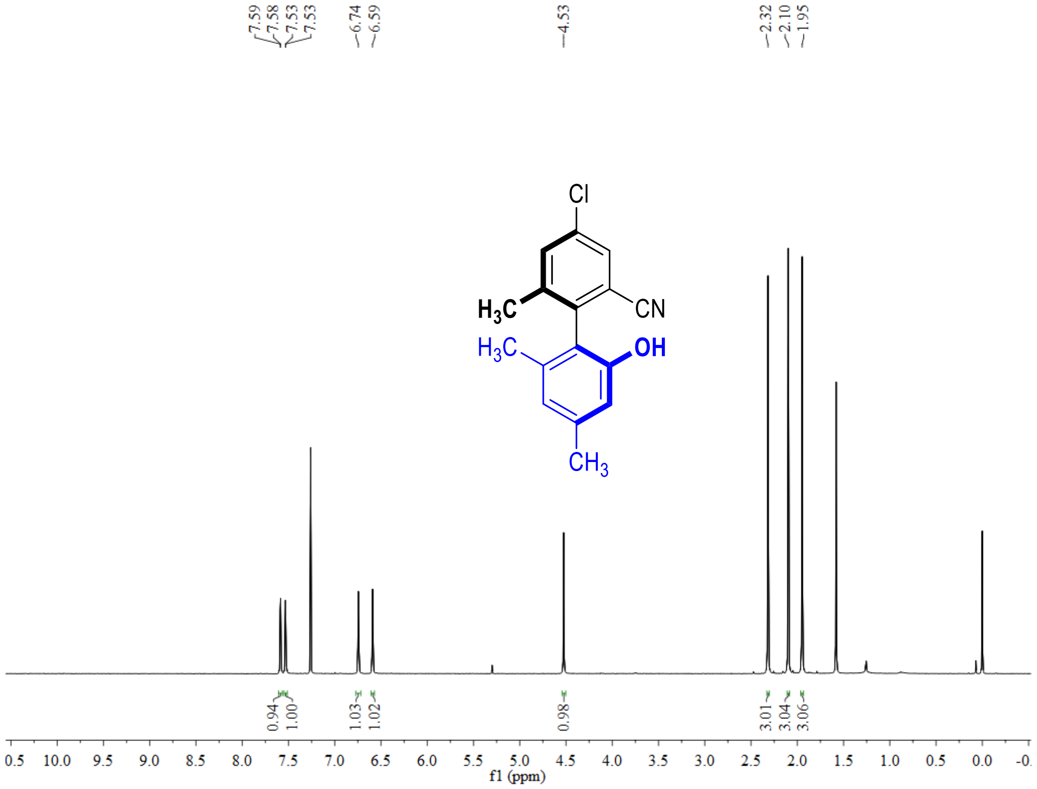

Supplementary Figure 122 ^{13}C NMR (400 MHz, $CDCl_3$) of **3o**

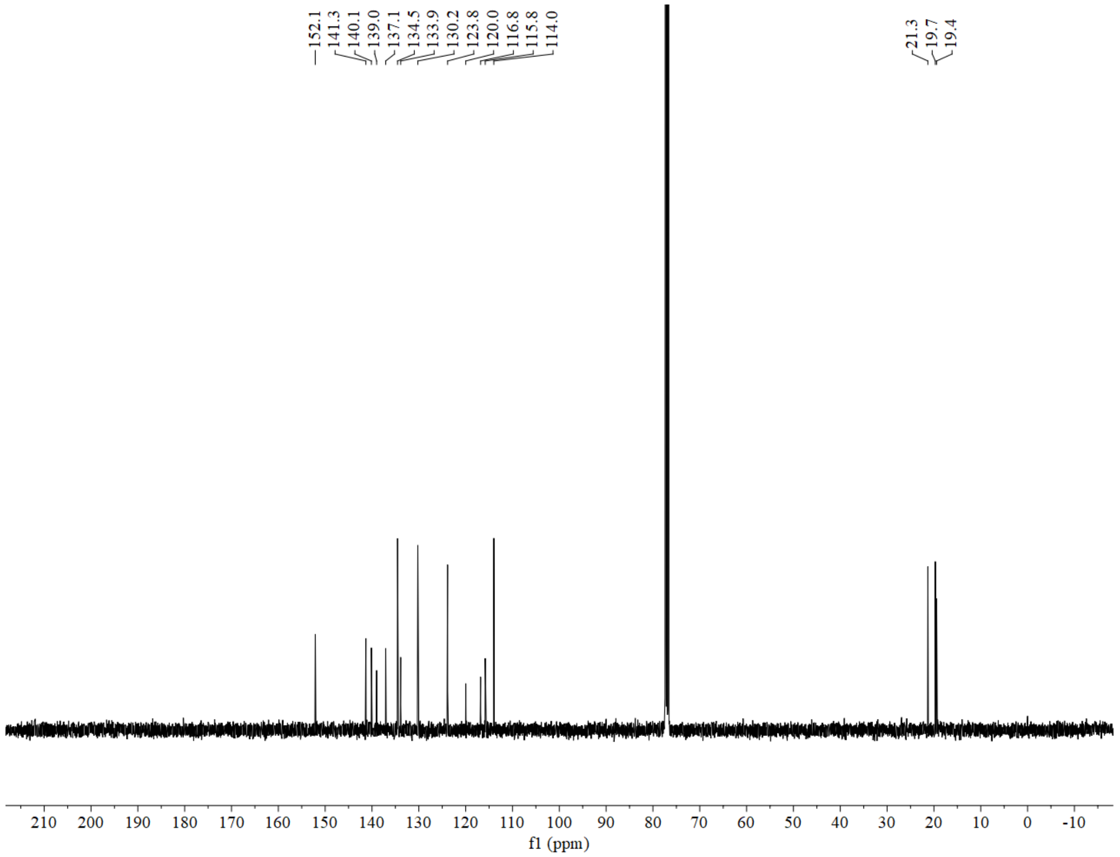

Supplementary Figure 123 HPLC spectra of racemic **3o**

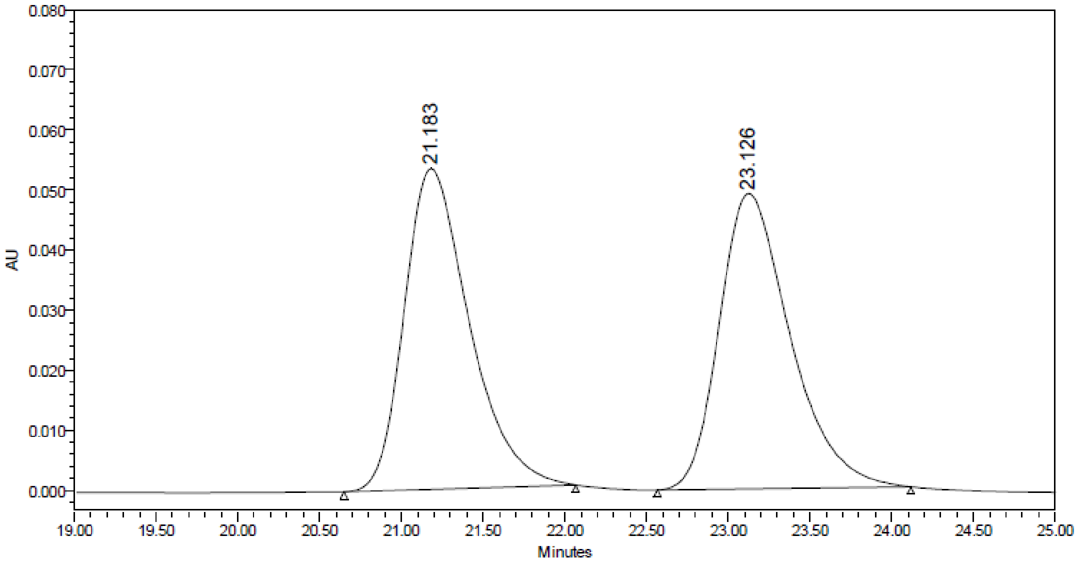

	RT	Area	% Area	Height
1	21.183	1478807	49.93	53382
2	23.126	1482870	50.07	49204

Supplementary Figure 124 HPLC spectra of (*S*)- **3o**

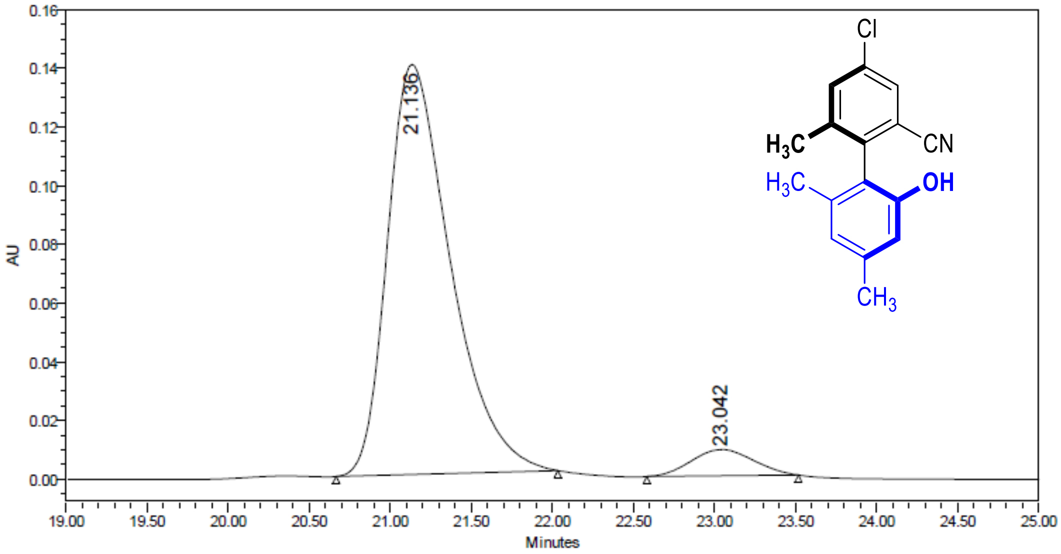

	RT	Area	% Area	Height
1	21.136	3779471	94.06	139749
2	23.042	238550	5.94	8951

Supplementary Figure 125 ^{1}H NMR (400 MHz, $CDCl_3$) of **3p**

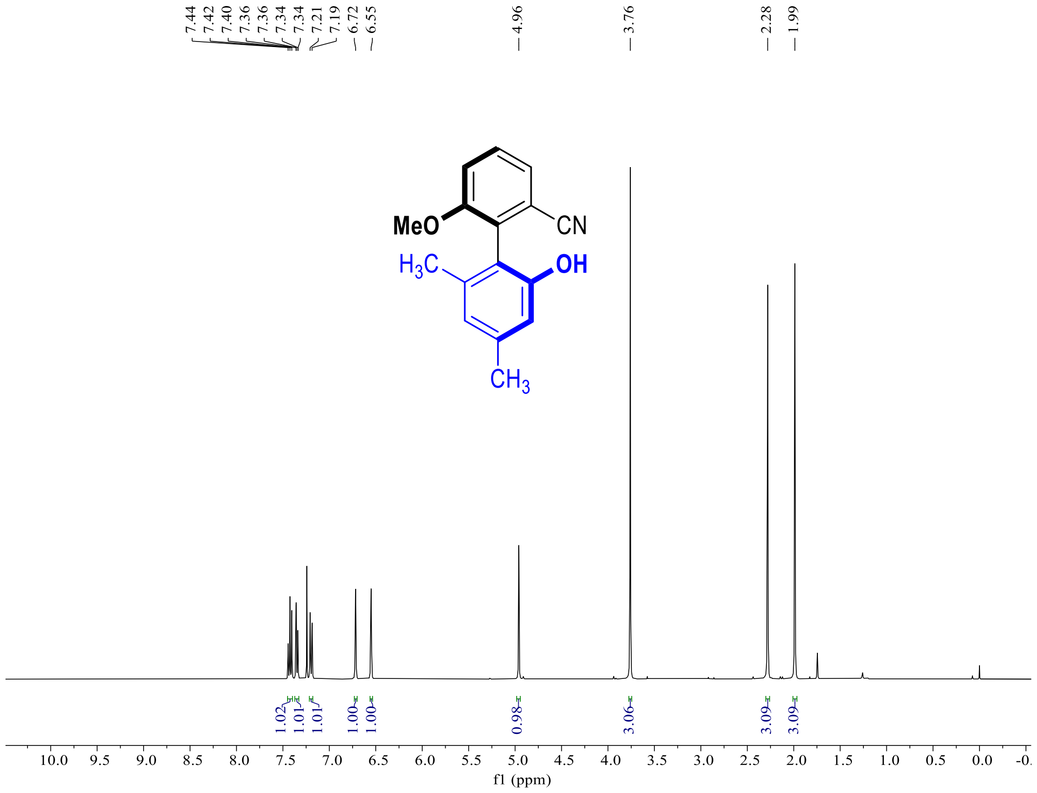

Supplementary Figure 126 ^{13}C NMR (400 MHz, $CDCl_3$) of **3p**

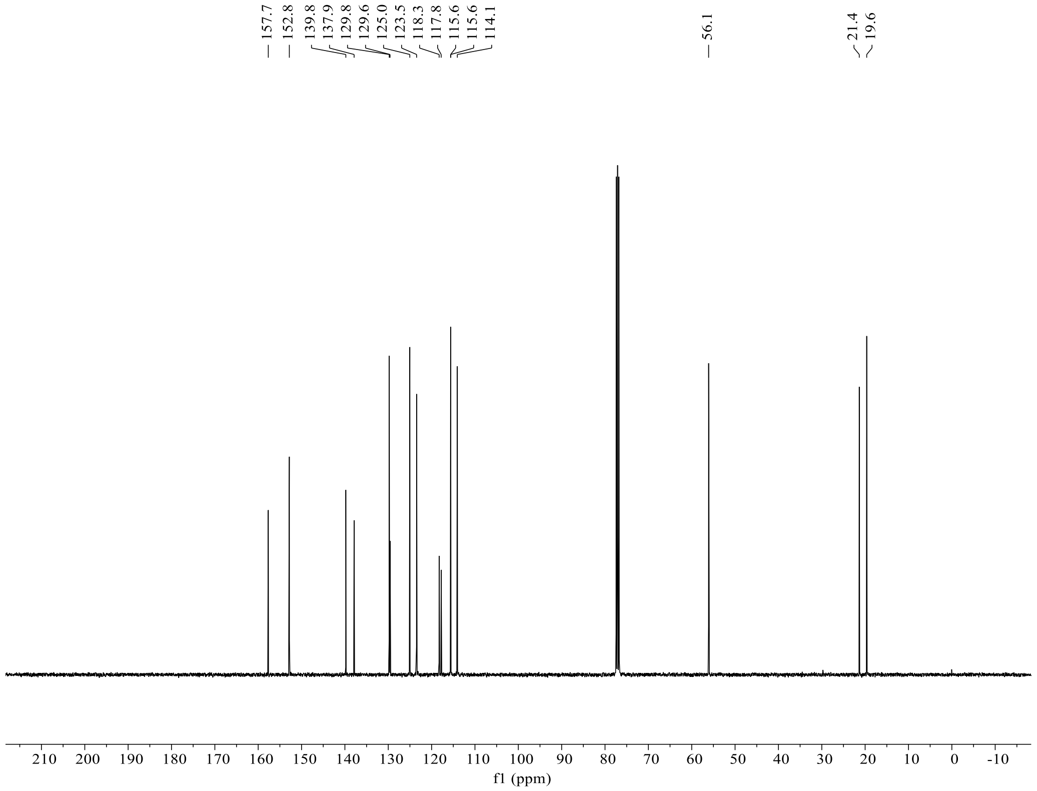

Supplementary Figure 127 HPLC spectra of racemic **3p**

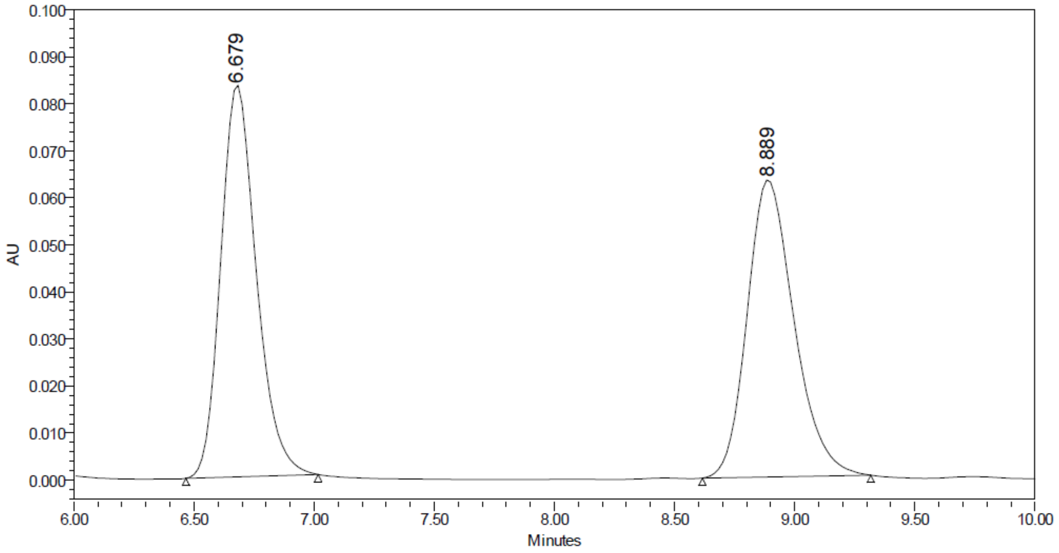

	RT	Area	% Area	Height
1	6.679	857943	50.05	83375
2	8.889	856082	49.95	63281

Supplementary Figure 128 HPLC spectra of (*R*)- **3p**

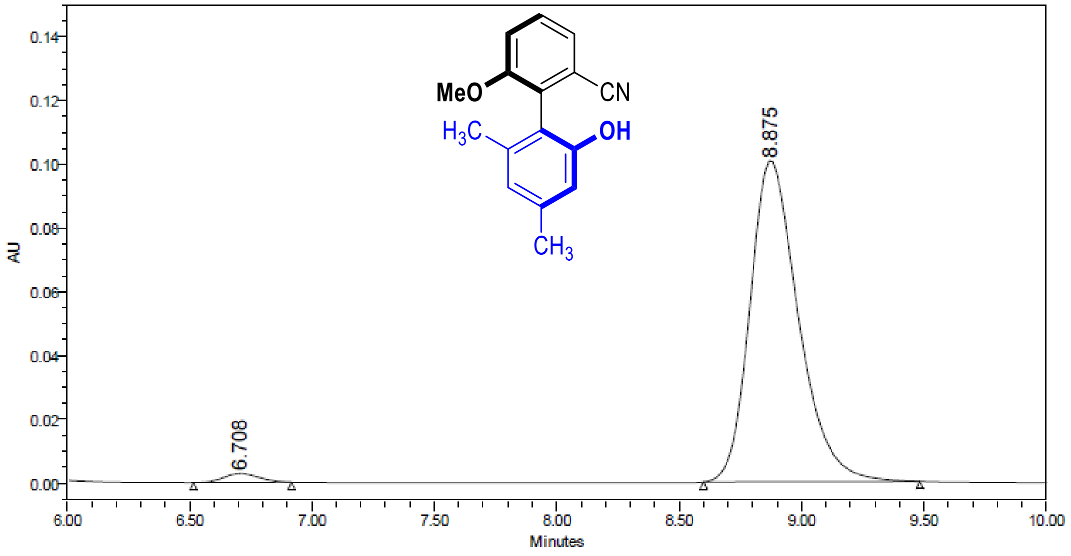

	RT	Area	% Area	Height
1	6.708	27614	1.95	2773
2	8.875	1389556	98.05	101090

Supplementary Figure 129 ^{1}H NMR (400 MHz, $CDCl_3$) of **3q**

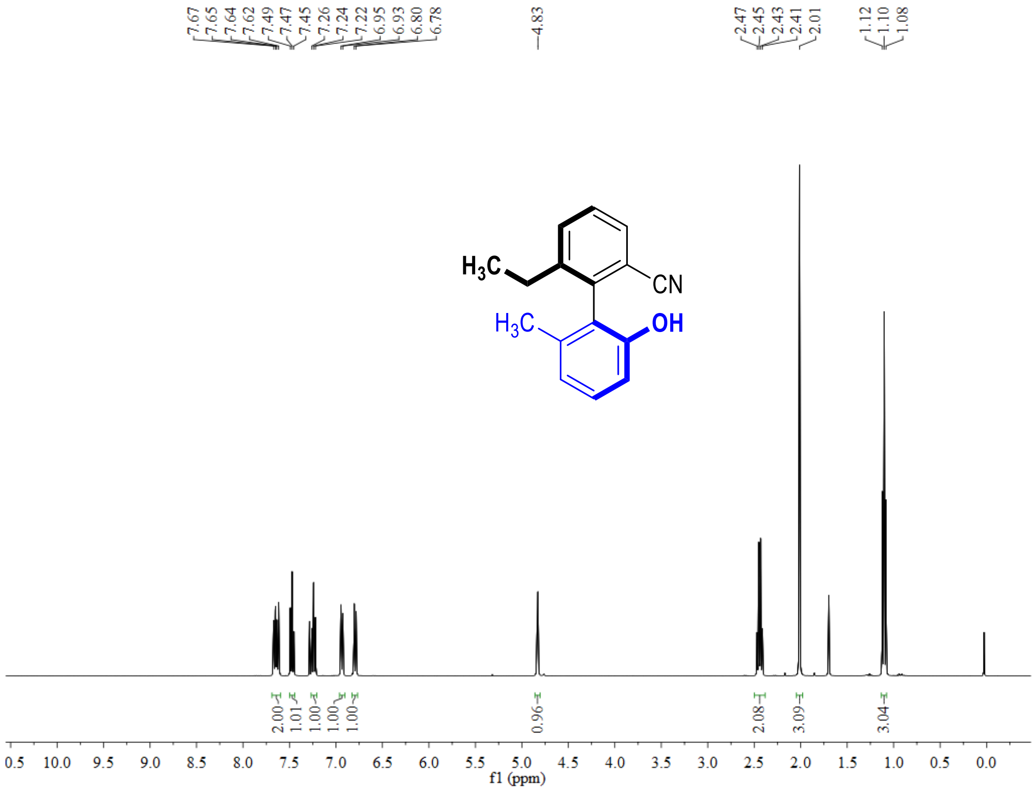

Supplementary Figure 130 ^{13}C NMR (400 MHz, $CDCl_3$) of **3q**

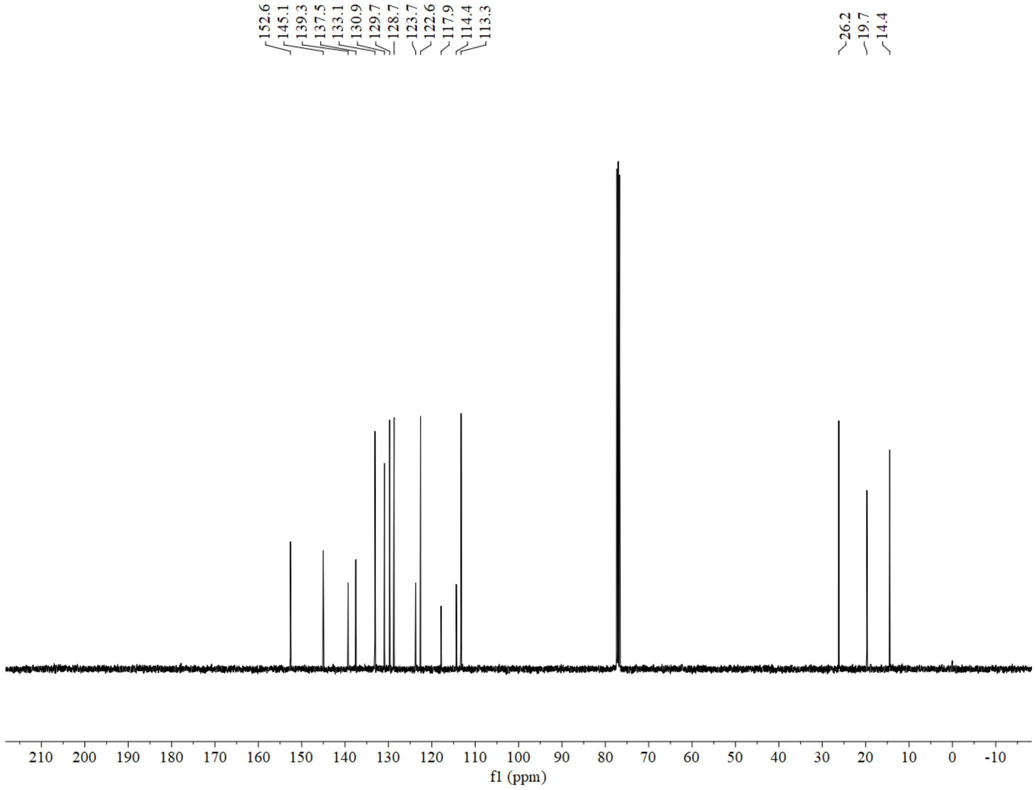

Supplementary Figure 131 HPLC spectra of racemic **3q**

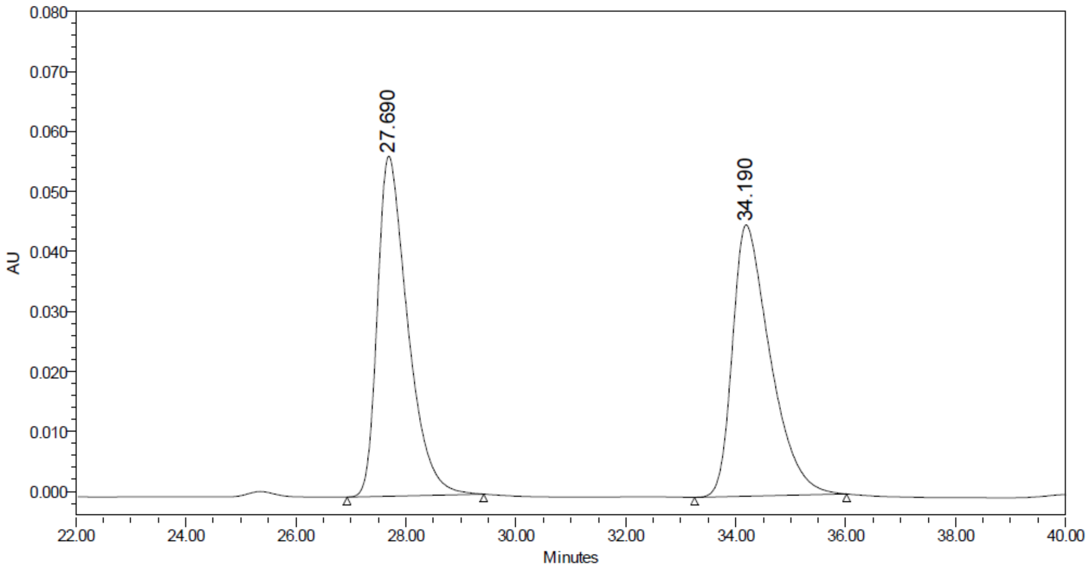

	RT	Area	% Area	Height
1	27.690	2167712	50.11	56685
2	34.190	2158506	49.89	45229

Supplementary Figure 132 HPLC spectra of (*S*)- **3q**

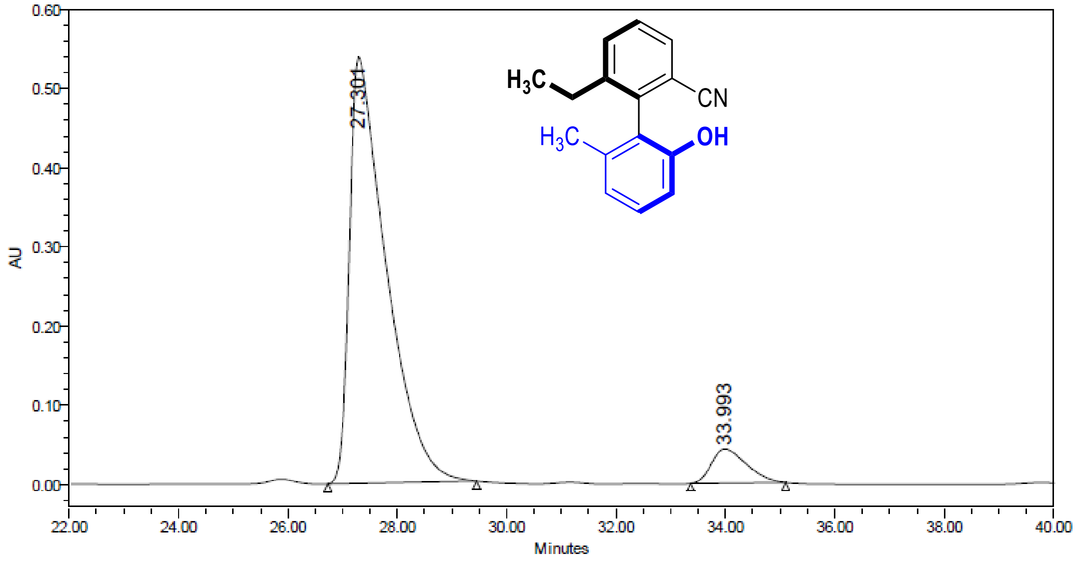

	RT	Area	% Area	Height
1	27.301	25077801	92.90	537973
2	33.993	1915630	7.10	43194

Supplementary Figure 133 ^{1}H NMR (400 MHz, Acetone-d_6) of **3r**

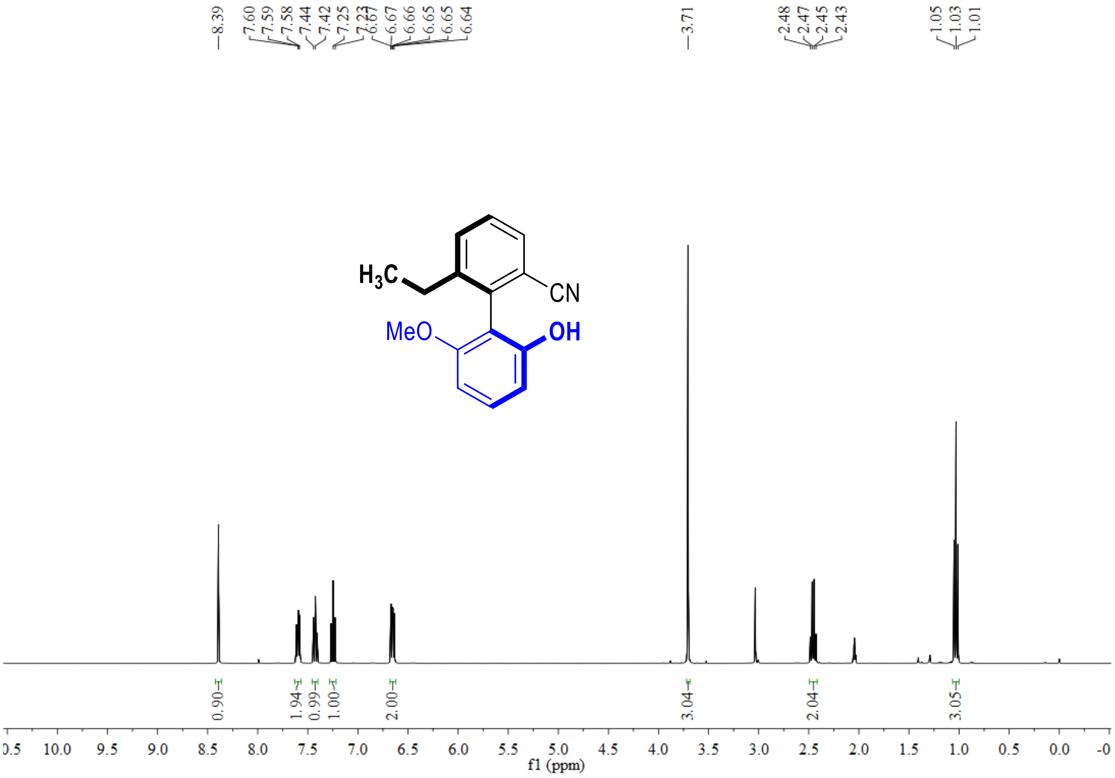

Supplementary Figure 134 ^{13}C NMR (400 MHz, Acetone-d_6) of **3r**

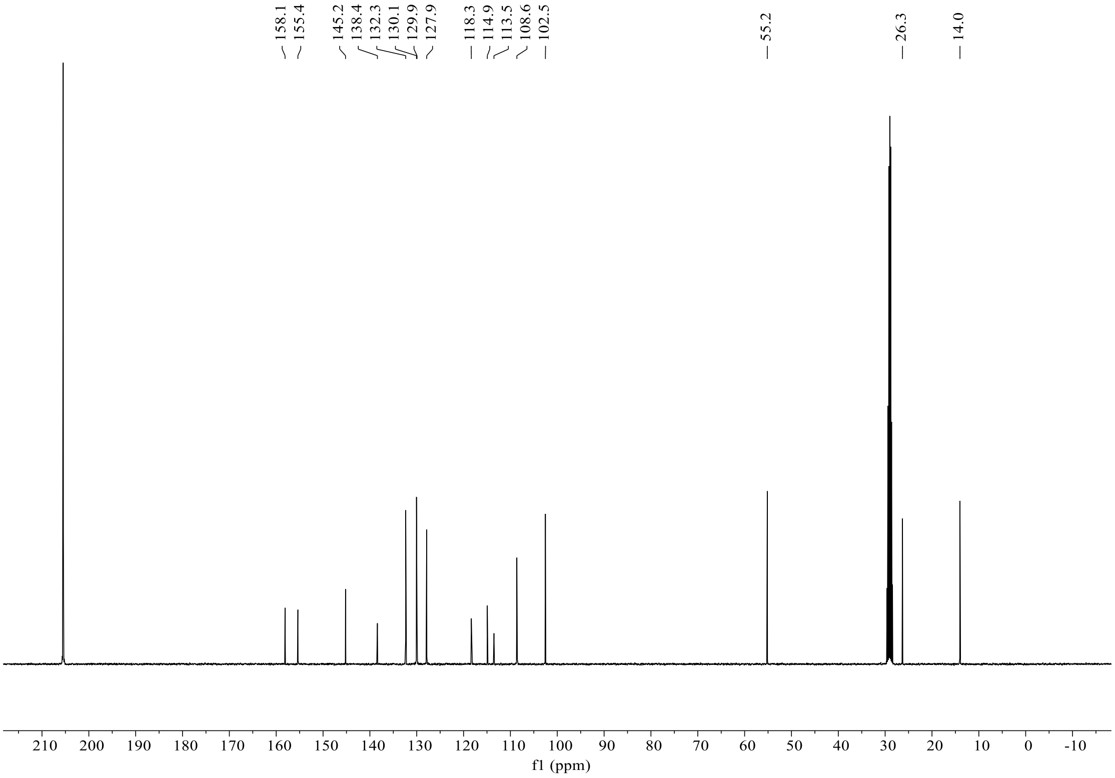

Supplementary Figure 135 HPLC spectra of racemic **3r**

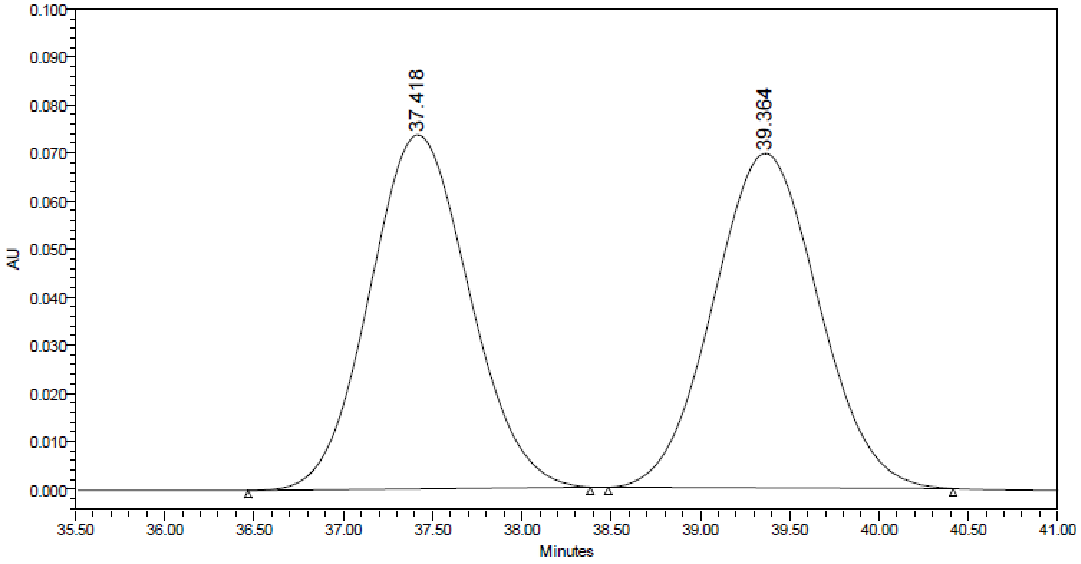

	RT	Area	% Area	Height
1	37.418	2863730	50.02	73664
2	39.364	2861734	49.98	69556

Supplementary Figure 136 HPLC spectra of (*R*)- **3r**

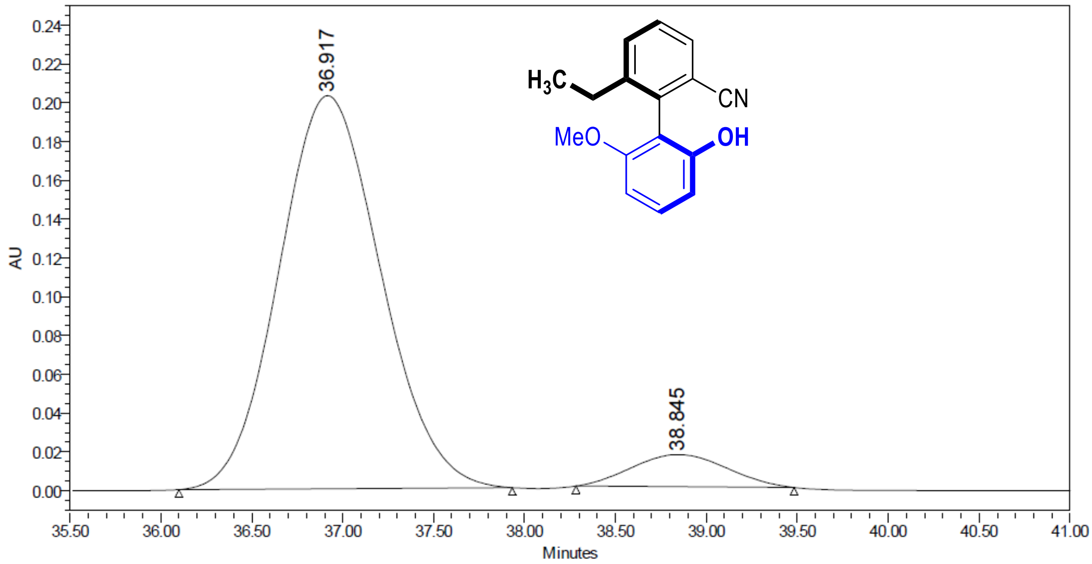

	RT	Area	% Area	Height
1	36.917	7883479	92.90	202714
2	38.845	602736	7.10	16580

Supplementary Figure 137 ^{1}H NMR (400 MHz, $CDCl_3$) of **3s**

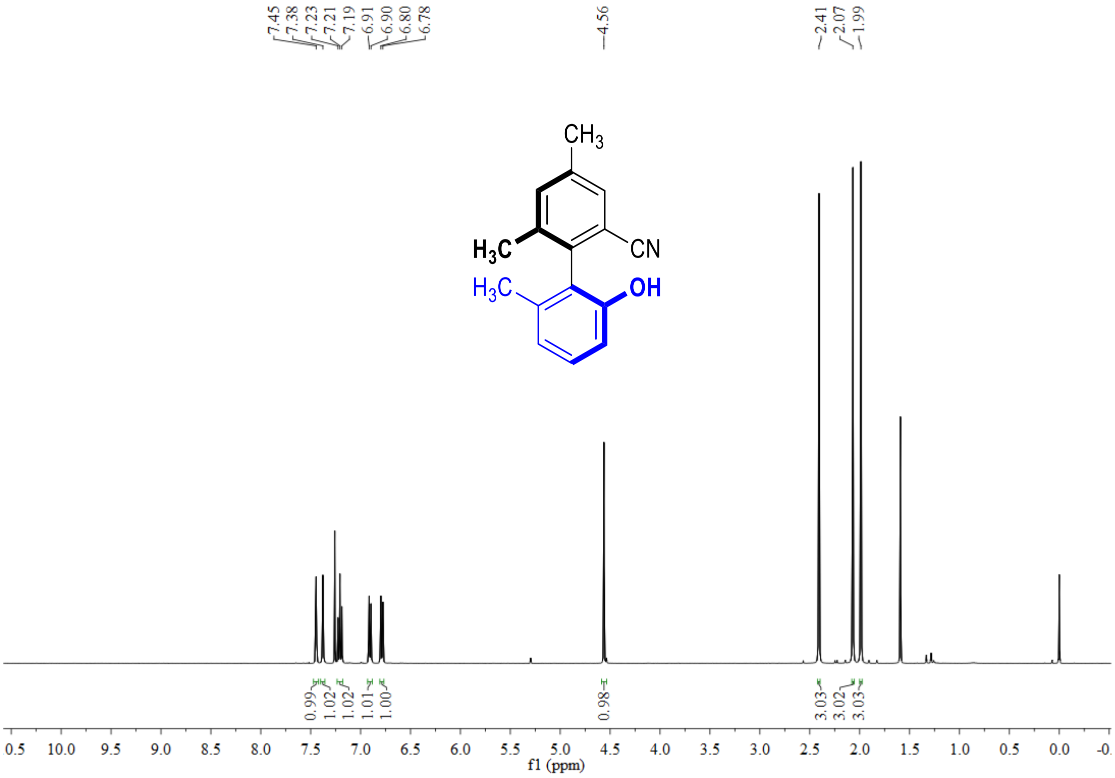

Supplementary Figure 138 ^{13}C NMR (400 MHz, $CDCl_3$) of **3s**

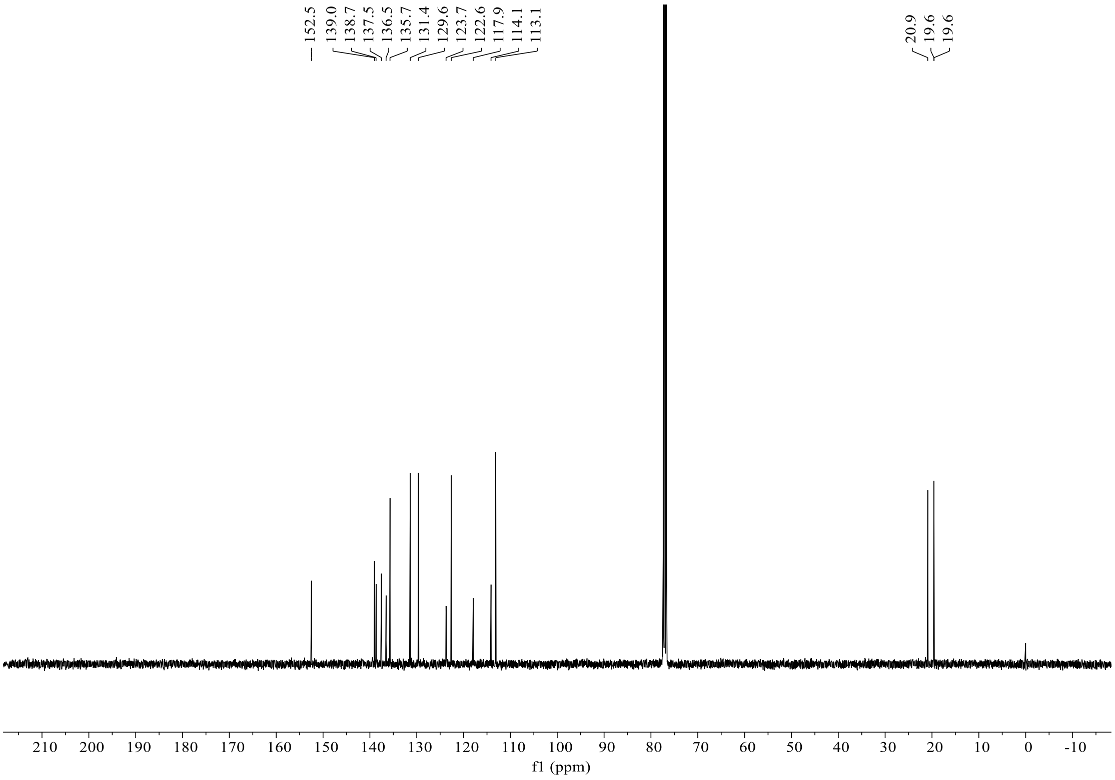

Supplementary Figure 139 HPLC spectra of racemic **3s**

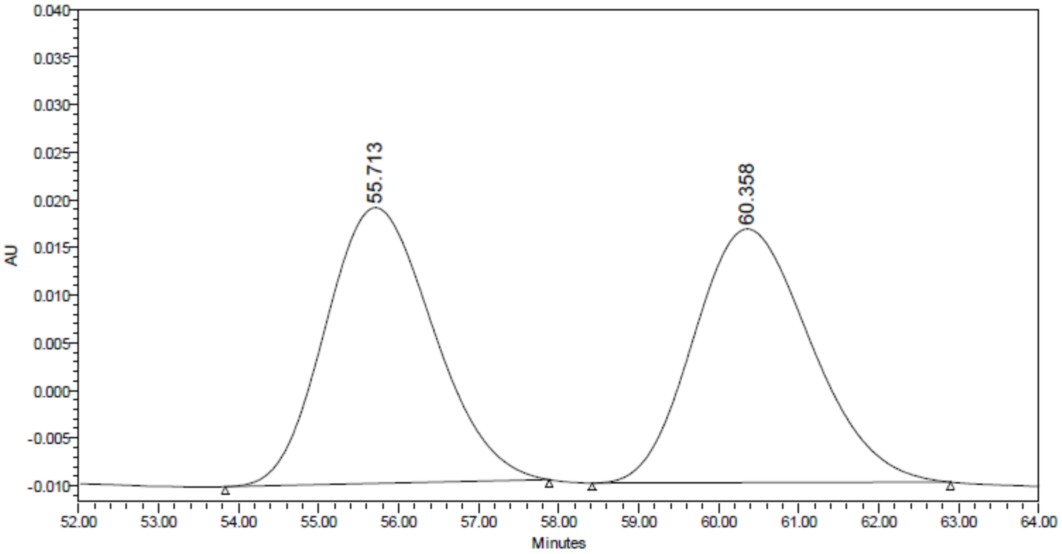

	RT	Area	% Area	Height
1	55.713	2715042	50.02	28953
2	60.358	2712808	49.98	26634

Supplementary Figure 140 HPLC spectra of (*S*)- **3s**

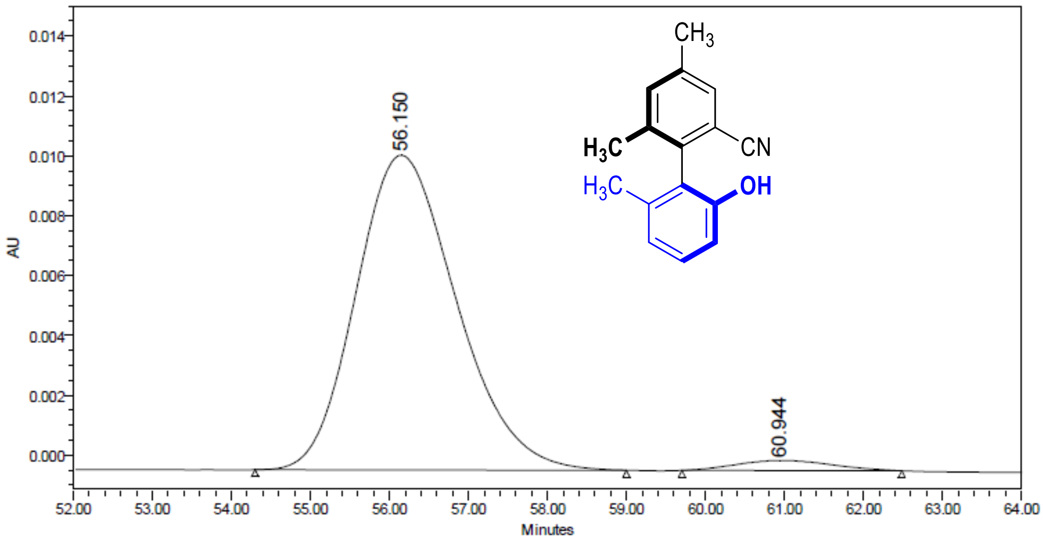

	RT	Area	% Area	Height
1	56.150	929282	97.03	10515
2	60.944	28462	2.97	337

Supplementary Figure 141 ^{1}H NMR (400 MHz, Acetone-d_6) of **3t**

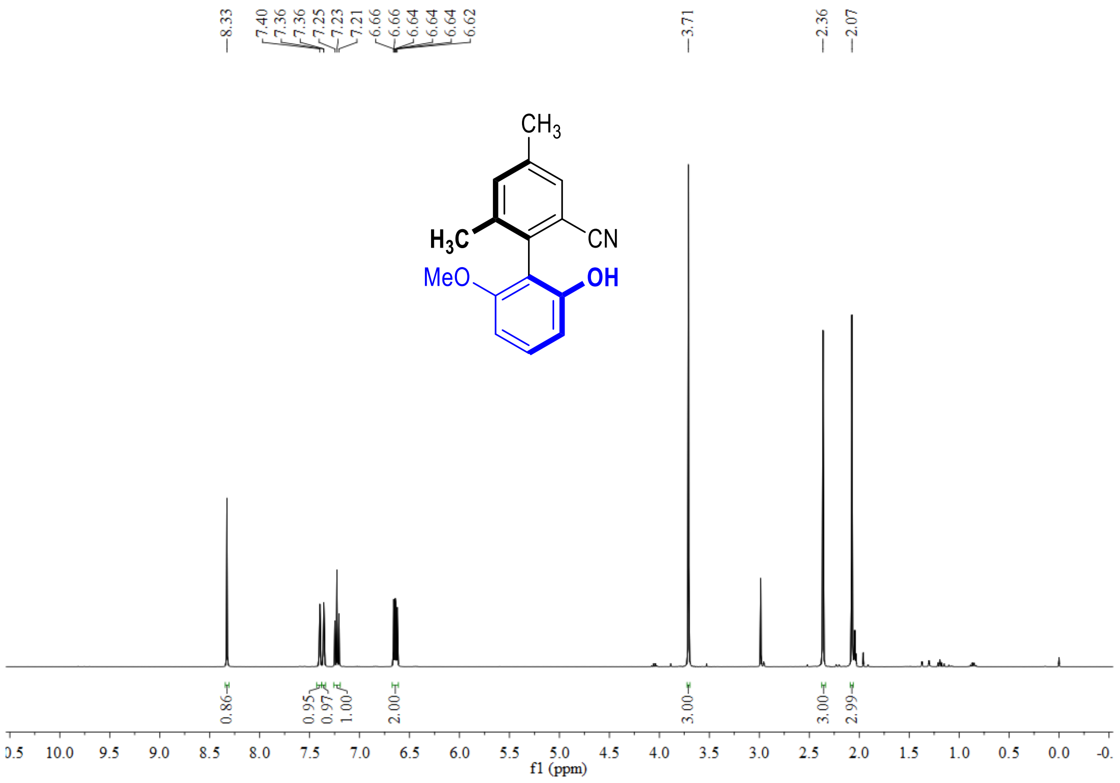

Supplementary Figure 142 ^{13}C NMR (400 MHz, Acetone-d_6) of **3t**

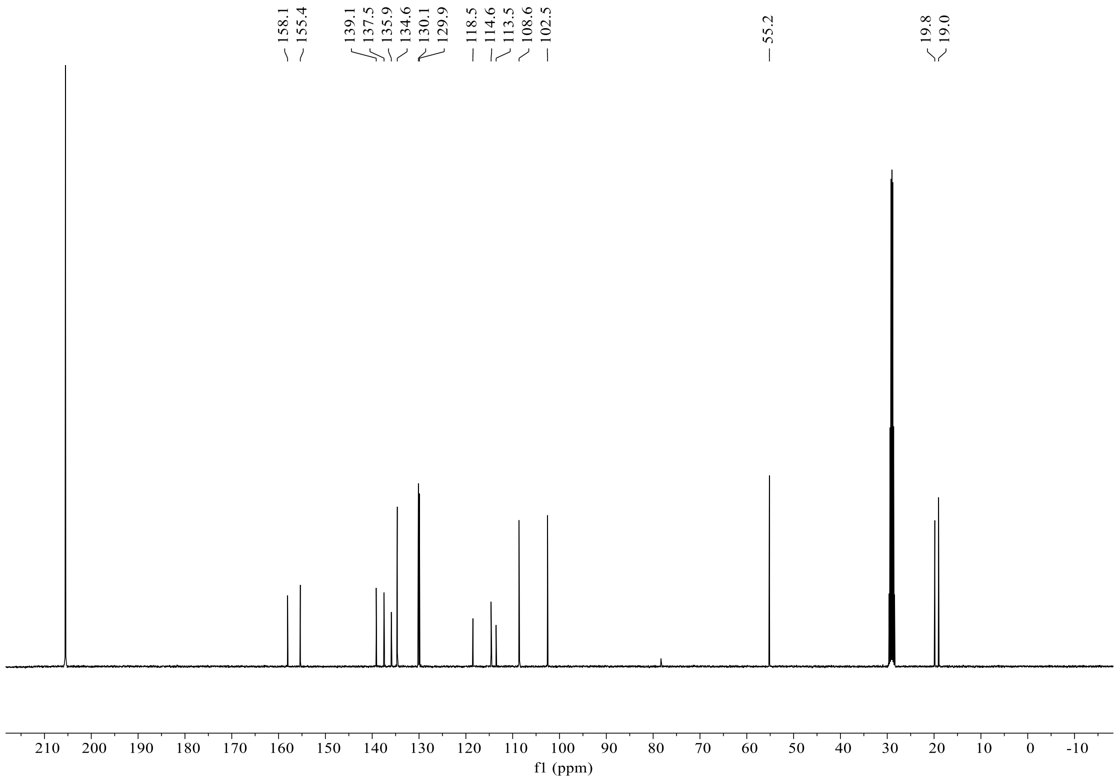

Supplementary Figure 143 HPLC spectra of racemic **3t**

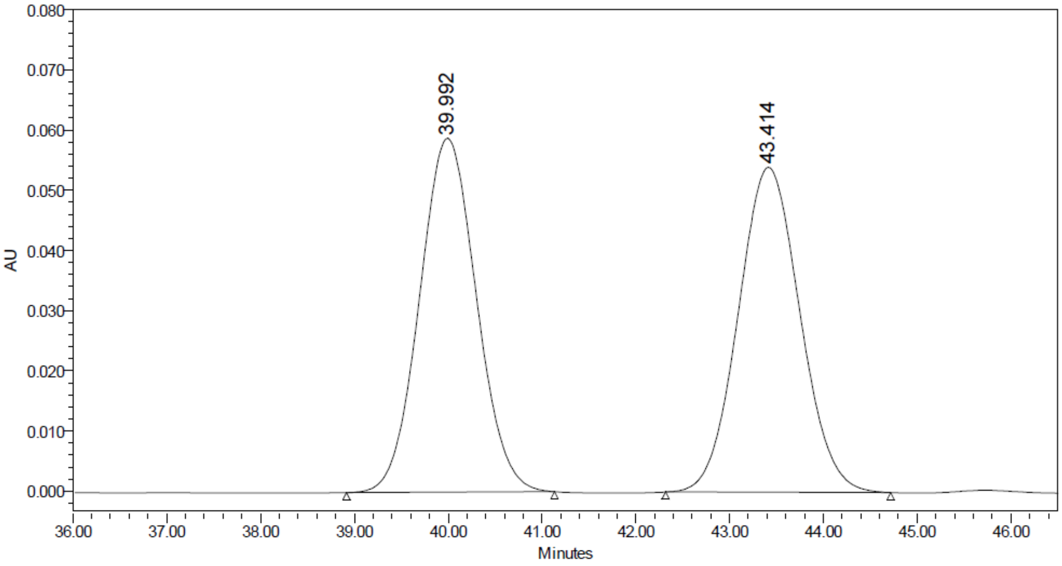

	RT	Area	% Area	Height
1	39.992	2474786	50.07	58774
2	43.414	2467431	49.93	53957

Supplementary Figure 144 HPLC spectra of (*R*)- **3t**

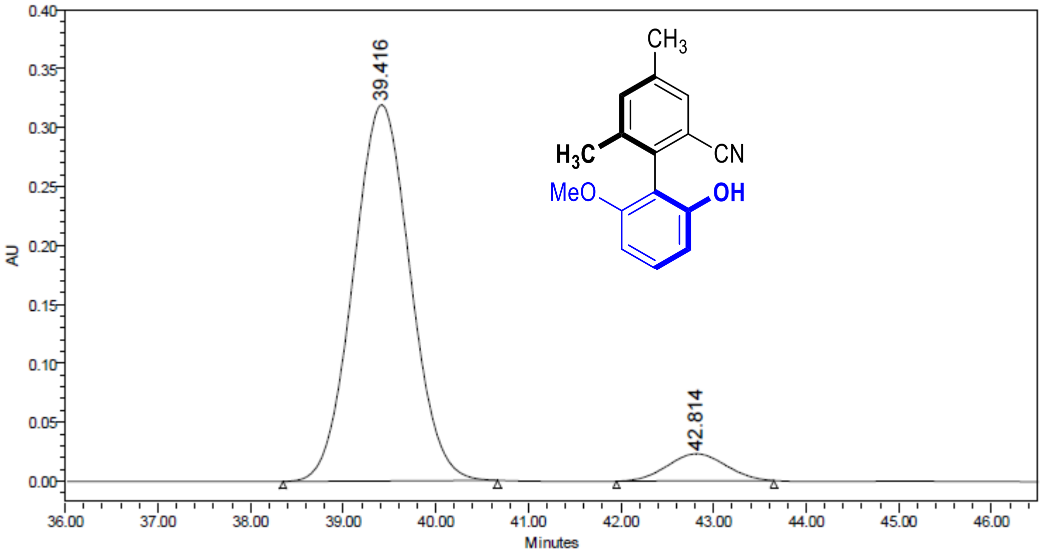

	RT	Area	% Area	Height
1	39.416	13618706	93.11	319263
2	42.814	1007548	6.89	22937

Supplementary Figure 145 ^{1}H NMR (400 MHz, $CDCl_3$) of **3u**

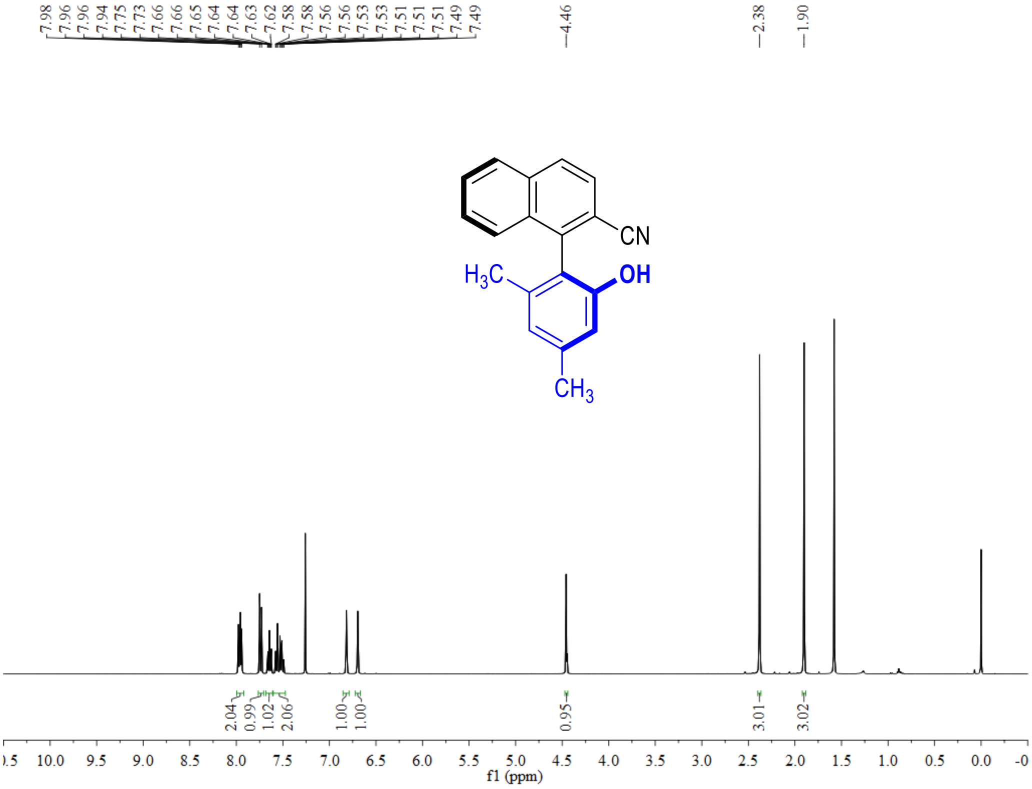

Supplementary Figure 146 ^{13}C NMR (400 MHz, $CDCl_3$) of **3u**

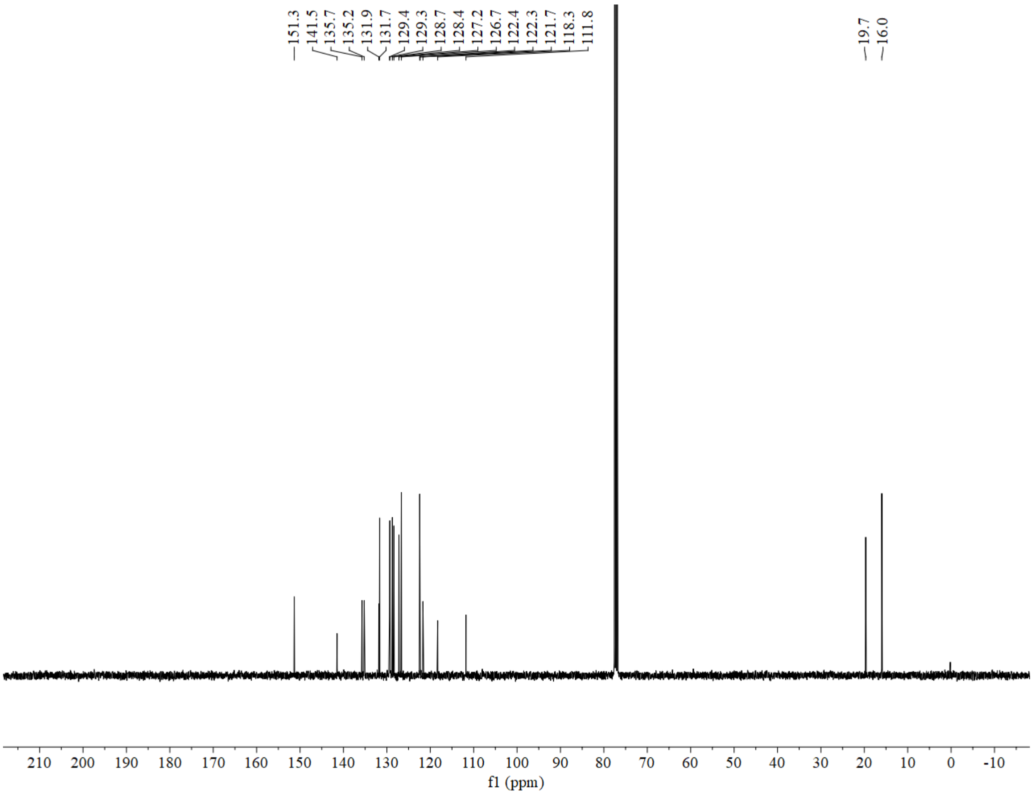

Supplementary Figure 147 HPLC spectra of racemic **3u**

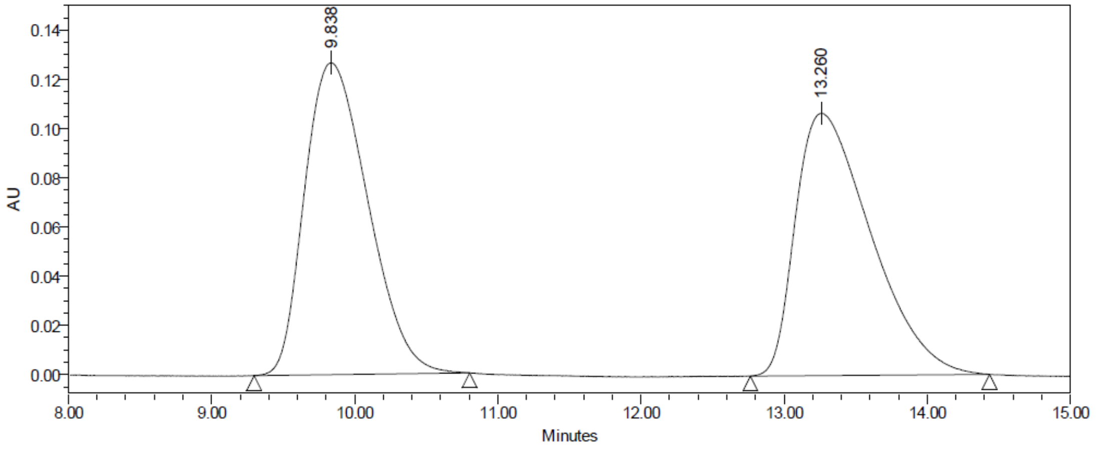

	RT	Area	% Area	Height
1	9.838	3899796	49.95	126861
2	13.260	3907608	50.05	106624

Supplementary Figure 148 HPLC spectra of (*S*)- **3u**

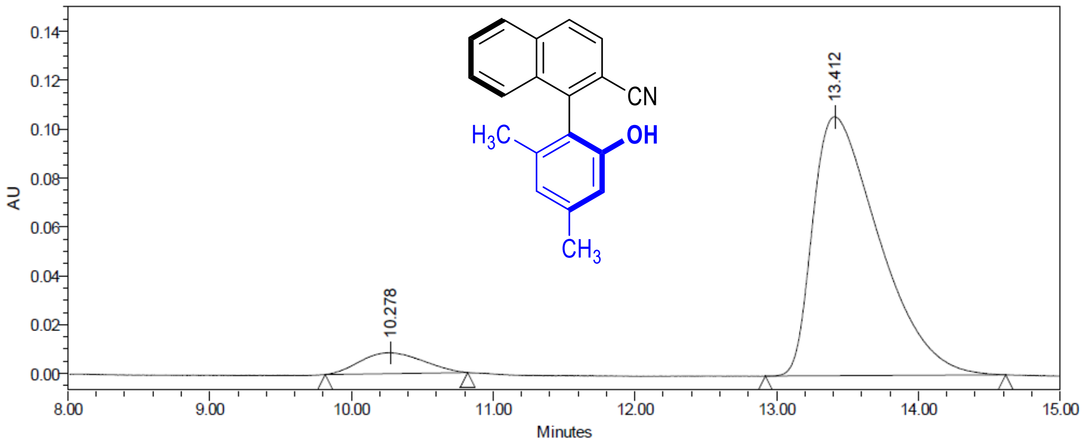

	RT	Area	% Area	Height
1	10.278	261749	7.06	8527
2	13.412	3444256	92.94	105897

Supplementary Figure 149 ^{1}H NMR (400 MHz, Acetone-d_6) of **3v**

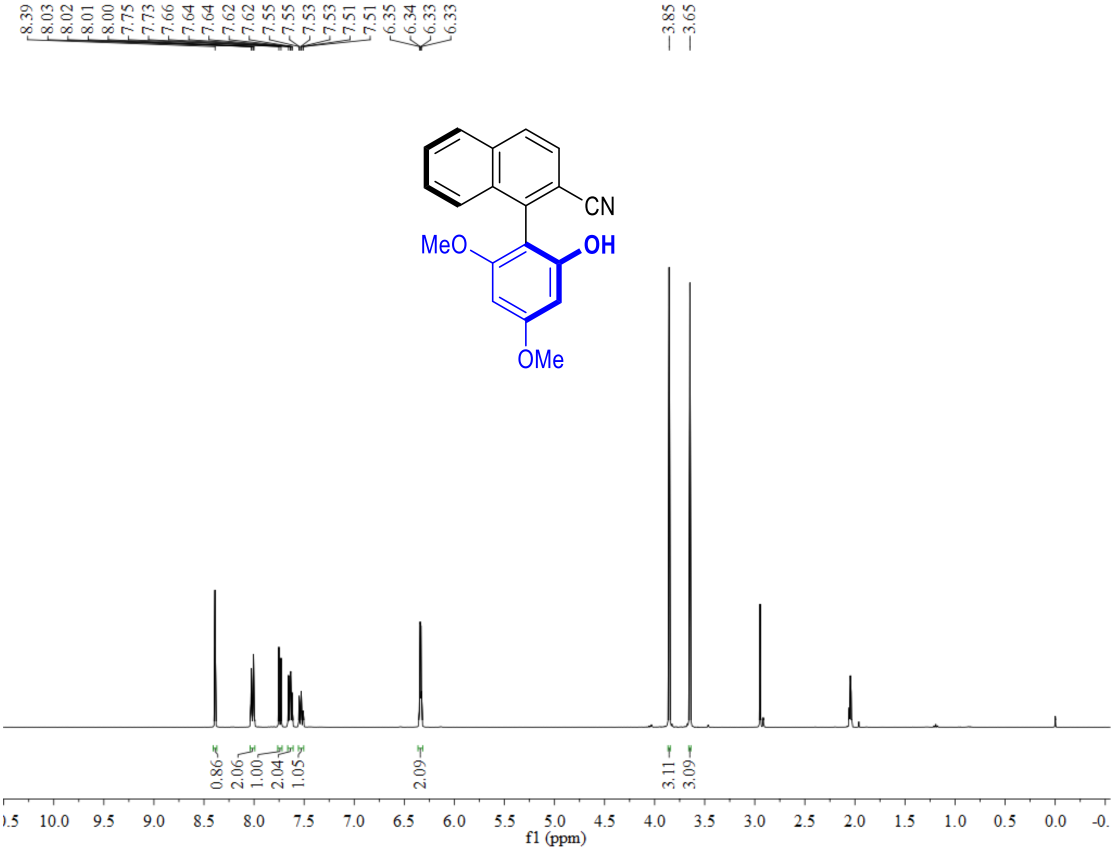

Supplementary Figure 150 ^{13}C NMR (400 MHz, Acetone-d_6) of **3v**

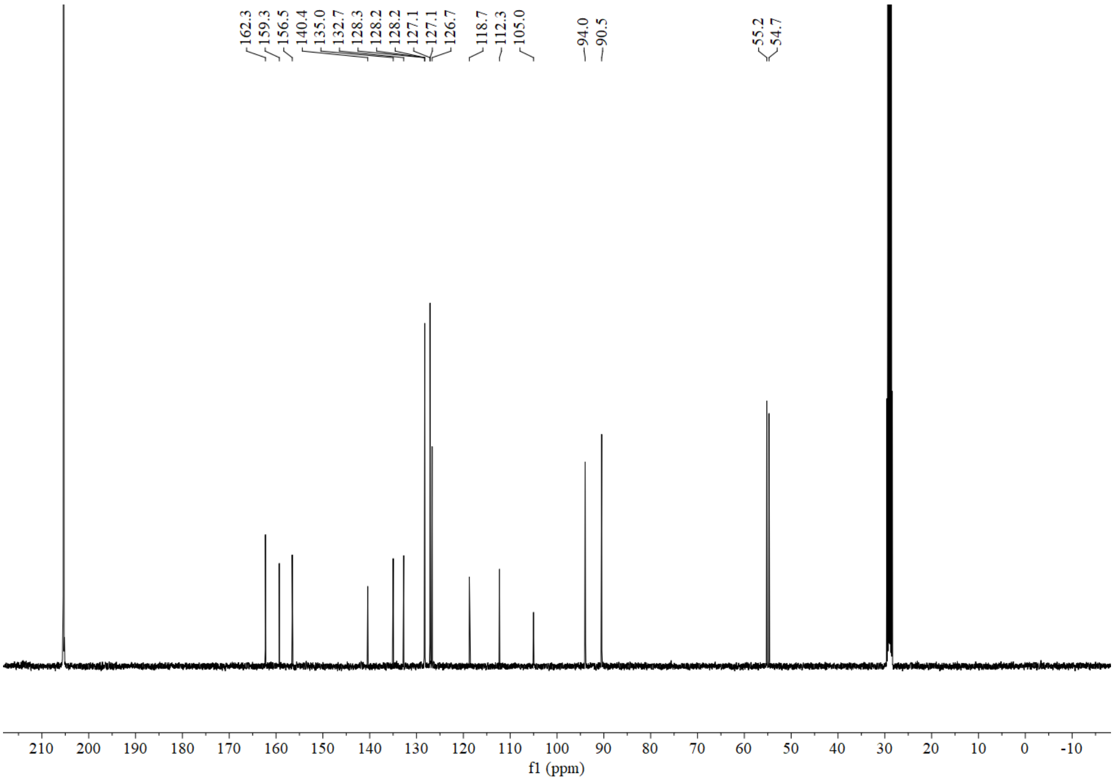

Supplementary Figure 151 HPLC spectra of racemic **3v**

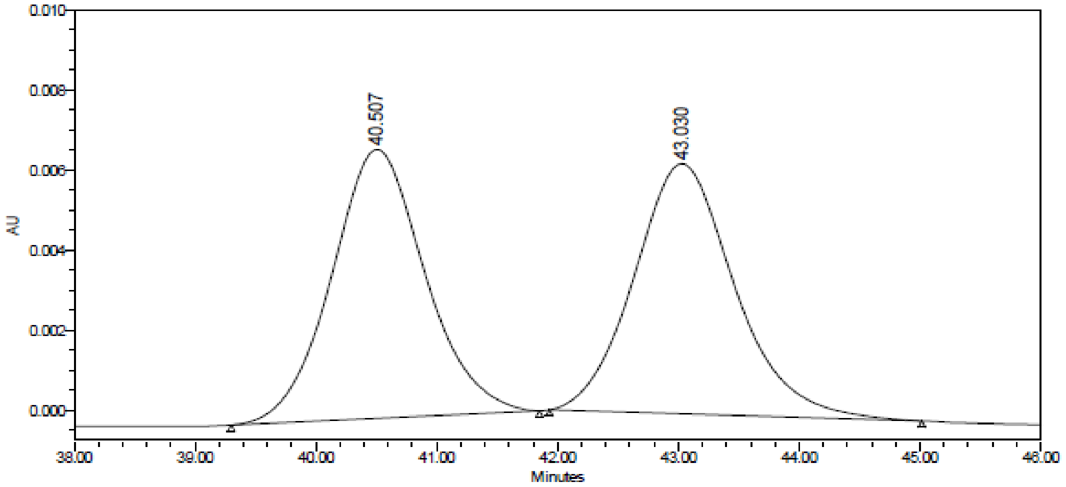

	RT	Area	% Area	Height
1	40.507	359976	49.91	6716
2	43.030	361271	50.09	6245

Supplementary Figure 152 HPLC spectra of (*R*)- **3v**

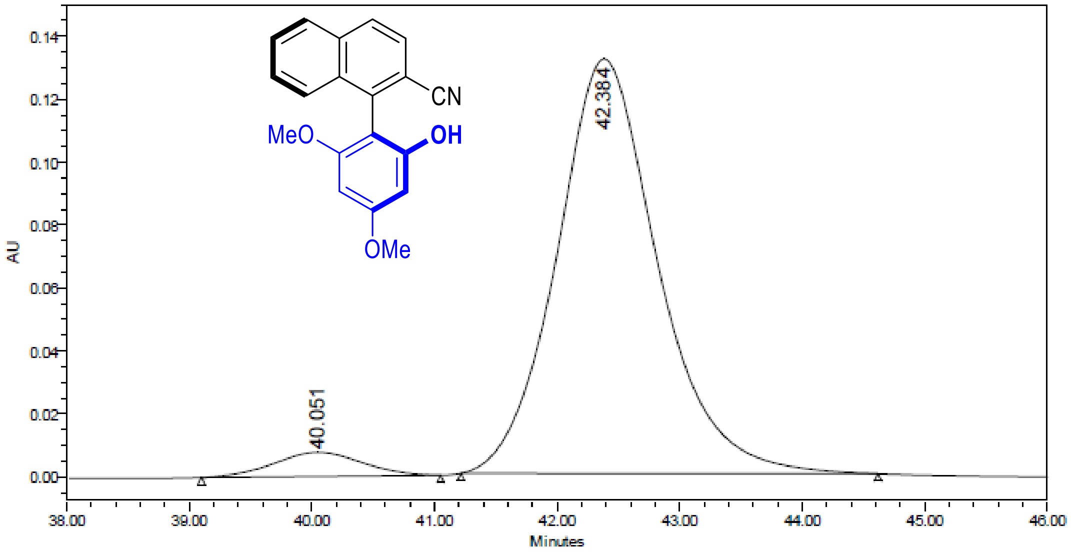

	RT	Area	% Area	Height
1	40.051	382158	4.83	7655
2	42.384	7528730	95.17	131650

Supplementary Figure 153 ^{1}H NMR (400 MHz, $CDCl_3$) of **3w**

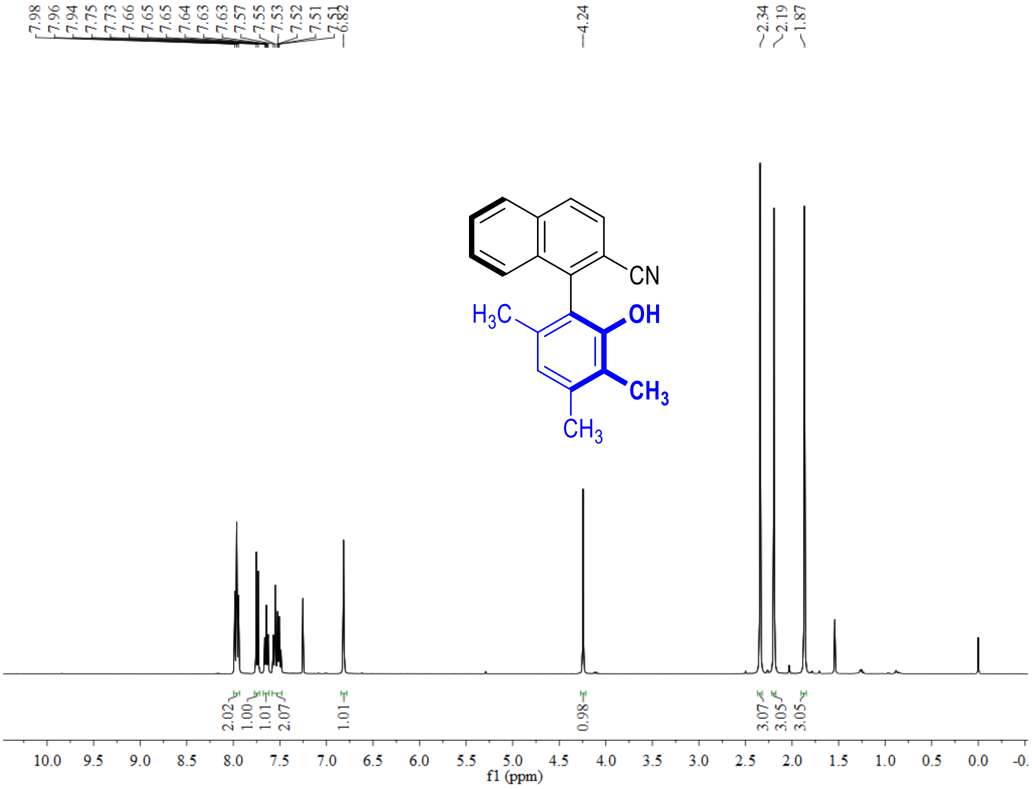

Supplementary Figure 154 ^{13}C NMR (400 MHz, $CDCl_3$) of **3w**

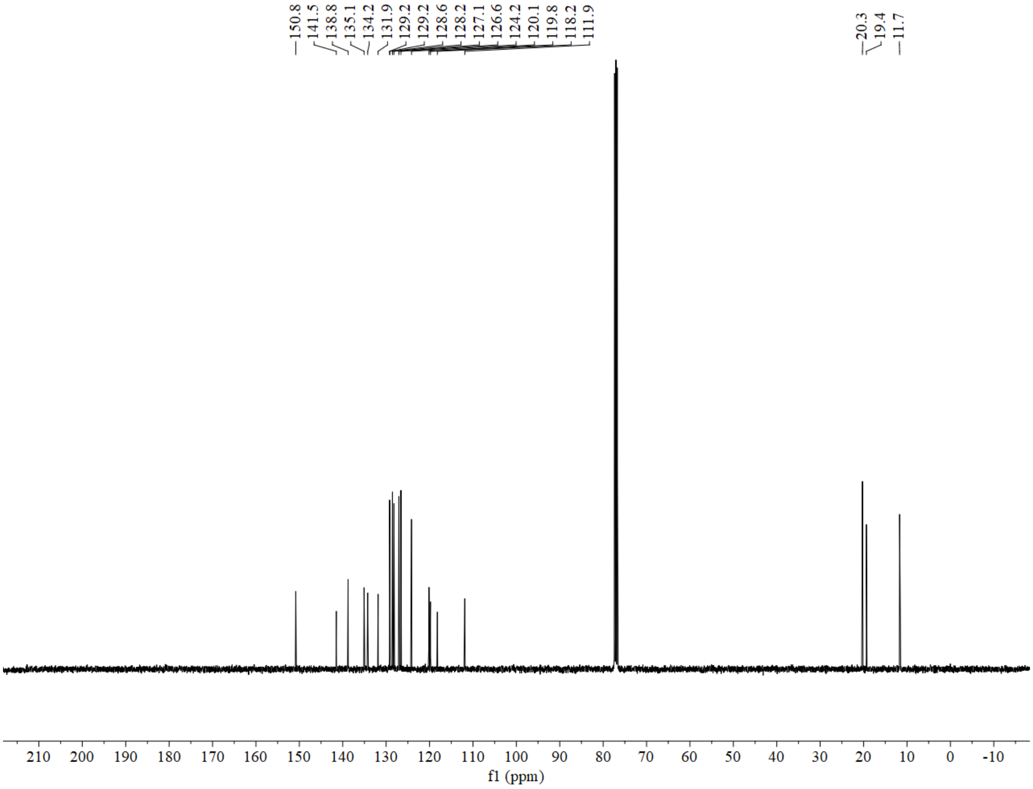

Supplementary Figure 155 HPLC spectra of racemic **3w**

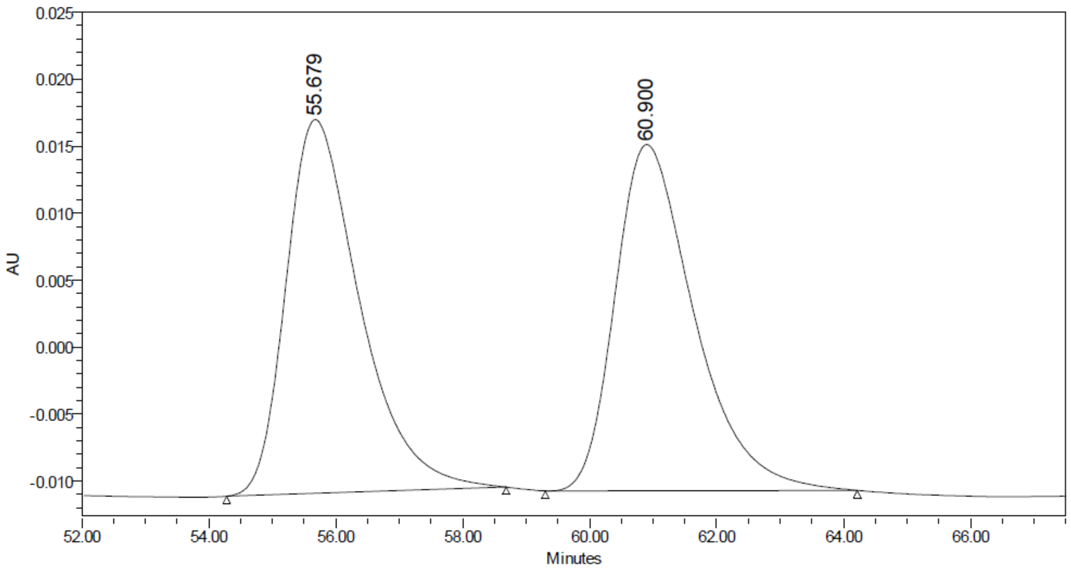

	RT	Area	% Area	Height
1	55.679	2255099	50.15	27904
2	60.900	2242029	49.85	25854

Supplementary Figure 156 HPLC spectra of (*S*)- **3w**

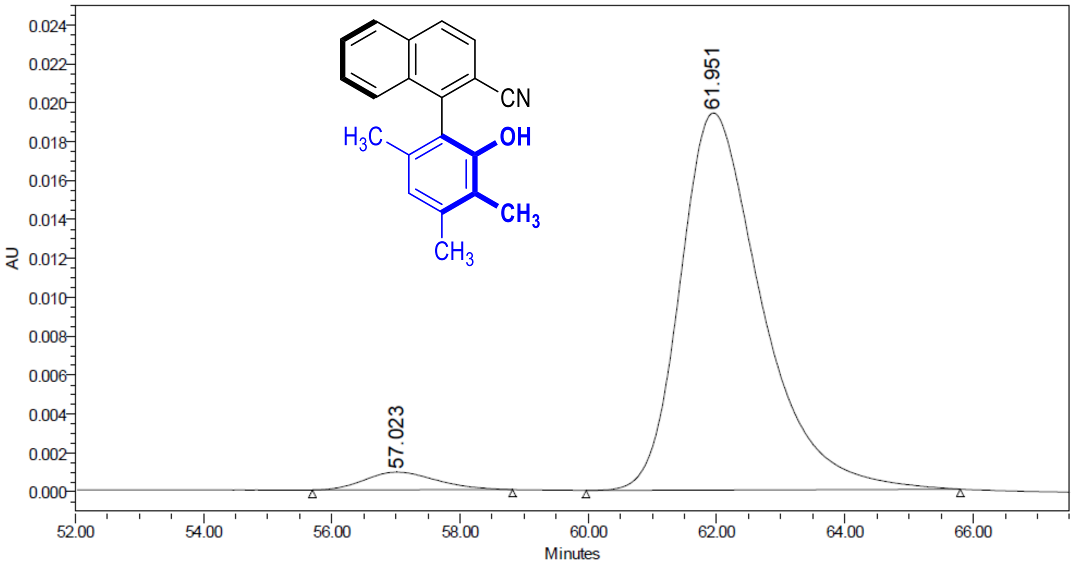

	RT	Area	% Area	Height
1	57.023	70977	3.98	912
2	61.951	1712186	96.02	19385

Supplementary Figure 157 ^{1}H NMR (400 MHz, $CDCl_3$) of **3x**

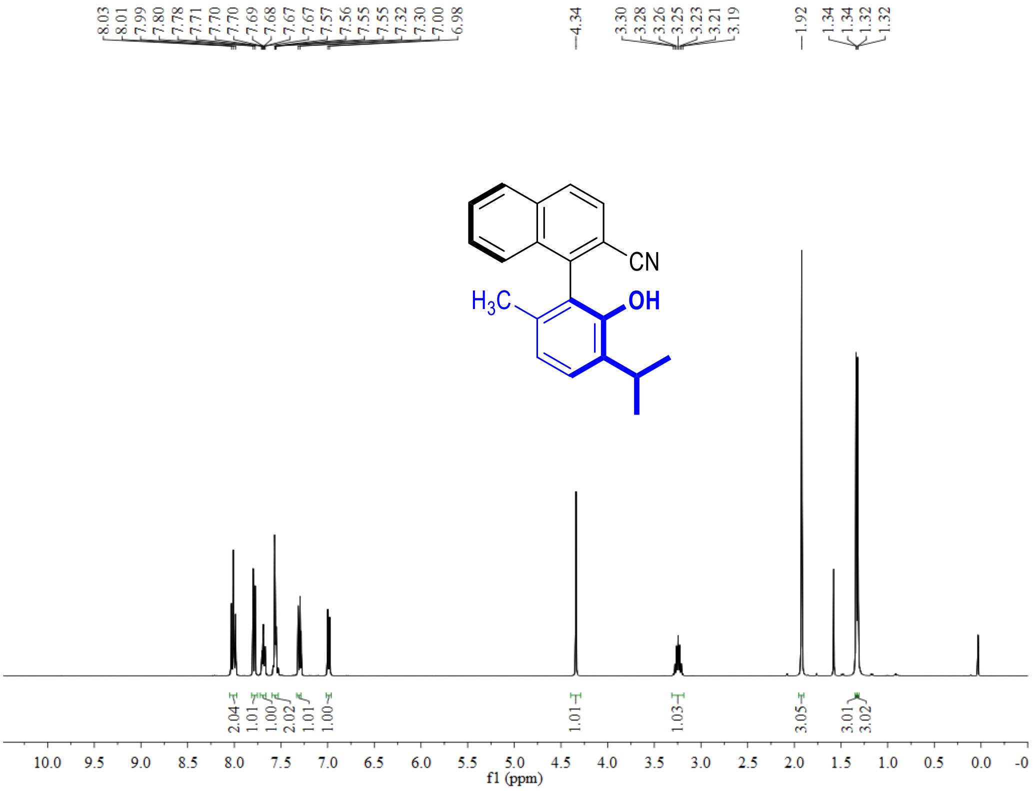

Supplementary Figure 158 ^{13}C NMR (400 MHz, $CDCl_3$) of **3x**

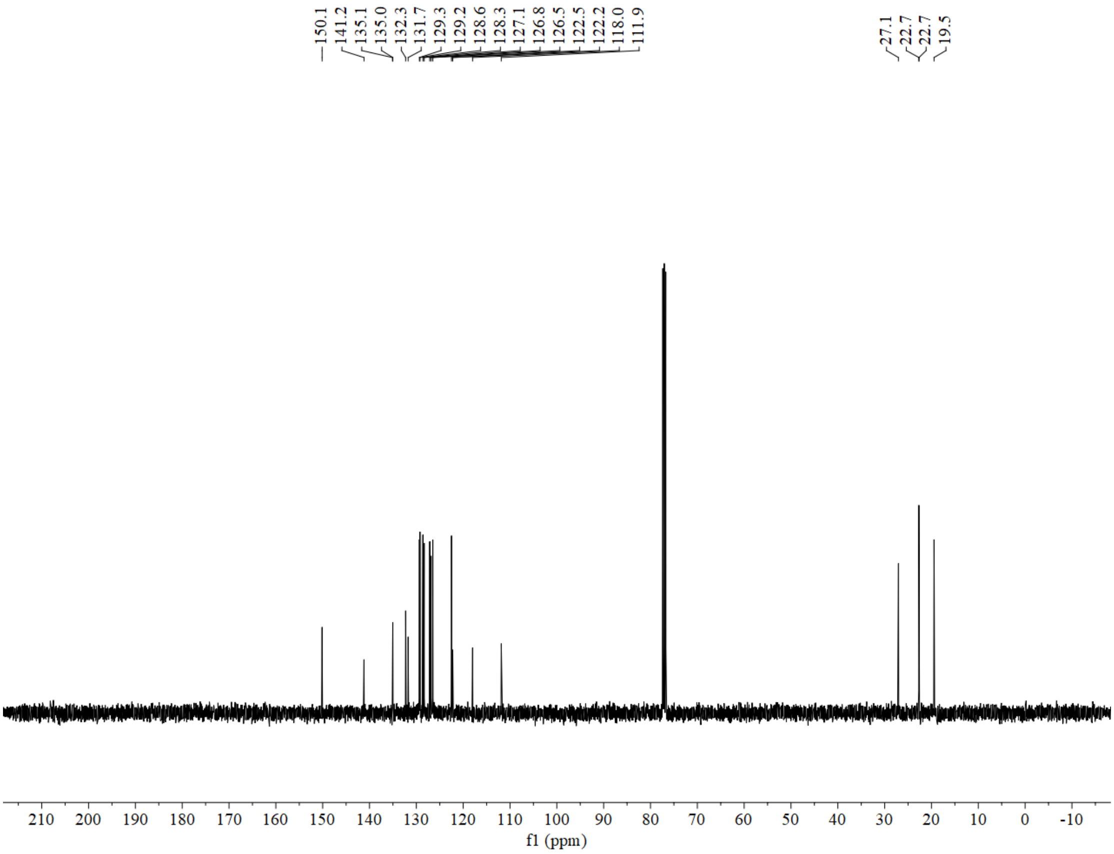

Supplementary Figure 159 HPLC spectra of racemic **3x**

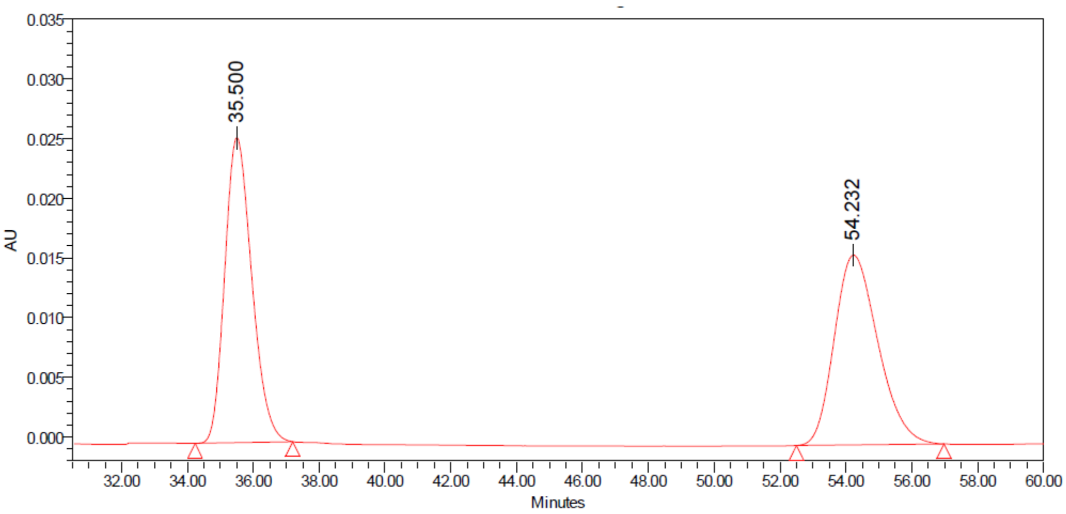

	RT	Area	% Area	Height
1	35.500	1460102	49.95	25547
2	54.232	1463300	50.05	15904

Supplementary Figure 160 HPLC spectra of (*S*)- **3x**

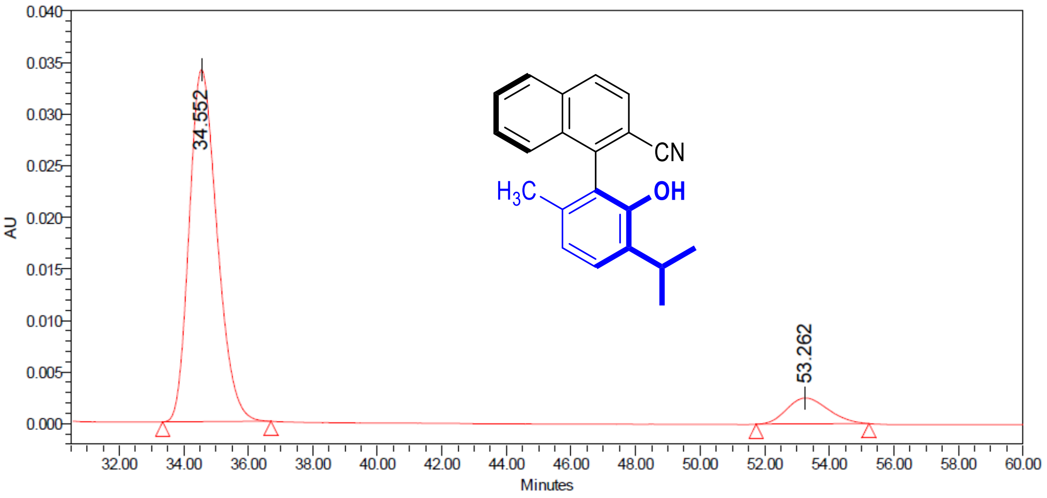

	RT	Area	% Area	Height
1	34.552	2048186	90.16	34119
2	53.262	223602	9.84	2512

Supplementary Figure 161 ^{1}H NMR (400 MHz, $CDCl_3$) of **3y**

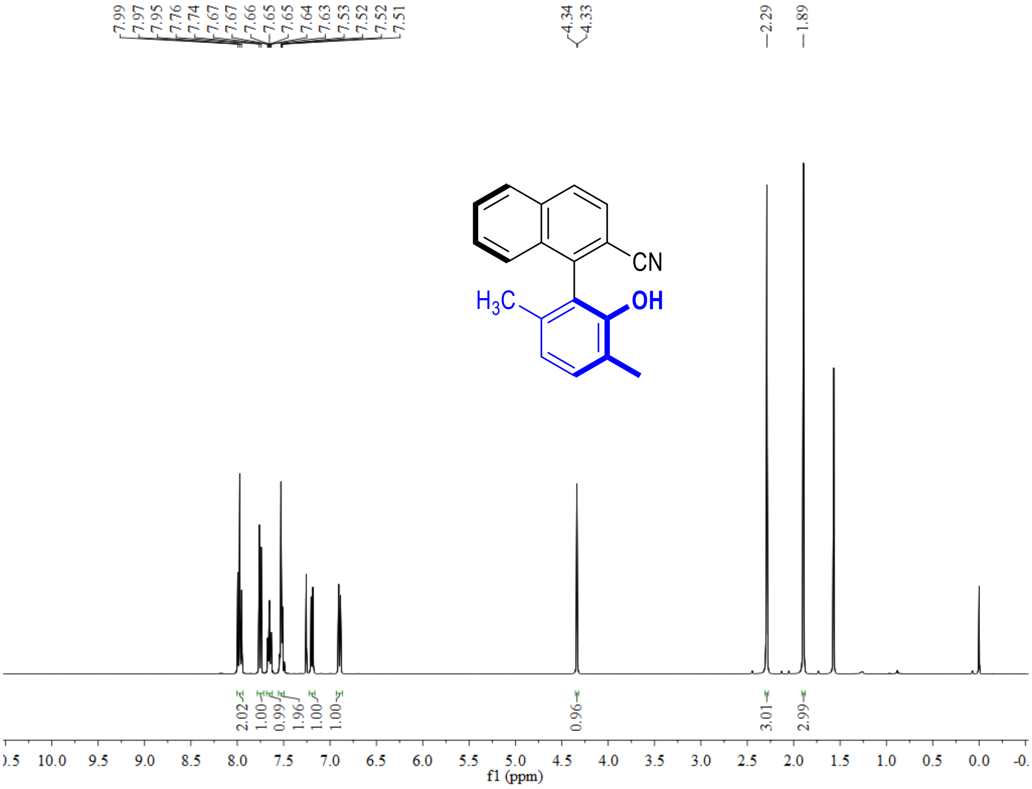

Supplementary Figure 162 ^{13}C NMR (400 MHz, $CDCl_3$) of **3y**

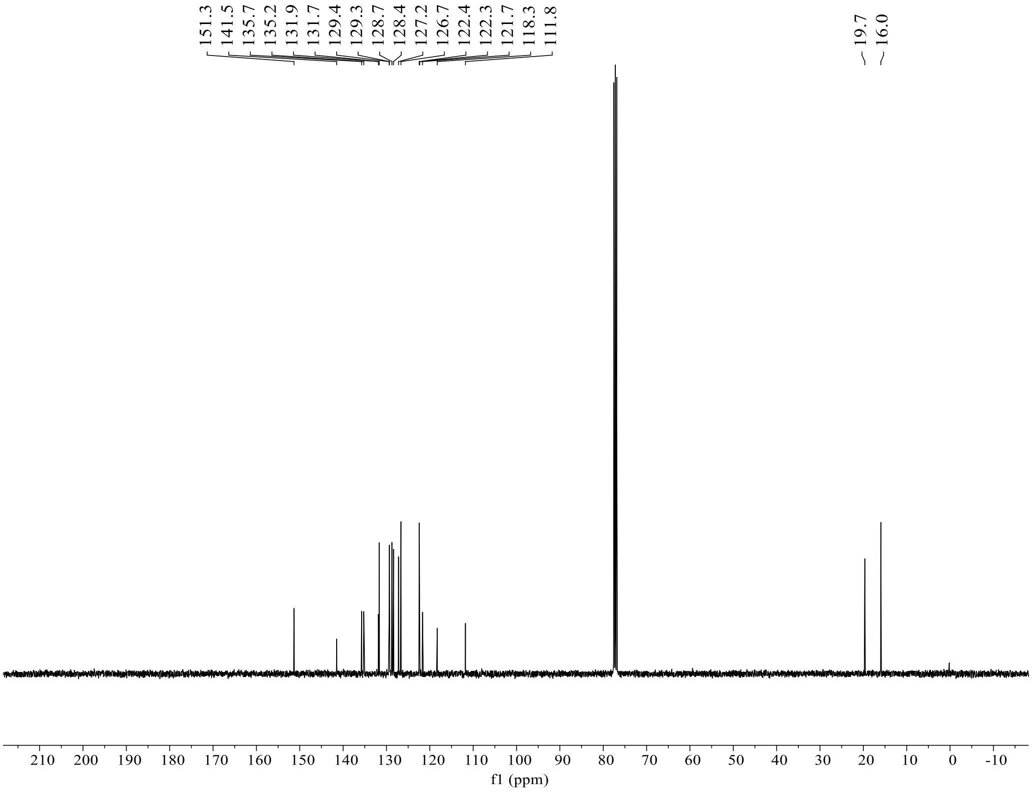

Supplementary Figure 163 HPLC spectra of racemic **3y**

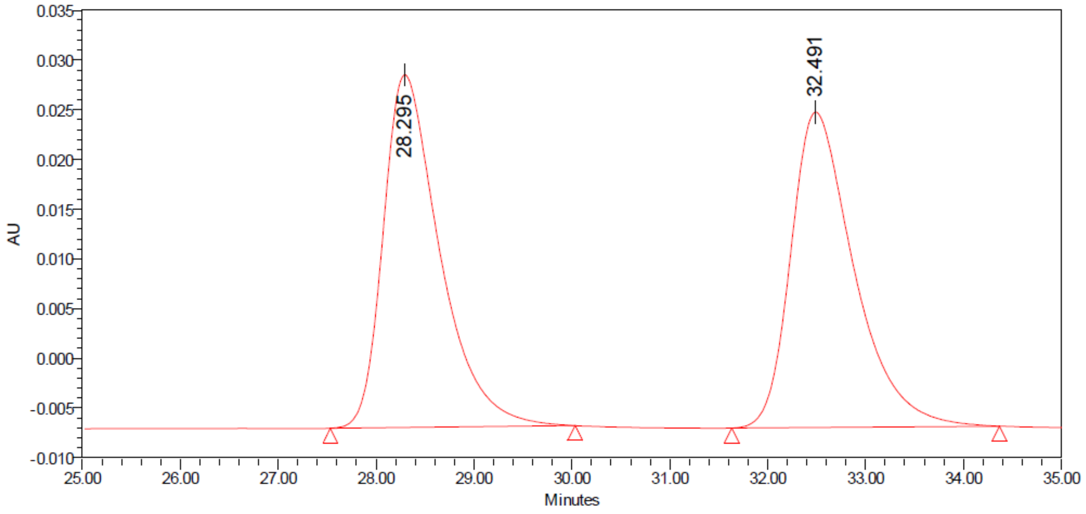

	RT	Area	% Area	Height
1	28.295	1423181	50.00	35461
2	32.491	1423094	50.00	31718

Supplementary Figure 164 HPLC spectra of (*S*)- **3y**

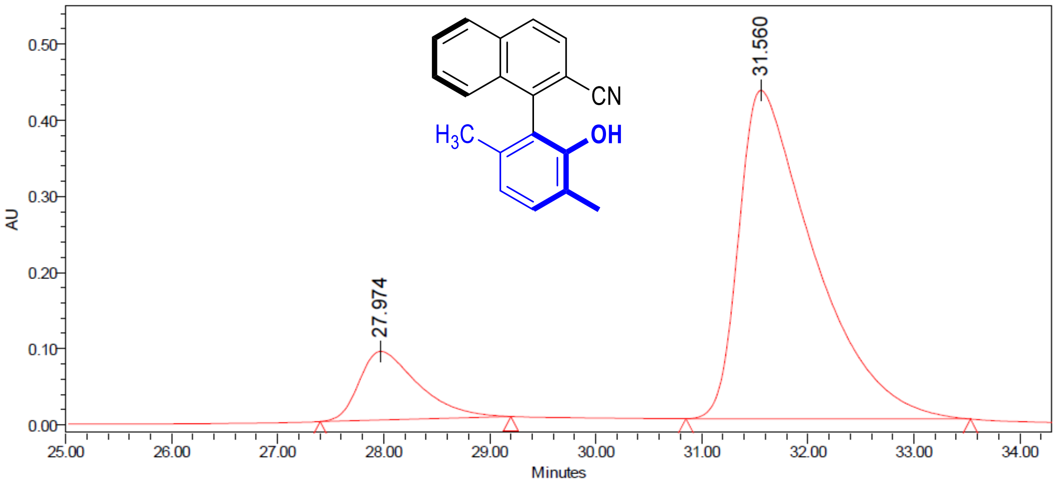

	RT	Area	% Area	Height
1	27.974	3481267	14.05	90106
2	31.560	21299756	85.95	431532

Supplementary Figure 165 ^{1}H NMR (400 MHz, $CDCl_3$) of **5**

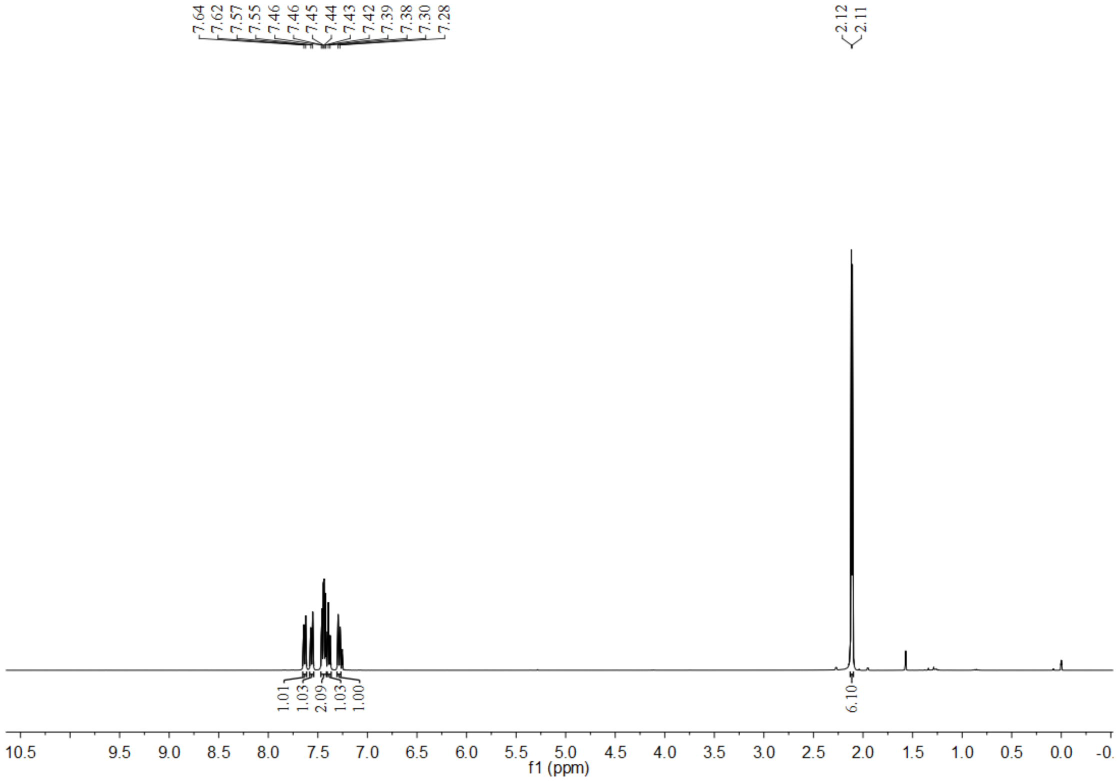

Supplementary Figure 166 ^{13}C NMR (400 MHz, $CDCl_3$) of **5**

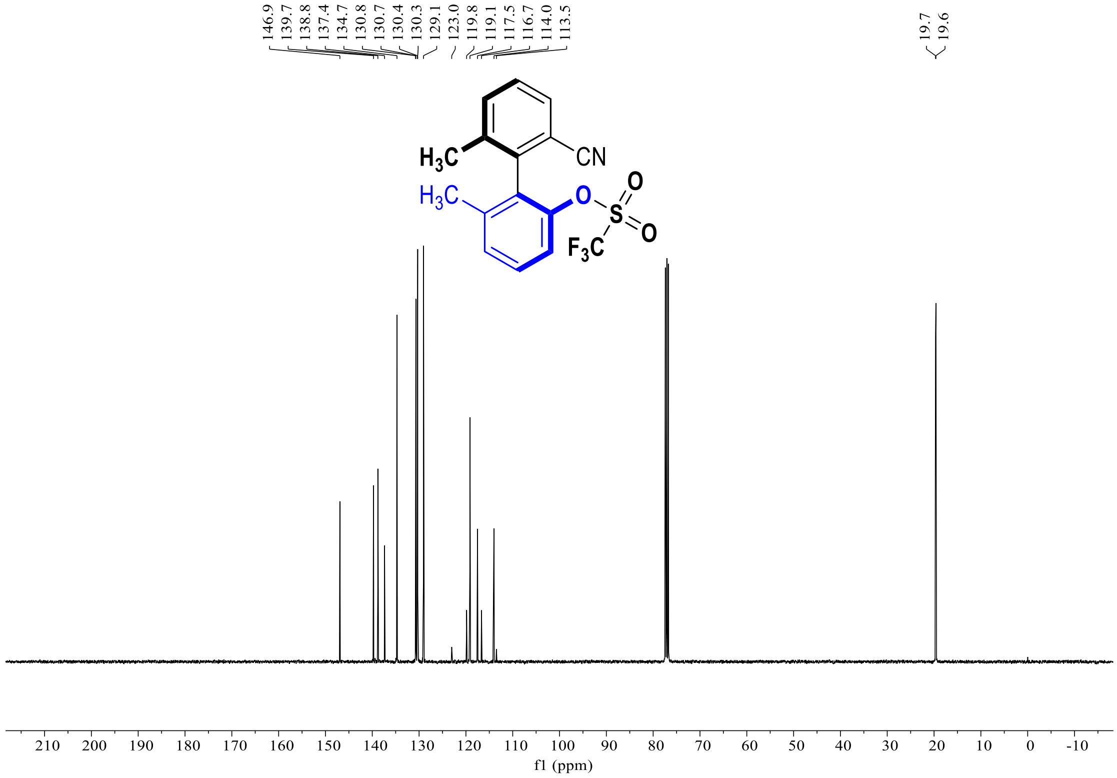

Supplementary Figure 167 ^{19}F NMR (400 MHz, $CDCl_3$) of **5**

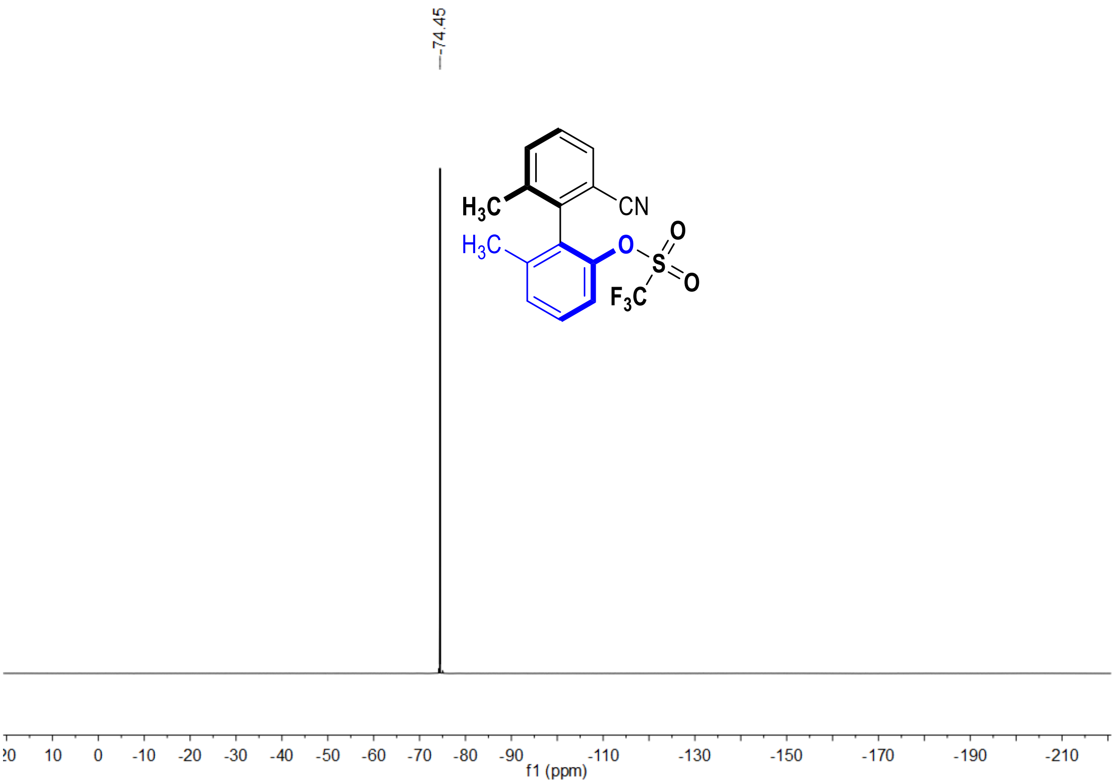

Supplementary Figure 168 HPLC spectra of racemic **5**

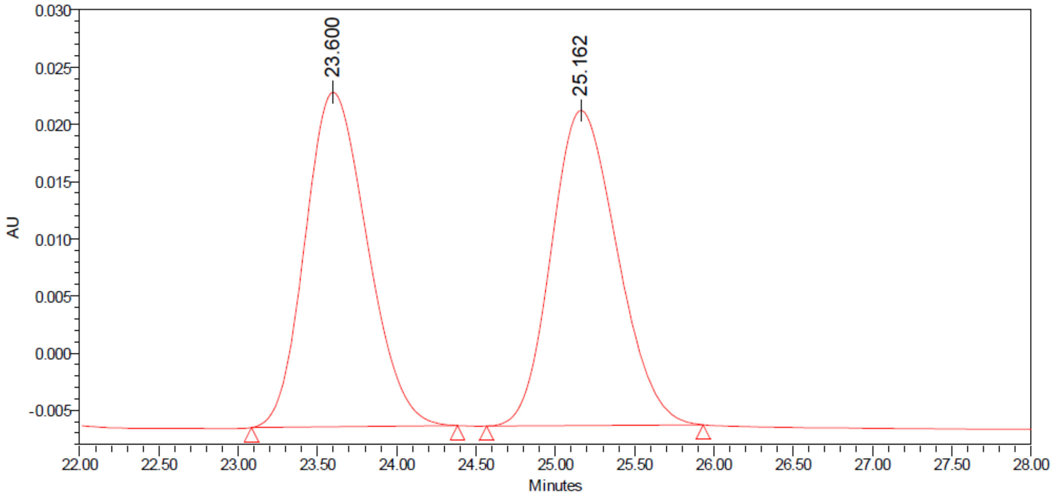

	RT	Area	% Area	Height
1	23.600	795736	50.02	29250
2	25.162	794994	49.98	27550

Supplementary Figure 169 HPLC spectra of (*S*)- **5**

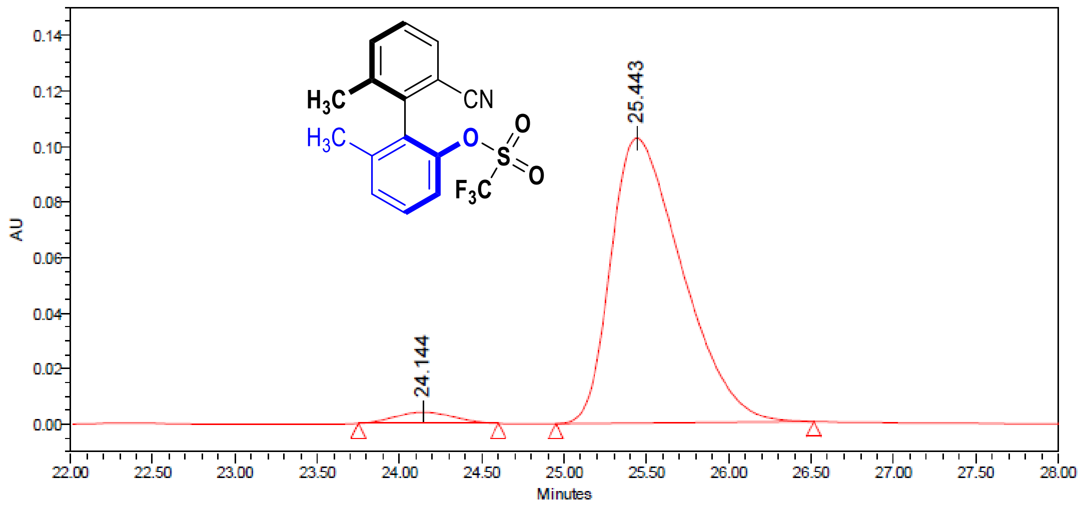

	RT	Area	% Area	Height
1	24.144	98559	3.10	4042
2	25.443	3081979	96.90	102526

Supplementary Figure 70 ^{1}H NMR (400 MHz, $CDCl_3$) of **6**

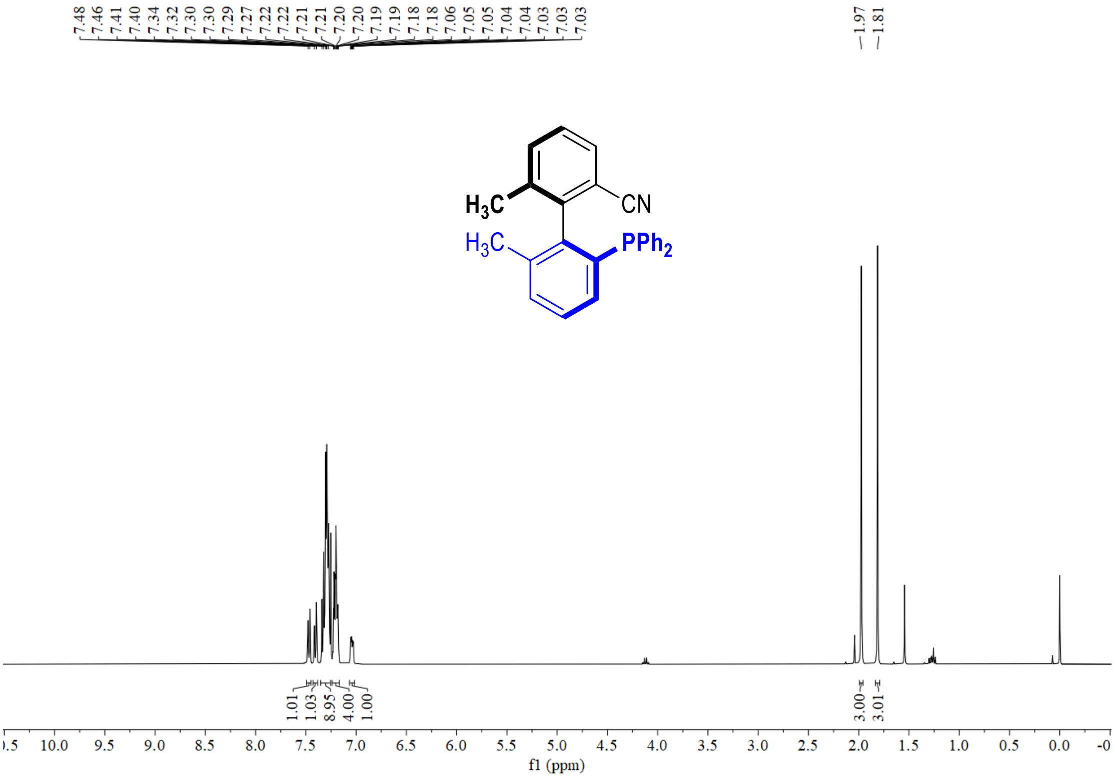

Supplementary Figure 171 ^{13}C NMR (400 MHz, $CDCl_3$) of **6**

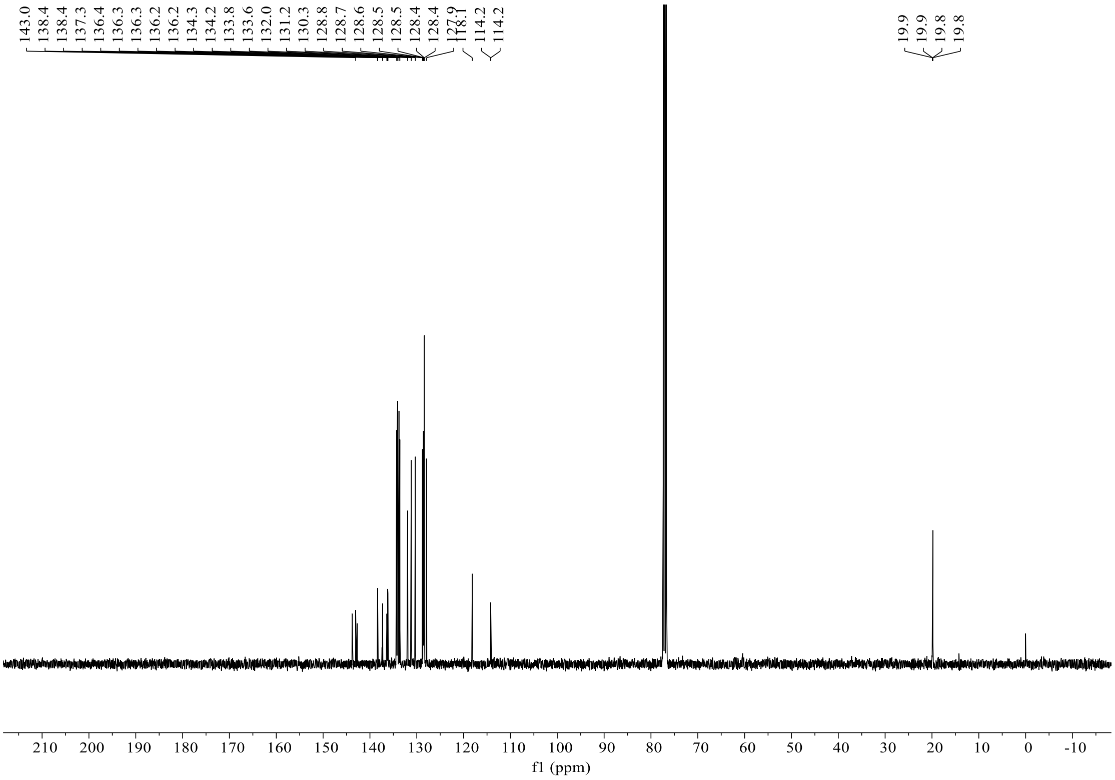

Supplementary Figure 172 ^{31}P NMR (400 MHz, $CDCl_3$) of **6**

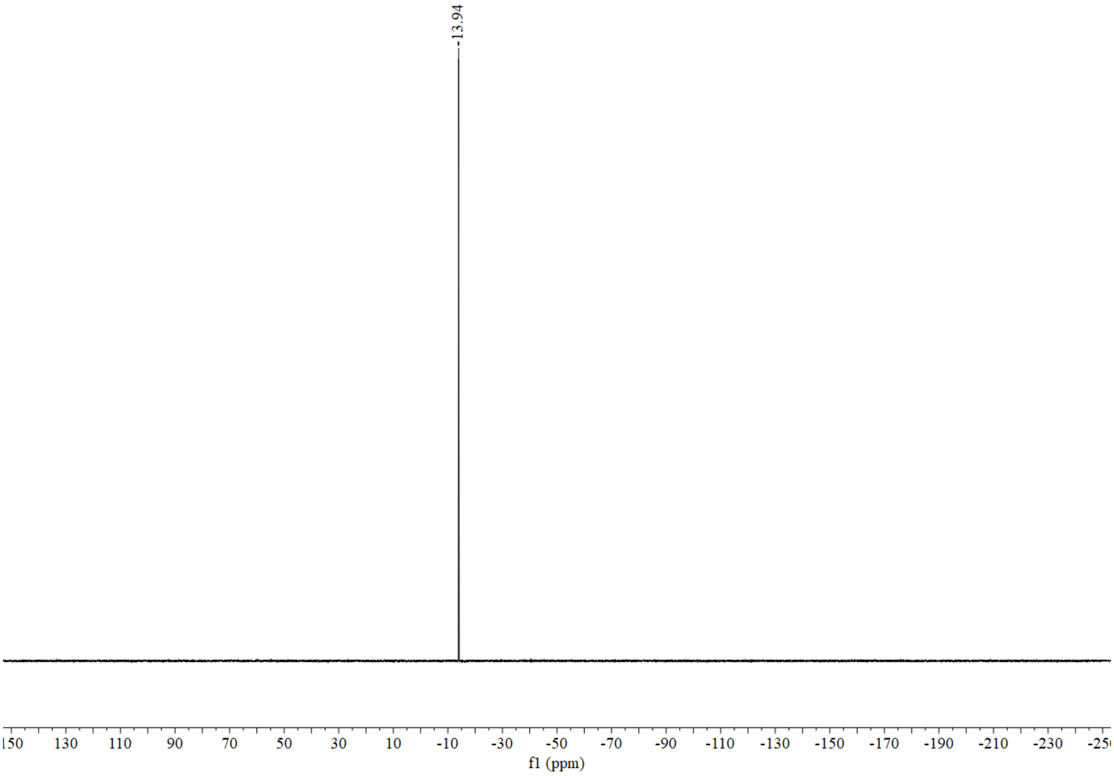

Supplementary Figure 173 HPLC spectra of racemic **6**

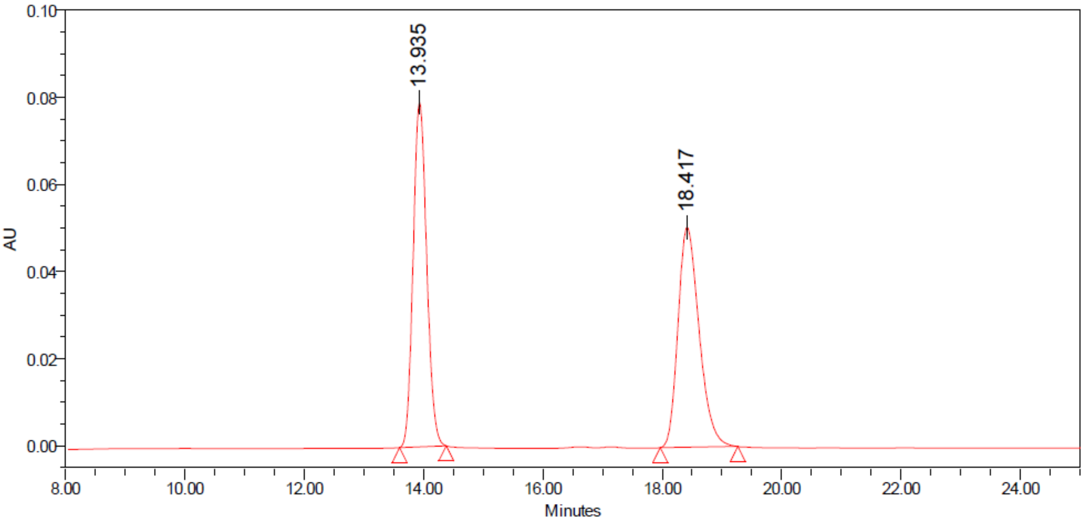

	RT	Area	% Area	Height
1	13.935	1245186	50.07	79122
2	18.417	1241801	49.93	50506

Supplementary Figure 174 HPLC spectra of (*S*)- **6**

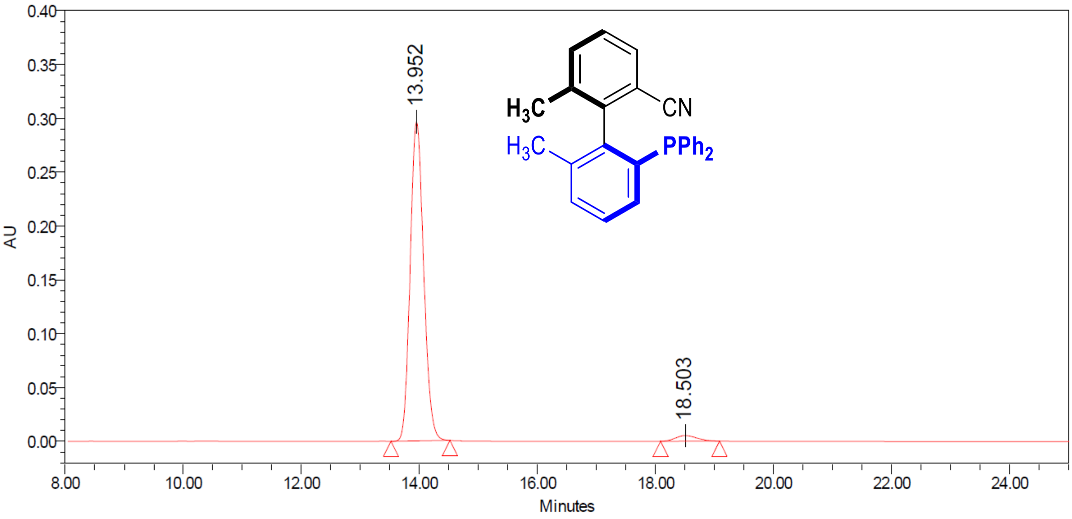

	RT	Area	% Area	Height
1	13.952	4699379	97.28	296779
2	18.503	131636	2.72	5279

Supplementary Figure 175 ^{1}H NMR (400 MHz, $CDCl_3$) of **7**

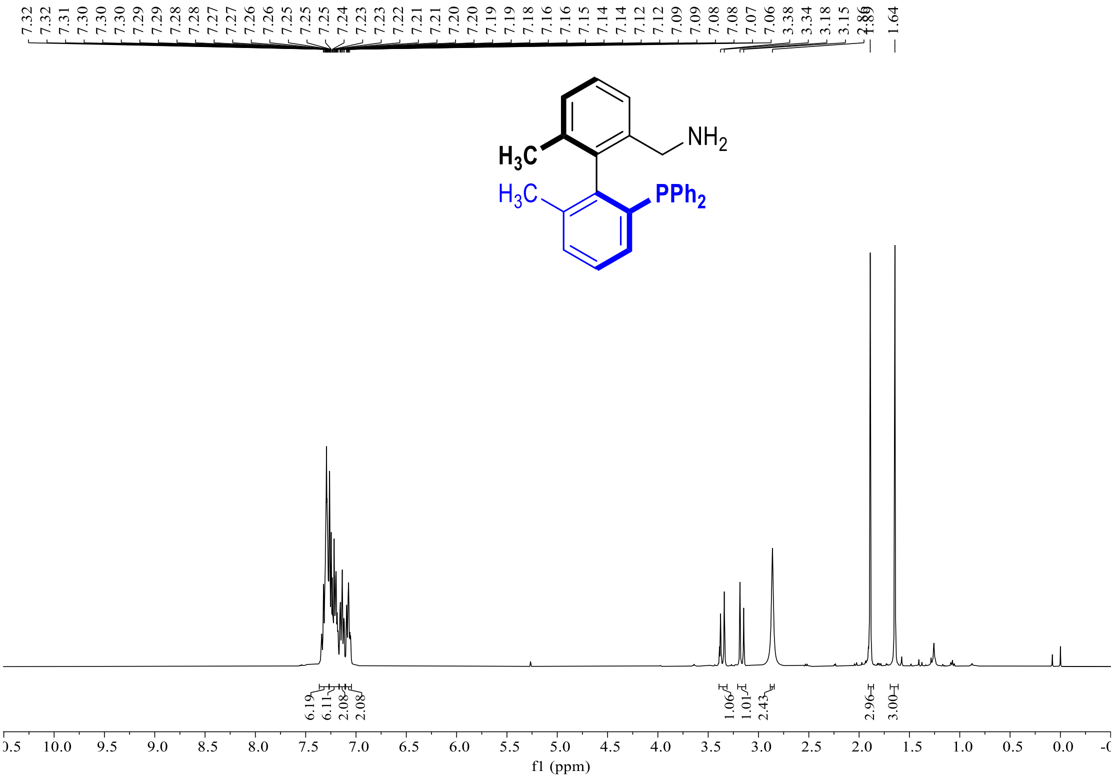

Supplementary Figure 176 ^{13}C NMR (400 MHz, $CDCl_3$) of **7**

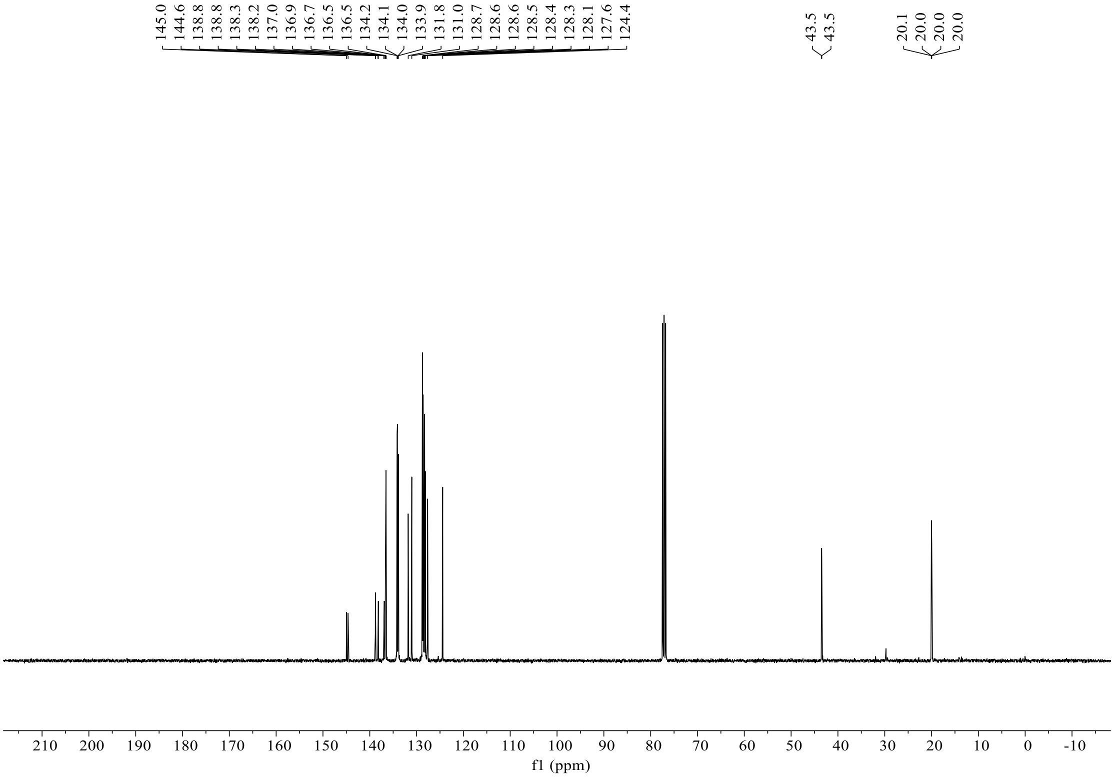

Supplementary Figure 177 ^{31}P NMR (400 MHz, $CDCl_3$) of **7**

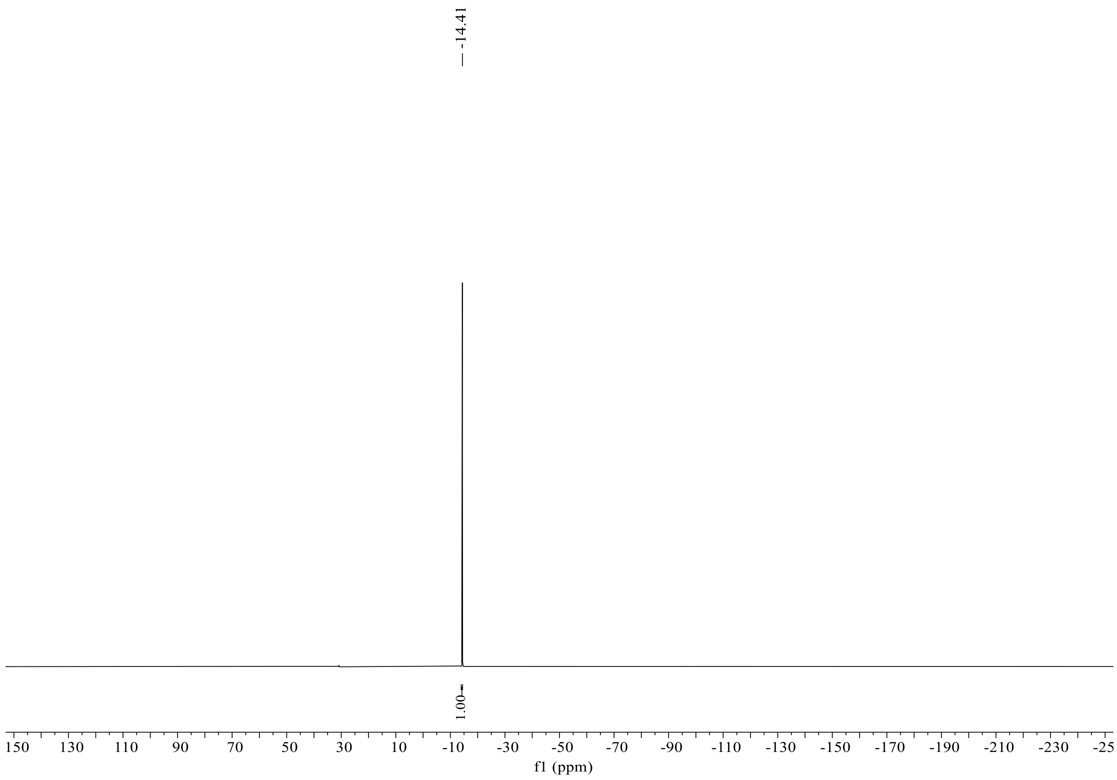

Supplementary Figure 178 ^{1}H NMR (400 MHz, $CDCl_3$) of **8**

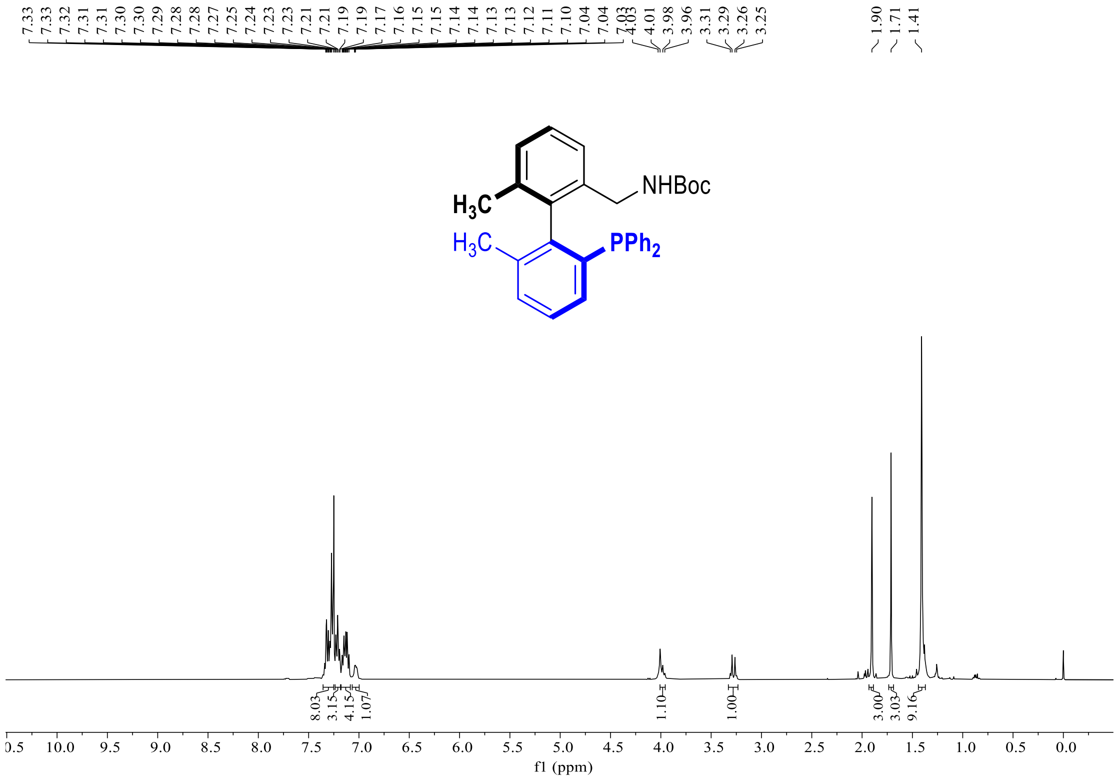

Supplementary Figure 179 ^{13}C NMR (400 MHz, $CDCl_3$) of **8**

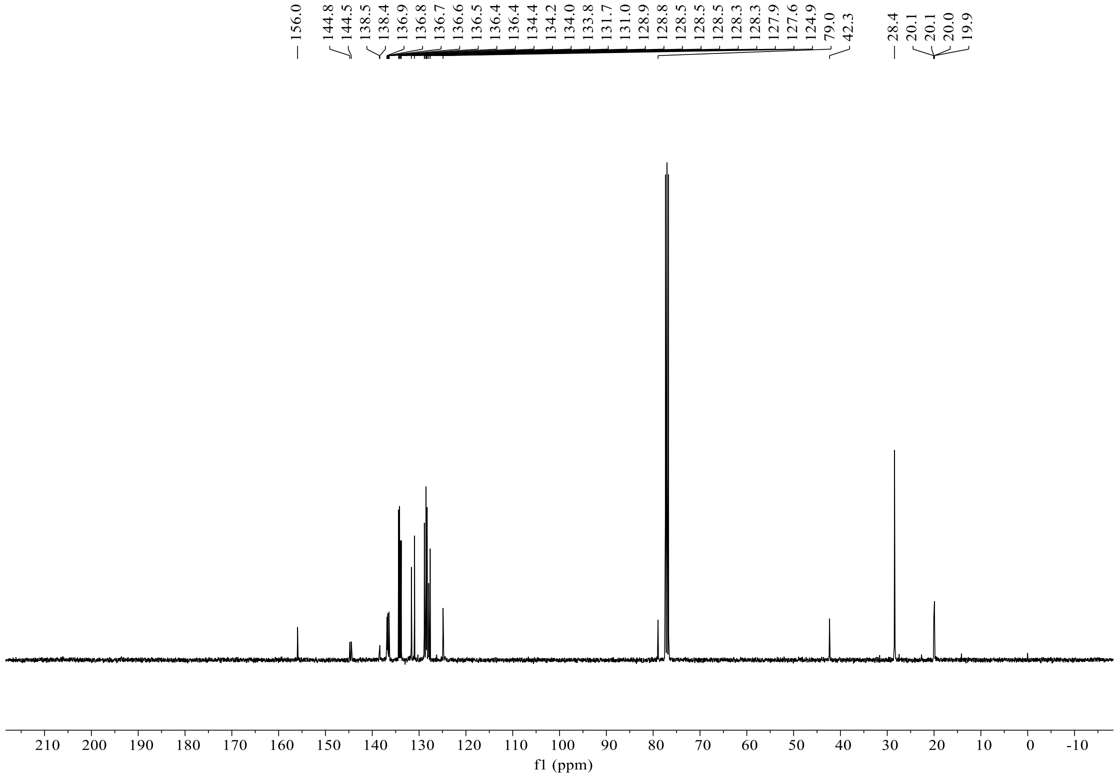

Supplementary Figure 180 ^{31}P NMR (400 MHz, $CDCl_3$) of **8**

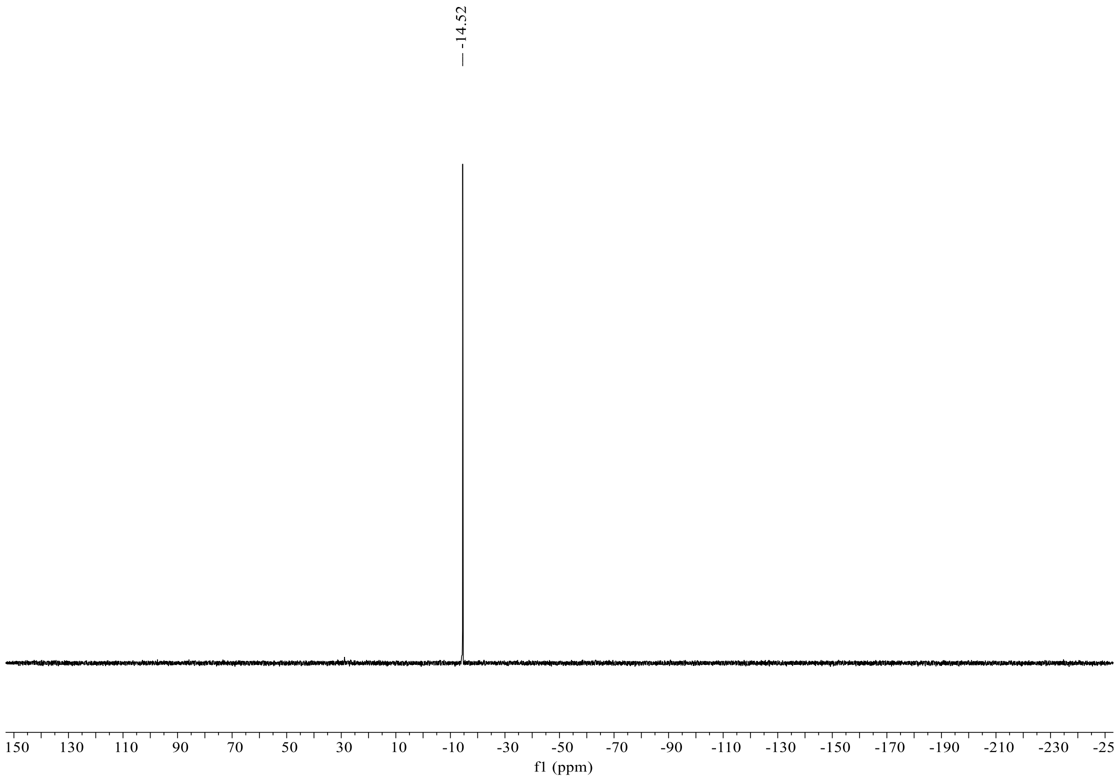

Supplementary Figure 181 HPLC spectra of racemic **8**

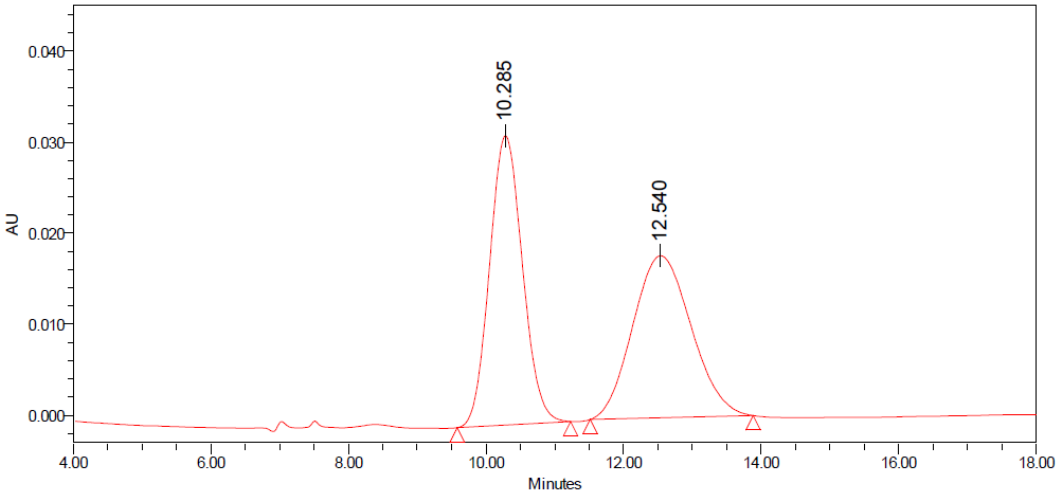

	RT	Area	% Area	Height
1	10.285	1054511	50.09	31770
2	12.540	1050857	49.91	17801

Supplementary Figure 182 HPLC spectra of (*S*)- **8**

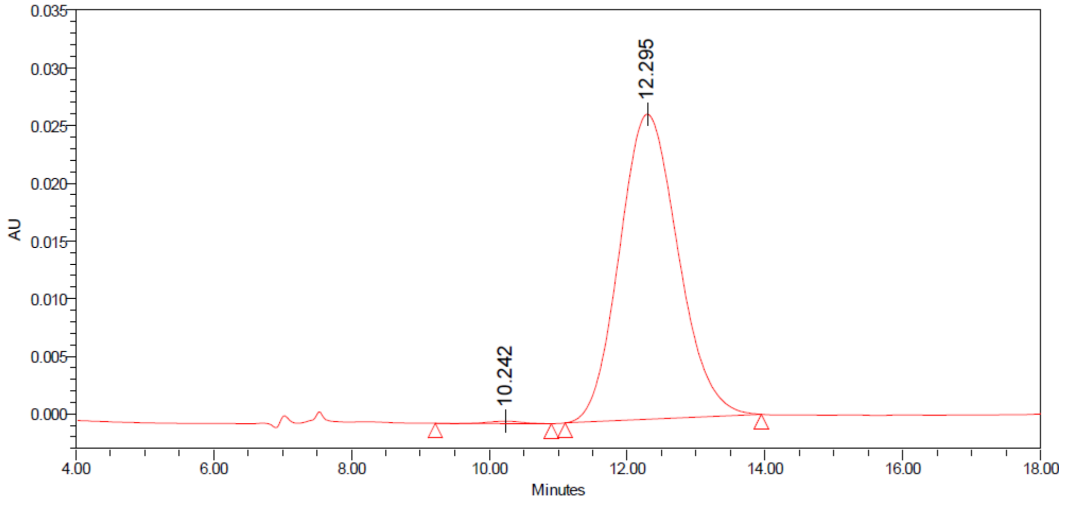

	RT	Area	% Area	Height
1	10.242	7243	0.46	204
2	12.295	1564162	99.54	26438

Supplementary Figure 183 ^{1}H NMR (400 MHz, Acetone-d_6) of **9**

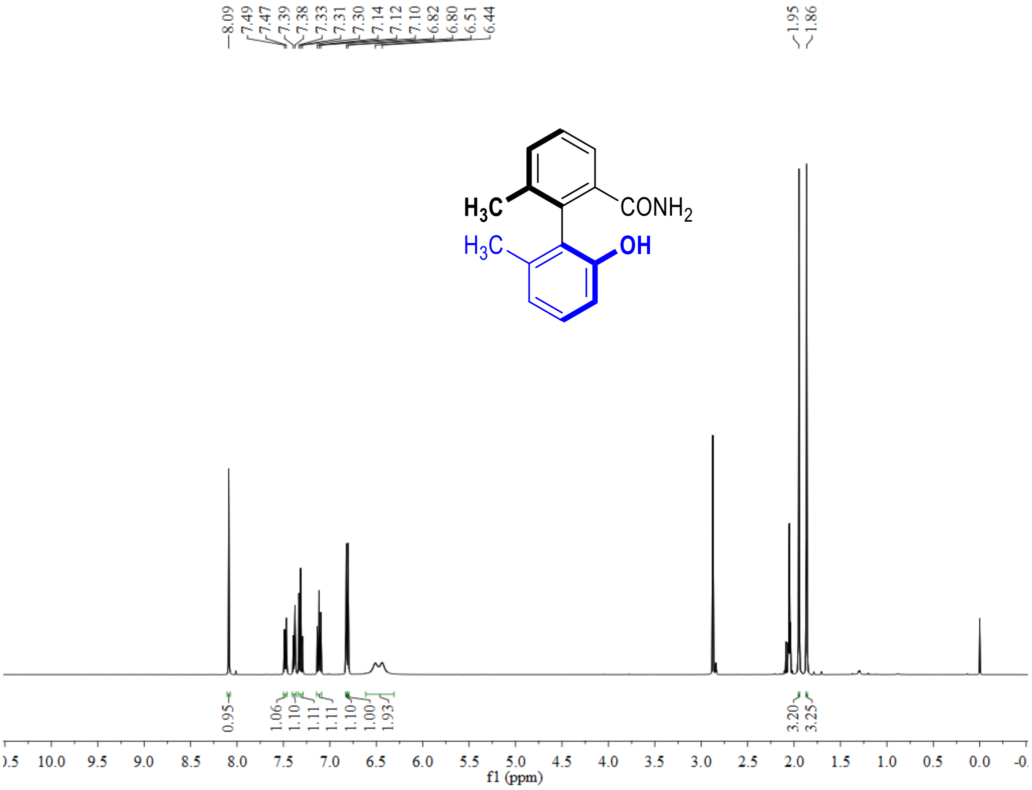

Supplementary Figure 184 ^{13}C NMR (400 MHz, Acetone-d_6) of **9**

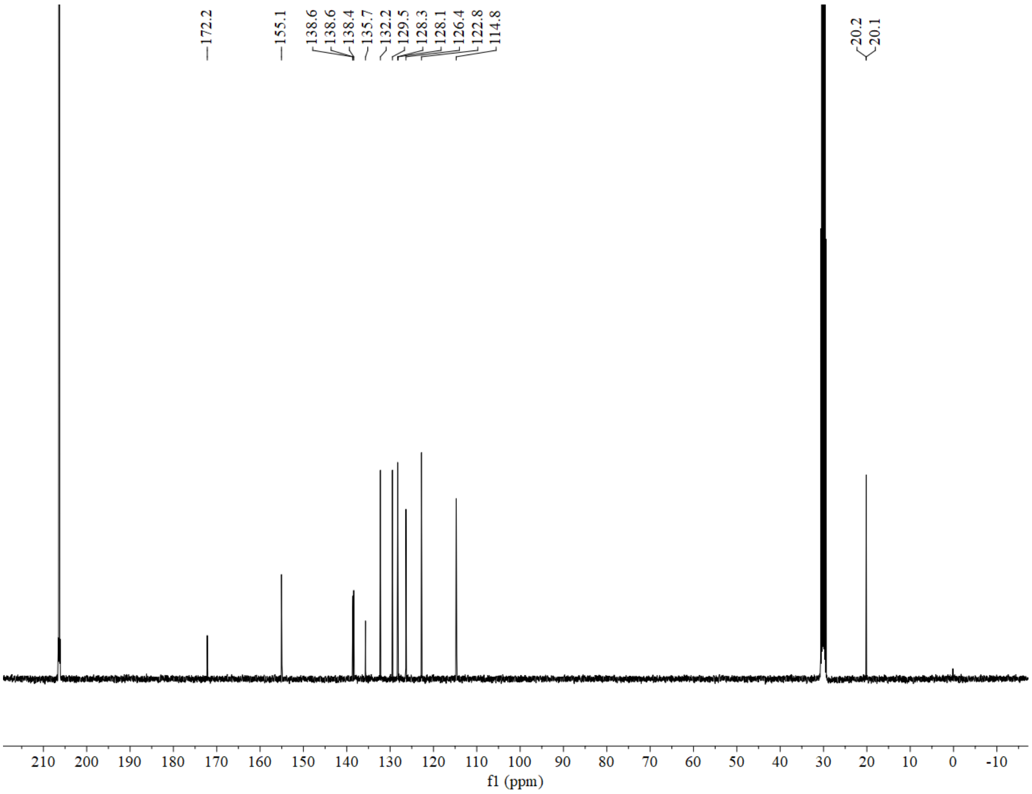

Supplementary Figure 185 HPLC spectra of racemic **9**

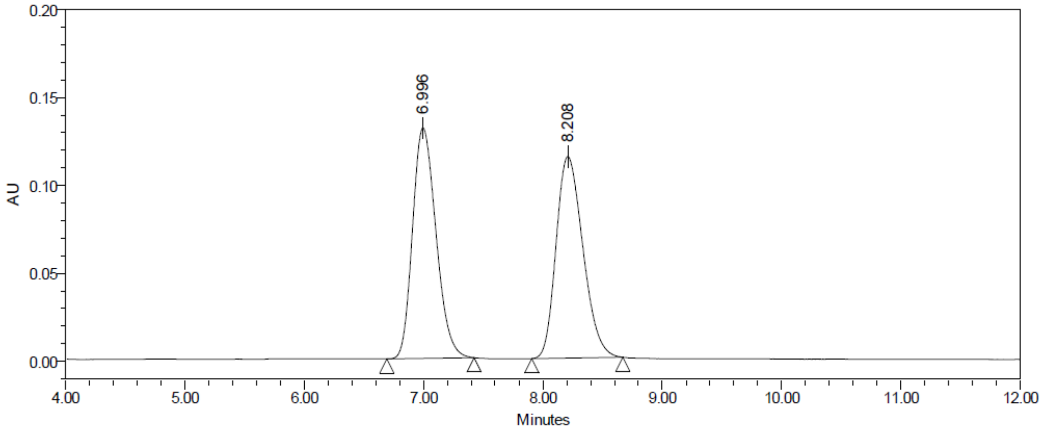

	RT	Area	% Area	Height
1	6.996	1791200	50.01	131212
2	8.208	1790302	49.99	114659

Supplementary Figure 186 HPLC spectra of (*S*)- **9**

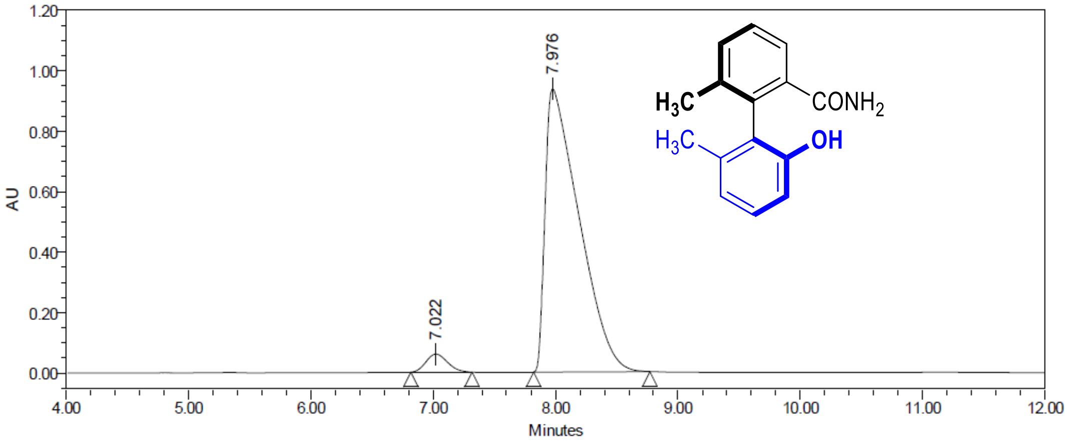

	RT	Area	% Area	Height
1	7.022	730876	3.89	59684
2	7.976	18039112	96.11	935616

Supplementary Figure 187 ^{1}H NMR (400 MHz, $CDCl_3$) of **10**

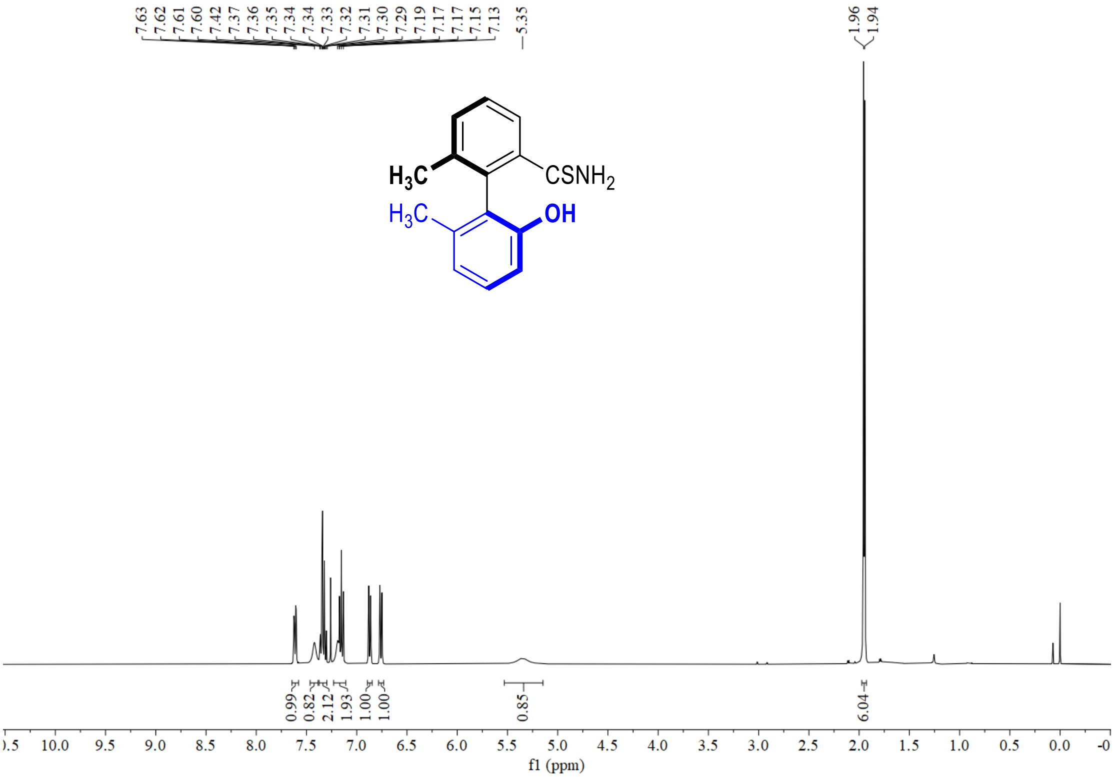

Supplementary Figure 188 ^{13}C NMR (400 MHz, $CDCl_3$) of **10**

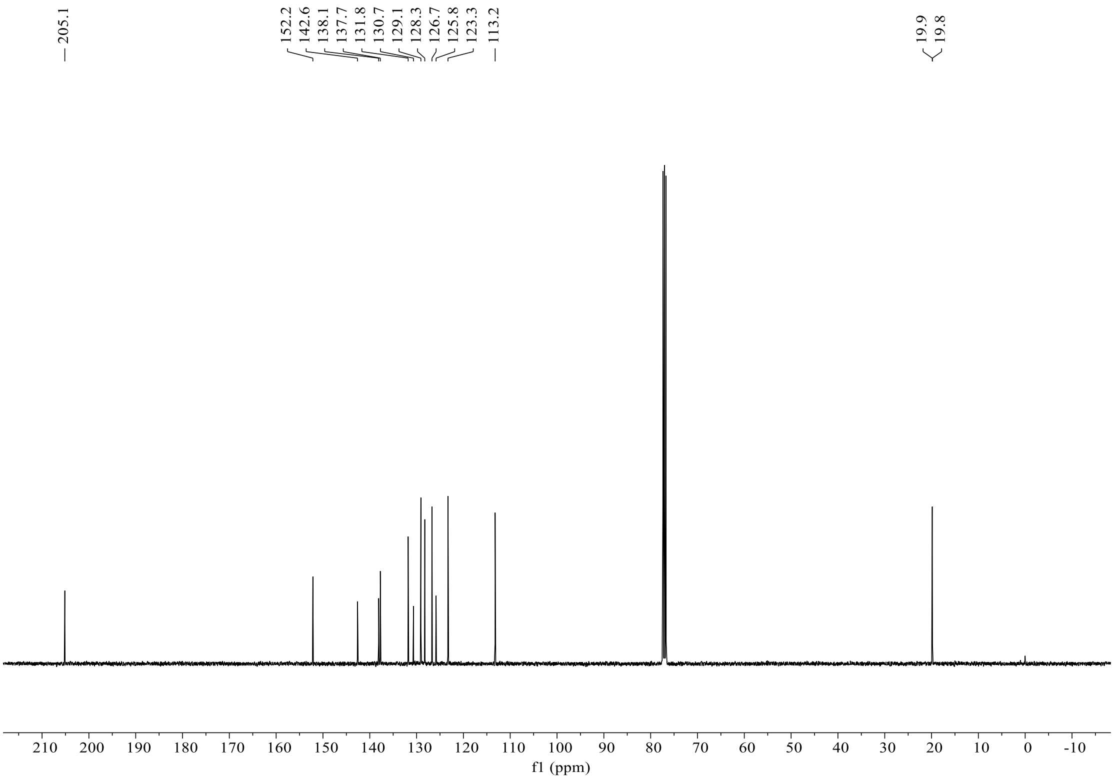

Supplementary Figure 189 HPLC spectra of racemic **10**

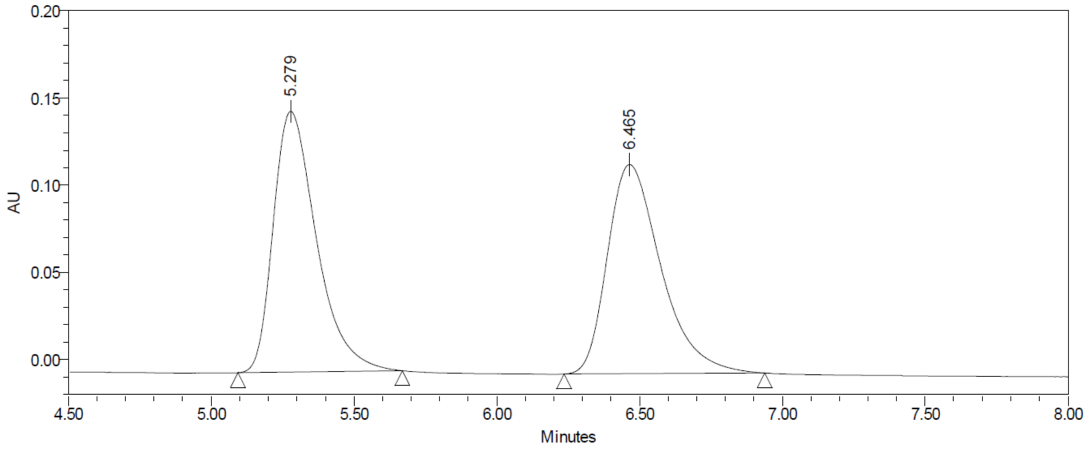

	RT	Area	% Area	Height
1	5.279	1570439	50.13	149381
2	6.465	1562049	49.87	119893

Supplementary Figure 190 HPLC spectra of (*S*)- **10**

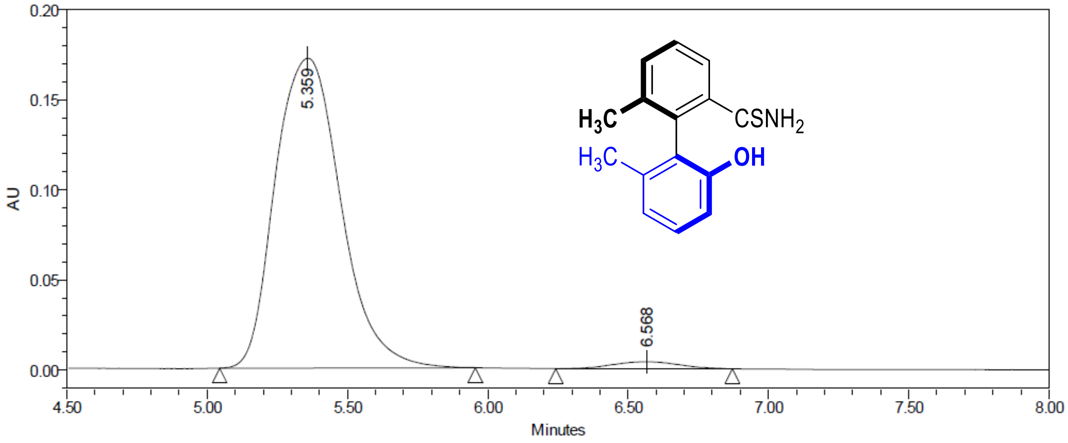

	RT	Area	% Area	Height
1	5.359	2803217	97.83	172148
2	6.568	62249	2.17	3770

Supplementary Figure 191 ^{1}H NMR (400 MHz, $CDCl_3$) of **11**

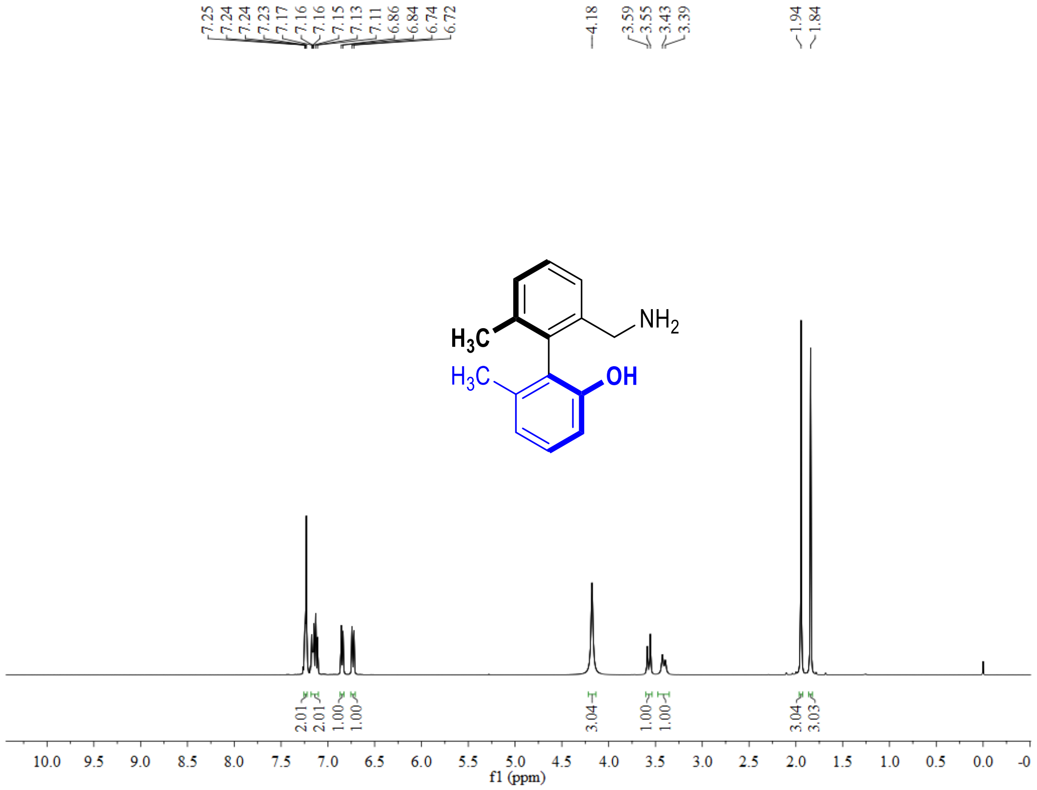

Supplementary Figure 192 ^{13}C NMR (400 MHz, $CDCl_3$) of **11**

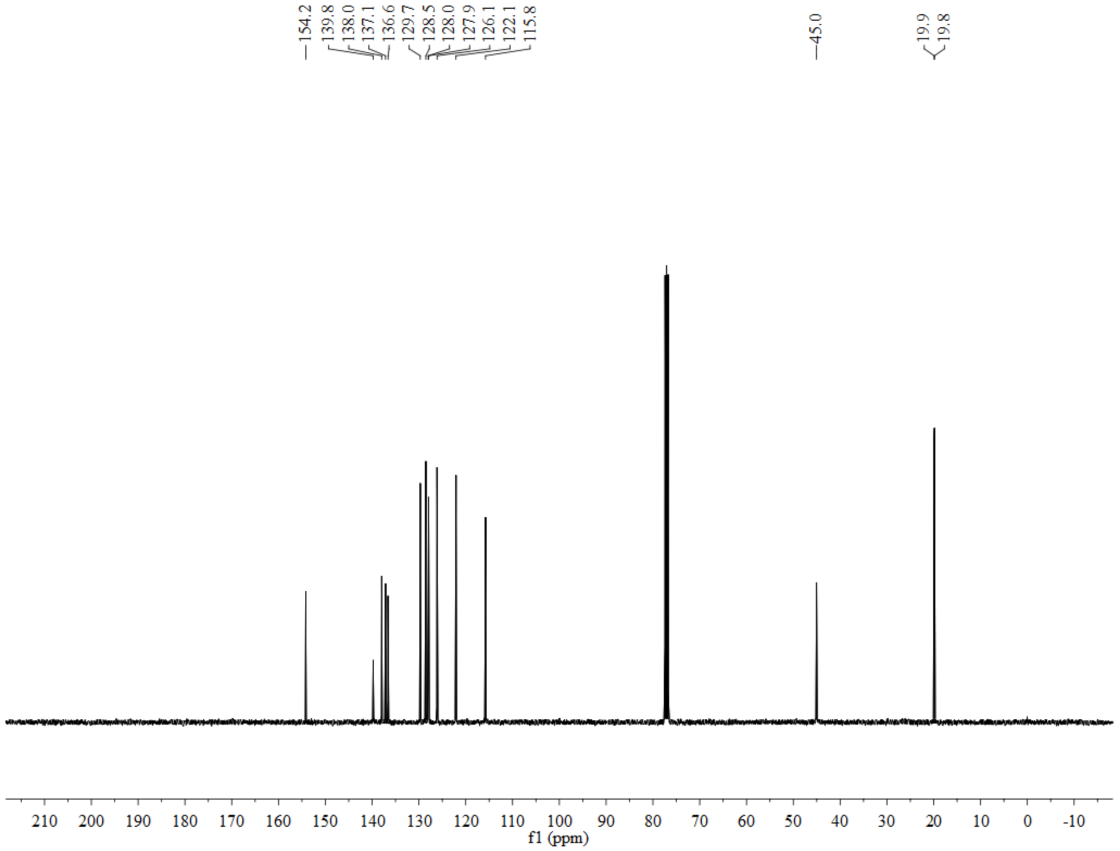

Supplementary Figure 193 ^{1}H NMR (400 MHz, $CDCl_3$) of **12**

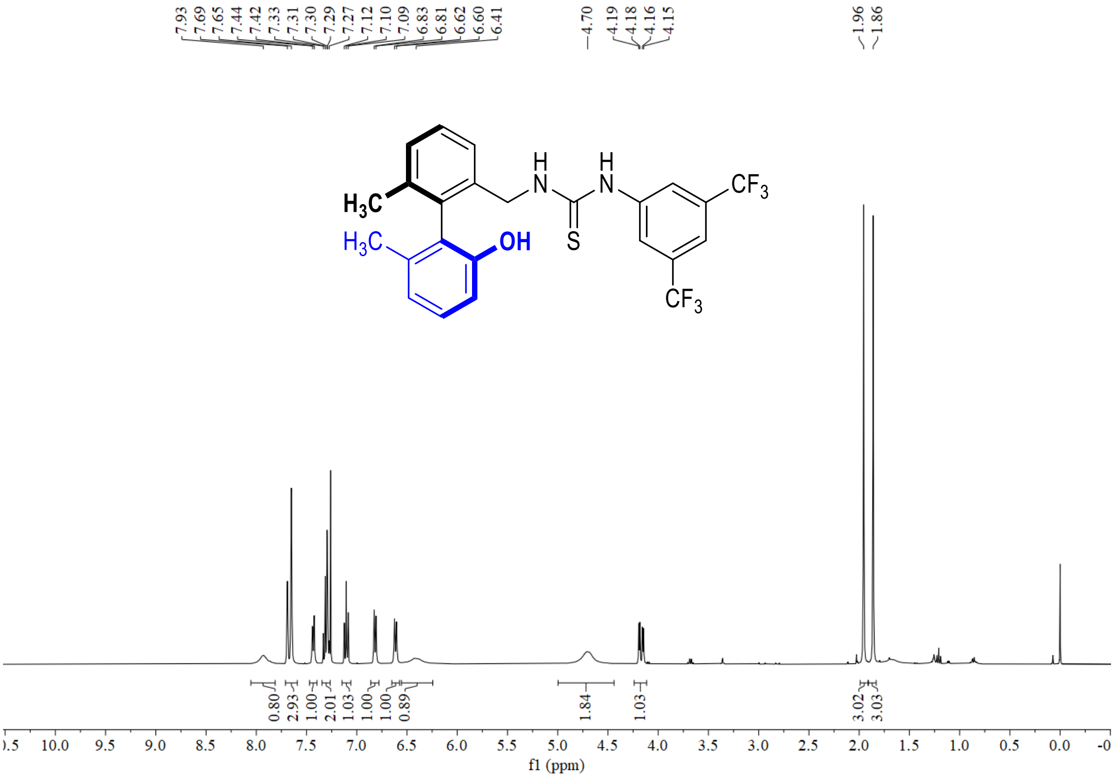

Supplementary Figure 194 ^{13}C NMR (400 MHz, $CDCl_3$) of **12**

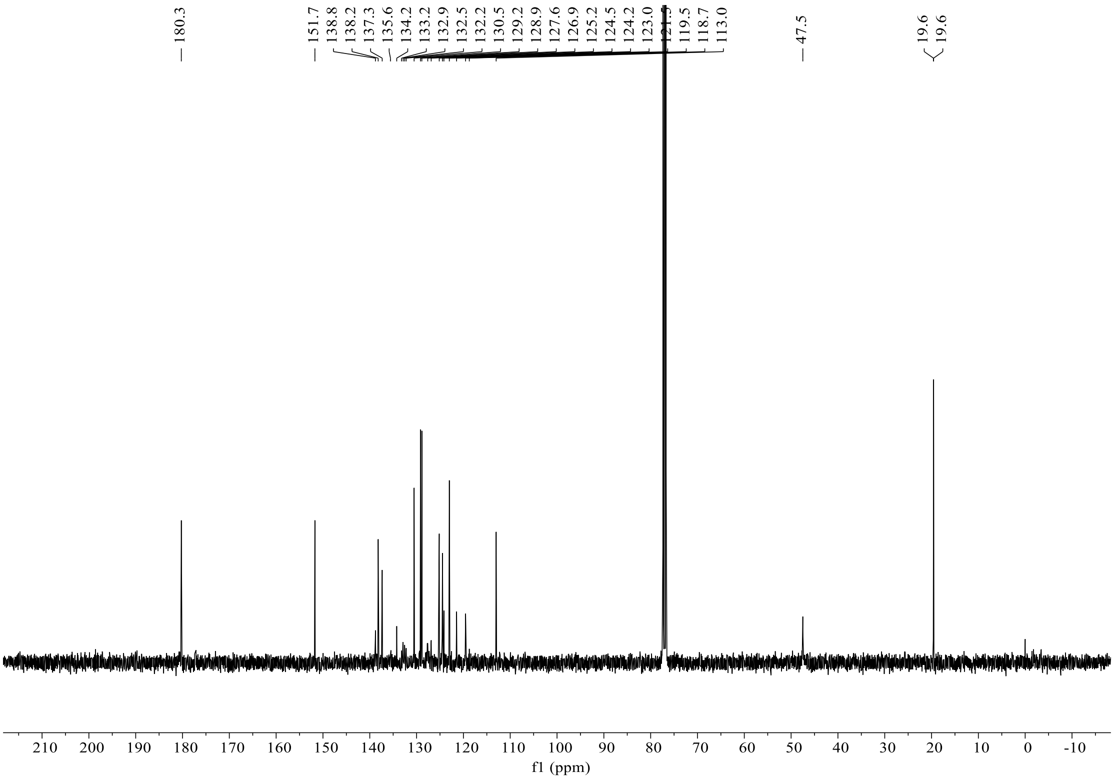

Supplementary Figure 195 ^{19}F NMR (400 MHz, $CDCl_3$) of **12**

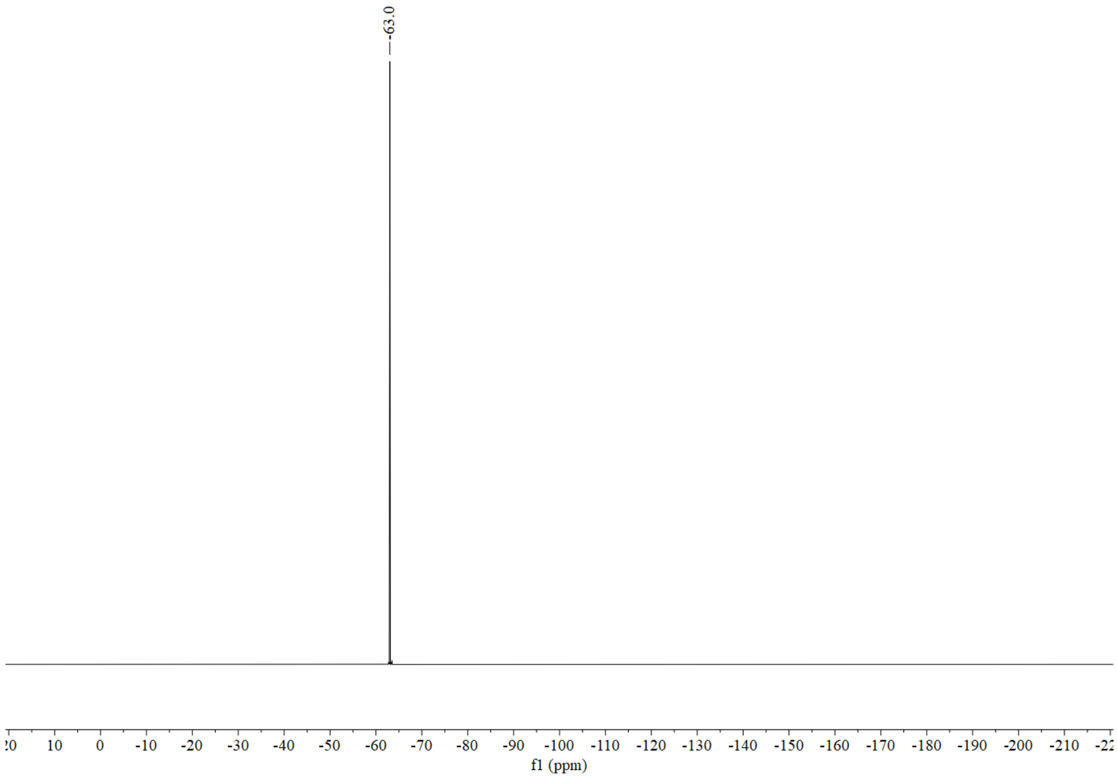

Supplementary Figure 196 HPLC spectra of racemic **12**

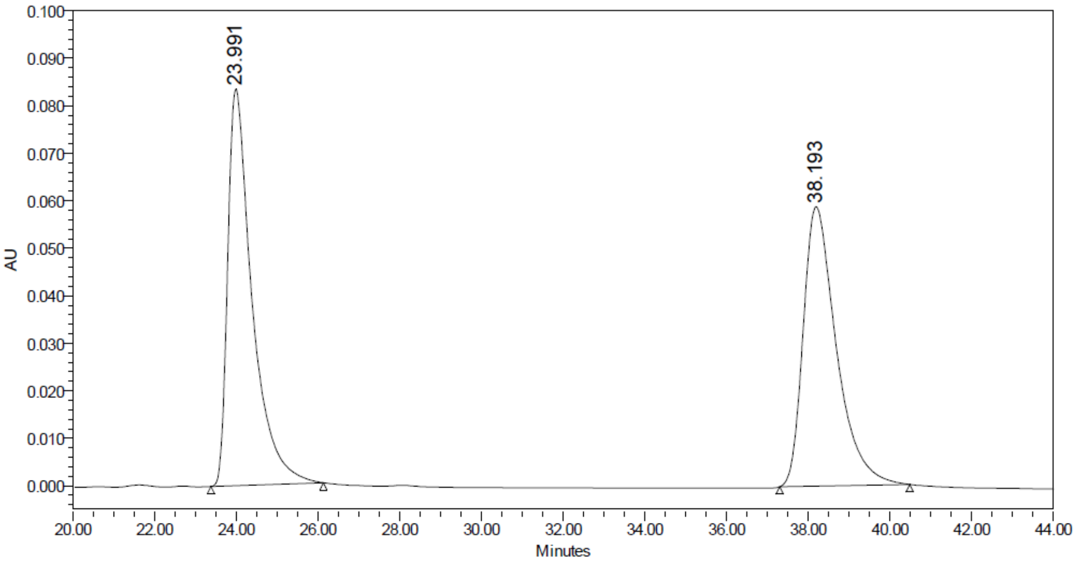

	RT	Area	% Area	Height
1	23.991	3455734	51.08	83473
2	38.193	3309241	48.92	58814

Supplementary Figure 197 HPLC spectra of (*S*)- **12**

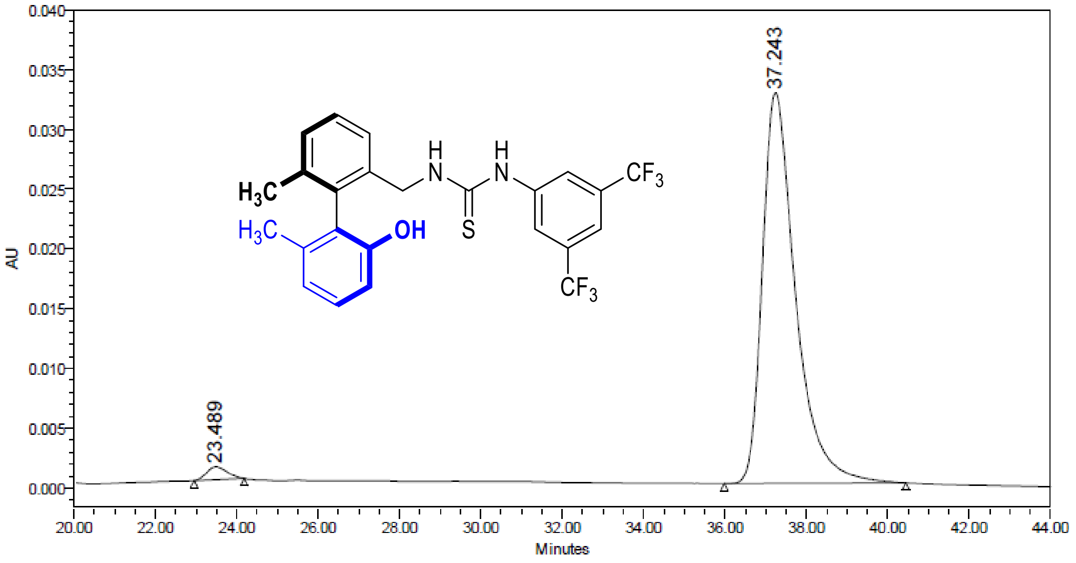

	RT	Area	% Area	Height
1	23.489	37242	1.98	1061
2	37.243	1845287	98.02	32661

Supplementary Figure 198 ^{1}H NMR (400 MHz, $CDCl_3$) of **13**

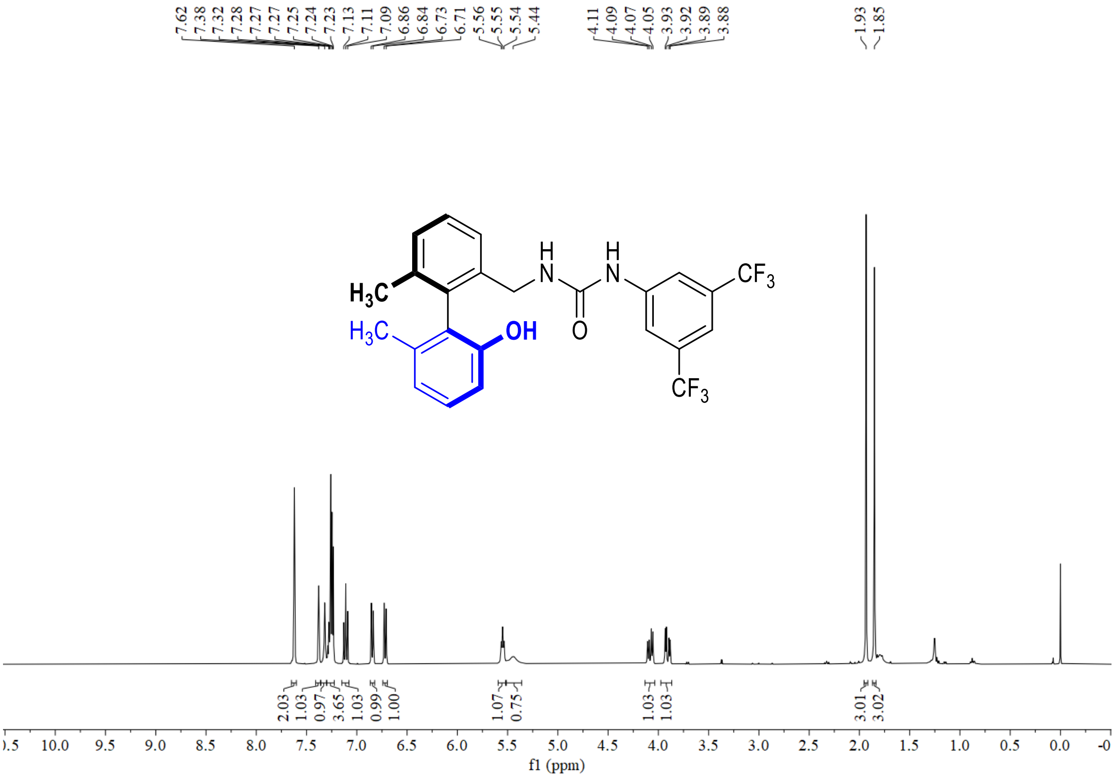

Supplementary Figure 199 ^{13}C NMR (400 MHz, $CDCl_3$) of **13**

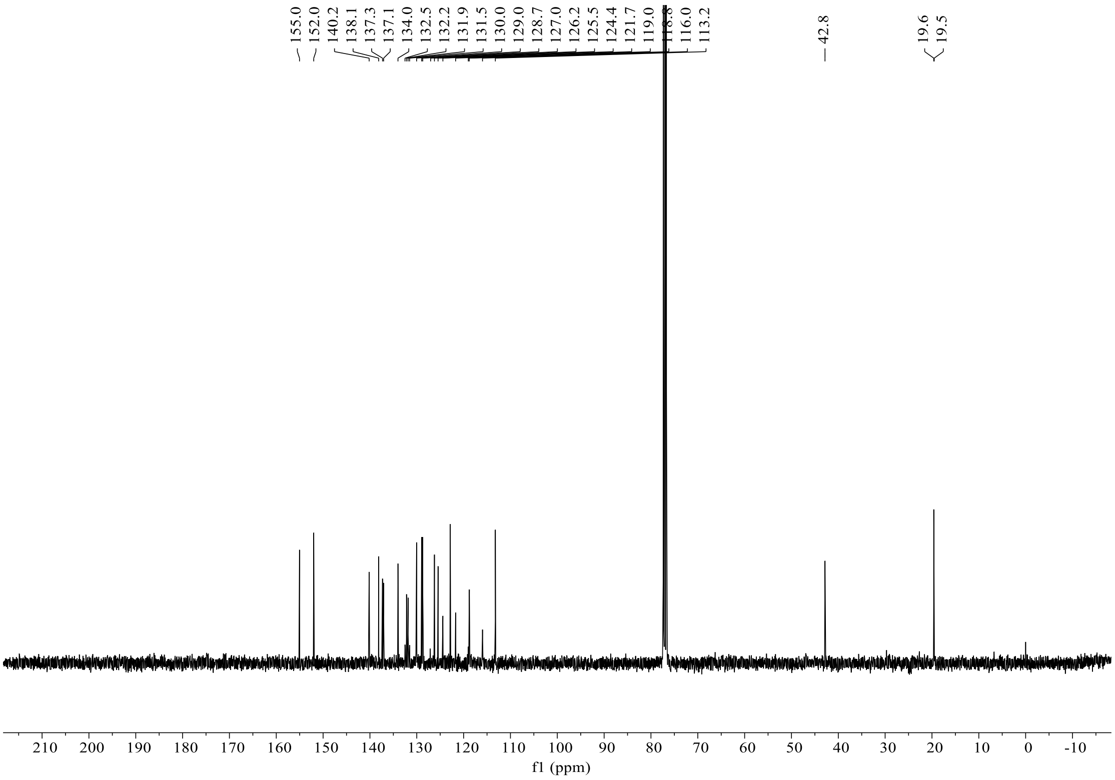

Supplementary Figure 200 ^{19}F NMR (400 MHz, $CDCl_3$) of **13**

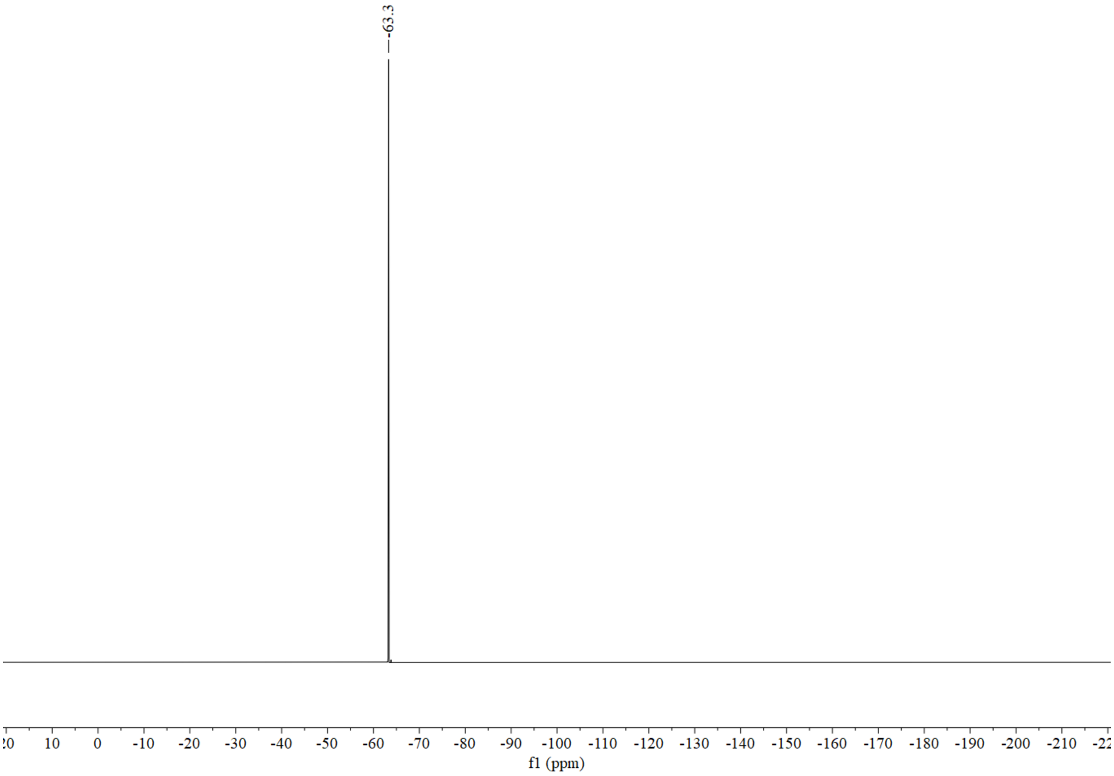

Supplementary Figure 201 HPLC spectra of racemic **13**

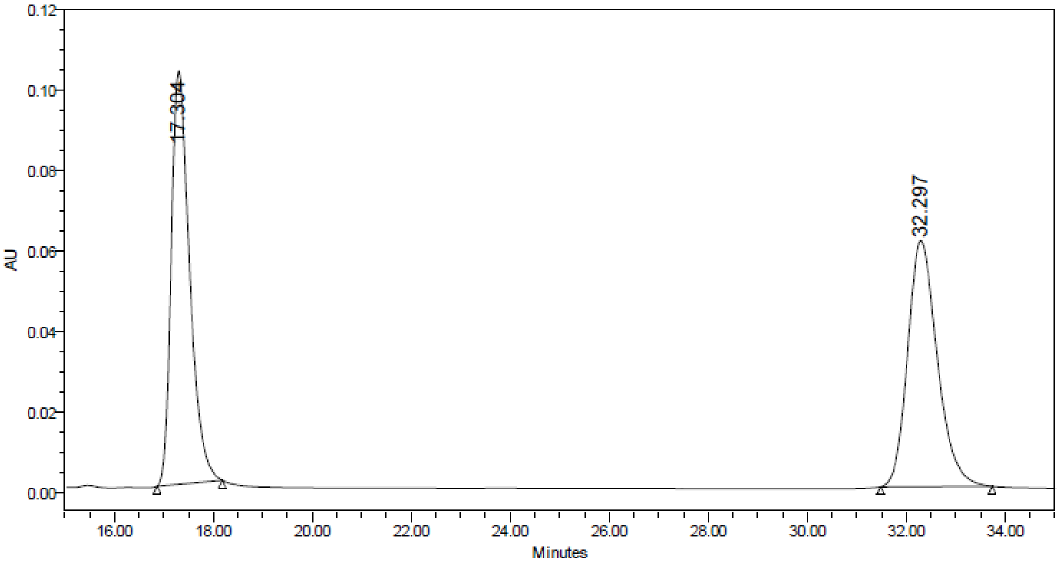

	RT	Area	% Area	Height
1	17.304	2635762	50.97	102503
2	32.297	2535348	49.03	60986

Supplementary Figure 202 HPLC spectra of (*S*)- **13**

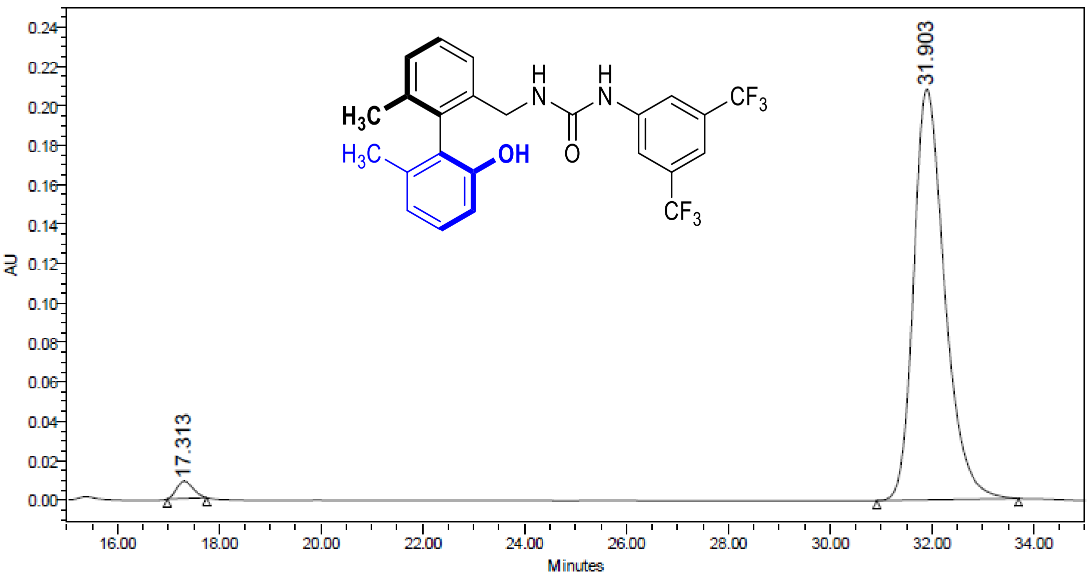

	RT	Area	% Area	Height
1	17.313	195899	2.17	8804
2	31.903	8851527	97.83	208600

Supplementary Figure 203 ^{1}H NMR (400 MHz, $CDCl_3$) of **14**

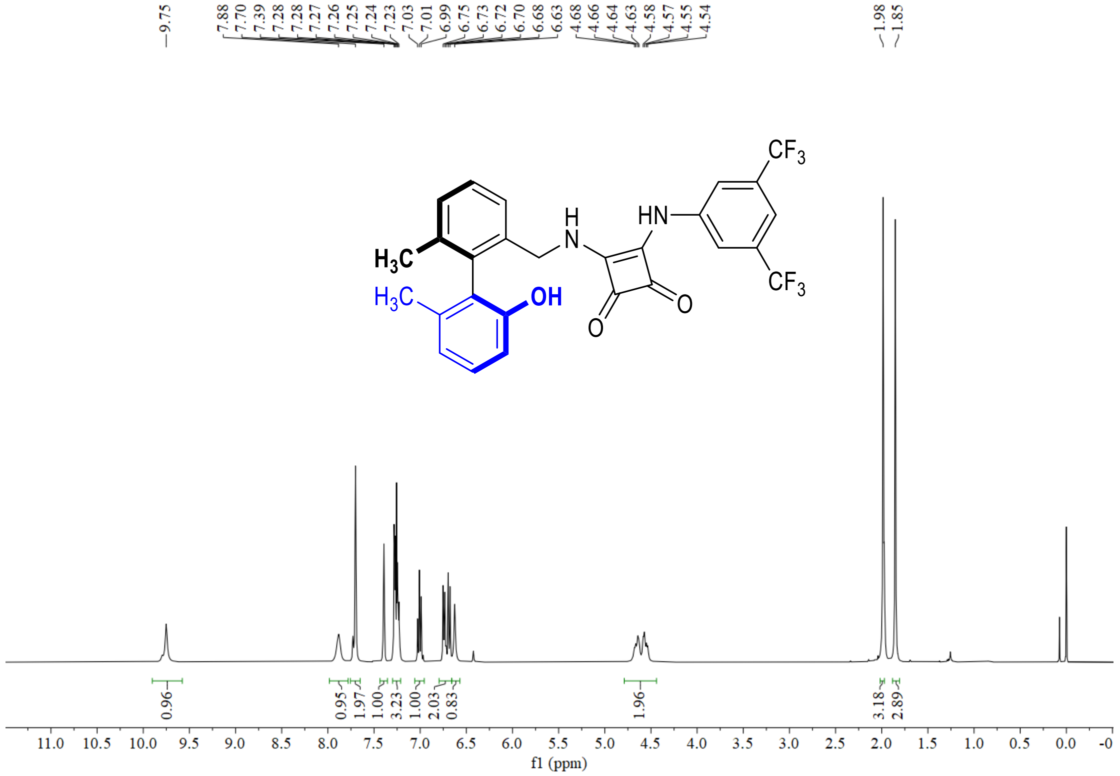

Supplementary Figure 204 ^{13}C NMR (400 MHz, $CDCl_3$) of **14**

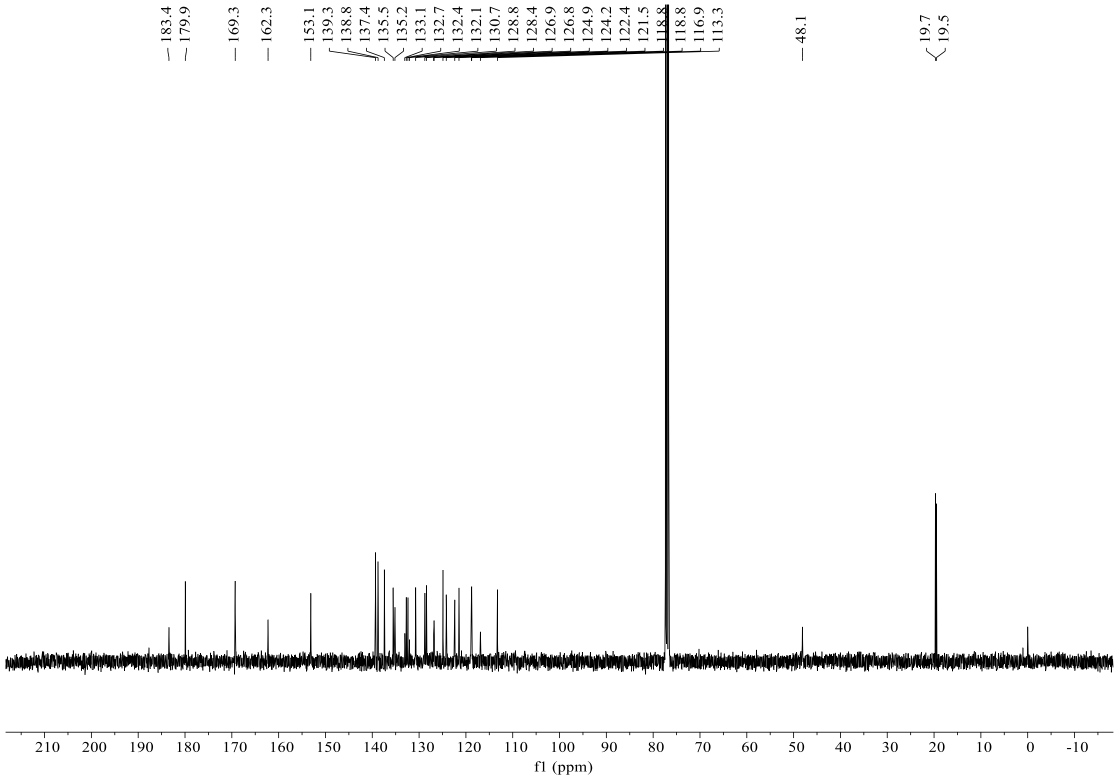

Supplementary Figure 205 ^{19}F NMR (400 MHz, $CDCl_3$) of **14**

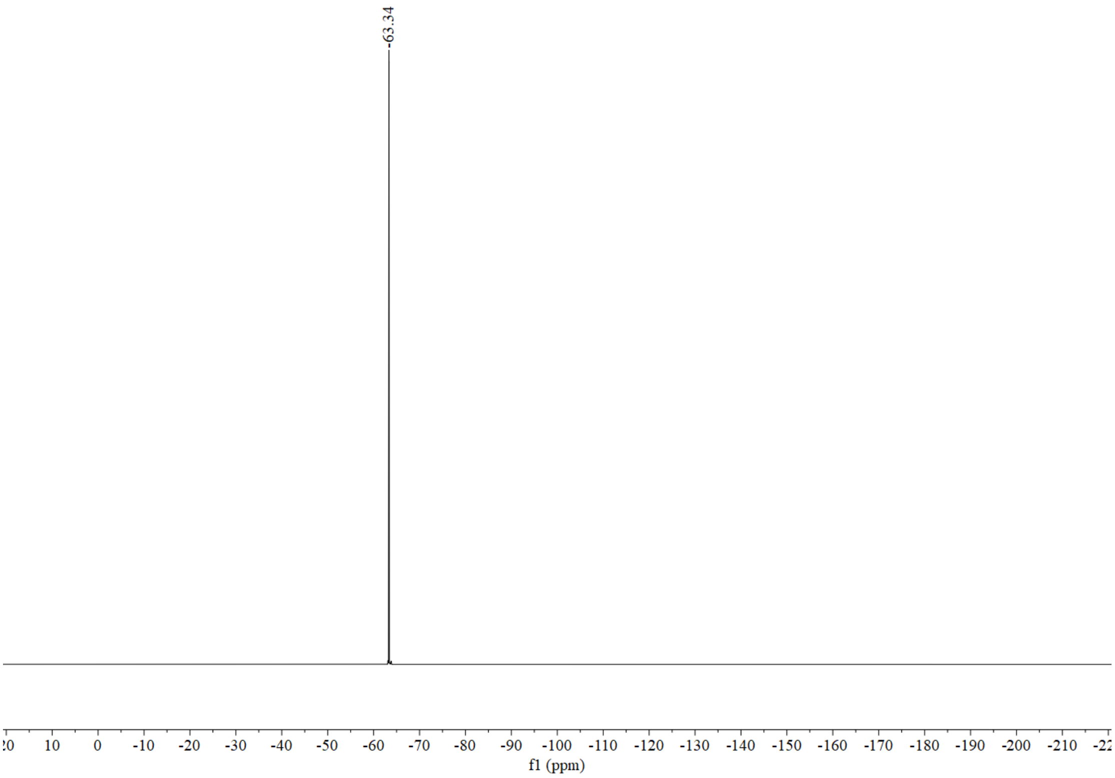

Supplementary Figure 206 HPLC spectra of racemic **14**

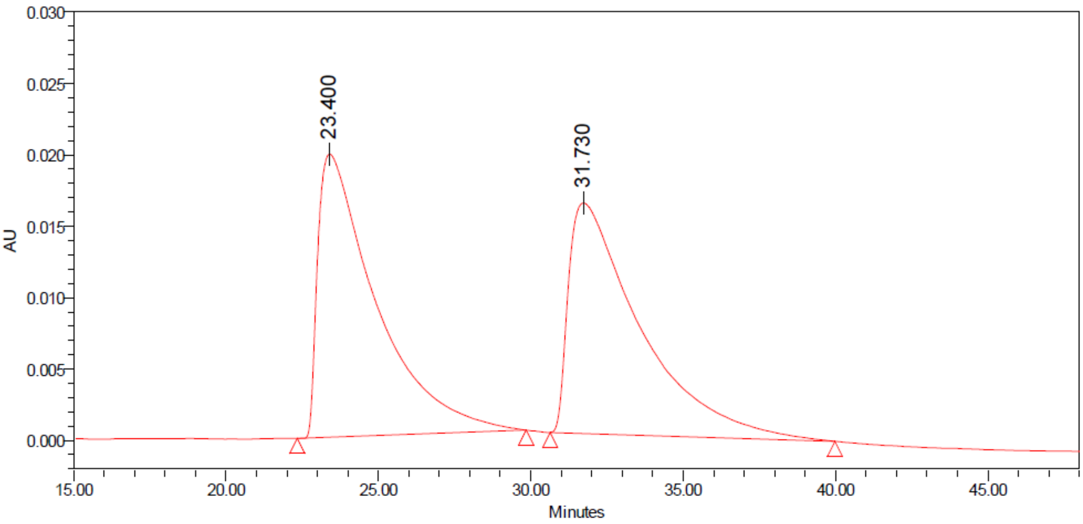

	RT	Area	% Area	Height
1	23.400	2638417	50.01	19815
2	31.730	2636888	49.99	16138

Supplementary Figure 207 HPLC spectra of (*S*)- **14**

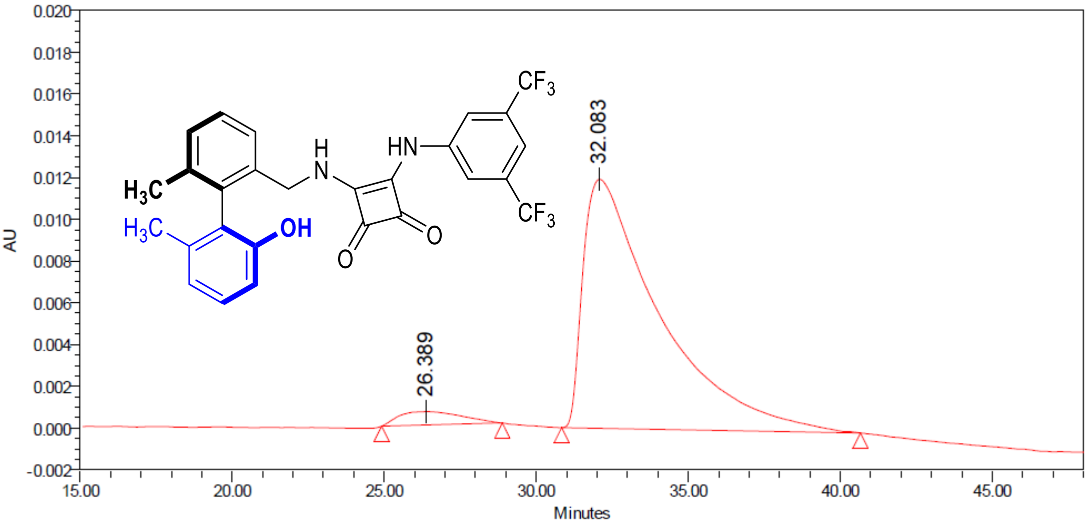

	RT	Area	% Area	Height
1	26.389	91949	4.23	642
2	32.083	2081488	95.77	11929

Supplementary Figure 208 ^{1}H NMR (400 MHz, $CDCl_3$) of **15**

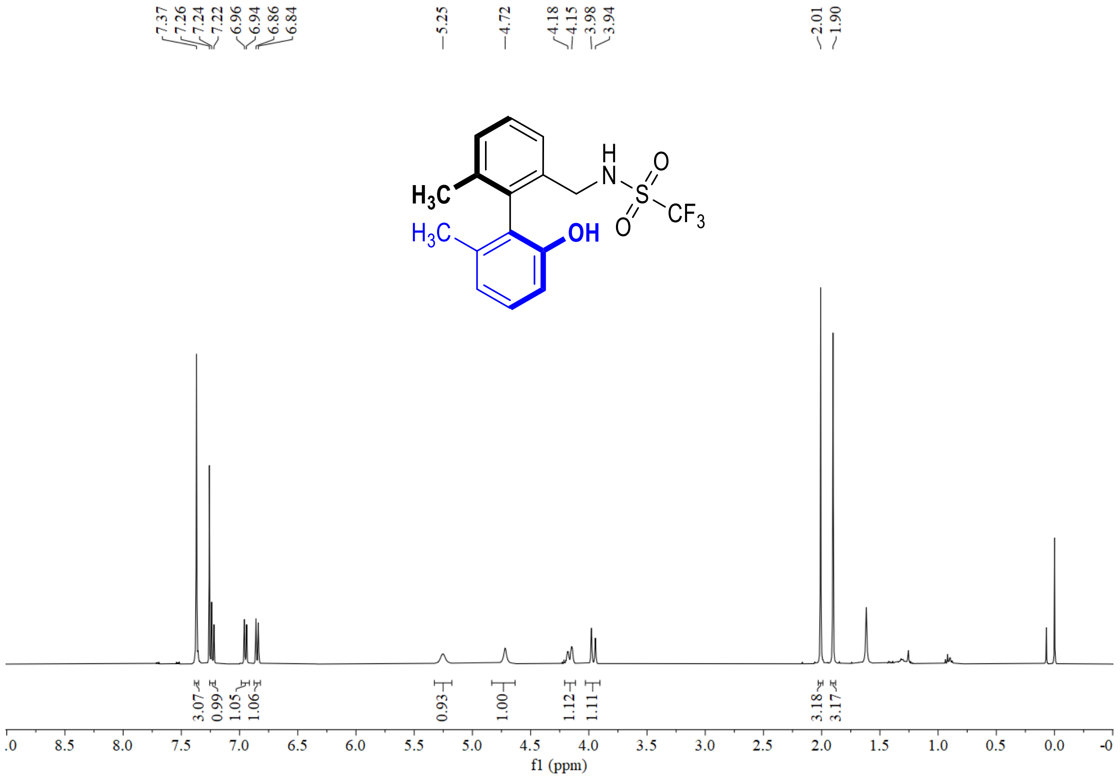

Supplementary Figure 209 ^{13}C NMR (400 MHz, $CDCl_3$) of **15**

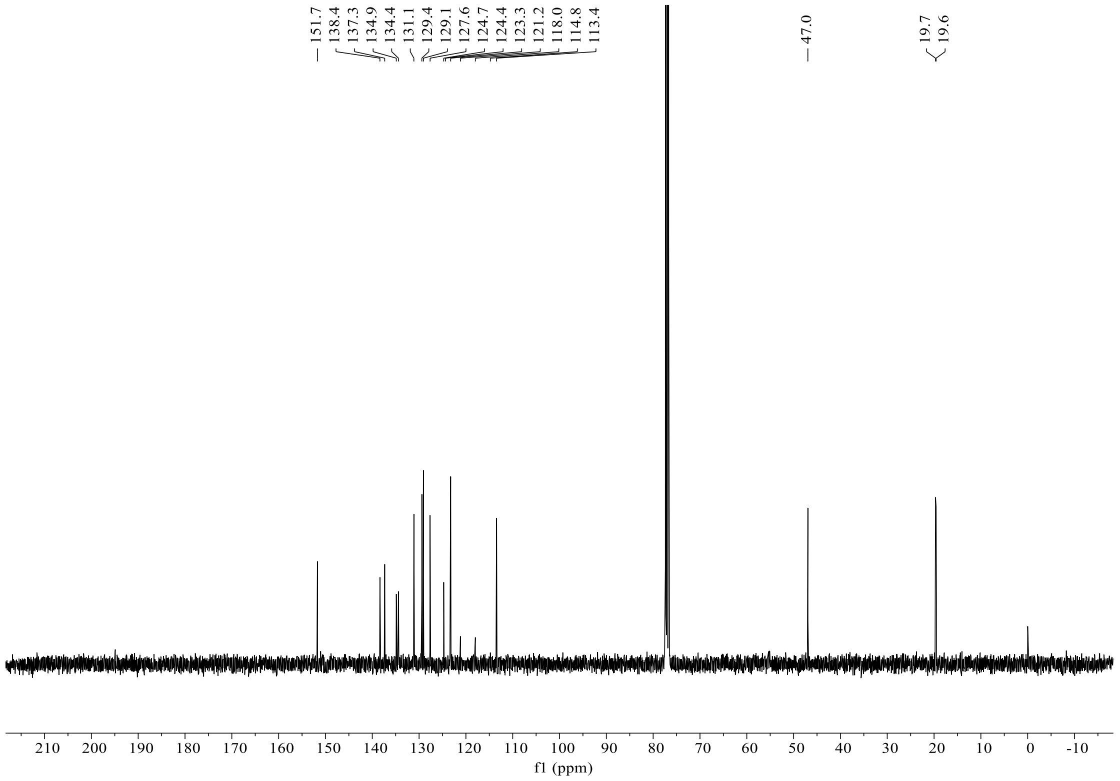

Supplementary Figure 210 ^{19}F NMR (400 MHz, $CDCl_3$) of **15**

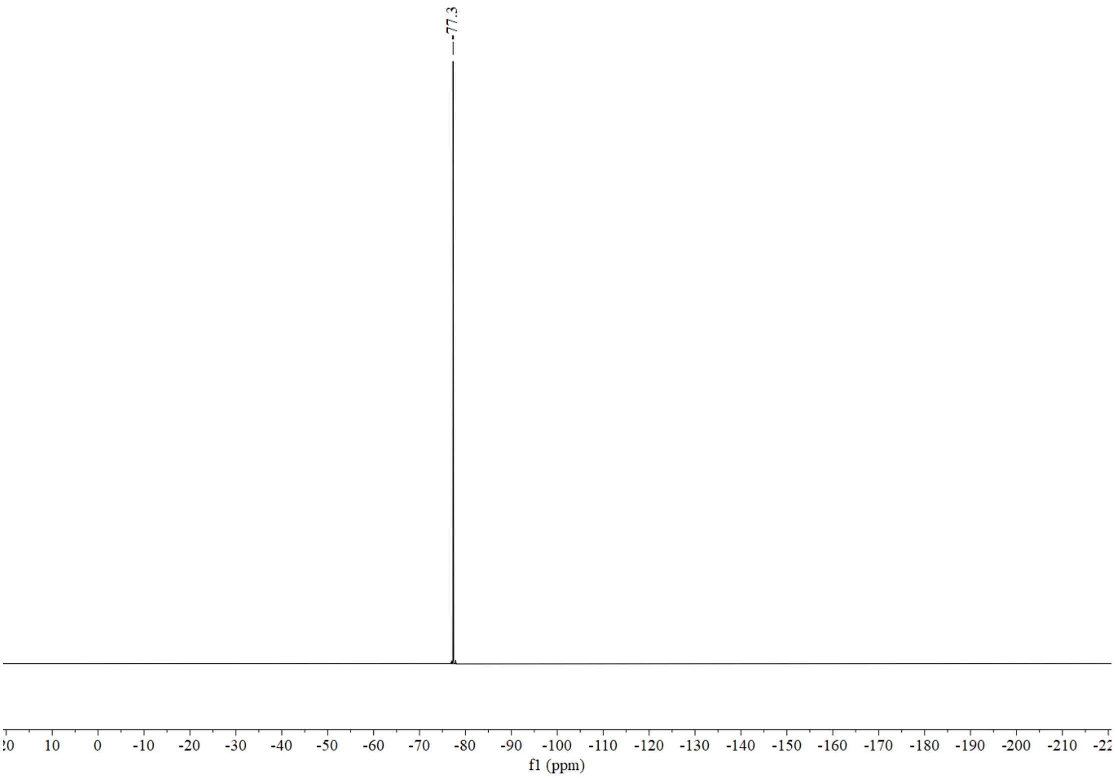

Supplementary Figure 211 HPLC spectra of racemic **15**

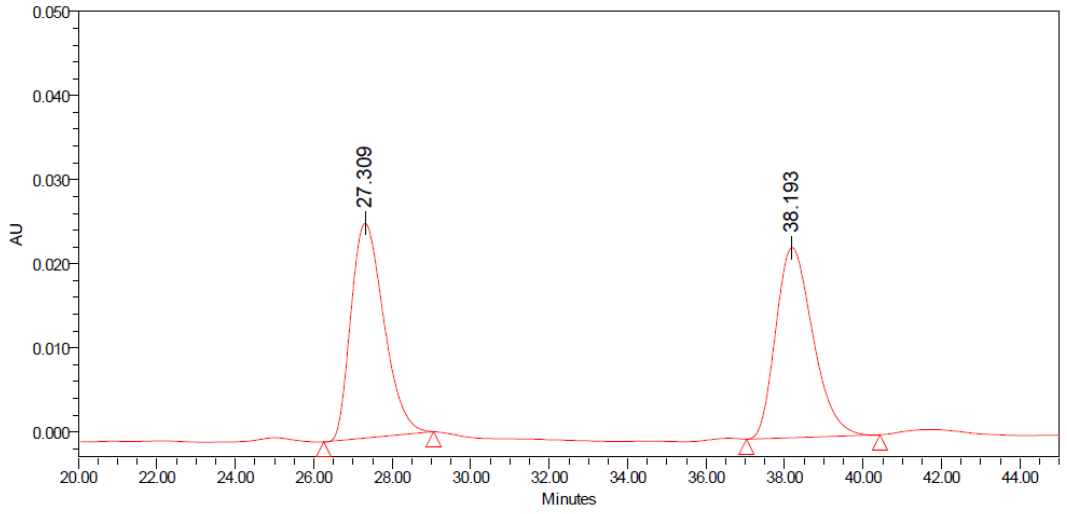

	RT	Area	% Area	Height
1	27.309	1491842	50.08	25561
2	38.193	1486833	49.92	22615

Supplementary Figure 212 HPLC spectra of (*S*)- **15**

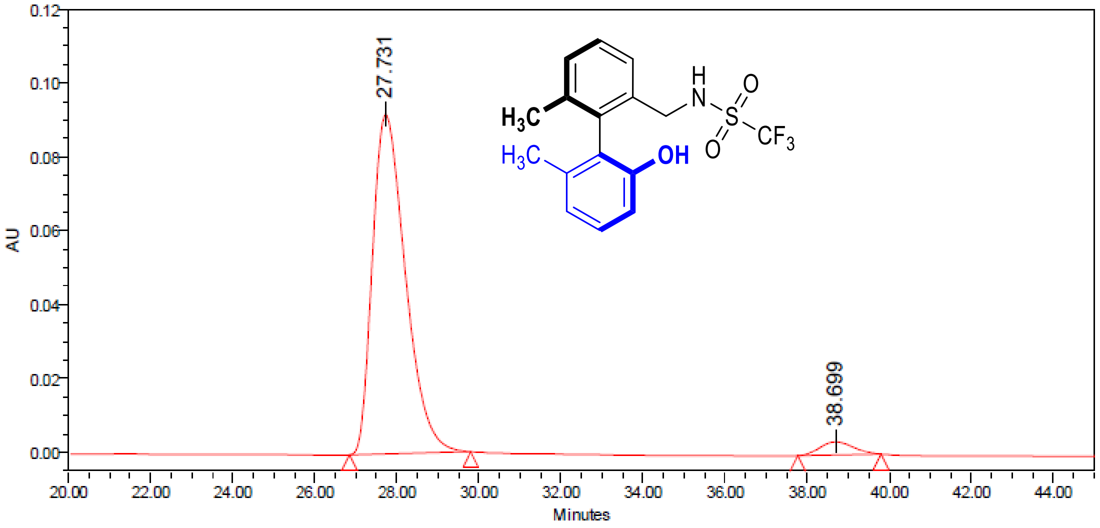

	RT	Area	% Area	Height
1	27.731	5171057	96.08	92012
2	38.699	211046	3.92	3674

Supplementary Figure 213 ^{1}H NMR (400 MHz, $CDCl_3$) of **16**

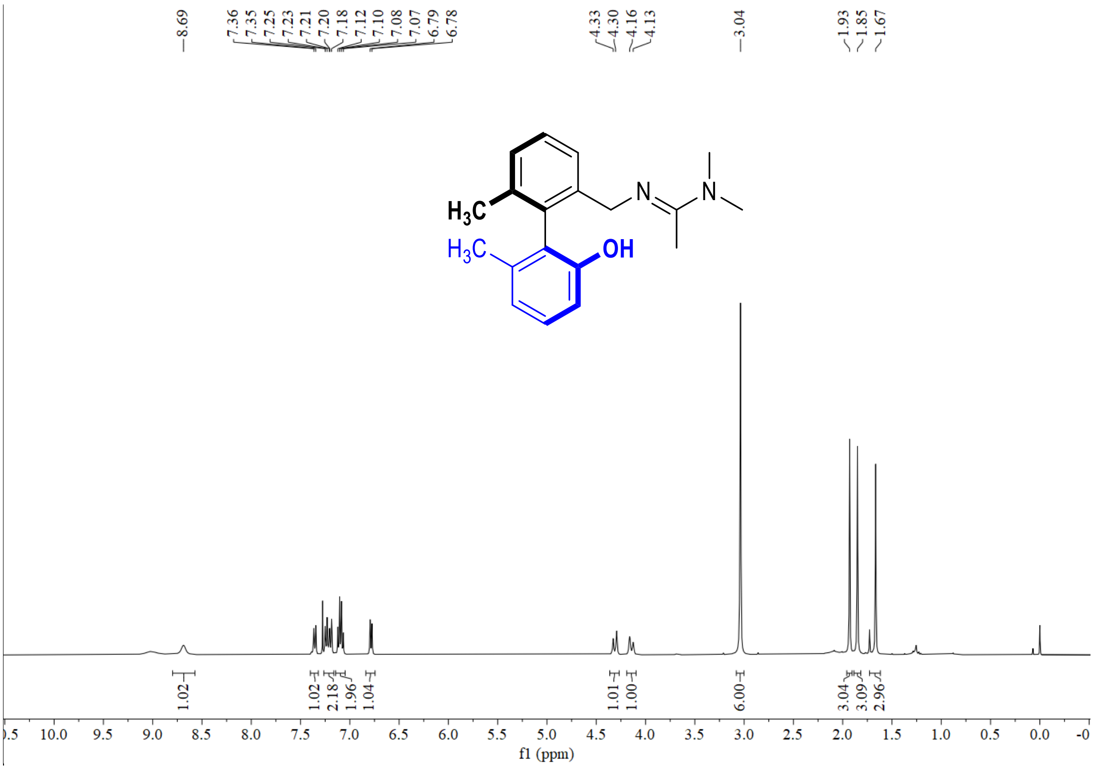

Supplementary Figure 214 ^{13}C NMR (400 MHz, $CDCl_3$) of **16**

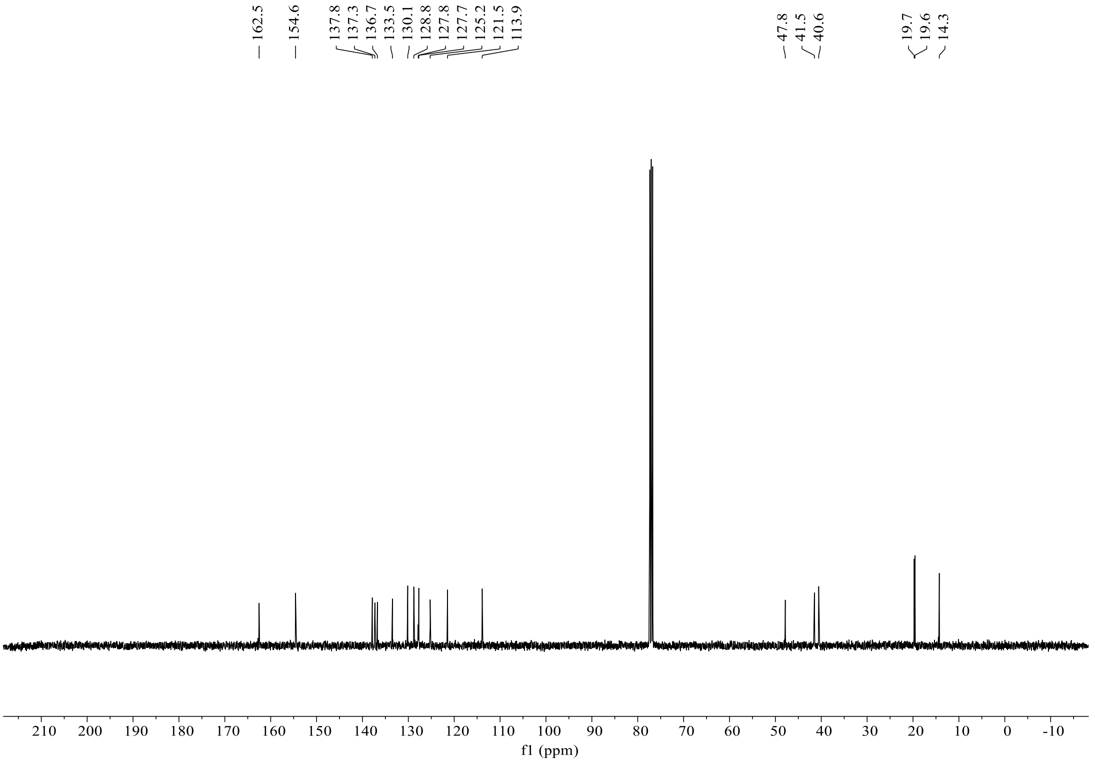

Supplementary Figure 215 ^{1}H NMR (400 MHz, $CDCl_3$) of **17**

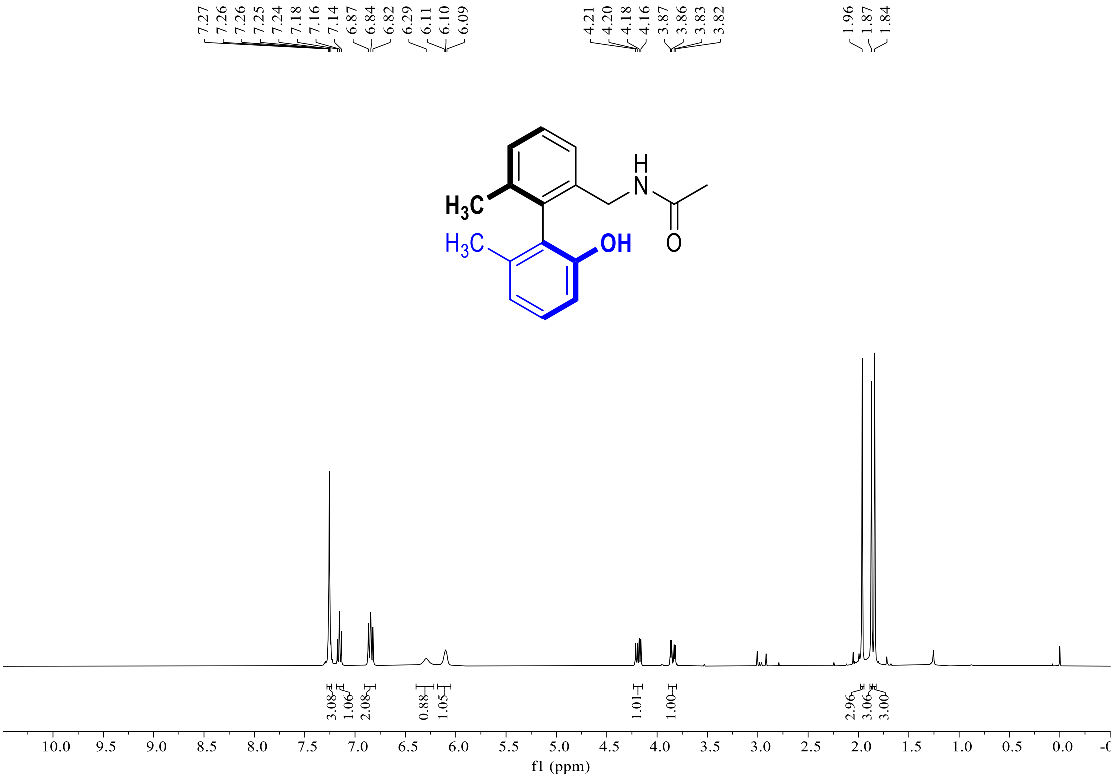

Supplementary Figure 216 ^{13}C NMR (400 MHz, $CDCl_3$) of **17**

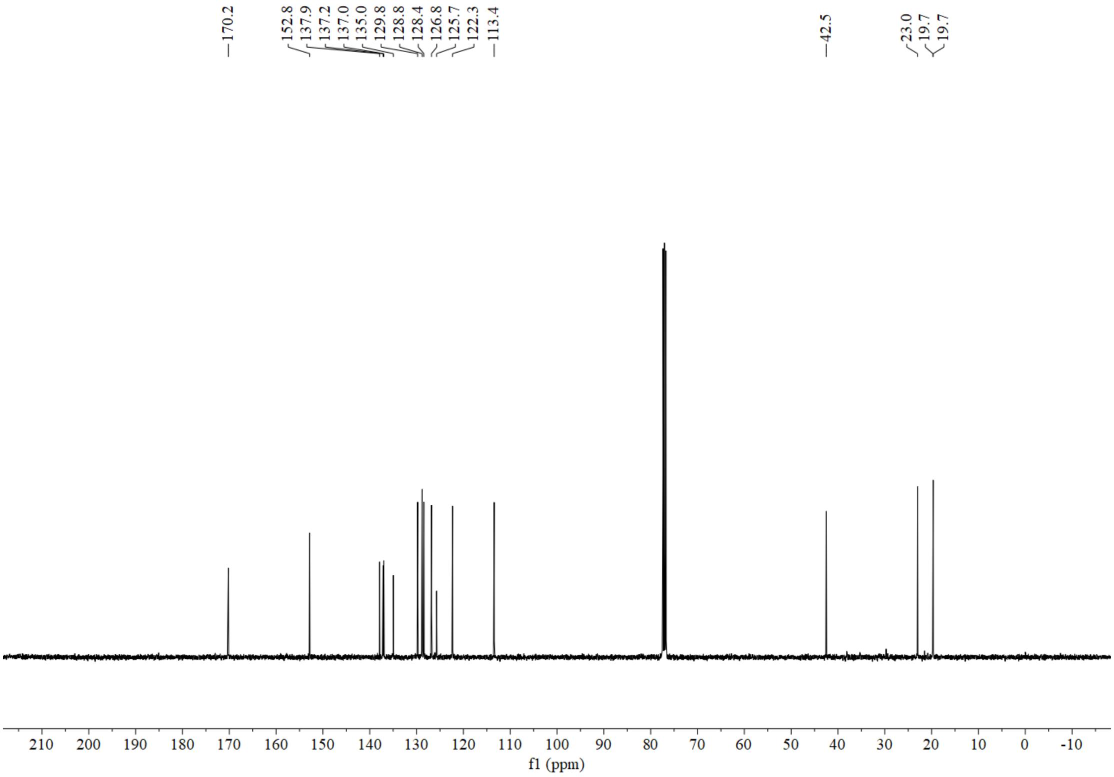

Supplementary Figure 217 HPLC spectra of racemic **17**

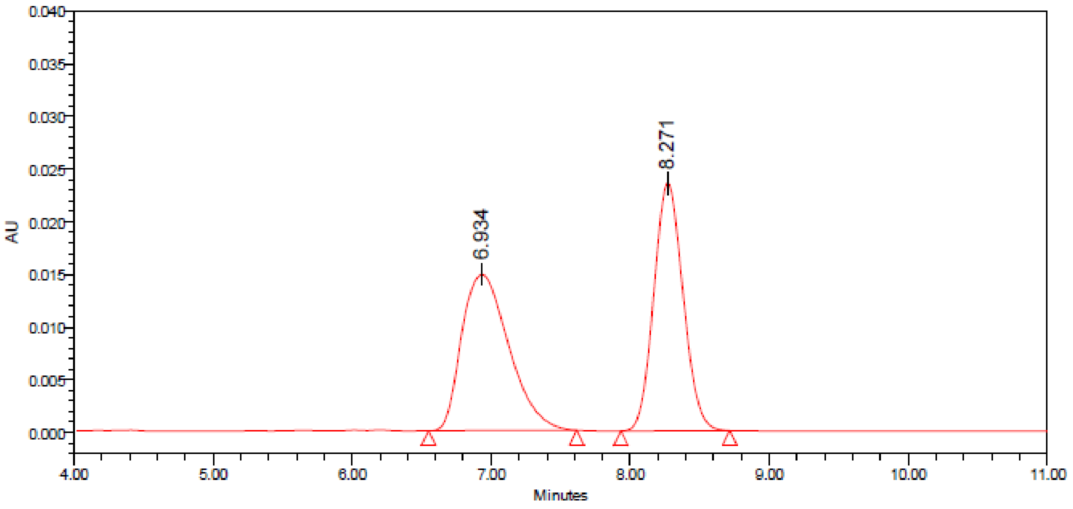

	RT	Area	% Area	Height
1	6.934	349683	50.40	14836
2	8.271	344190	49.60	23531

Supplementary Figure 218 HPLC spectra of (*S*)- **17**

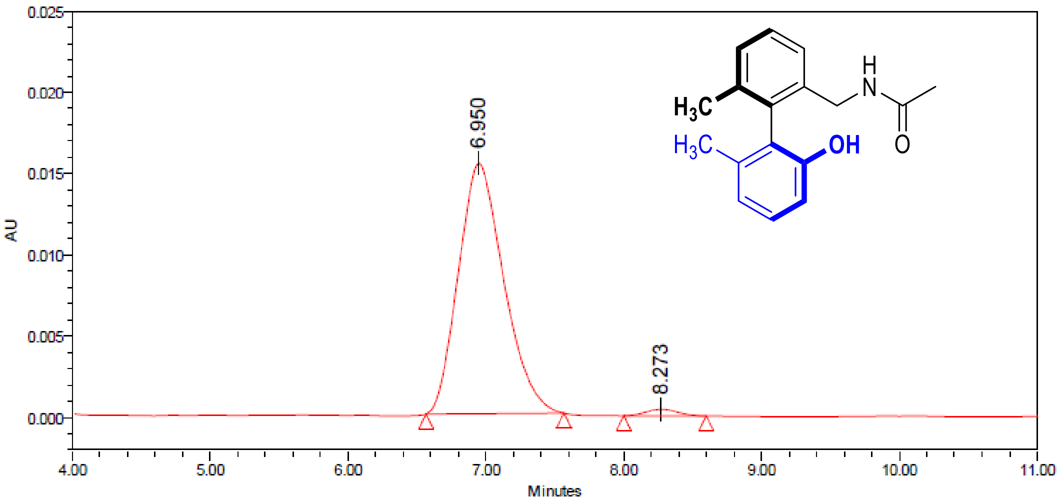

	RT	Area	% Area	Height
1	6.950	352893	98.23	15436
2	8.273	6349	1.77	409

Supplementary Figure 219 ^{1}H NMR (400 MHz, $CDCl_3$) of **18**

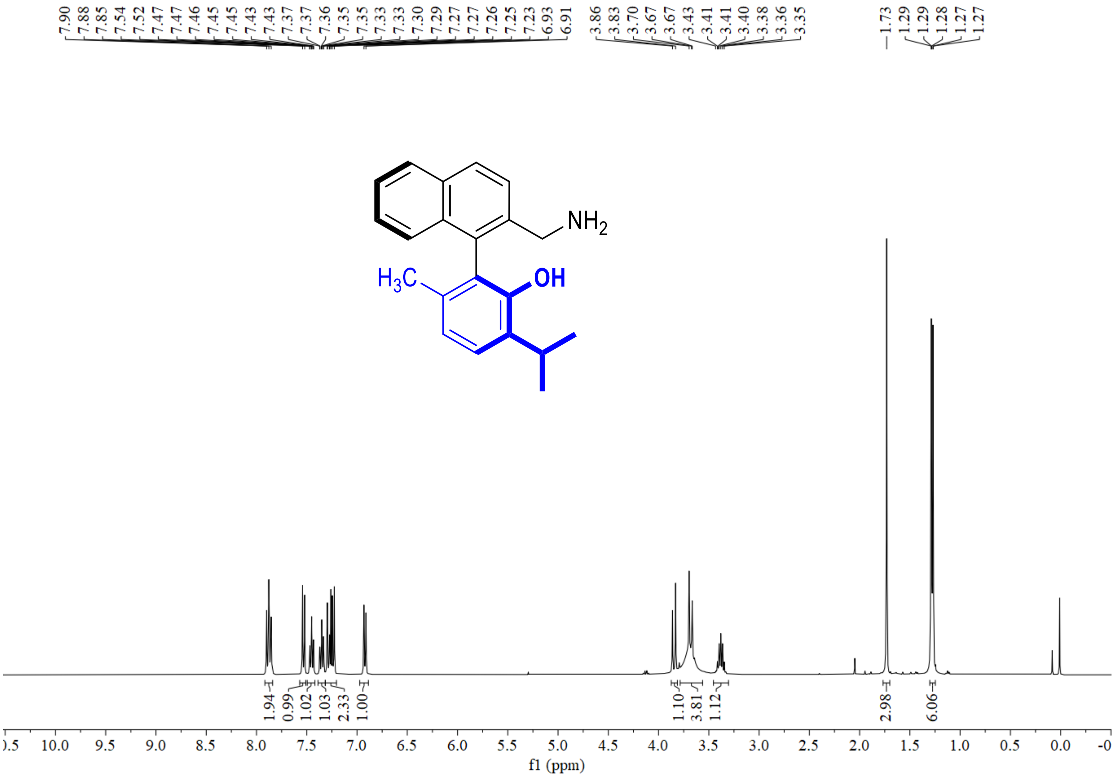

Supplementary Figure 220 ^{13}C NMR (400 MHz, $CDCl_3$) of **18**

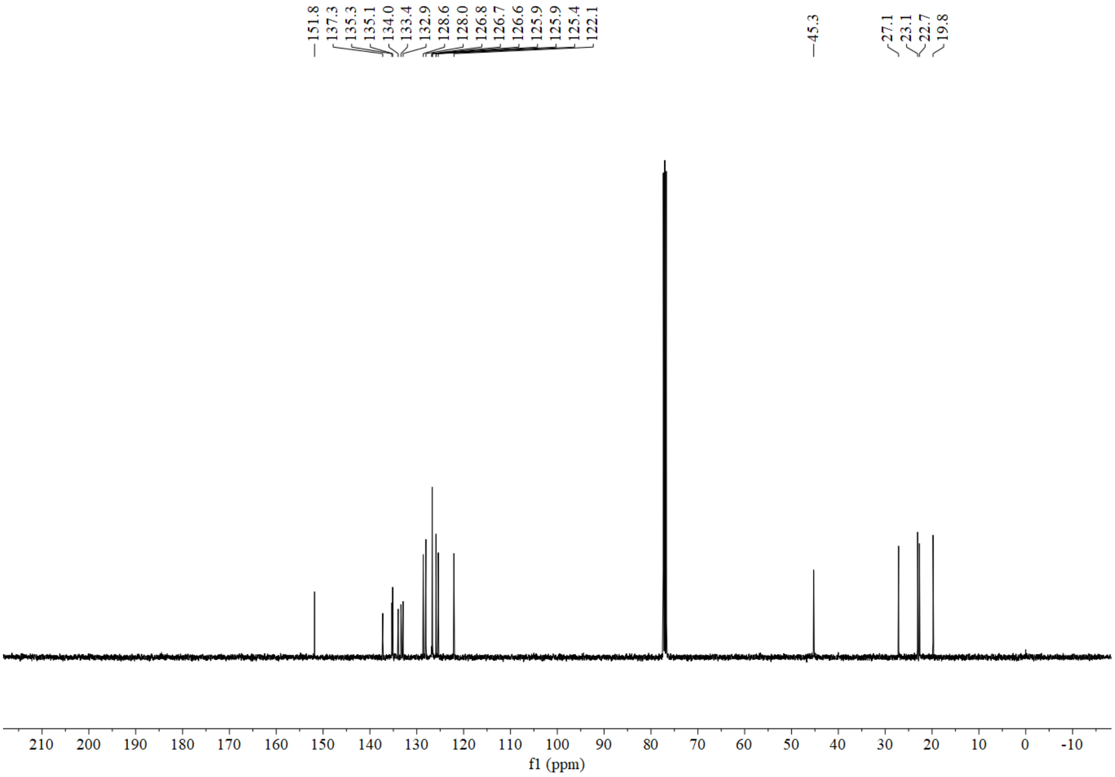

Supplementary Figure 221 ^{1}H NMR (400 MHz, $CDCl_3$) of **19**

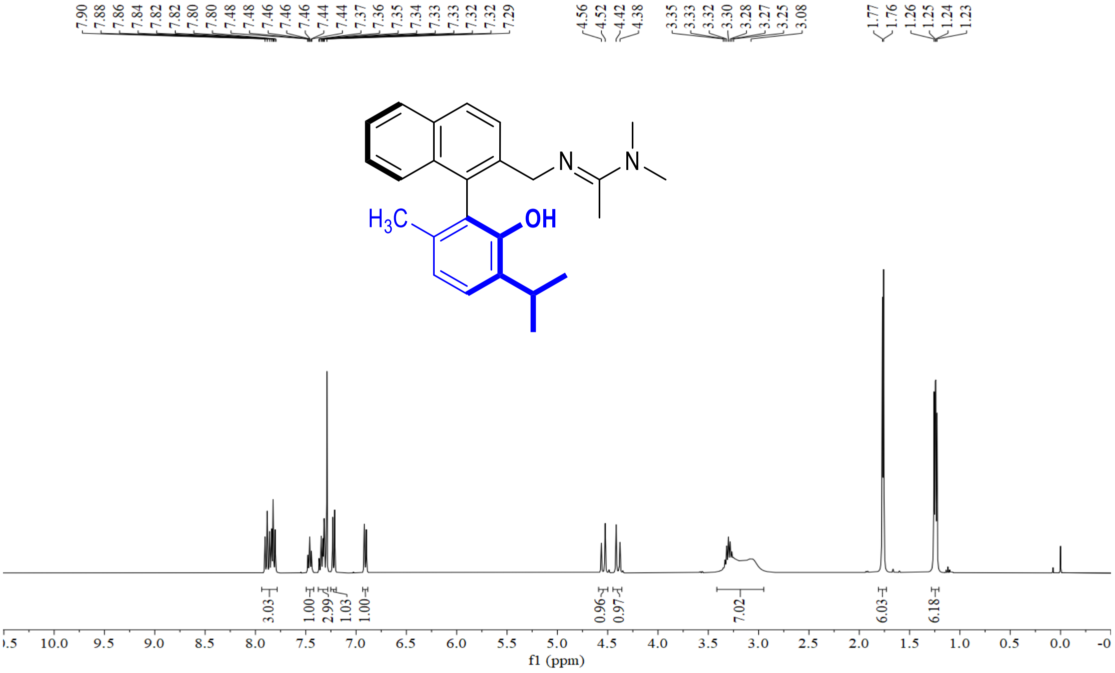

Supplementary Figure 222 ^{13}C NMR (400 MHz, $CDCl_3$) of **19**

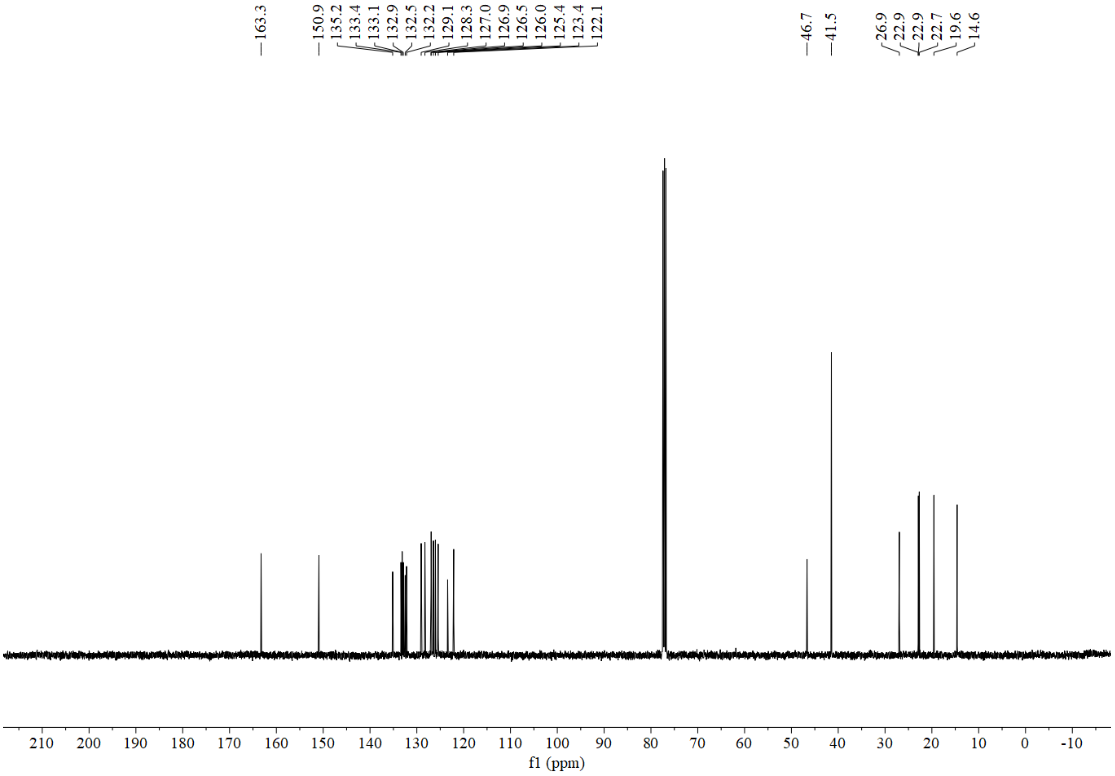

Supplementary Figure 223 ^{1}H NMR (400 MHz, $CDCl_3$) of **20**

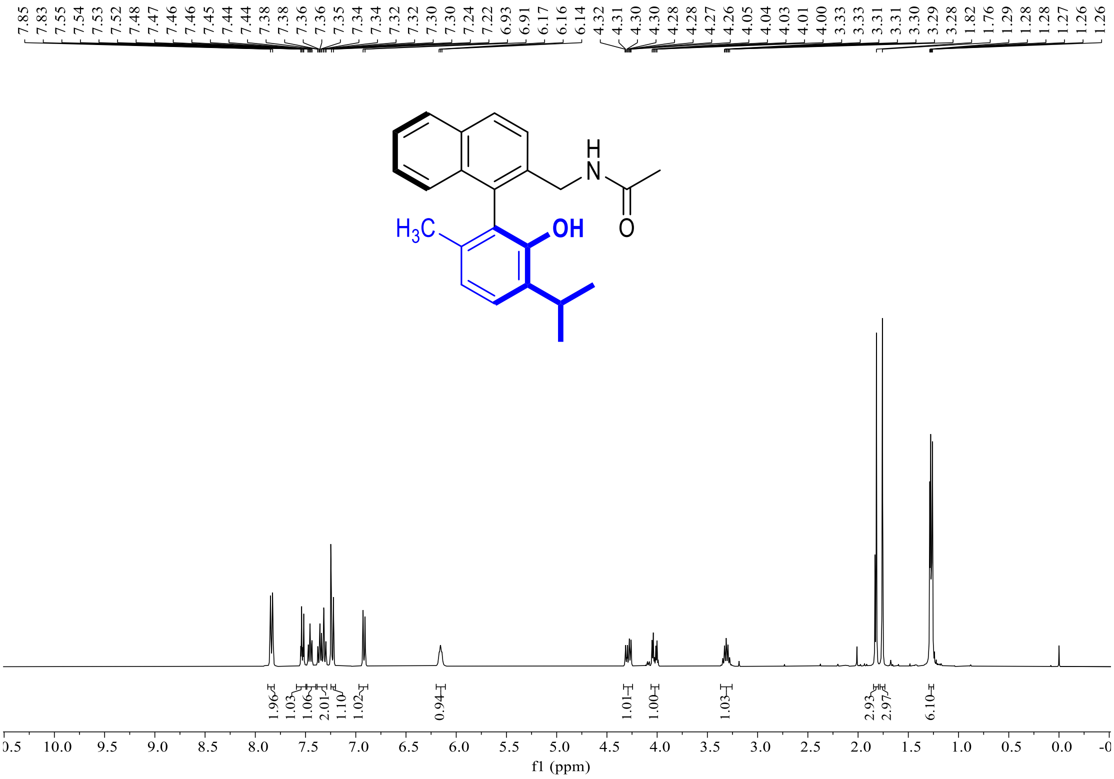

Supplementary Figure 224 ^{13}C NMR (400 MHz, $CDCl_3$) of **20**

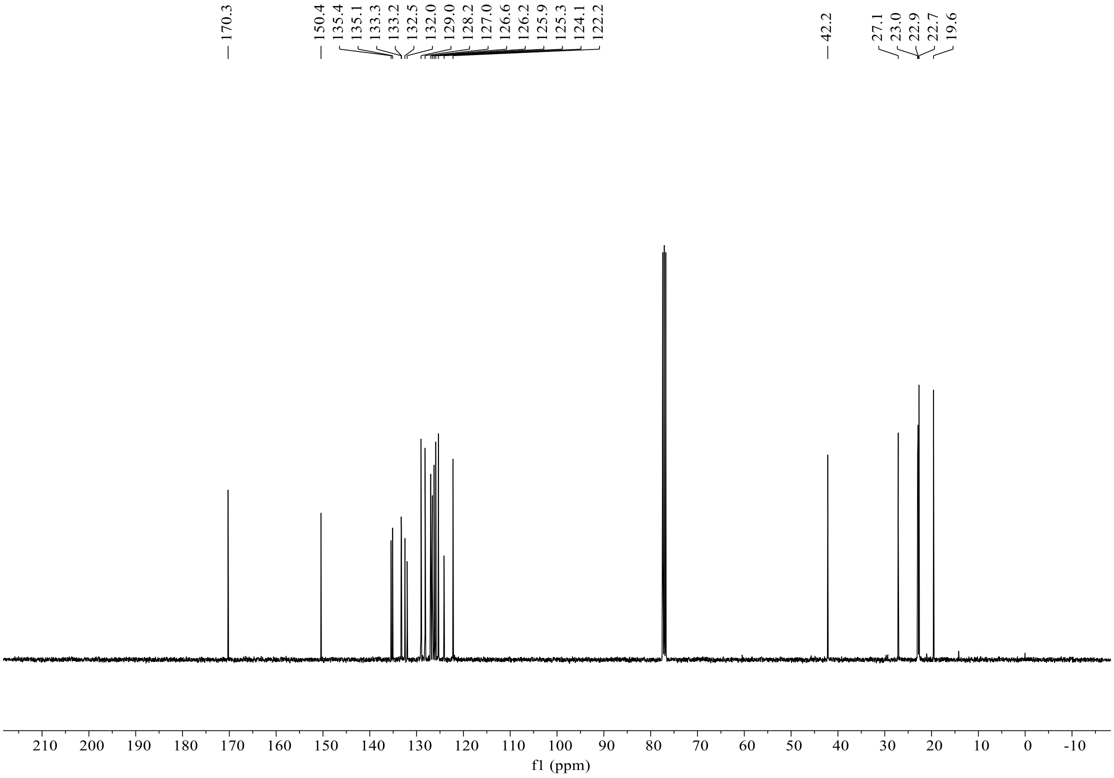

Supplementary Figure 225 HPLC spectra of racemic **20**

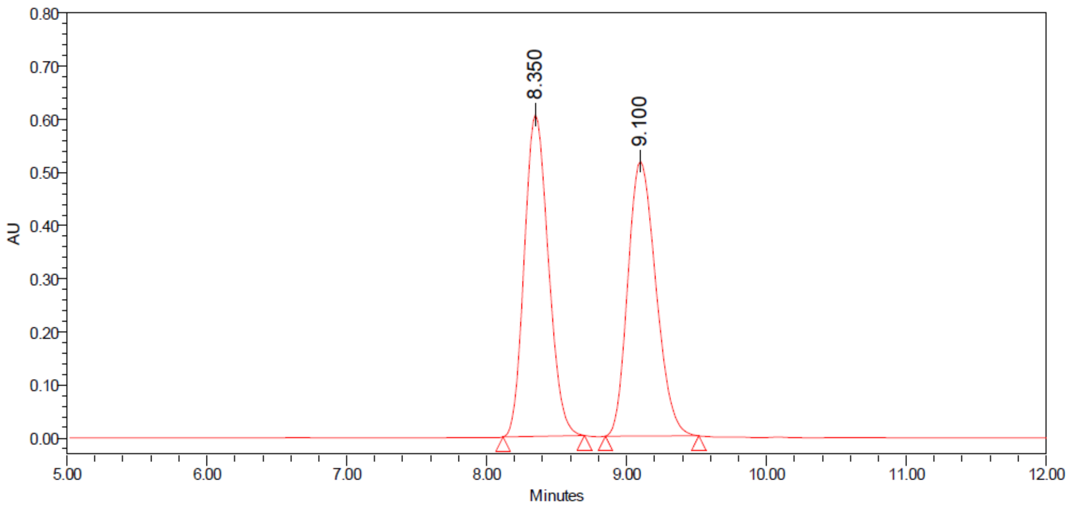

	RT	Area	% Area	Height
1	8.350	7219227	50.05	605777
2	9.100	7203880	49.95	517157

Supplementary Figure 226 HPLC spectra of (*S*)- **20**

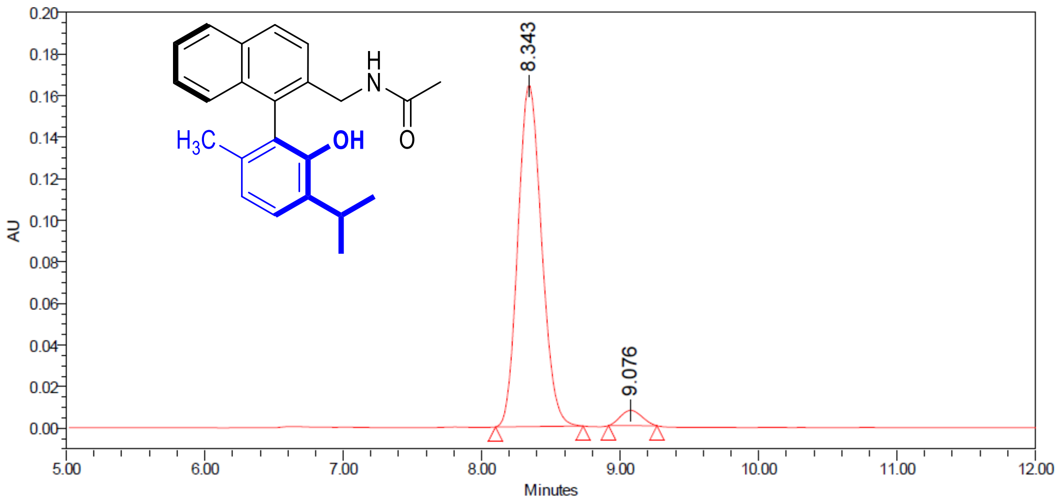

	RT	Area	% Area	Height
1	8.343	1946085	96.10	164532
2	9.076	78901	3.90	7356

Supplementary Figure 227 ^{1}H NMR (400 MHz, $CDCl_3$) of **23**

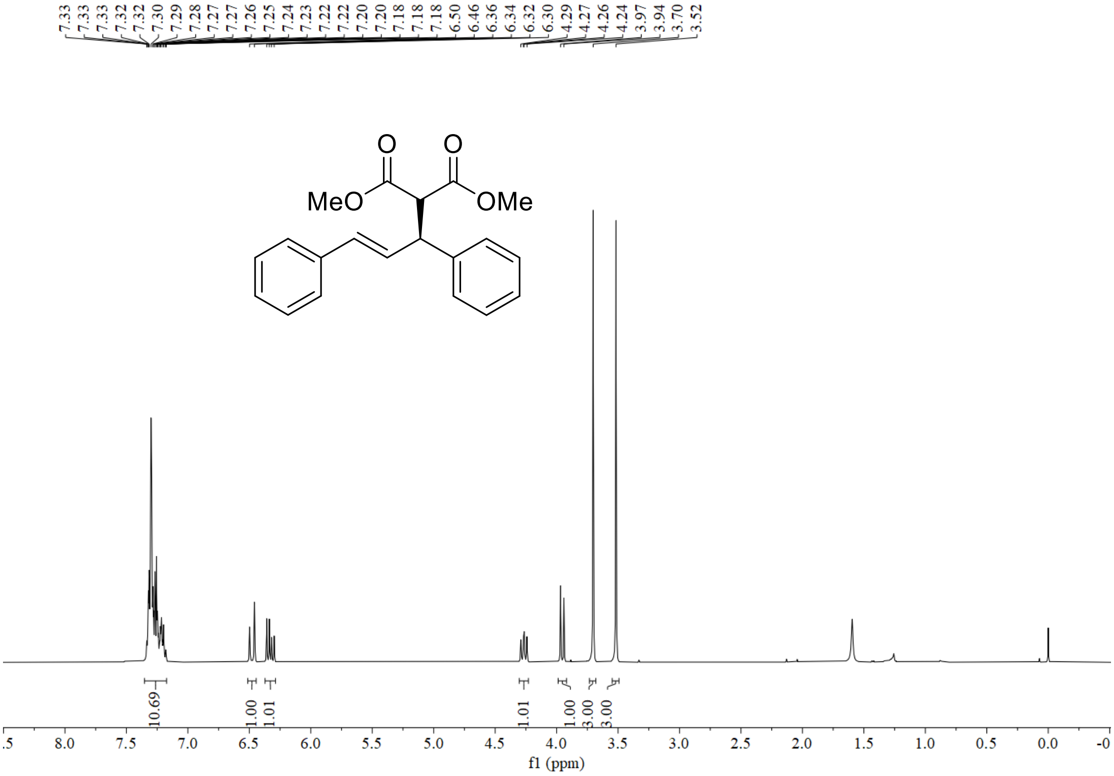

Supplementary Figure 228 ^{13}C NMR (400 MHz, $CDCl_3$) of **23**

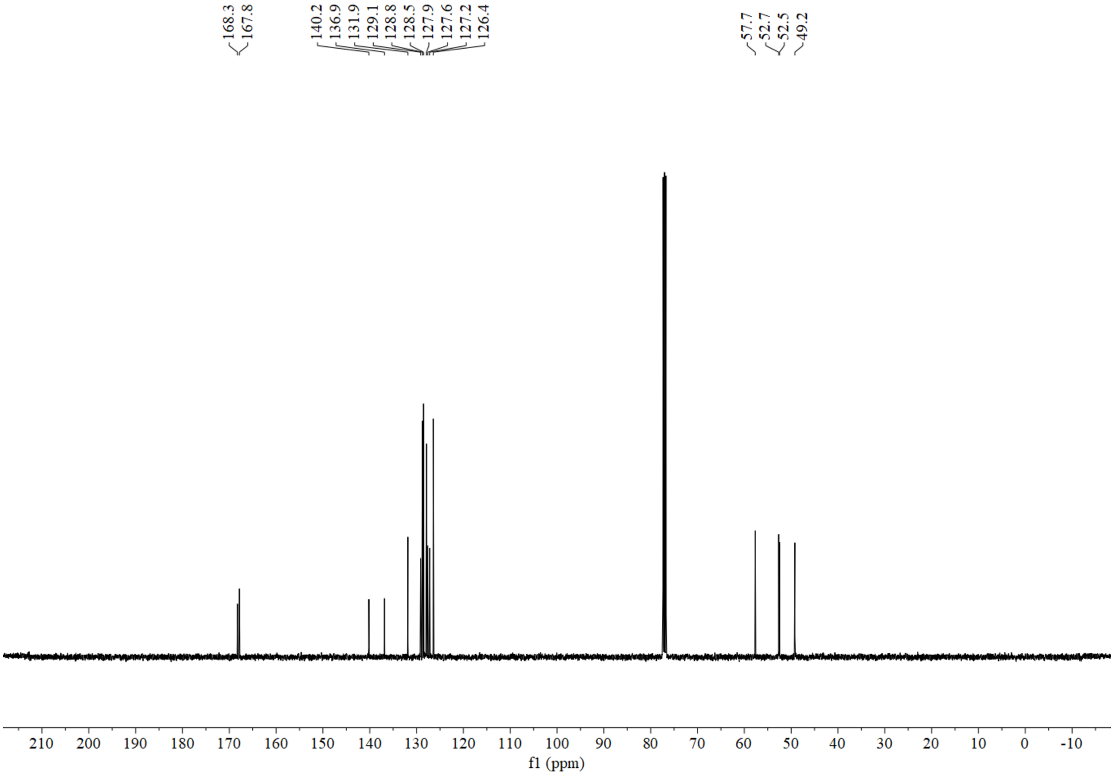

Supplementary Figure 229 HPLC spectra of racemic **23**

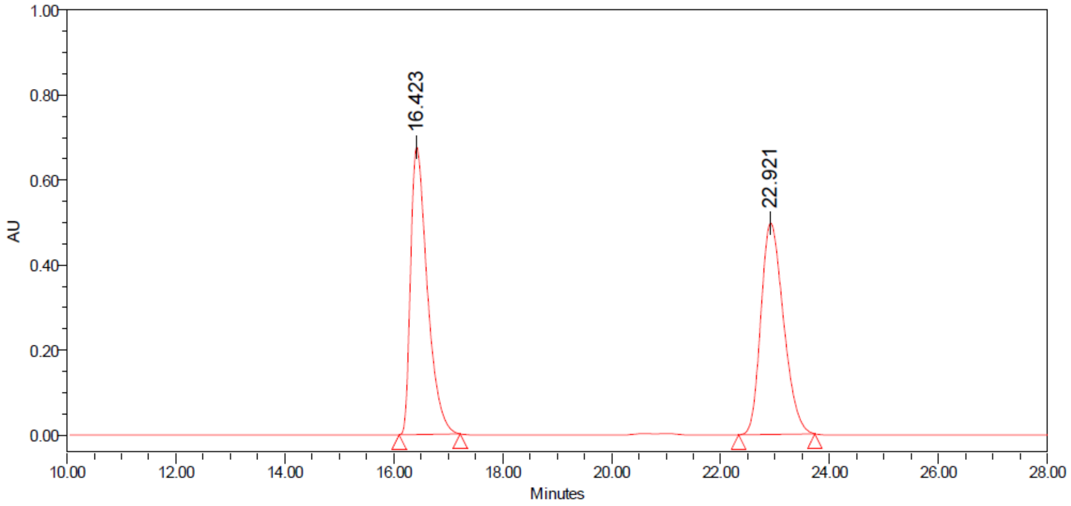

	RT	Area	% Area	Height
1	16.423	14362420	50.02	677461
2	22.921	14350665	49.98	497537

Supplementary Figure 230 HPLC spectra of (*S*,*E*)- **23**

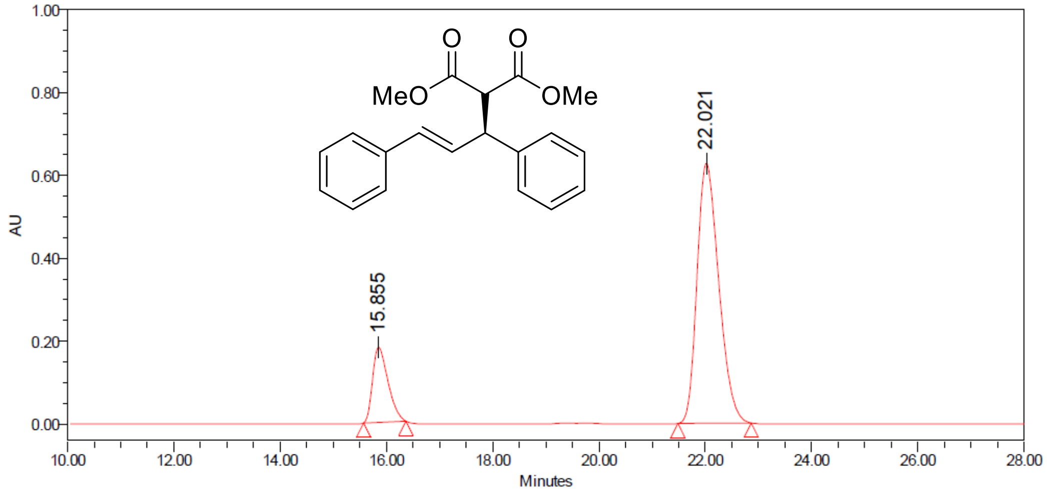

	RT	Area	% Area	Height
1	15.855	3658347	17.01	181373
2	22.021	17850602	82.99	627587

Supplementary Figure 231 ^{1}H NMR (400 MHz, $CDCl_3$) of **25**

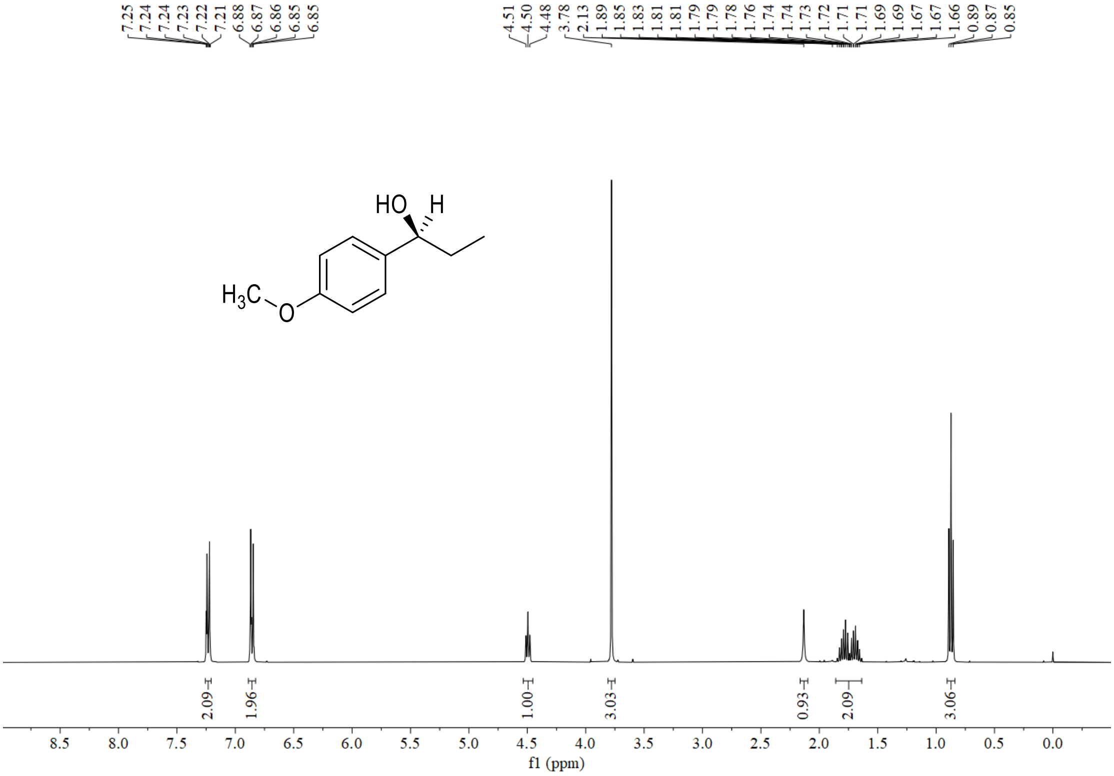

Supplementary Figure 232 ^{13}C NMR (400 MHz, $CDCl_3$) of **25**

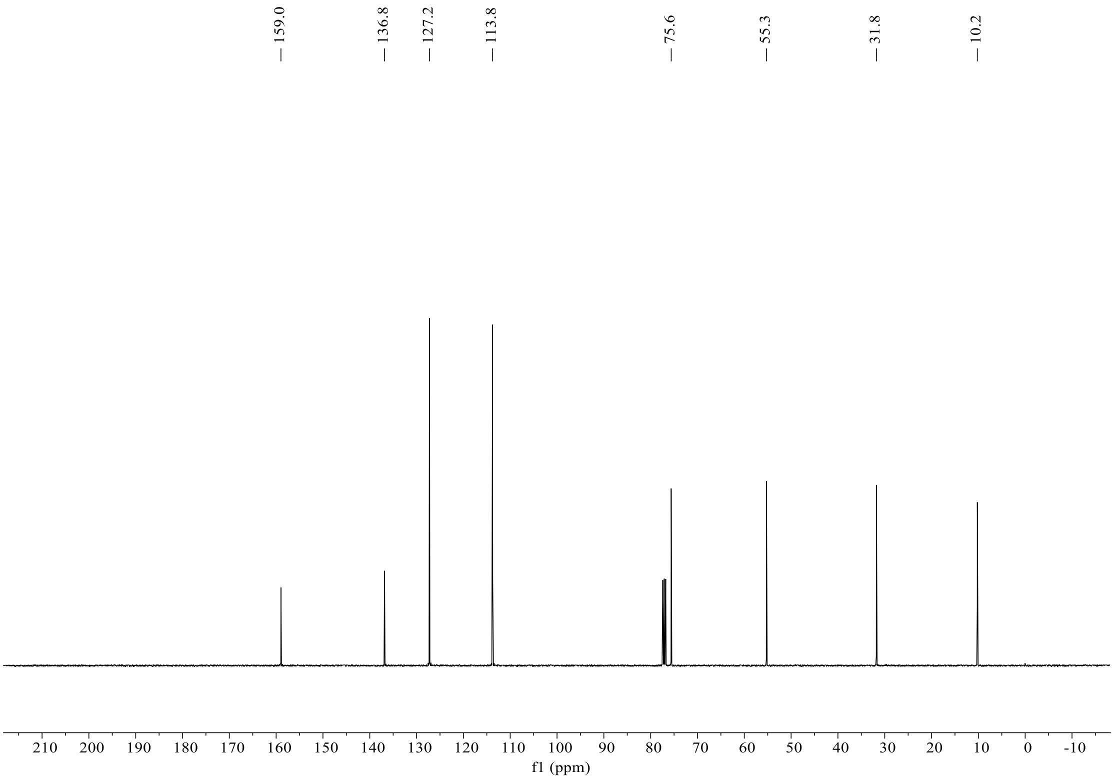

Supplementary Figure 233 HPLC spectra of racemic **25**

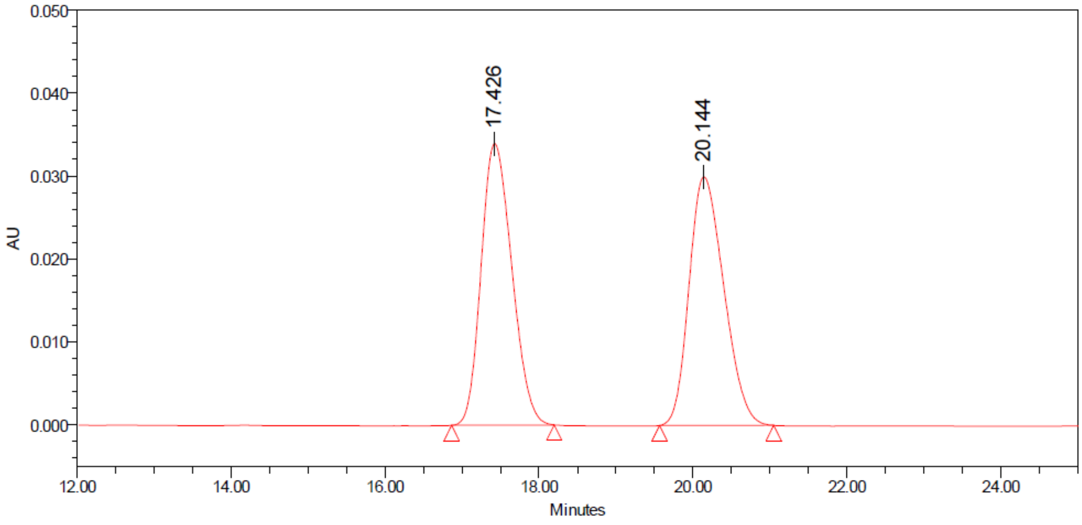

	RT	Area	% Area	Height
1	17.426	943506	50.01	33990
2	20.144	943211	49.99	30003

Supplementary Figure 234 HPLC spectra of (*S*)- **25**

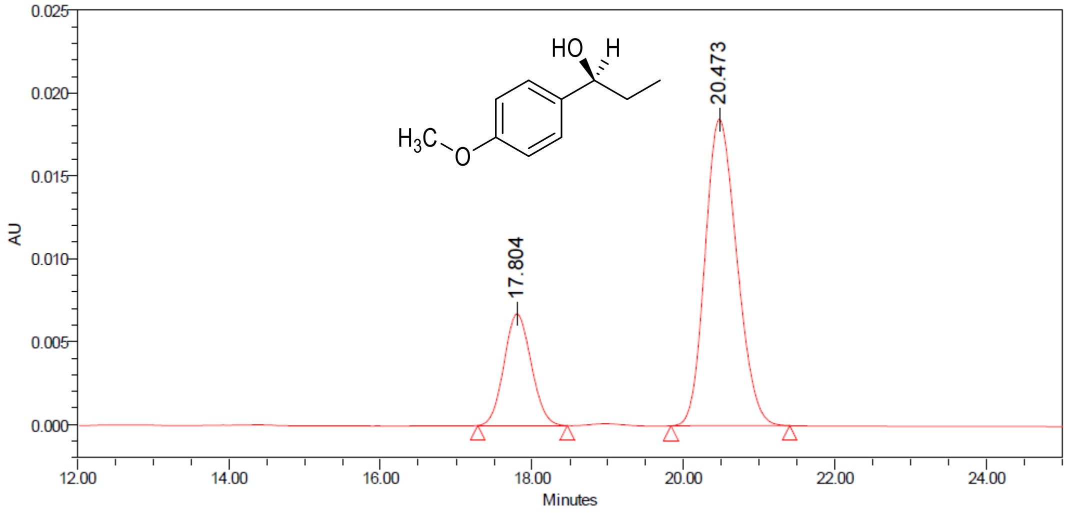

	RT	Area	% Area	Height
1	17.804	167776	23.75	6725
2	20.473	538719	76.25	18498

Supplementary Figure 235 ^{1}H NMR (400 MHz, $CDCl_3$) of **28**

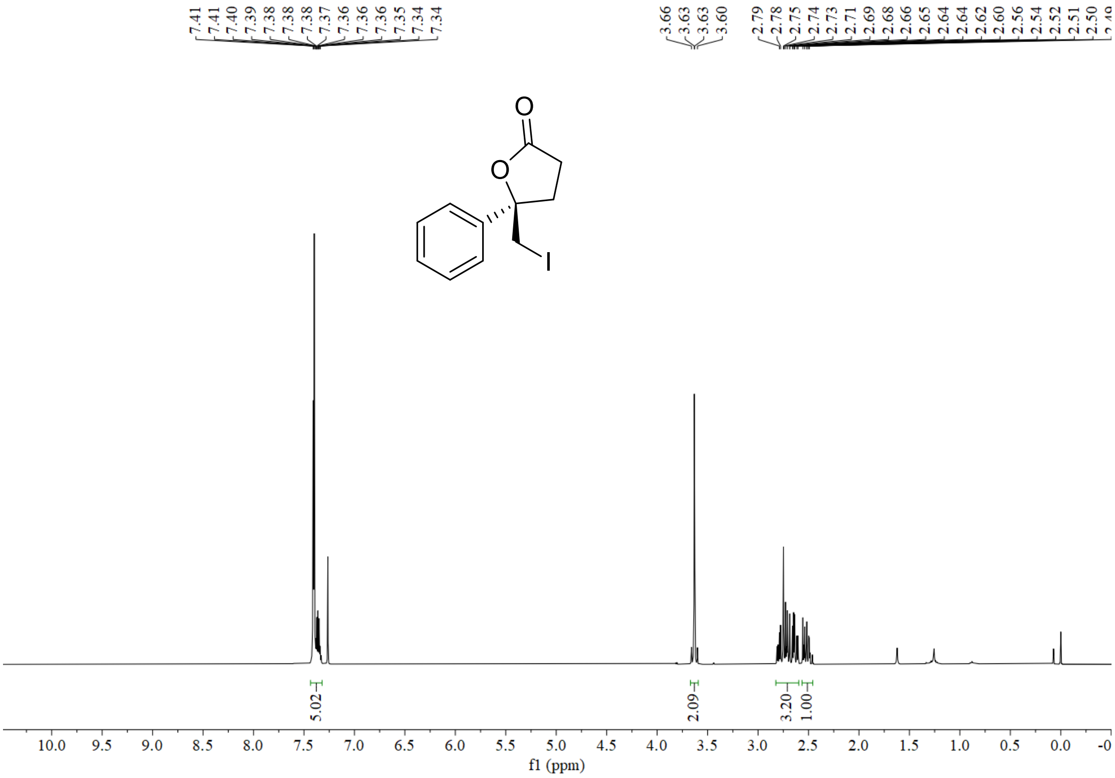

Supplementary Figure 236 ^{13}C NMR (400 MHz, $CDCl_3$) of **28**

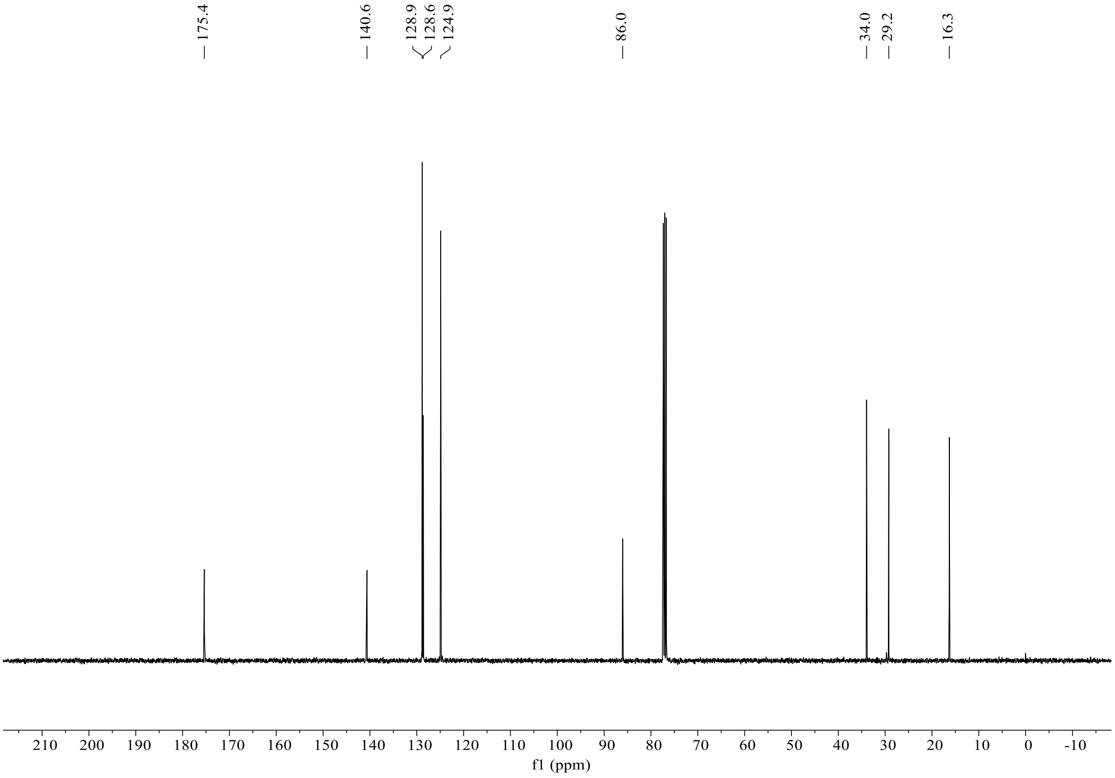

Supplementary Figure 237 HPLC spectra of racemic **28**

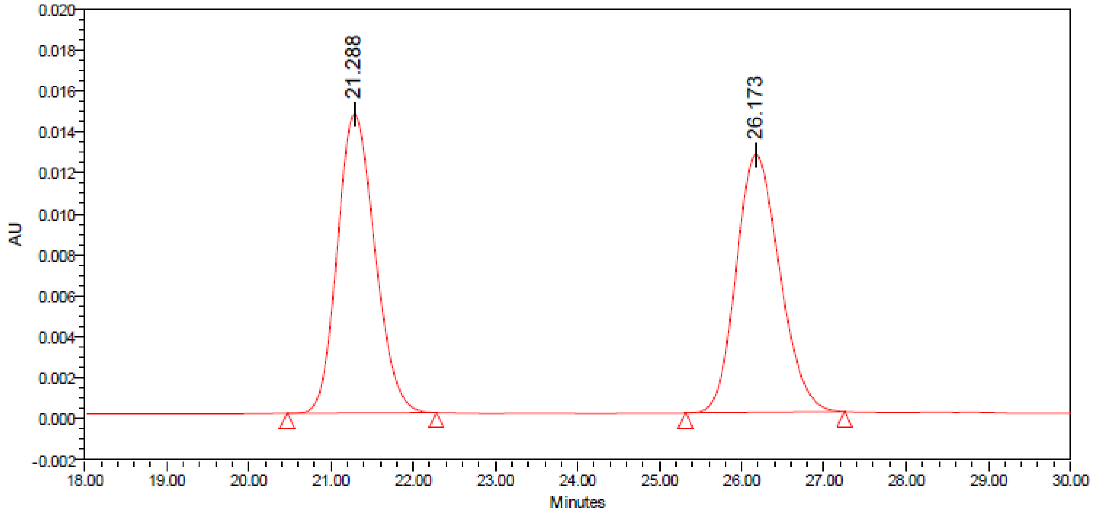

	RT	Area	% Area	Height
1	21.288	464049	50.02	14595
2	26.173	463605	49.98	12575

Supplementary Figure 238 HPLC spectra of (*R*)- **28**

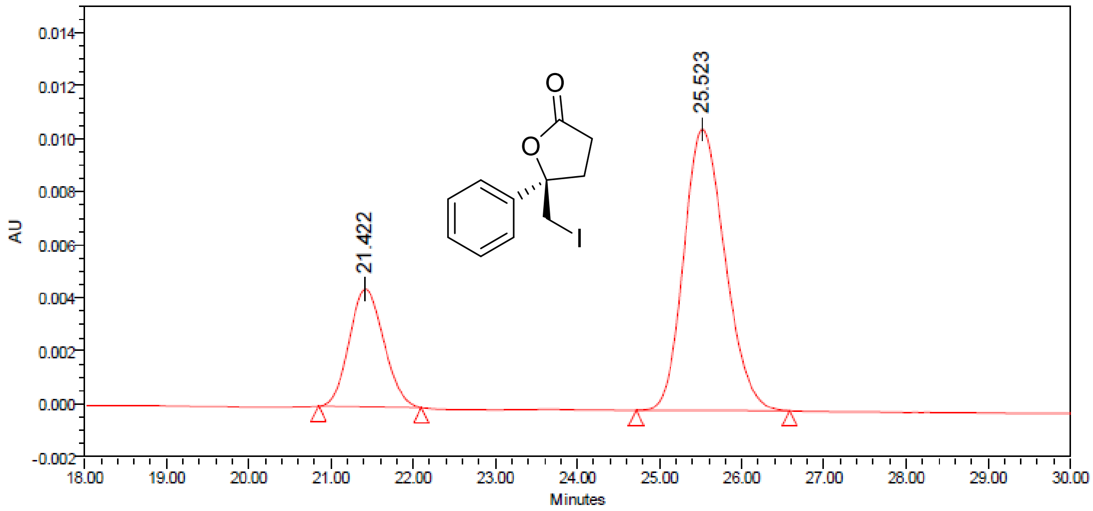

	RT	Area	% Area	Height
1	21.422	130005	25.99	4439
2	25.523	370282	74.01	10616

References:

Full reference for Gaussian software:

Gaussian 16, Revision A.01, Frisch, M. J.; Trucks, G. W.; Schlegel, H. B.; Scuseria, G. E.; Robb, M. A.; Cheeseman, J. R.; Scalmani, G.; Barone, V.; Mennucci, B.; Petersson, G. A.; Nakatsuji, H.; Caricato, M.; Li, X.; Hratchian, H. P.; Izmaylov, A. F.; Bloino, J.; Zheng, G.; Sonnenberg, J. L.; Hada, M.; Ehara, M.; Toyota, K.; Fukuda, R.; Hasegawa, J.; Ishida, M.; Nakajima, T.; Honda, Y.; Kitao, O.; Nakai, H.; Vreven, T.; Montgomery Jr., J. A.; Peralta, J. E.; Ogliaro, F.; Bearpark, M.; Heyd, J. J.; Brothers, E.; Kudin, K. N.; Staroverov, V. N.; Kobayashi, R.; Normand, J.; Raghavachari, K.; Rendell, A.; Burant, J. C.; Iyengar, S. S.; Tomasi, J.; Cossi, M.; Rega, N.; Millam, J. M.; Klene, M.; Knox, J. E.; Cross, J. B.; Bakken, V.; Adamo, C.; Jaramillo, J.; Gomperts, R.; Stratmann, R. E.; Yazyev, O.; Austin, A. J.; Cammi, R.; Pomelli, C.; Ochterski, J. W.; Martin, R. L.; Morokuma, K.; Zakrzewski, V. G.; Voth, G. A.; Salvador, P.; Dannenberg, J. J.; Dapprich, S.; Daniels, A. D.; Farkas, Ö.; Foresman, J. B.; Ortiz, J. V; Cioslowski, J.; Fox, D. J. Gaussian, Inc., Wallingford CT, 2016.

1. Guo, D., Zhang, J., Zhang, B. & Wang, J. Ruthenium-catalyzed atropoenantioselective synthesis of axial biaryls via reductive amination and dynamic kinetic resolution. *Org. Lett*. **20**, 6284-6288 (2018).

2. Frisch, M. J. et al. Gaussian 16, Revision B.01. 2016.

3. Zhao, Y. & Truhlar, D. G. The M06 suite of density functionals for main group thermochemistry, thermochemical kinetics, noncovalent interactions, excited states, and transition elements: Two new functionals and systematic testing of four Mo6-class functionals and 12 other function. *Theor. Chem. Acc.* **120**, 215–241 (2008).

4. Weigend, F. & Ahlrichs, R. Balanced basis sets of split valence, triple zeta valence and quadruple zeta valence quality for H to Rn: Design and assessment of accuracy. *Phys. Chem. Chem. Phys.* **7**, 3297–3305 (2005).

5. Weigend, F. Accurate coulomb-fitting basis sets for H to Rn. *Phys. Chem. Chem. Phys*. **8**, 1057–1065 (2006).

6. Grimme, S. Supramolecular binding thermodynamics by dispersion-corrected density functional theory. *Chem. Eur. J.* **18**, 9955–9964 (2012).

7. Luchini, G., Alegre-Requena, J. V., Funes-Ardoiz, I. & Paton, R. S. GoodVibes: Automated thermochemistry for heterogeneous computational chemistry data. *F1000Research,* **9**, 291 (2020).

8. Riplinger, C. & Neese, F. An efficient and near linear scaling pair natural orbital based local coupled cluster method. *J. Chem. Phys.* **138**, 034106 (2013).

9. Riplinger, C., Sandhoefer, B., Hansen, A. & Neese, F. Natural triple excitations in local coupled cluster calculations with pair natural orbitals. *J. Chem. Phys.* **139**, 134101 (2013).

10. Neese, F. The ORCA program system. *Wiley Interdiscip. Rev. Comput. Mol. Sci.* **2**, 73–78 (2012).

11. Neese, F. Software Update: The ORCA program system, Version 4.0. *Wiley Interdiscip. Rev. Comput. Mol. Sci.* **8**, e1327 (2018).

12. Neese, F., Wennmohs, F., Becker, U. & Riplinger, C. The ORCA quantum chemistry program package. *J. Chem. Phys.* **152**, 224108 (2020).

13. Guo, Y.; Riplinger, C.; Becker, U.; Liakos, D. G.; Minenkov, Y.; Cavallo, L.; Neese, F. An Improved Linear Scaling Perturbative Triples Correction for the Domain Based Local Pair-Natural Orbital Based Singles and Doubles Coupled Cluster Method [DLPNO- CCSD(T)]. *J. Chem. Phys.* **148**, 011101 (2018).

14. Kollmar, C. The role of energy denominators in self-consistent field (SCF) calculations for open shell systems", *J. Chem. Phys.* 105, 8204. (1996)

15. Halkier, A. et al. Basis-set convergence in correlated calculations on Ne, N_2 , and H_2O. *Chem. Phys. Lett.* **286**, 243–252 (1998).

16. Helgaker, T., Klopper, W., Koch, H. & Noga, J. Basis-set convergence of correlated calculations on Water. *J. Chem. Phys.* **106**, 9639–9646 (1997).

17. Halkier, A., Helgaker, T., Jørgensen, P., Klopper, W. & Olsen, J. Basis-set convergence of the energy in molecular hartree-fock calculations. *Chem. Phys. Lett.* **302**, 437–446 (1999).

18. Dunning, T. H. Gaussian basis sets for use in correlated molecular calculations. I. The atoms boron through neon and hydrogen. *J. Chem. Phys.* **90**, 1007–1023 (1989).

19. Woon, D. E. & Dunning, T. H. Gaussian basis sets for use in correlated molecular calculations. III. The atoms aluminum through argon. *J. Chem. Phys.* **98**, 1358–1371 (1993).

20. Woon, D. E. & Dunning, T. H. Gaussian basis sets for use in correlated molecular calculations. V. Core-valence basis sets for boron through neon. *J. Chem. Phys.* **103**, 4572–4585 (1995).

21. Kendall, R. A.; Dunning, Jr., T. H.; Harrison, R. J. Electron affinities of the first-row atoms revisited. Systematic basis sets and wave functions. *J. Chem. Phys.* **96**, 6796 (1992)

22. Woon, D. E. & Dunning, T. H. *J.* Gaussian basis sets for use in correlated molecular calculations. III. The atoms aluminum through argon. *Chem. Phys.* **98**, 1358 (1993).

23. Stoychev, G. L.; Auer, A. A.; Neese, F. Automatic Generation of Auxiliary Basis Sets. *J. Chem. Theory Comput.* **13**, 554, (2017).

24. Provasi, P. F.; Aucar, G. A.; Sauer, S. P. A. The effect of lone pairs and electronegativity on the indirect nuclear spin–spin coupling constants in CH2XCH2X (X=CH2,(X=CH2, NH, O, S): *Ab initio* calculations using optimized contracted basis sets. *J. Chem. Phys.* **115**, 1324 (2001).

25. Marenich, A. V., Cramer, C. J. & Truhlar, D. G. Universal solvation model based on solute electron density and on a continuum model of the solvent defined by the bulk dielectric constant and atomic surface tensions. *J. Phys. Chem. B* **113**, 6378–6396 (2009).

26. Schrödinger, L. The PyMOL molecular graphics development component, *Version 1*. **8**, (2015).

27. Sun, F. et al. A Combined experimental and computational study of NHC-promoted desulfonylation of tosylated aldimines. *Org. Chem. Front.* **7**, 578–583 (2020).

28. Bannwarth, C., Ehlert, S. & Grimme, S. GFN2-xTB - An accurate and broadly parametrized self-consistent tight-binding quantum chemical method with multipole

electrostatics and density-dependent dispersion contributions. *J. Chem. Theory Comput.* **15**, 1652–1671 (2019).

29. Pracht, P. & Grimme, S. Calculation of absolute molecular entropies and heat capacities made simple. *Chem. Sci.* **12**, 6551–6568 (2021).

30. Grimme, S. Exploration of chemical compound, conformer, and reaction space with meta-dynamics simulations based on tight-binding quantum chemical calculations. *J. Chem. Theory Comput.* **15**, 2847–2862 (2019).

31. Pracht, P., Bohle, F. & Grimme, S. Automated exploration of the low-energy chemical space with fast quantum chemical methods. *Phys. Chem. Chem. Phys.* **22**, 7169–7192 (2020).

32. Cezar, H. M. Clustering traj https://github.com/hmcezar/clustering-traj. Accessed 07 Aug 2021.
